# AI-Discovered Cognitive Models Reveal Novel Insights into Human and Animal Learning

**DOI:** 10.64898/2026.05.18.725921

**Published:** 2026-05-21

**Authors:** Daniel Kasenberg, Pablo Samuel Castro, Maria K. Eckstein, Noémi Éltető, Will Dabney, Caroline Wang, Martin Engelcke, Rishika Mohanta, Aparna Dev, Matthew M. Botvinick, Nenad Tomasev, Glenn C. Turner, Vincent Costa, Nathaniel D. Daw, Kimberly L. Stachenfeld, Kevin J. Miller

**Affiliations:** 1 Google DeepMind.; 2 Janelia Farm Research Campus, Howard Hughes Medical Institute.; 3 Laboratory of Neurophysiology and Behavior, The Rockefeller University.; 4 Emory National Primate Research Center and Department of Psychiatry and Behavioral Sciences, Emory University.; 5 Princeton Neuroscience Institute and Department of Psychology, Princeton University.; 6 Center for Theoretical Neuroscience, Columbia University.; 7 Sainsbury Wellcome Centre, University College London.

## Abstract

Scientific models are widely used across the natural sciences as an interface between scientific theories and empirical data [1]. Such models play a key role, for example, in the study of human and animal learning, where they express algorithmic hypotheses and relate them to psychology and neuroscience data [2, 3]. These models are traditionally handcrafted by expert researchers based on existing theory or new insights. Such handcrafted models, however, are now known to fall short of capturing the full richness of behavior, even in their narrow domains [4–7]. An alternative data-driven approach has emerged, seeking to discover new insights by fitting and interpreting flexible models [8–11]. However, these tools require substantial human effort to derive insight from data, and it has been unclear how to discover new ideas from data efficiently. Here, we present DataDIVER, a general approach for automatically discovering computational models from data, and demonstrate that these models surface novel mechanistic insights into human and animal learning. Our approach delivers models that take the form of short computer programs, which are optimized both to fit data well and to be simple. These programs explicitly connect with existing theoretical frameworks and are readily understandable by human scientists. They can also be used to make novel predictions, some of which we show are borne out in re-analysis of existing data. General-purpose tools for surfacing new ideas from data, especially in combination with the large datasets that are increasingly available in many fields, stand to dramatically accelerate scientific discovery.

Mechanistic computational models are an important tool in many sciences, allowing abstract theoretical ideas to be instantiated and tested quantitatively against data. In many domains, this has traditionally been a “theory-first” process, with models constructed based on existing theory and used as tools for exploring the implications of that theory [12, 13]. Recently, however, a new generation of data-driven model discovery tools has begun to invert this, enabling researchers to discover useful scientific models directly from data [9, 11, 14, 15]. The advent of strong and general-purpose generative AI in principle holds enormous potential for this practice, as machines can for the first time generate exactly the types of objects that human researchers use to express scientific models, including human-readable, literature-aware computer code. Recently, AI-optimized code has shown an impressive ability to propose statistical models and data processing pipelines [16, 17], and even identify unknown solutions to problems in mathematics and engineering [18, 19]. However, discovery in these domains is fundamentally unlike that in basic science, because progress in basic science requires surfacing novel insights into the underlying structure of the real-world processes.

A key open problem of the latter kind is identifying algorithms that humans and other animals use to learn from reward. Despite being one of the oldest questions in psychology [20, 21], it remains unsolved. Current computational approaches are heavily theory-driven, taking inspiration from reinforcement learning [22, 23] as well as Bayesian optimality [24, 25]. Recent work has demonstrated that these theory-driven models are decisively outperformed by blackbox predictive models in terms of quality-of-fit [4, 5, 26, 27]. This suggests that better mechanistic models very likely exist, but does not on its own suggest a way to discover them. Reward learning, like other problems in cognitive science, is particularly challenging as it involves the update over time of internal (“cognitive”) variables that are only indirectly reflected in observable behavior like choices. Model discovery in these contexts therefore requires inferring from data how many internal variables exist, how they are updated based on external input, and how they result in behavior.

Here, we present DataDIVER (Data-driven Discovery of Interpretable models Via Evolutionary Refinement), a tool for discovering scientifically interpretable symbolic models from data. DataDIVER relies on AlphaEvolve, a recently developed tool that has shown impressive performance on program optimization problems in mathematics and computer science by using LLMs to edit programs within an evolutionary algorithm that optimizes for a user-specified objective [19]. DataDIVER extends AlphaEvolve, allowing it to optimize programs based on both quality-of-fit to a dataset and on simplicity.

A challenge for the problem of automating model discovery is deciding on the target(s) of optimization, as scientifically useful models must meet two important criteria. The first is that they should capture data accurately, which in our setting can be quantified as predicting the observed data with high likelihood (“quality-of-fit”). While this is straightforward to quantify, it is difficult to optimize, and earlier attempts with LLM-based search techniques simpler than AlphaEvolve have not been able to match the performance of deep learning models at predicting learning behavior [28, 29]. The second is that they should convey human-interpretable insights about the data. While human interpretability is ultimately subjective, a close correlate is program complexity, for which a number of quantitative metrics have been proposed [30]. Furthermore, quality-of-fit often trades off against complexity metrics in practice, and there is no single answer to how much relative value to place on each. Different applications demand different tradeoffs between complexity and quality-of-fit, from simple abstract models that are “wrong but useful” [31] to detailed models that do not “surrender the adequate representation of a single datum of experience” [32].

Our approach to this tension is to optimize a set of models that each strikes a different balance between quality-of-fit and simplicity. We apply this approach to five datasets of learning behavior from a range of species and behavioral settings. We find that the best-fitting programs match the quality-of-fit of “blackbox” neural network models, which to our knowledge has never been achieved with purely symbolic models on learning behavior datasets [28, 29]. Perhaps unsurprisingly, these extremely well fitting programs are lengthy, redundant, complex programs that do not readily afford interpretation. The remaining spectrum of programs surfaces meaningfully novel insights in an accessible way, with the simplest models shedding light on the basic organization of learning behavior, and more complex programs revealing more detailed structure (Fig. 2a). Some of these discovered learning mechanisms suggested the presence of previously unknown patterns which we verified by reexamining the behavioral data. Broadly, these results showcase the usefulness of AI tools not just to predict behavior but also to explain it.

## 1 Data-driven optimization of programs

The DataDIVER pipeline consists of two program optimization stages, both of which are powered by AlphaEvolve [19]. AlphaEvolve is an evolutionary algorithm that uses a large language model (LLM) to modify (or “mutate”) programs as instructed in user-specified prompts. These programs are evaluated according to a user-specified “fitness” objective, with higher scoring programs preferentially sampled for further modification. Following Castro et al. [28], our programs implement a learning rule that takes in relevant information about the subject’s past experience (previous choice, previous reward, other information about the previous and current trial if relevant, and an “agent state” maintaining memory) and output a probabilistic prediction about the animal’s next choice. The programs have individual parameters which are fit separately to data from each subject, and validated on a held-out portion of the subject’s data using two-fold cross-validation. These parameters might include learning rates, exploration parameters, or whatever individual parameters DataDIVER decides to implement. We run three separate instances of this evolutionary process for each dataset, considering half of the subjects in the dataset. The remaining subjects are held-out for use in evaluating the discovered models. Programs are implemented in Python using JAX [33] so that they are differentiable, allowing efficient parameter optimization. We run three independent runs of both DataDIVER stages.

**Fig. 1:**
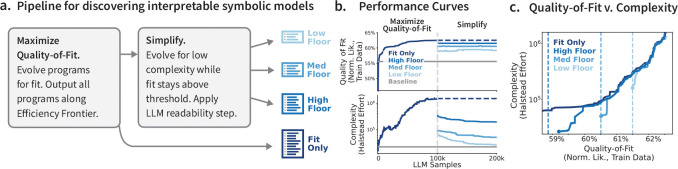
Overview of DataDIVER Pipeline. **(a)** Our pipeline consists of two stages: in the *Maximize Quality-of-Fit* stage, AlphaEvolve is used to optimize programs that maximize the normalized likelihood of the observed data. In the *Simplify* stage, a second AlphaEvolve run minimizes Halstead complexity metrics while keeping the quality-of-fit score above a floor, after which an LLM-powered code refactor improves human readability. These stages result in four programs: a very complex “Fit only” program emerging from stage one, and three simplified programs that strike different balances of complexity and quality-of-fit. **(b)** Performance curves illustrating likelihood and complexity scores over the course of evolution. In the first stage, both likelihood and complexity increase; in the second stage, likelihood is maintained above the specified floor while complexity is reduced. **(c)** Quality-of-fit versus complexity for programs that strike efficient tradeoffs of fit and complexity. Darkest line depicts programs generated during the *Maximize Quality-of-Fit* stage; lighter lines depict programs generated during the *Simplify* stages for different floors (vertical dashed lines). Each *Simplify* stage improves the frontier (i.e. identifying simpler programs that satisfy each floor).

In the first stage (*Maximize Quality-of-Fit*; Figure 1A), programs are optimized to maximize quality-of-fit on the behavioral data without regard to complexity(see Supplement C.1). This results in a large set of programs that vary widely both in quality-of-fit and in complexity. We quantify complexity using Halstead effort [30], a heuristic measure that uses the number of variables and operations in the program to estimate how much time and effort a human programmer would need to understand it (Figure 1C). We select for further consideration the single program with the greatest quality-of-fit.

The second stage (*Simplify*; Fig. 1A) aims to produce simpler programs which only modestly sacrifice quality-of-fit. We run three separate instances of this stage per dataset, each with a different quality-of-fit floor that programs cannot fall beneath. This enabled us to recover a set of programs that optimize for different tradeoffs between complexity and quality-of-fit. The stage is initialized with all programs from the *Maximize Quality-of-Fit* stage that efficiently trade off quality-of-fit and complexity: that is, programs on the “Pareto frontier” for which no discovered program improves one objective without sacrificing the other (Fig. 1c). Each *Simplify* stage succeeds if it succeeds in pushing this frontier, which they do by identifying simpler models that maintain quality-of-fit. In this stage, the LLM prompt encourages simplifying the programs (see Supplement C.2), and the fitness function rejects programs that fall beneath the quality-of-fit floor but otherwise selects programs based on their simplicity. We select for further consideration the single program with the greatest simplicity (smallest Halstead effort) from each *Simplify* run.

Minimizing Halstead complexity encourages the programs to implement simpler mechanisms, but ignores important human readability considerations like documentation, organization, and consistency. We use an LLM (Gemini 2.5 Pro) to rewrite each simplified program to improve readability without changing the program’s function (prompts in Supplement C.3). The final output of DataDIVER consists of the best-fitting programs from the *Maximize Quality-of-Fit* stage and the rewritten programs from the *Simplify* stage (see Figure 4 for an example program; additional examples for each dataset are found in Supplement A). In addition to these automatically-generated programs, we also manually constructed a single “synthesis” program for each dataset combining the key human-interpretable mechanisms discovered for that dataset.

## 2 Fit-only programs are strong predictive and generative models

**Fig. 2:**
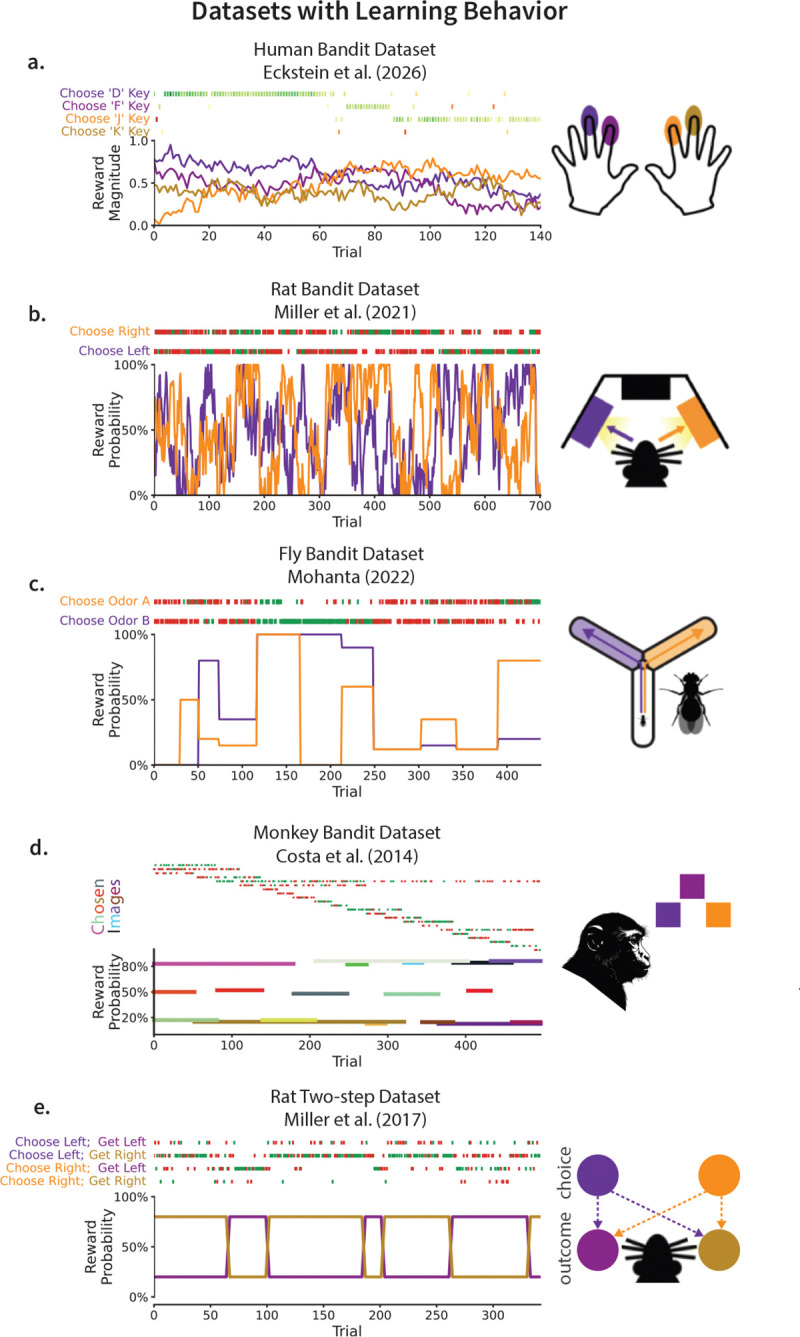
Illustration of datasets. Each dataset contains human or animal behavior in different reward-guided learning experiments. In each dataset, subjects chose among discrete choices to receive dynamically changing rewards, with each line above denoting the reward structure for each choice over time. The datasets vary in the subject species, the way the animal experiences stimuli and demonstrates choices, the number of choices available, and the latent reward structure.

We apply DataDIVER to five datasets containing humans and other animals performing diverse reward learning tasks: *Human Bandit* [10], *Rat Bandit* [8], *Fly Bandit* [34], *Monkey Bandit* [35, 36], *Rat Two-step* [37] (Figure 2). In each of these tasks, a subject repeatedly selects one of several available actions and receives rewards whose magnitude or probability depend on the chosen action and vary over time. Each dataset contains a large number of sessions, making them suitable for data-driven model discovery. Each has also been the focus of intensive previous computational modeling efforts which have yielded a strong handcrafted baseline model [8, 10, 34, 35, 38, 39], enabling us to robustly benchmark DataDIVER-discovered models.

**Fig. 3:**
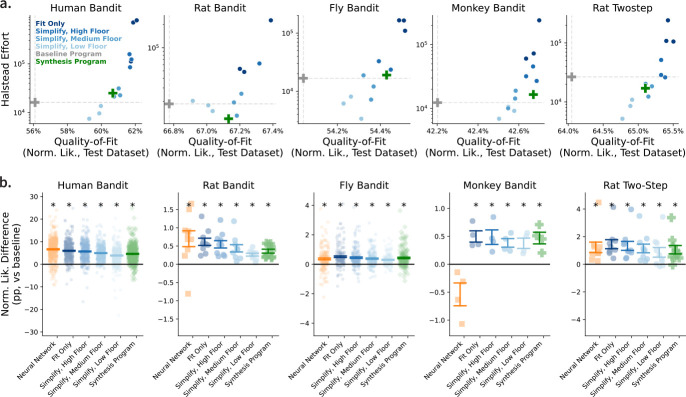
Evolved programs fit data well and trade off quality-of-fit and complexity. **(a)** Quality-of-fit (normalized likelihood on held-out data) against complexity (Halstead effort) for each DataDIVER program, the state-of-the-art handcrafted baseline program from the literature, and the synthesis program, plotted for each dataset. Discovered programs achieve higher quality-of-fit than the baseline program. Programs simplified to lower floors tend to have lower quality-of-fit. **(b)** Quality-of-fit difference compared to the baseline model, plotted separately for each participant, for the best-fitting program at each floor (see B14 for all programs). Points indicate the quality-of-fit difference between the indicated model and the baseline model for individual subjects. Error bars indicate standard errors over subjects. Asterisks indicate significant differences from the handcrafted baseline model (*p <* 0*.*05, t-test).

First, we consider the best-fitting “fit-only” programs from the first *Maximize Quality-of-Fit* stage of DataDIVER by evaluating their quality-of-fit on the held-out subjects for each dataset using trial-normalized likelihood [2]. Each program significantly outperformed the baseline model for its dataset (average difference in normalized likelihood of 4.1 percentage points, all p *<*0.02 on fifteen separate t-tests over subjects; Figure B14). These differences were consistent over three independent runs of DataDIVER (average difference in normalized likelihood was 5.8 percentage points for the *Human Bandit* dataset, p=0.0004, t-test over three DataDIVER runs; *Rat Bandit* 0.50pp p=0.01; *Fly Bandit* 0.51pp, p=0.0002; *Monkey Bandit* 0.47pp, p= 0.002; *Rat Two-step* 1.4pp, p=0.0005; Figure 3A). We also compare our programs to generic recurrent neural networks (RNNs; see Section 7.9), which have been widely found to outperform symbolic models when applied to large behavioral datasets [5, 9, 10, 26, 28]. We find that the fit-only models discovered by DataDIVER overall perform similarly to RNNs (average performance difference 0.05pp, p=0.88, t-test across datasets). Considering individual datasets, the fit-only programs significantly outperformed the best RNNs on three datasets (*Fly Bandit*, average difference 0.14pp, p=0.003, t-test across DataDIVER seeds; *Rat Two-step*, average difference 0.17pp, p=0.03; and *Monkey Bandit*, the only dataset for which the RNN underperformed the baselines, likely due to smaller dataset size, average difference 1.0pp, p=0.0003) and significantly underperformed them on one (*Human Bandit*, average difference 0.9pp, p=0.02).

In order to test whether the discovered programs generate behavior that matches the characteristics of the real data [3, 40], we simulated choices in an environment matched to the experimental conditions to create an artificial dataset (see Section 7.10 for details). We then computed a trial-history regression model for the real and artificial datasets [41, 42] to quantify the effects of past trials’ outcomes on current choice (see Section 7.11). We find that behavior generated by our AI-discovered programs closely matches the patterns seen in the real datasets, while datasets generated by the handcrafted baseline models fail to capture many features (Figures A2, A6, A9, A11, A13). Taken together, these results indicate that the fit-only models discovered by DataDIVER are strong predictive and generative models, outperforming handcrafted baseline models and closing most or all of the gap with blackbox neural networks.

## 3 “Simplified” programs trade off complexity and quality-of-fit

**Fig. 4:**
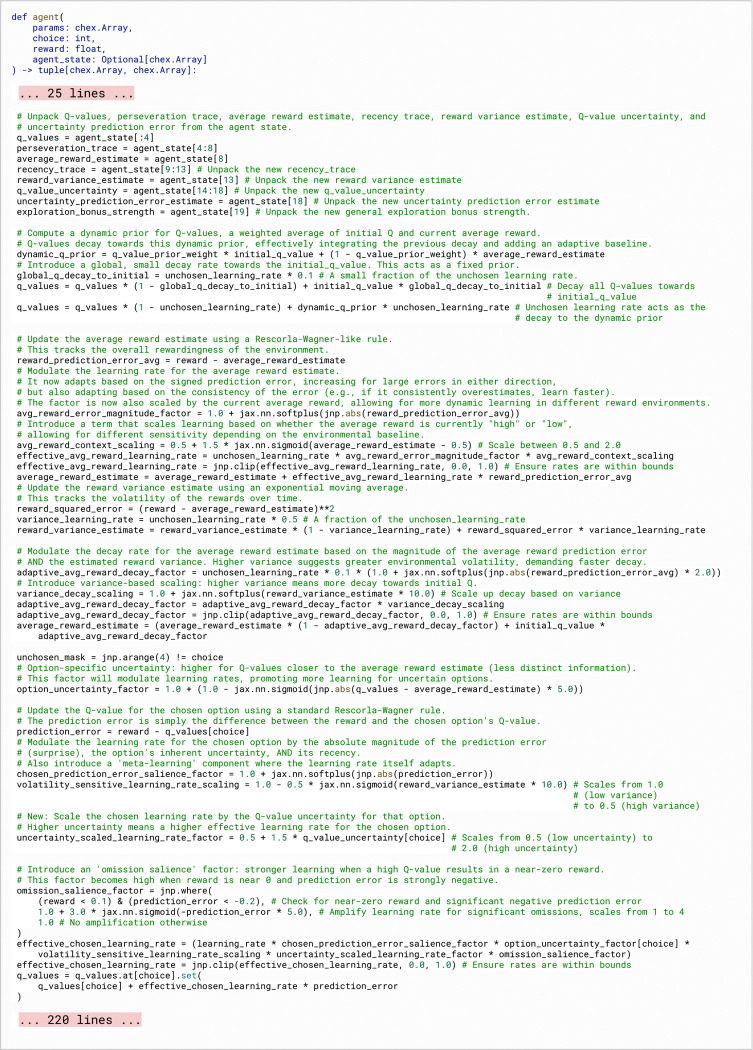

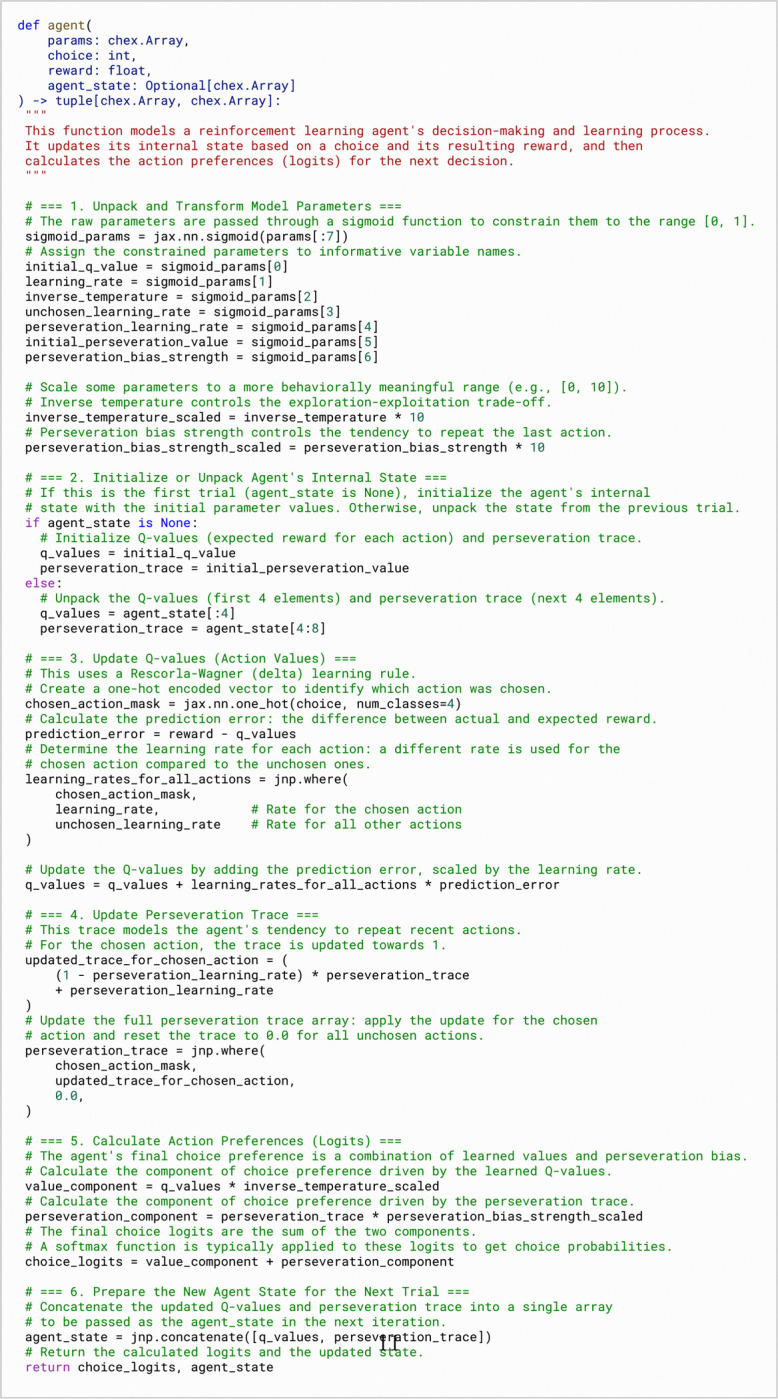
(a) Example Discovered Fit-only Program. This is a representative “fitonly” program outputted by the *Maximize Quality-of-fit* stage. It shows certain promising characteristics–informative variable names and recognizable computational motifs–but the program is quite complex (20 state variables are defined), the operations are complex and highly nonlinear, and the mechanisms are partially overlapping (e.g. multiple variations on reward prediction error-driven updates). Program is truncated and adjusted to fit on one page; it is otherwise unedited. We note that jnp = jax.numpy; programs were implemented in jax to permit gradient-based parameter optimization. **(b) Example Discovered Simplified, Low-floor Program.** This is an example program outputted by the *Simplify* stage (*Human Bandit*, low floor, run 1). It shows characteristics seen across other discovered programs: interpretable variable names, single computations per line, and comments describing hard-to-parse operations and organizing the computational structure. It is unedited except for adjustments to the spacing for compactness.

The programs that emerge from the *Maximize Quality-of-Fit* stage are strong predictive and generative models, but they are very complex (Figure 4A, see full code for one program in A.1.6). We see that the program is thoroughly commented and includes recognizable variable names and mechanisms; however, it is highly repetitive and overwhelmingly long, and individual operations are very complex. These programs exceed the baseline programs in terms of Halstead effort (by a factor of 12.7 ± 3.7; mean ± standard error over all programs), number of lines of code (by a factor of 2.7 ± 0.6), and number of state variables (by a factor of 3.2 ± 0.4). This complexity substantially reduces their appeal to human scientists, as complex models are difficult to interpret and often considered to be less plausible as mechanistic models.

The *Simplify* stage of our pipeline (Figure 1A) succeeded in generating substantially simpler programs: the high, medium, and low floor programs respectively had 30 ± 6%, 14 ± 2.5%, and 9.8 ± 2.8% the Halstead effort of the fit-only program (Figure 3A). Strikingly, programs simplified to the low floor were simpler even than the handcrafted baseline programs, having on average about half the Halstead effort (59 ± 0.1 %). The simplification procedure did affect quality-of-fit, with programs simplified to a lower quality-of-fit floor having lower fit quality (*Human Bandit*: p=0.0001; *Rat Bandit* p=0.005; *Fly Bandit*: p=0.0001; *Monkey Bandit*, p=0.003; *Rat Two-step* p=0.0001; Page’s trend test [43] over DataDIVER seeds and quality-of-fit floors). Nevertheless, the vast majority of the AI-discovered models significantly outperformed our handcrafted benchmark models in terms of quality-of-fit (54/58 evolved programs significantly better than baseline at *p <* 0*.*05, paired t-tests over subjects; Figure B14).

Qualitatively, these simplified programs were surprisingly readable, and far more accessible than the fit-only programs. Figure 4B shows an example low-floor program (additional programs are found in Supplement A). The discovered programs use literature-aware variable names like “learning rate”, “q-values”, and “perseveration” as well as mechanisms like error-driven learning and forgetting by decay. Unlike the fit-only program in Figure 4A, the entire program can be viewed on a single page, and does not contain obviously redundant code or unnecessarily complex operations. The simplified programs use comments to organize the code into numbered sections, which can be browsed as a “table of contents”, and additional comments describe complex steps. Some of these computations are unusual (e.g. the update on unchosen action values, the forgetting update on the perseveration trace), but the mechanisms can still be readily understood. Furthermore, a similar organization is applied across the different discovered programs for each dataset, allowing straightforward comparison and synthesis. In general, we found the low-floor programs to be readily understandable, the medium-floor programs could be fully understood with moderate effort, and the high floor programs often could not be fully understood, though it was nevertheless typically possible to glean testable insights from inspecting them.

## 4 Evolved models surface novel mechanistic insights

Several themes emerged across the discovered programs. First, although the programs are written in familiar, literature-aware vocabulary, there was often surprising structure to how internal variables were organized and updated and how they mapped onto decisions. One notable tendency across species and complexity levels was to introduce additional or different cognitive variables than those assumed by previous models. Such reorganizations challenge the prior interpretations of the baseline models’ putative subcomponents; for example, our discovered models break assumptions that novelty and reward preference are folded into a single common-currency reward expectancy (*Monkey Bandit*) or that learning rules update a scalar preference for one choice over the other rather than per-choice statistics (*Rat Bandit*, *Rat Two-step*). Second, discovered programs often included nonstationary, nonlinear modulation of various operations or parameters (e.g. softmax temperatures or decay targets). This potentially captures the subjects’ implicit adaptation to statistics of the task like the average reward level, a type of adaptive learning that has often been neglected when studying any individual dataset. Notably, a number of discovered motifs suggested novel insights about the data that were supported by reanalysis–a striking instance of AI leading to a novel data-driven discovery (e.g. Figure 7).

**Fig. 5:**
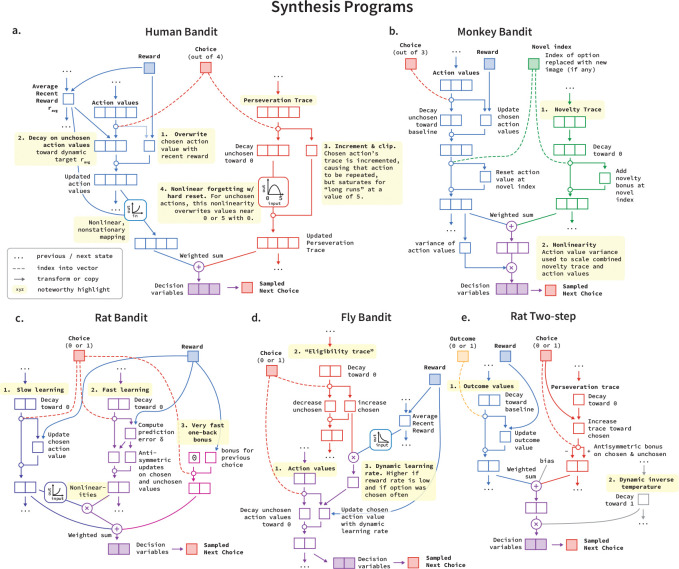
Human expert synthesis of mechanisms in evolved programs. : Each diagram illustrates the logic of the synthesis programs. Distinct modules are denoted with different colors.

**Fig. 6:**
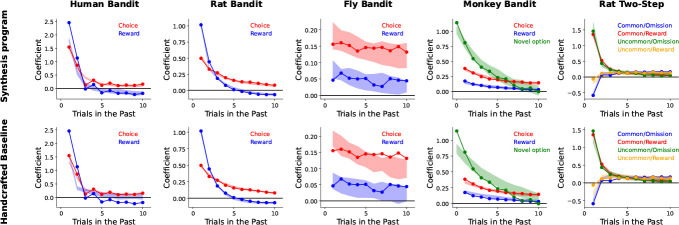
Synthesis programs are strong generative models. We generate artificial data using the synthesis programs, which aggregate information from across the discovered programs, and compute trial-history regression models, which measure the effects of trial variables (like choices and rewards) from earlier trials on the current choice. Coefficients estimated from the real experimental data are shown as dots and lines; and compared to the range given by the same analyses applied to simulations (where shaded patches show the 95% prediction intervals over artificial datasets). The datasets generated by our synthesis programs (top) closely match the patterns seen in the real datasets, often qualitatively better than the original handcrafted baselines (bottom). Lagged regression plots for all models can be found in Supplement A.

We briefly summarize insights from the DataDIVER discovered programs below (see Supplement A for more detail). To simplify visualization and verification of the many programs produced by DataDIVER, we also consolidated the different programs for each dataset into a single synthesis programs, which are depicted in Figures 5 and 6. Discovered motifs were excluded from the synthesis program if removing them had no effect on quality-of-fit, and prioritized if they evidently contributed to their program’s predictive or generative performance (see Section 7.14 for more detail). All insights discussed below are included in the synthesis program.

### 4.1 Human Bandit

**Fig. 7:**
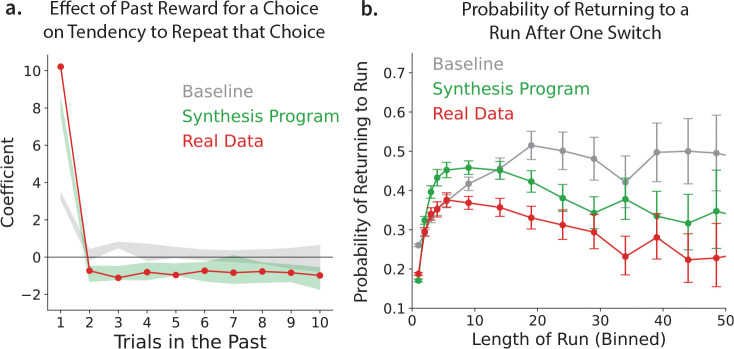
Inspecting discovered programs reveals unexpected patterns in *Human Bandit* data. **(a)** Trial-lagged regression analysis showing the effect (coefficient, positive promotes repeating) of being rewarded for a choice in a previous trial (1–10 trials ago) on tendency to repeat that choice. We note two features of interest predicted by our analysis of discovered programs. First, because previous reward overwrites the chosen action value, we predict that the immediately preceding trial should have an outsized effect, which we see for the real data and synthesis program’s generated data but not baseline program’s. Second, since the unchosen action values are slowly updated toward the recent average reward and chosen action values are overwritten, rewards that are more than one trial in the past actually have a longer-term effect on incrementing unchosen action values—regardless whether they were received at the same arm or a different one, thereby driving a tendency to switch. This is reflected in the negative coefficients on trial lags 2–10 seen for the real data and synthesis program’s generated data but not the baseline program’s. **(b)** Probability of returning to a “run” (a sequence of repeated choices) after a single switch as a function of how long that run was. The discovered update mechanism reflected in the synthesis program predicts that a single switch should “reset” the perseveration trace following long runs in particular. Accordingly, we see that the probability of returning to the run rises with the length of that run for runs of lengths 5 or less, then begins to decrease. The synthesis program’s generated data shows a similar pattern, while the baseline’s continues to rise mostly monotonically.

In this experiment, human subjects choose repeatedly among four options by pressing one of four keys on their computer keyboard, and receive rewards indicated by a number of points between 1 and 100 (Figure 2A). A question in the literature regarding this behavior concerns the role of memory: recent analyses of this dataset and others suggest that humans may rely on rapid memorization of rewards received for chosen actions in working memory [10, 44] rather than the classically described incremental trial-and-error reward learning mechanism [39]. We noted upon first reading the discovered programs that the learning update on the chosen action value appeared to be an incremental error-driven rule. However, automatic ablations revealed that this could be simplified to a working memory rule where the previous value is overwritten, being replaced by the value of the reward received (Figure 5A.1). In contrast, we did see incremental updating on the values of the *unchosen* actions, which decayed gradually toward a recency-weighted average reward (Figure 5A.2). This means that recent rewards for one action positively update all other action values. Together, these update patterns make a surprising prediction about how past rewards affect the tendency to repeat a choice: the immediately previous reward should cause a tendency to repeat choice, but all other past rewards should promote switching, because they increment the alternative choices and are overwritten for the current choice. In contrast, a key signature of standard error-driven learning rules like our baseline model is the tendency to repeat choices that have led to higher rewards in the recent past [42]. We designed an additional lagged regression model to check for this pattern, and found that it is indeed present in the dataset (Figure 7A).

The discovered programs also exhibited novel patterns of forgetting on their perseveration variables. Models in the literature which incorporate perseveration [45] typically incrementally update choice statistics and use this to implement a tendency to repeat recently selected actions. In two of the evolved models (one low- and one medium-floor program), the perseveration trace had a mechanism that instead could reset to zero if that action went unchosen a single time (Figure 5A.3-4). This mechanism makes another surprising prediction validated in data: after a long run of choices of the same action, even a single choice of a different action entirely erases the perseverative tendency to repeat that choice. The model predicts that the longer the run (after an initial saturation point), the lower the chance of returning to it; this is in contrast to standard models, according to which longer runs predict strictly more likely returns. We verified this pattern in the dataset (Figure 7B).

#### 4.1.1 Rat Bandit

In this experiment, rats decide between two nose ports and receive binary rewards whose probabilities followed independent random walks for each port (Figure 2B). The baseline model developed for this task included three learning systems operating fully independently, each governing the update of a single decision variable, and the decision on each trial was based on the sum of these variables [8]. While the discovered programs did consistently contain multiple learning systems, these systems did not operate independently. Instead, they typically interacted, either serving as update targets for one another or nonlinearly modulating the impact of one another on choice (Figure 5C.1-3). The discovered programs also differed from the baseline model in that each learning system updated not a single decision variable representing a preference between the two actions, but instead a pair of variables each representing the value of one of the actions. While this was missing from the baseline model, it is a feature that is common in other models for datasets of these kinds [2, 39, 41]. They also differed in a number of details about the balance of learning and forgetting as well as in the relative influence of rewards and reward omissions on learning within each system.

#### 4.1.2 Fly Bandit

In this experiment, fruit flies decided repeatedly between two odors, indicating their choice by entering an arm in a Y-maze that was filled with this odor. They received binary rewards whose probabilities changed independently in blocks (Figure 2C). The evolved programs departed from the baseline models in that they exhibited a typical error-driven learning rule modulated by an atypical, nonstationary learning rate (Figure 5D). The evolved programs with highest quality-of-fit made use of “eligibility traces”, which dynamically modulated learning rate depending on how frequently an option had recently been chosen. This motif is novel with respect to models of learning behavior in flies (Figure 5D.2). Nearly all of our discovered programs maintained a form of reward history, which was also used to modulate learning and/or update rates (Figure 5D.3).

#### 4.1.3 Monkey Bandit

In this experiment, monkeys chose among three images on a screen and received binary rewards with a fixed probability for each image (Figure 2B). Periodically, a novel image with an unknown reward probabilities was exchanged for one of the existing images. This design allows studying how novelty preferences interact with reward learning to guide exploration. The discovered programs outperformed the handcrafted baseline model by restructuring how novelty preference interacts with reward-guided learning (8/9 programs). Previous models largely assumed a single action value tracking average reward which was initialized, for new options, with a fixed novelty bonus [35, 46]. This reflects a substantive theoretical idea about the neural mechanisms of exploration: that the value of exploring a novel option is accounted in common currency with other (e.g., primary) rewards, and processed equivalently as action value by the same brain systems such as dopamine [47]. Empirically, though, this unified approach consistently underestimates the monkeys’ initial novelty seeking (bottom panel of Figure 6), since in the combined model it is forced to decay at the same rate as other rewards. The discovered programs solve this problem, by replacing the unified values with two separate cognitive variables: “action values”; tracking average reward, and a decaying “novelty trace”; which independently tracks perceptual novelty (Figure 5B.1). Instead of the common-currency assumption, this architecture reinforces the theory that novelty-driven exploration instead relies on dissociable cognitive streams [46, 48], which in turn has testable implications for the neural correlates of these variables [36, 48–50].

Consistent with the theme of discovered nonlinear, nonstationary updates, all discovered programs (9/9) also exhibited a nonstationary, nonlinear operation in which the decision variables are scaled by the variance of the action values, which has the effect of making behavior more exploratory (i.e. more stochastic) when the action values are similar.

#### 4.1.4 Rat Two-step

In the *Rat Two-step* experiment (Figure 2E), the connection between a rat’s action and resulting reward is mediated by a stochastic intermediate state, so as to distinguish between *model-based* reinforcement learning (which forecasts action values indirectly via the environment’s dynamics) and *model-free* reinforcement learning (which does not). Accordingly, discovered programs typically use both model-based and model-free action values, though the model-free values can typically be ablated with little or no effect on quality-of-fit [37, 38]. Similar to the *Rat Bandit* dataset (above), all 9 discovered programs differed from the baseline [37, 38] in representing the action values as two-dimensional vectors (one for each option) rather than a single variable summarizing relative preference (Figure 2E.1). As with *Rat Bandit*, this distinction in the learned representation has behaviorally detectable consequences because it allows the model to implement asymmetries: in this case, to decay the reward history for unchosen actions asymmetrically from chosen ones. Discovered models also often agreed (3/6 of low and medium-floor programs) on a specific form for this decay (with shared forgetting for both options, applied before the learning update for the chosen one), which differed from previous models in the literature [39]. Finally, also as in other datasets, nonstationary modulation was sometimes noted; for instance, two discovered programs incorporate a dynamic inverse temperature parameter that increases the entropy of the first choices in each session, perhaps reflecting a difference in strategy early in the session [38].

## 5 Discussion

The fundamental goal of basic science is not prediction or control, but understanding. Applying AI tools to solve basic science problems therefore requires centering human understanding as a key output. An increasingly popular approach for AI modeling of scientific data is to build “foundation models”; that is, to train large-scale neural network models on diverse large-scale datasets [51–54] in order to simulate the system’s behavior in different settings. This approach centers predictive performance as the primary target of optimization and primary metric of success. Scientific understanding might come from probing these models post-hoc, for example using the tools of mechanistic interpretability, but this hope remains somewhat speculative. Our approach uses large-scale neural networks not as models in themselves but as tools for generating models, expressed in a human-readable form and optimized both to fit data well and to be simple.

Model discovery for cognitive science requires discovering stateful computational models from indirect observations. Recent work has developed interpretable neural network approaches to this problem [9–11, 55–57]. Neural models are appealing because they are easy to optimize, but lack the explainability, generalizability, formulaic precision, and identifiability of symbolic models. Symbolic models are more difficult to optimize as one cannot use gradient descent. Existing symbolic regression (SR) [58, 59] and program induction [60–63] use discrete optimization techniques to discover closed-form symbolic models. These methods require handcrafted libraries of basis functions and operations which simultaneously makes the search problem potentially tractable and limits their expressivity [62, 63]. Symbolic regression approaches also cannot directly be applied to problems like those we study here, where the timeseries being modeled are not directly observed (though see [64, 65]). Other recent work has used LLMs to discover symbolic models, as they can easily generate programs in general purpose programming languages [16–19, 66, 67]. Very recent work, including from our group, has applied LLM program synthesis to discover symbolic models from data [28, 29, 68]. These approaches have not aimed to identify programs that match the quality-of-fit of blackbox models. We demonstrate that DataDIVER is capable of discovering symbolic models of latent dynamics from data using general-purpose tools, and to produce novel predictions that can be verified in the data.

The discovered models are readily interpretable as mechanistic hypotheses. Interpreted in this way they bear both striking similarities and important differences to hypotheses that are currently popular in the field. For example, it is common in the literature to use quite similar models in tasks that are implemented using diverse species (humans, monkeys, rats, fruit flies), available actions (button presses, eye movements, whole body movements) and rewards (points, sugar water, optogenetic stimulation). In contrast, we find that the models discovered for the different datasets are quite different. For example, the *Human Bandit* model does not include incremental reward learning, and may resemble a working memory process [10, 44], and the *Fly* and *Rat Bandit* models contain multiple learning mechanisms operating at different timescales [8]. This suggests that these conceptually-similar reward learning tasks may recruit different cognitive algorithms, and therefore different neural mechanisms [69].

Despite this diversity, there are at least two common themes in how the discovered models depart from the literature. First, they frequently introduce novel cognitive variables, such as the novelty tracking in the monkey dataset and the eligibility traces in the fly dataset. Interpreted as mechanistic hypotheses, these make the prediction that there are distinct neural correlates of these novel variables, and that neural perturbation experiments to specific brain regions might specifically affect the aspects of behavior that they mediate in the model. Second, discovered models frequently introduce nonlinear transformations between cognitive variables that are propagated through time and the computation of choice. These make the prediction that learning processes and choice processes in the brain may be more separate than is typically thought.

An important note of caution is that, as with any data-driven discovery tool, there is no guarantee that models discovered by DataDIVER will correspond with the true cognitive mechanisms used by the brain. Instead, they are best viewed as candidate hypotheses, which must be evaluated by human scientists to determine which, if any, contain plausible new ideas. That they take the form of computer programs facilitates this evaluation process, and makes it easy to mix and match ideas between models. The ultimate test of a model is whether it can successfully make predictions for new experiments involving radically different types of data – for our models this might take the form of very different behavior experiments in the same species and experimental setups, of neural recording experiments seeking correlates of the novel cognitive variables our models identify, or neural perturbation experiments asking whether focal changes to the model result in simulated behavior that resembles that of animals with focal perturbations to their brains.

While we have applied DataDIVER to questions about reward learning, the tool is general. Many scientific fields, for example ecology, epidemiology, and economics, require inferring the structure of indirectly observable latent processes in situations where data is plentiful but theories explaining the data are limited. To date, AI for scientific discovery has largely focused on prediction, forecasting, or mathematical problems that can be formulated as maximizing a single score with a black-box model [52, 53, 70]. They have steered clear of scientific problems where a breakthrough consists instead of a novel explanation. Generative AI’s ability to automatically generate artifacts that humans can readily inspect and understand, be it computer code, natural language, or images and charts, provides a new opportunity to start taking on these new types of discovery problems. Our work represents a step toward a vision in which these tools are used to help scientists make sense of the natural world as captured by data.

## 7 Methods

### 7.1 Dataset structure

Each dataset consists of data from multiple *subjects*, with each subject having completed multiple *sessions* comprised of a sequence of *trials*^1^. Across all datasets, a trial entails a subject making a *choice* and receiving a *reward* based on that choice. Two of the datasets include additional information specific to the experimental design. For the *Monkey Bandit* dataset, in which images representing choices are occasionally exchanged for new images with unknown reward, trial information includes about which (if any) options have been replaced by a novel option. For the *Rat Two-step* dataset, in which the rat’s reward is mediated by an observed outcome which is stochastically sampled based on the rat’s choice, trial information includes this outcome. For each dataset, we follow Castro et al. [28] in assigning half of the subjects (those with even indices) to the training split and using them for program generation, and holding out the remaining subjects for post-hoc program evaluation only.

Individual datasets are described in more detail in Appendix A.

### 7.2 Program structure

Each program accepts as input parameters, information about the current trial, and the previous agent state, and outputs a probabilistic prediction about the next choice as well as an updated agent state. The parameters allow the program to modify its behavior to match individual participants, and do not change over time or across sessions. The names and roles assigned to each parameter are not set ahead of time, and are instead established by DataDIVER. The trial information contained recent choice and recent reward, as well as dataset-specific information for *Monkey Bandit* (novel option, if any) and *Rat Two-step* (recent outcome). The previous agent state was required to be an array that did not change in size across or within sessions; however, the dimensions of the array and the names and roles assigned to agent state variables could be determined by DataDIVER. The agent state had a null value if none was provided, meaning that the program had to define the agent state during the first trial.

All programs were written in JAX [33] so that parameters could be optimized with gradient descent.

### 7.3 Quality-of-fit

We follow the bi-level cross-validation process of Castro et al. [28] in which an outer loop optimizes programs across subjects and an inner loop optimizes per-subject parameters across a subject’s sessions. Parameters were optimized using two-fold cross-validation, with folds comprised of even and odd sessions, respectively. This involved fitting parameters to each fold by maximizing the summed log likelihood of the all choices under the program’s predictions, and validating the parameters on the opposite fold, producing two log likelihood validation scores. To produce a single score for the subject, the two log likelihood scores are summed, divided by the total number of trials across both folds, and exponentiated to produce a “normalized likelihood” score. This can be interpreted as the geometric average probability that the model would have made the choices that the participants made [2]. Our final “quality-of-fit” score for each program is the average of these normalized likelihoods across participants. Unless specifically indicated (e.g. in Figure 1B,C), quality-of-fit scores are always reported on held-out test subjects that were not used for program evolution.

Because in the *Fly Bandit* experiment each fly only participated in one session. Thus, both programs and parameters were optimized across subjects, and could be thought of as capturing fly behavior in aggregate. This is described in Appendix A.3.2.

Maximum likelihood parameters were estimated using gradient-based optimization, with initial parameters sampled uniformly from 𝒰(-2,2). Gradient descent terminated if the convergence criterion was met, the maximum number of steps $M_{gd}$ was reached, or the score became undefined (e.g., due to exploding state variables).

To address the issue of nonconvexity, we conducted multiple fitting attempts per dataset using different initializations. After the minimum number of fitting attempts $m_{\text{fit}}$ occurred, the process terminated once $n_{\text{fit}}$ runs converged to values near the best obtained score s*, defined as scores s satisfying $\frac{\left|s-s*\right|}{s*}<\epsilon_{\text{fit}}$, or once $M_{\text{fit}}$ total attempts had elapsed.

During training, we used a set of optimization hyperparameters tuned for speed. For the final evaluation and reported results, we employed a more stringent set of hyperparameters and a different gradient-based optimizer (L-BFGS instead of AdaBelief) to ensure accuracy. The convergence criteria also differed: during training, we checked the score every $k_{gd}$ steps and declared convergence if $\frac{\left|s_{t}-s_{t-k}\right|}{s_{t-k}}<\epsilon_{gd}$, where $s_{t}$ is the current score and $s_{t-k}$ is the score from k steps prior. During evaluation, we used the gradient infinity norm, declaring convergence if $\frac{{max}_{i}\;\left|g_{i}\right|}{s_{t-1}}<\epsilon_{gd}$, where $g_{i}$ is the gradient of the i-th parameter.

Additionally, we used distinct random seeds for evaluation to ensure results did not reflect overfitting to a specific initialization order. Because of the time-consuming nature of evaluating automatic ablations, these were computed using the training hyperparameters and seeds. See Table 1 for the complete list of optimization parameters.

Parameter optimization was done using custom code implemented in Python and JAX.

### 7.4 Halstead complexity metrics

We use Halstead complexity metrics to capture program complexity [30]. These are syntax-only analysis measures developed to quantify software complexity, maintainability, and size based on the number of operators and operands in source code. Our pipeline involves three of these metrics–volume V, difficulty D, and effort E– which can be defined in terms of the number of distinct operators $\eta_{1}$, distinct operands $\eta_{2}$, total operators $N_{1}$, and total operands $N_{2}$:

$$V=N\times {log}_{2}\left(\eta_{1}+\eta_{2}\right)$$

$$D=\frac{\eta_{1}}{2}\times \frac{N_{2}}{\eta_{2}}$$

E=E×V

Volume represents the program’s total information content, difficulty represents the code density/obfuscation, and effort represents the mental work of implementing the code.

### 7.5 Optimizing programs with AlphaEvolve

Program optimization was performed using AlphaEvolve [19], which implements an evolutionary algorithm that optimizing programs using large language models as the mutation operator. The language model used for all AlphaEvolve runs was Gemini 2.5 Flash [77]. A simple dead code elimination algorithm was applied to each generated program to remove unused or overwritten variable assignments. All AlphaEvolve runs were halted after 100, 000 steps.

### 7.6 Two-stage program optimization

DataDIVER consists of two stages: a “Maximize Quality-of-Fit” first stage, and a “Simplify” second stage. Each stage involved a separate AlphaEvolve run with different objectives. Three independent runs of the entire two stage process were conducted, starting from different seeds.

Performing the multiobjective optimization in two stages permits discovery of models in the high quality-of-fit, high complexity region of the model space before simplicity constraints are introduced. This is similar to the strategy of KL-annealing for training variational autoencoders, wherein models are trained such that fit-to-data is prioritized early in training and complex latent activity is penalized later to prevent posterior collapse [78].

#### 7.6.1 Stage 1: “Maximize Quality-of-Fit”

In the first stage of DataDIVER, “Maximize Quality-of-Fit”, the target of optimization is the average normalized likelihood (described in Section 7.3). The LLM is prompted with text that contains context on the computational modeling task, examples of prior programs, a “parent program” that it will edit, and instructions for how to format edits (see the complete prompt in section C.1. Instructions suggesting different strategies for editing the program (e.g. “combine the strengths of the programs above”, “implement an idea not present not present but commonly used in the literature.”) are sampled stochastically (see Table C8 for full specifications).

The product of this stage is a vast set of programs generated by the LLM. The program with the highest quality-of-fit is referred to as the “fit only” program because it was optimized with only quality-of-fit in mind. The set of programs on the “Pareto frontier” of quality-of-fit versus Halstead effort are also extracted and used to initialize stage 2. The Pareto frontier refers to programs that are optimal for some trade-off of the two metrics; more specifically, any program for which no other program exists that is better at one metric without being worse at the other.

#### 7.6.2 Stage 2: “Simplify”

In a second “Simplify” stage, the Pareto frontier programs from the first stage are further evolved in order to better trade off complexity and quality-of-fit. Here, the LLM is prompted to simplify the program rather while maintaining similar quantitative performance (see the complete prompt in Section C.2, with stochastically sampled prompts suggesting different strategies for simplifying the code (see Table C9). AlphaEvolve was run in multiobjective mode with both Halstead difficulty and volume as optimization metrics at this stage. For each “Simplify” stage we specified a quality-of-fit floor, and any generated programs with quality-of-fit scores below this floor were discarded.

Floors were selected to lie at fixed fractions α of the gap between the fixed handcrafted baseline model’s quality-of-fit score $s_{B}$ and that of the best program discovered across all three runs s*, such that each floor can be written as $s_{B}+\alpha \left(s*-s_{B}\right)$. For each of the three DataDIVER runs, we run this “Simplify” AlphaEvolve run three independent times with different values for α: a “low floor” α of 50%, a “medium floor” α of 75%, and a “high floor” α of 90%. From each “Simplify” run, we extract the program with the best Halstead effort, leaving us with nine programs in most cases (3 DataDIVER runs × 3 “Simplify” runs each). Occasionally, due to variance in performance of the best programs in the best first-stage runs, some stage 1 runs did not exceed the α=90% performance threshold; these runs thus fail to produce any valid programs, resulting in some datasets having fewer than 9 total program simplification experiments.

For each of the (at most) 9 program simplification runs, we select the generated program with the minimum Halstead effort (the product of the difficulty and volume metrics optimized above). We then apply a final readability refactoring step, which involves prompting Gemini 2.5 Pro to rewrite the program to be more readable while ensuring the program’s functionality is unaffected (the rewriting prompt is specified in Section C.3). A hash function is used to ensure that the program’s functionality is unaffected. For each input program, a hash was computed by computing the output logits and state values across 100 rollouts of length 10 with randomly sampled parameters and inputs. Discrete inputs (e.g. choices) are sampled from a uniform multinomial distribution, continuous inputs (reward in *Human Bandit*) from a uniform distribution on the [0, 1] interval, and parameters from a normal distribution with mean 0 and standard deviation 1. The flattened and concatenated vector of outputted logits and state values constitutes an approximately unique signature of program behavior. The refactor is deemed successful if a program is generated whose hash matches the hash of the un-rewritten program, given the same random inputs. Rewriting is attempted a maximum of ten times, and with a deadline of 600 seconds total. Refactor succeeded for 41 out of 43 programs; the other two (*Human Bandit*, high floor, run 2; *Rat Two-step*, low floor, run 2) were kept in their unrefactored form.

### 7.7 Program evaluation

For each dataset, up to twelve total programs are evaluated: the three “fit-only” programs, which are optimized for quality-of-fit only and are outputted by the “Maximize Quality-of-Fit” stage (3 programs per dataset); and the simplified programs that emerge from the “Simplify” stages (up to 9 programs per dataset). For each of these programs, we calculate the quality-of-fit for the held out evaluation subjects, again using cross-validation procedure across each subject’s even/odd sessions as described in Section 7.3. Note that this entails two levels of validation: programs are validated on never-before-seen subjects, while parameters are validated across sessions for each subject.

### 7.8 Automatic ablations

In order to test which of a program’s many operations contributed to the program’s performance, we implemented an automated ablation procedure. This consists of generating all programs that modify the input program in one of the following ways:

- deleting a line of code; (e.g. removing a line x=x+y)
- setting the right-hand side of one variable assignment to zero or a size-matched array of zeros (e.g. with x=jnp.zeros_like(x+y));
- setting the right-hand side of one variable assignment to one or a size-matched array of ones (e.g. with x=jnp.ones_like(x+y));
- replacing a binary operator (e.g., + or *) with either its left or its right argument (e.g. x=x+y→x=x).

We then recompute the quality-of-fit across evaluation subjects for each of these programs, and measure the drop in score. This is useful for identifying whether any of the computations that the “Simplify” stage failed to prune are in fact unnecessary, and understand how much each operation is contributing to performance in terms of magnitude of explained likelihood and significance across subjects.

### 7.9 Recurrent neural network (RNN) baselines

We compare the programs generated using DataDIVER to recurrent neural network (RNN) baselines. The RNNs each consist of one gated recurrent unit (GRU) layer followed by a linear readout layer [79].

For the *Rat Bandit*, *Monkey Bandit*, and *Rat Two-step* datasets, in which each subject participated in a large number of sessions, separate RNN models were trained for each subject. This involved performing two-fold cross-validation by training one network on that subject’s even-indexed sessions and evaluating on that subject’s odd sessions (and vice versa). We performed a sweep over the learning rate (0*.*00001,0*.*0001,0*.*001), the number of hidden units (1, 2, 4, 8, 16, 32, 64, 128), and three random initialization seeds in order to find the optimal hyperparameters for each dataset. In all cases, we train for 100,000 learning steps, and log parameters and quality-of-fit every 100 steps. The quality-of-fit, cross-validated over sessions, is computed for the even-indexed training subjects (the same set of subjects used for generating programs in DataDIVER) and used for hyperparameter selection: we select the learning rate, the number of hidden units, and an early stopping point, that maximize quality-of-fit across subjects and random seeds. The reported RNN performance is on the odd-indexed evaluation subjects (the same subjects on which we DataDIVER programs), using the hyperparameters and early stopping point selected from the even-indexed subjects. We report the results of the best-performing random seed.

For the *Fly Bandit* and *Human Bandit* datasets, in which each subject participated in few sessions (*Human Bandit*) or one session (*Fly Bandit*), there were not enough sessions to train and validate a separate RNN for each subjects [28]. Sessions were therefore combined across subjects, and RNNs were trained on this aggregate data. Even-indexed subjects’ sessions were combined into one training split, and odd-indexed subjects’ sessions were combined into an evaluation split. Note that this preserved the train and evaluation subject splits used by DataDIVER. For each split, these combined sessions were further subdivided in half for two-fold cross-validation. The cross-validated normalized likelihood computed for the training subjects was used for hyperparameter selection (learning rate, number of hidden units, and early stopping point). Reported performance scores are computed on the odd-indexed evaluation subjects. We note that rather than report the average performance across all combined sessions, we re-normalized across subjects for apples-to-apples comparison with DataDIVER programs. As with DataDIVER, we use the results for the best-performing random seed. The best-performing hyperparameters for each dataset can be found in Table 2.

### 7.10 Artificial Data Generation

In order to understand the behavior of the different models, artificial datasets were generated from each of DataDIVER-discovered programs, the synthesis programs, the handcrafted baselines, and the RNNs. This entailed running artificial experiments with the models and their optimized parameters using an *experimental environment* configured to match the outputs or possible outcomes of the experiment used to collect the real data.

To generate a simulated session of data, the model was initialized with the optimized parameters, a null agent state, the first action and reward observed in the real data, and any other relevant trial information, if applicable (outcome for *Rat Two-step*, novel option for *Monkey Bandit*). Optimized parameters used for artificial data generation were those obtained using cross-validation in order to score the models. This means that for each session of artificial data in one fold, the parameters used to generate that data were those obtained by optimizing parameters over the data in the opposite fold. On each timestep, the model would output an updated state and a probability distribution from which the next choice would be sampled. Whenever the choice matched the choice made by the animal, the same reward (and outcome, for *Rat Two-step*)) was observed; otherwise, rewards (and other outcomes) were sampled from the experimental environment given the experimental configuration. This was applied iteratively for as many trials as the animal completed in the corresponding session to collect artificial data. In all, five artificial datasets (with different random seeds governing the choice sampling) were generated per model.

The experimental environment was instantiated such that it would return rewards (and other outcomes, for *Rat Two-step*) that were observed in the real data whenever the model’s choices matched those of the real subject at the same trial. When choices differed, the environment would return rewards (and outcomes) sampled from the distribution specified by the experimental configuration (i.e. observations the subject might have seen had it made that choice). For *Monkey Bandit*, the experimental environment also contained when existing options were swapped out for novel options so that these were matched to the real data.

The rewards and other inputs are sampled in the following way, depending on dataset:

- For the *Human Bandit* dataset, rewards are determined by a payout matrix that indicates the exact reward that each participant would receive for each available choice in each trial of the experiment.
- The *Rat Bandit*, *Fly Bandit*, and *Monkey Bandit* datasets include the *probability of (binary) reward* for each (chosen or unchosen) choice in each trial of the experiment. For each trial of the synthetic experiment, if the choice does not match the choice made by the real subject, a binary reward is sampled according to the probability specified for the agent function’s choice in the equivalent natural trial.
- The *Rat Two-step* dataset also includes the probabilities of reward, but these depend only indirectly on the agent’s choice: instead, choices stochastically determine the binary *outcome*, and outcomes determine reward probabilities. For each trial, a choice could be “congruent” (choice and outcome located on the same side) or incongruent (choice and outcome located on opposite sides). When generating the artificial dataset, we first determine whether each trial’s choice and outcome were congruent or incongruent in the real data. This congruency relationship was preserved in the experimental environment regardless of the choice. Rewards are then sampled conditional on the outcome, using the reward probabilities specified for that trial in the natural dataset. When the sampled outcome matches the outcome observed in the real data, the rewards will also be matched.

### 7.11 Trial-Lagged Regression Plots

To determine the extent to which each model captured relevant features of the corresponding real data, we used trial-history regression analyses common in the literature [8, 37, 41, 42].

For the *Rat Bandit* and *Fly Bandit* datasets, the following logistic regression model was fit to the data (as in Castro et al. [28]):

$$log\left(\frac{p_{1,t}}{p_{0,t}}\right)=\sum_{\tau =1}^{T}\left[\alpha_{\tau }\left(2c_{t-\tau }-1\right)+\beta_{\tau }\left(2c_{t-\tau }-1\right)\left(2r_{t-\tau }-1\right)\right]$$

with the choices $c_{t}\in \{0,1\}$, rewards $r_{t}\in \{0,1\}$, and where we characterize $log\left(\frac{p_{1,t}}{p_{0,t}}\right)$, the log-odds of choosing $c_{t}=1$, in terms of the past choices and the past products of choice and reward (after normalizing both choice and reward be in {−1, 1}). The $\alpha_{\tau }$ coefficient characterizes the extent to which the subject tends to repeat the choice made τ trials ago; the β coefficients characterize the extent to which the subject tends to repeat rewarding choices and avoid non-rewarding choices from τ trials ago.

Because the *Human Bandit* and *Monkey Bandit* datasets are four- and three-armed bandit tasks respectively, we use a conditional logit regression model [80], where a linear model outputs a utility $U_{i}$ for each choice i, and choices are sampled according to the softmax of the utilities. For the *Monkey Bandit* dataset, the utility for choice i∈{0,1,2} is

$$U_{i,t}=\gamma_{0}n_{t,i}+\sum_{\tau =1}^{T}\left[\alpha_{\tau }1_{c_{t-\tau }=i}+\beta_{\tau }\left(2*1_{c_{t-\tau }=i}-1\right)\left(r_{t-\tau }-\overset{‾}{r}\right)+\gamma_{i}n_{t-\tau ,i}\right],$$

where $1_{c_{t-\tau }=i}$ is 1 if the τ-back choice $c_{t-\tau }$ is equal to choice i and 0 otherwise, $\overset{‾}{r}$ is the mean reward over the entire dataset and $r_{t-\tau }-\overset{‾}{r}$ is the mean-centered reward received τ trials back, and $n_{t,i}$ is 1 if choice i is the novel option at time t and 0 otherwise. Here $\alpha_{\tau }$ again represents the tendency to repeat a choice made τ trials back, $\beta_{\tau }$ represents the tendency to repeat choices that yielded above-average reward τ trials ago and avoid those that did not, and $\gamma_{i}$ represents the tendency to select novel options added to the choice set τ trials ago.

The *Human Bandit* regression model is identical except it excludes the novel option terms and their coefficients.

For the *Rat Two-step* experiment, we repeat the trial-history lagged regression model introduced by Miller et al. [37]. In that experiment, the transitions between rats’ choices and the (observable) outcomes were stochastic, with one transition being *common* (happening 80% of the time) and one being *uncommon* (happening 20% of the time) for each choice (note: this is different from congruency, see Appendix A.5). The outcome that was common for one choice was the uncommon outcome for the other. Given choice at time $tc_{t}\in \{0,1\}$, reward $r_{t}\in \{0,1\}$, and $b_{t}\in \{0,1\}$ indicating whether the transition at time t was a common transition, we can define regressors capturing relevant information for this task: $CR(t)=\left(2c_{t}-1\right)b_{t}r_{t}$ (Common Reward, common outcome occurred and was followed by reward), $CO(t)=\left(2c_{t}-1\right)b_{t}\left(1-r_{t}\right)$ (Common Omission), $UR(t)=\left(2c_{t}-1\right)\left(1-b_{t}\right)r_{t}$ (Uncommon Reward), and $UO(t)=\left(2c_{t}-1\right)\left(1-b_{t}\right)\left(1-r_{t}\right)$ (Uncommon Omissions). The logistic regression model is defined as follows:

$$log\left(\frac{p_{1,t}}{p_{0,t}}\right)=\sum_{\tau =1}^{T}\;\left[\beta_{CR}CR(t-\tau )+\beta_{CO}CO(t-\tau )+\right.\;\;\left.\beta_{UR}UR(t-\tau )+\beta_{UO}UO(t-\tau )\right]$$

By breaking down the dependency of the current trial on past trials’ choices and rewards into terms depending on the commonness of the choice → outcome transition, we can distinguish between agents which update the values of choices in a model-free manner (and thus are more likely to repeat an action that resulted in reward, regardless if that reward resulted from the common transition) and those which engage in some planning (which are *less* likely to repeat an action that resulted in reward if that action was the result of an uncommon transition, since the outcome which is uncommon for one choice is common for the other).

The error bars for all trial lagged regression plots reflect the 95% prediction intervals. This indicates the range that is expected, with 95% probability, to contain the value of a single future observation.

### 7.12 Trial-lagged regression: repeating rewarding choices

To assess whether past reward promotes a tendency to stay with the same choice ($c_{t}=c_{t+1}$) or switch to a different choice ($c_{t}\neq c_{t+1}$), we performed a lagged regression model that was broken down into whether the τ-back reward had been received for a choice of the current arm (“same choice”) vs an alternative one (“different choice”). For this, the linear model could be written as:

$$log\left(\frac{p\left(c_{t}=c_{t+1}\right)}{p\left(c_{t}\neq c_{t+1}\right)}\right)=\sum_{\tau =1}^{T}\left[\alpha_{\tau }1_{c_{t-\tau }=c_{t}}r_{t-\tau }+\beta_{\tau }1_{c_{t-\tau }\neq c_{t}}r_{t-\tau }+\gamma_{\tau }1_{c_{t-\tau }=c_{t}}\right]$$

We choose T=10. Figure 7a plots the values of $\alpha_{\tau }$ for τ∈{1,⋯,10}; the values of $\beta_{\tau }$ and $\gamma_{\tau }$ can be found in Figure A3 in the Supplement.

### 7.13 Run return probabilities

In order to test whether the discovered forgetting rule was present in the *Human Bandit* dataset, the probability of returning to a “run” (a sequence of repeated choices) after a single switch was observed. A run of length n is defined as a sequence in which the same choice is consecutively made for exactly n steps. For each session, all runs were identified and counted. For each run, we computed the number of times that the second choice made after ending a run was the same as the choice made during the run (the choice immediately following each run is, by definition, different). The fraction of runs in which the subject returned to the choice made during the run was computed for each run length and shown in Figure 7B. The error bars show 95% confidence intervals computed across all sessions and subjects combined.

### 7.14 Synthesis Programs

In order to unify the different discovered programs into a single programs, synthesis programs were manually constructed. We note that this step was not required to produce interpretable programs; rather, this allows us to verify that different motifs discovered across different programs do not interfere when combined and therefore can each be safely interpreted. Broadly, the procedure for constructing these programs was to first start with a discovered program, then remove elements that did not contribute to the overall likelihood (as assessed with automated ablations; see Section 7.8). Motifs from other programs were substituted in if they contributed to performance in other programs, or if they contributed to uniquely good performance for some diagnostic (as in *Human Bandit*, where the selected forgetting rule led to high performance on the Run Return statistics). Crucially, no extra motifs based on background knowledge of the literature were added.

## Supplementary Material

Supplement 1

Supplement 2

Supplement 3

## Figures and Tables

**Table 1:** Optimization hyperparameters used to compute quality-of-fit.

Parameter	Training	Evaluation

Optimizer	AdaBelief	L-BFGS
Learning rate	0.05	N/A
Convergence criterion	$\frac{\left|s_{t}-s_{t-k}\right|}{s_{t-k}}<\epsilon_{gd}$	$\frac{{max}_{i}\;\left|g_{i}\right|}{s_{t-1}}<\epsilon_{gd}$
*k*_gd_	100	N/A
*ϵ*_gd_	0.01	0.0001
*M*_gd_	10, 000	10, 000
*n*_fit_	3	5
*ϵ*_fit_	0.01	0.0001
*m*_fit_	0	10
*M*_fit_	10	1, 000

**Table 2:** Hyperparameters which maximize RNN performance on even-indexed subjects or even split, per dataset.

Dataset	# hidden units	Learning rate	Early stopping point (# steps)

*Human Bandit*	16	0.0001	99800
*Rat Bandit*	128	0.001	200
*Fly Bandit*	128	0.001	200
*Monkey Bandit*	2	0.001	1900
*Rat Two-step*	128	0.001	100

## Footnotes

1 For the *Fly Bandit* dataset, each subject completed only one session, necessitating a different strategy for dividing test and train data, which is described in more detail in Appendix A.3.2
