# Supplementary material for: AI-Discovered Cognitive Models Reveal Novel Insights into Human and Animal Learning
Source: bioRxiv. 2026 May 21:2026.05.18.725921. Preprint. [Version 1] doi: 10.64898/2026.05.18.725921 (PMC13228651; doi:10.64898/2026.05.18.725921)
Supplement: Supplement 2 [file media-2.zip › ablation_performance_fly_bandit_run1_high_floor_20260420.pdf]

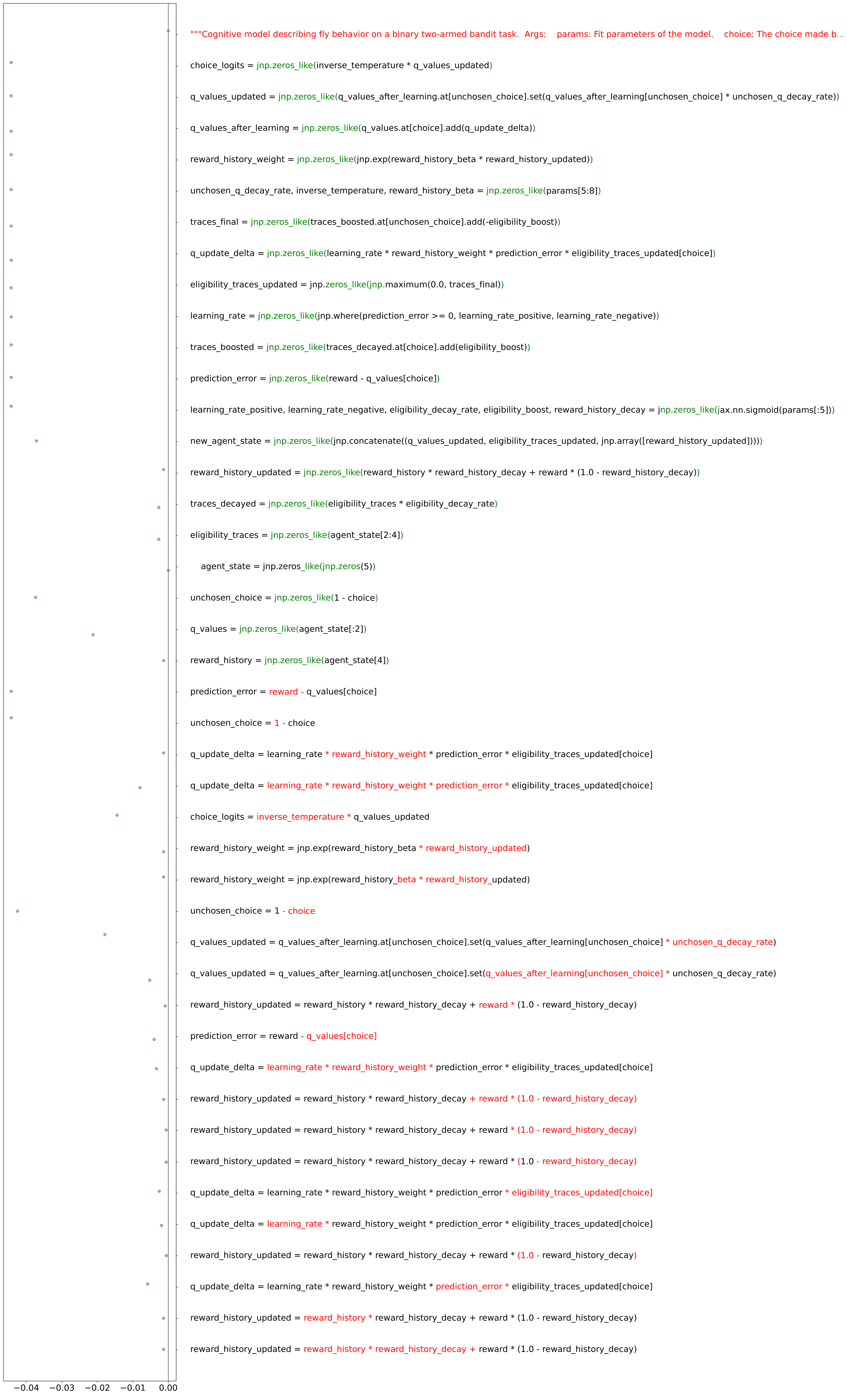
"""Cognitive model describing fly behavior on a binary two-armed bandit task. Args: params: Fit parameters of the model. choice: The choice made b...
choice_logits = jnp.zeros_like(inverse_temperature * q_values_updated)
q_values_updated = jnp.zeros_like(q_values_after_learning.at[unchosen_choice].set(q_values_after_learning[unchosen_choice] * unchosen_q_decay_rate))
q_values_after_learning = jnp.zeros_like(q_values.at[choice].add(q_update_delta))
reward_history_weight = jnp.zeros_like(jnp.exp(reward_history_beta * reward_history_updated))
unchosen_q_decay_rate, inverse_temperature, reward_history_beta = jnp.zeros_like(params[5:8])
traces_final = jnp.zeros_like(traces_boosted.at[unchosen_choice].add(-eligibility_boost))
q_update_delta = jnp.zeros_like(learning_rate * reward_history_weight * prediction_error * eligibility_traces_updated[choice])
eligibility_traces_updated = jnp.zeros_like(jnp.maximum(0.0, traces_final))
learning_rate = jnp.zeros_like(jnp.where(prediction_error >= 0, learning_rate_positive, learning_rate_negative))
traces_boosted = jnp.zeros_like(traces_decayed.at[choice].add(eligibility_boost))
prediction_error = jnp.zeros_like(reward - q_values[choice])
learning_rate_positive, learning_rate_negative, eligibility_decay_rate, eligibility_boost, reward_history_decay = jnp.zeros_like(jax.nn.sigmoid(params[:5]))
new_agent_state = jnp.zeros_like(jnp.concatenate((q_values_updated, eligibility_traces_updated, jnp.array([reward_history_updated]))))
reward_history_updated = jnp.zeros_like(reward_history * reward_history_decay + reward * (1.0 - reward_history_decay))
traces_decayed = jnp.zeros_like(eligibility_traces * eligibility_decay_rate)
eligibility_traces = jnp.zeros_like(agent_state[2:4])
agent_state = jnp.zeros_like(jnp.zeros(5))
unchosen_choice = jnp.zeros_like(1 - choice)
q_values = jnp.zeros_like(agent_state[:2])
reward_history = jnp.zeros_like(agent_state[4])
prediction_error = reward - q_values[choice]
unchosen_choice = 1 - choice
q_update_delta = learning_rate * reward_history_weight * prediction_error * eligibility_traces_updated[choice]
q_update_delta = learning_rate * reward_history_weight * prediction_error * eligibility_traces_updated[choice]
choice_logits = inverse_temperature * q_values_updated
reward_history_weight = jnp.exp(reward_history_beta * reward_history_updated)
reward_history_weight = jnp.exp(reward_history_beta * reward_history_updated)
unchosen_choice = 1 - choice
q_values_updated = q_values_after_learning.at[unchosen_choice].set(q_values_after_learning[unchosen_choice] * unchosen_q_decay_rate)
q_values_updated = q_values_after_learning.at[unchosen_choice].set(q_values_after_learning[unchosen_choice] * unchosen_q_decay_rate)
reward_history_updated = reward_history * reward_history_decay + reward * (1.0 - reward_history_decay)
prediction_error = reward - q_values[choice]
q_update_delta = learning_rate * reward_history_weight * prediction_error * eligibility_traces_updated[choice]
reward_history_updated = reward_history * reward_history_decay + reward * (1.0 - reward_history_decay)
reward_history_updated = reward_history * reward_history_decay + reward * (1.0 - reward_history_decay)
reward_history_updated = reward_history * reward_history_decay + reward * (1.0 - reward_history_decay)
q_update_delta = learning_rate * reward_history_weight * prediction_error * eligibility_traces_updated[choice]
q_update_delta = learning_rate * reward_history_weight * prediction_error * eligibility_traces_updated[choice]
reward_history_updated = reward_history * reward_history_decay + reward * (1.0 - reward_history_decay)
q_update_delta = learning_rate * reward_history_weight * prediction_error * eligibility_traces_updated[choice]
reward_history_updated = reward_history * reward_history_decay + reward * (1.0 - reward_history_decay)
reward_history_updated = reward_history * reward_history_decay + reward * (1.0 - reward_history_decay)
−0.04
−0.03
−0.02
−0.01
0.00
