# Supplementary material for: AI-Discovered Cognitive Models Reveal Novel Insights into Human and Animal Learning
Source: bioRxiv. 2026 May 21:2026.05.18.725921. Preprint. [Version 1] doi: 10.64898/2026.05.18.725921 (PMC13228651; doi:10.64898/2026.05.18.725921)
Supplement: Supplement 2 [file media-2.zip › ablation_performance_fly_bandit_run2_low_floor_20260420.pdf]

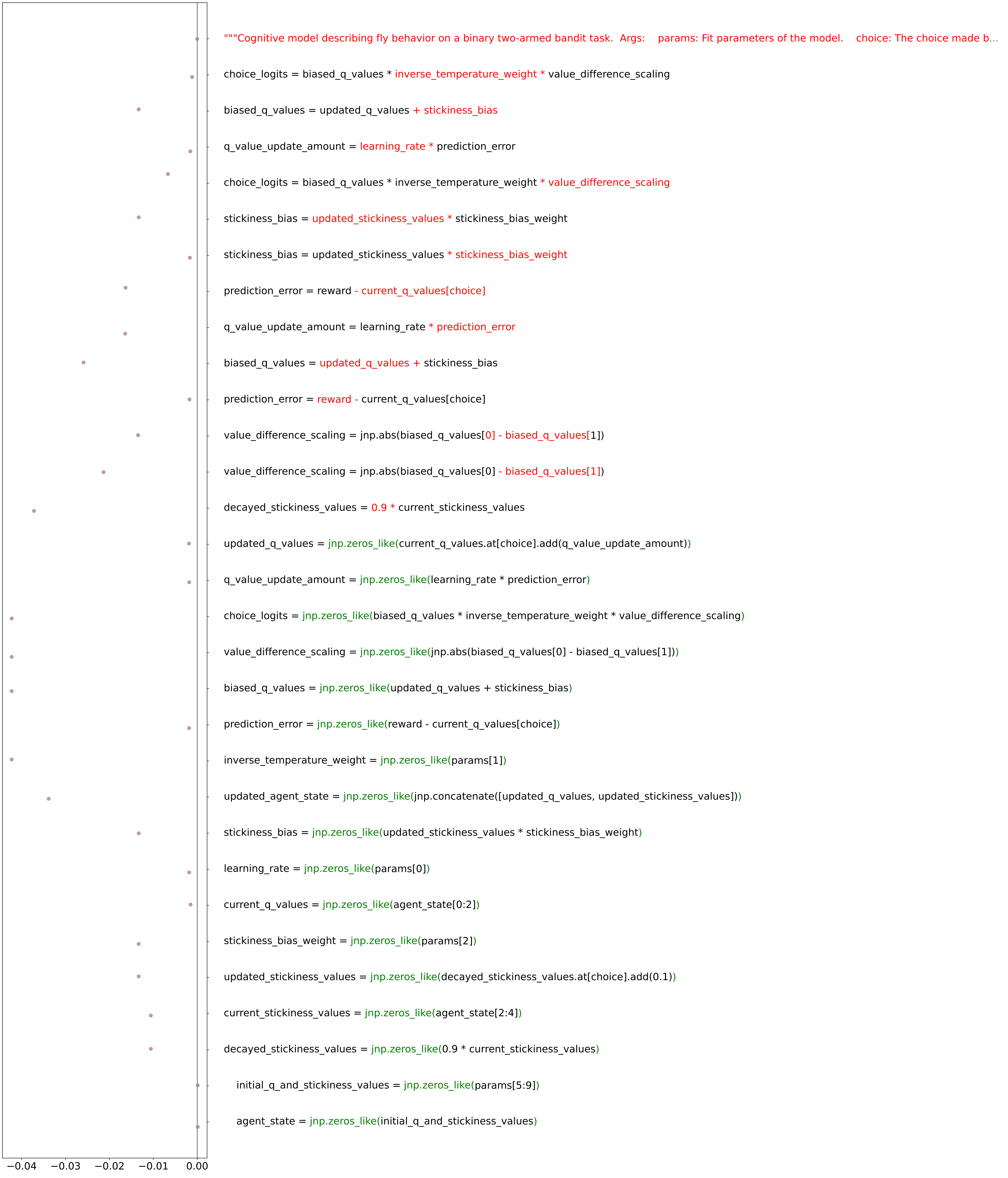
"""Cognitive model describing fly behavior on a binary two-armed bandit task. Args: params: Fit parameters of the model. choice: The choice made b...
choice_logits = biased_q_values * inverse_temperature_weight * value_difference_scaling
biased_q_values = updated_q_values + stickiness_bias
q_value_update_amount = learning_rate * prediction_error
choice_logits = biased_q_values * inverse_temperature_weight * value_difference_scaling
stickiness_bias = updated_stickiness_values * stickiness_bias_weight
stickiness_bias = updated_stickiness_values * stickiness_bias_weight
prediction_error = reward - current_q_values[choice]
q_value_update_amount = learning_rate * prediction_error
biased_q_values = updated_q_values + stickiness_bias
prediction_error = reward - current_q_values[choice]
value_difference_scaling = jnp.abs(biased_q_values[0] - biased_q_values[1])
value_difference_scaling = jnp.abs(biased_q_values[0] - biased_q_values[1])
decayed_stickiness_values = 0.9 * current_stickiness_values
updated_q_values = jnp.zeros_like(current_q_values.at[choice].add(q_value_update_amount))
q_value_update_amount = jnp.zeros_like(learning_rate * prediction_error)
choice_logits = jnp.zeros_like(biased_q_values * inverse_temperature_weight * value_difference_scaling)
value_difference_scaling = jnp.zeros_like(jnp.abs(biased_q_values[0] - biased_q_values[1]))
biased_q_values = jnp.zeros_like(updated_q_values + stickiness_bias)
prediction_error = jnp.zeros_like(reward - current_q_values[choice])
inverse_temperature_weight = jnp.zeros_like(params[1])
updated_agent_state = jnp.zeros_like(jnp.concatenate([updated_q_values, updated_stickiness_values]))
stickiness_bias = jnp.zeros_like(updated_stickiness_values * stickiness_bias_weight)
learning_rate = jnp.zeros_like(params[0])
current_q_values = jnp.zeros_like(agent_state[0:2])
stickiness_bias_weight = jnp.zeros_like(params[2])
updated_stickiness_values = jnp.zeros_like(decayed_stickiness_values.at[choice].add(0.1))
current_stickiness_values = jnp.zeros_like(agent_state[2:4])
decayed_stickiness_values = jnp.zeros_like(0.9 * current_stickiness_values)
initial_q_and_stickiness_values = jnp.zeros_like(params[5:9])
agent_state = jnp.zeros_like(initial_q_and_stickiness_values)
−0.04
−0.03
−0.02
−0.01
0.00
