# Supplementary material for: AI-Discovered Cognitive Models Reveal Novel Insights into Human and Animal Learning
Source: bioRxiv. 2026 May 21:2026.05.18.725921. Preprint. [Version 1] doi: 10.64898/2026.05.18.725921 (PMC13228651; doi:10.64898/2026.05.18.725921)
Supplement: Supplement 2 [file media-2.zip › ablation_performance_rat_bandit_run1_low_floor_20260420.pdf]

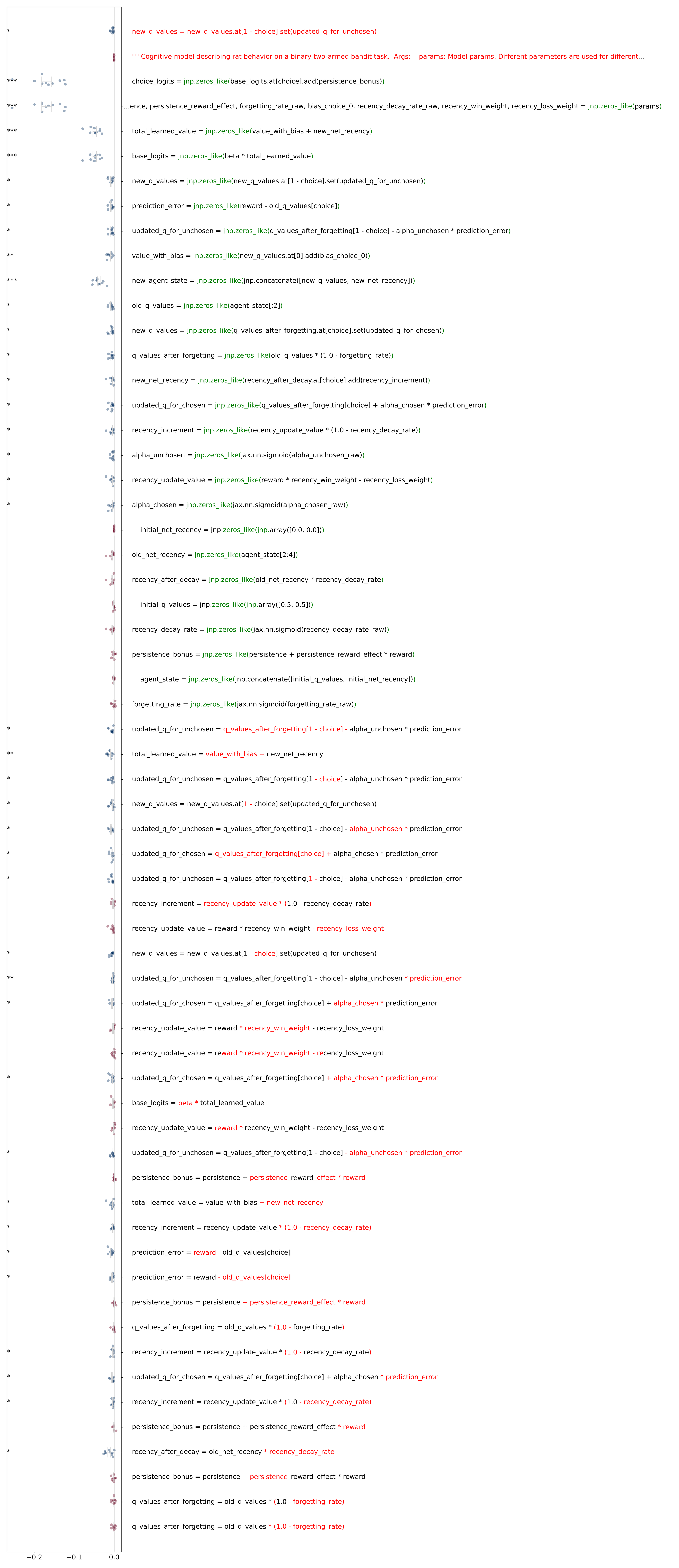
* new_q_values = new_q_values.at[1 - choice].set(updated_q_for_unchosen)
"""Cognitive model describing rat behavior on a binary two-armed bandit task. Args: params: Model params. Different parameters are used for different...
*** choice_logits = jnp.zeros_like(base_logits.at[choice].add(persistence_bonus))
*** ...ence, persistence_reward_effect, forgetting_rate_raw, bias_choice_0, recency_decay_rate_raw, recency_win_weight, recency_loss_weight = jnp.zeros_like(params)
*** total_learned_value = jnp.zeros_like(value_with_bias + new_net_recency)
*** base_logits = jnp.zeros_like(beta * total_learned_value)
* new_q_values = jnp.zeros_like(new_q_values.at[1 - choice].set(updated_q_for_unchosen))
* prediction_error = jnp.zeros_like(reward - old_q_values[choice])
* updated_q_for_unchosen = jnp.zeros_like(q_values_after_forgetting[1 - choice] - alpha_unchosen * prediction_error)
** value_with_bias = jnp.zeros_like(new_q_values.at[0].add(bias_choice_0))
*** new_agent_state = jnp.zeros_like(jnp.concatenate([new_q_values, new_net_recency]))
* old_q_values = jnp.zeros_like(agent_state[:2])
* new_q_values = jnp.zeros_like(q_values_after_forgetting.at[choice].set(updated_q_for_chosen))
* q_values_after_forgetting = jnp.zeros_like(old_q_values * (1.0 - forgetting_rate))
* new_net_recency = jnp.zeros_like(recency_after_decay.at[choice].add(recency_increment))
* updated_q_for_chosen = jnp.zeros_like(q_values_after_forgetting[choice] + alpha_chosen * prediction_error)
* recency_increment = jnp.zeros_like(recency_update_value * (1.0 - recency_decay_rate))
* alpha_unchosen = jnp.zeros_like(jax.nn.sigmoid(alpha_unchosen_raw))
* recency_update_value = jnp.zeros_like(reward * recency_win_weight - recency_loss_weight)
* alpha_chosen = jnp.zeros_like(jax.nn.sigmoid(alpha_chosen_raw))
initial_net_recency = jnp.zeros_like(jnp.array([0.0, 0.0]))
old_net_recency = jnp.zeros_like(agent_state[2:4])
recency_after_decay = jnp.zeros_like(old_net_recency * recency_decay_rate)
initial_q_values = jnp.zeros_like(jnp.array([0.5, 0.5]))
recency_decay_rate = jnp.zeros_like(jax.nn.sigmoid(recency_decay_rate_raw))
persistence_bonus = jnp.zeros_like(persistence + persistence_reward_effect * reward)
agent_state = jnp.zeros_like(jnp.concatenate([initial_q_values, initial_net_recency]))
forgetting_rate = jnp.zeros_like(jax.nn.sigmoid(forgetting_rate_raw))
* updated_q_for_unchosen = q_values_after_forgetting[1 - choice] - alpha_unchosen * prediction_error
** total_learned_value = value_with_bias + new_net_recency
* updated_q_for_unchosen = q_values_after_forgetting[1 - choice] - alpha_unchosen * prediction_error
* new_q_values = new_q_values.at[1 - choice].set(updated_q_for_unchosen)
* updated_q_for_unchosen = q_values_after_forgetting[1 - choice] - alpha_unchosen * prediction_error
* updated_q_for_chosen = q_values_after_forgetting[choice] + alpha_chosen * prediction_error
* updated_q_for_unchosen = q_values_after_forgetting[1 - choice] - alpha_unchosen * prediction_error
recency_increment = recency_update_value * (1.0 - recency_decay_rate)
recency_update_value = reward * recency_win_weight - recency_loss_weight
* new_q_values = new_q_values.at[1 - choice].set(updated_q_for_unchosen)
** updated_q_for_unchosen = q_values_after_forgetting[1 - choice] - alpha_unchosen * prediction_error
* updated_q_for_chosen = q_values_after_forgetting[choice] + alpha_chosen * prediction_error
recency_update_value = reward * recency_win_weight - recency_loss_weight
recency_update_value = reward * recency_win_weight - recency_loss_weight
* updated_q_for_chosen = q_values_after_forgetting[choice] + alpha_chosen * prediction_error
base_logits = beta * total_learned_value
recency_update_value = reward * recency_win_weight - recency_loss_weight
* updated_q_for_unchosen = q_values_after_forgetting[1 - choice] - alpha_unchosen * prediction_error
persistence_bonus = persistence + persistence_reward_effect * reward
* total_learned_value = value_with_bias + new_net_recency
* recency_increment = recency_update_value * (1.0 - recency_decay_rate)
* prediction_error = reward - old_q_values[choice]
* prediction_error = reward - old_q_values[choice]
persistence_bonus = persistence + persistence_reward_effect * reward
q_values_after_forgetting = old_q_values * (1.0 - forgetting_rate)
* recency_increment = recency_update_value * (1.0 - recency_decay_rate)
* updated_q_for_chosen = q_values_after_forgetting[choice] + alpha_chosen * prediction_error
* recency_increment = recency_update_value * (1.0 - recency_decay_rate)
persistence_bonus = persistence + persistence_reward_effect * reward
* recency_after_decay = old_net_recency * recency_decay_rate
persistence_bonus = persistence + persistence_reward_effect * reward
q_values_after_forgetting = old_q_values * (1.0 - forgetting_rate)
q_values_after_forgetting = old_q_values * (1.0 - forgetting_rate)
−0.2
−0.1
0.0
