# Supplementary material for: AI-Discovered Cognitive Models Reveal Novel Insights into Human and Animal Learning
Source: bioRxiv. 2026 May 21:2026.05.18.725921. Preprint. [Version 1] doi: 10.64898/2026.05.18.725921 (PMC13228651; doi:10.64898/2026.05.18.725921)
Supplement: Supplement 2 [file media-2.zip › ablation_performance_rat_twostep_run3_low_floor_20260420.pdf]

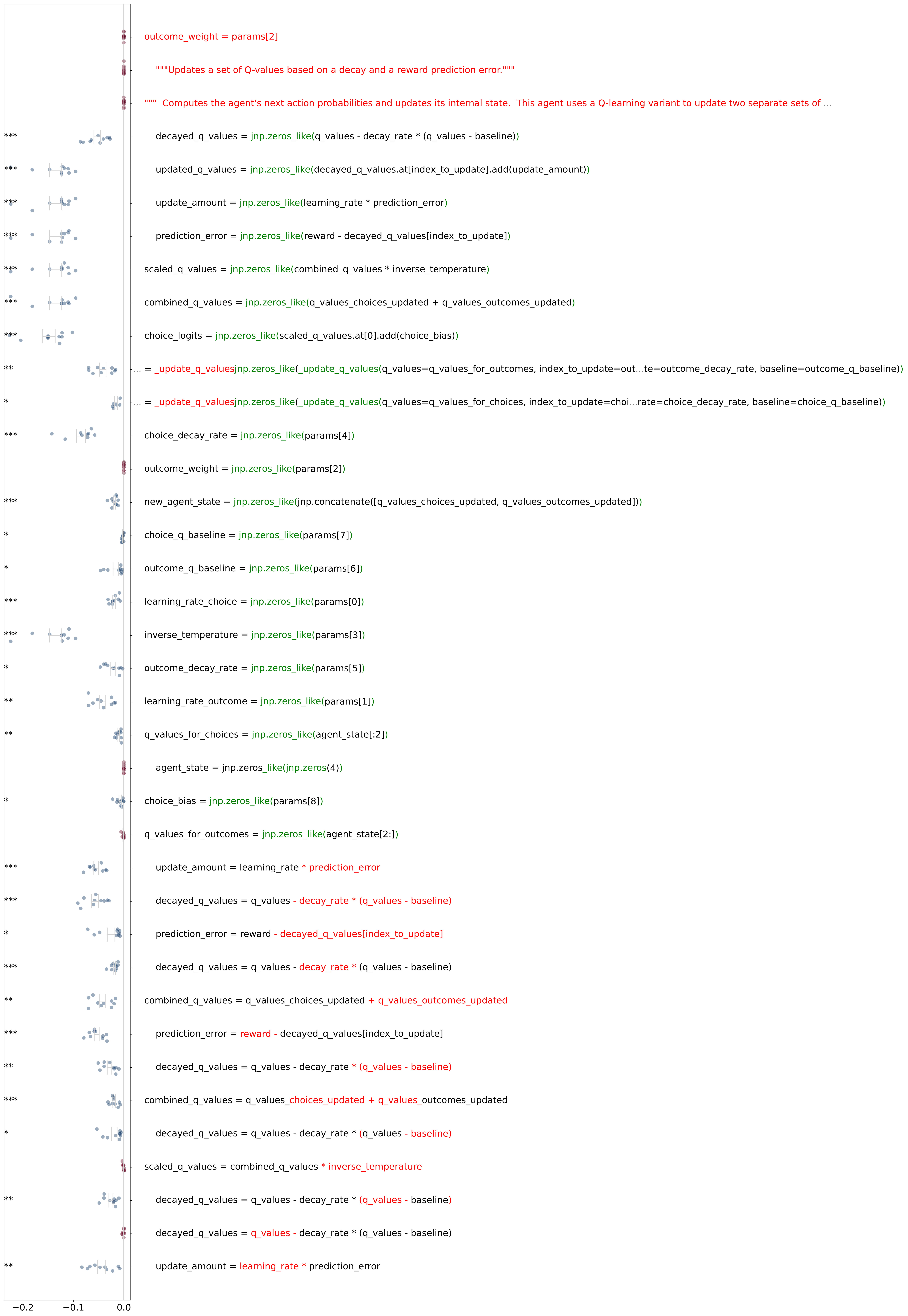
outcome_weight = params[2]
"""Updates a set of Q-values based on a decay and a reward prediction error."""
""" Computes the agent's next action probabilities and updates its internal state. This agent uses a Q-learning variant to update two separate sets of ...
decayed_q_values = jnp.zeros_like(q_values - decay_rate * (q_values - baseline))
updated_q_values = jnp.zeros_like(decayed_q_values.at[index_to_update].add(update_amount))
update_amount = jnp.zeros_like(learning_rate * prediction_error)
prediction_error = jnp.zeros_like(reward - decayed_q_values[index_to_update])
scaled_q_values = jnp.zeros_like(combined_q_values * inverse_temperature)
combined_q_values = jnp.zeros_like(q_values_choices_updated + q_values_outcomes_updated)
choice_logits = jnp.zeros_like(scaled_q_values.at[0].add(choice_bias))
... = _update_q_valuesjnp.zeros_like(_update_q_values(q_values=q_values_for_outcomes, index_to_update=out...te=outcome_decay_rate, baseline=outcome_q_baseline))
... = _update_q_valuesjnp.zeros_like(_update_q_values(q_values=q_values_for_choices, index_to_update=choi...rate=choice_decay_rate, baseline=choice_q_baseline))
choice_decay_rate = jnp.zeros_like(params[4])
outcome_weight = jnp.zeros_like(params[2])
new_agent_state = jnp.zeros_like(jnp.concatenate([q_values_choices_updated, q_values_outcomes_updated]))
choice_q_baseline = jnp.zeros_like(params[7])
outcome_q_baseline = jnp.zeros_like(params[6])
learning_rate_choice = jnp.zeros_like(params[0])
inverse_temperature = jnp.zeros_like(params[3])
outcome_decay_rate = jnp.zeros_like(params[5])
learning_rate_outcome = jnp.zeros_like(params[1])
q_values_for_choices = jnp.zeros_like(agent_state[:2])
agent_state = jnp.zeros_like(jnp.zeros(4))
choice_bias = jnp.zeros_like(params[8])
q_values_for_outcomes = jnp.zeros_like(agent_state[2:])
update_amount = learning_rate * prediction_error
decayed_q_values = q_values - decay_rate * (q_values - baseline)
prediction_error = reward - decayed_q_values[index_to_update]
decayed_q_values = q_values - decay_rate * (q_values - baseline)
combined_q_values = q_values_choices_updated + q_values_outcomes_updated
prediction_error = reward - decayed_q_values[index_to_update]
decayed_q_values = q_values - decay_rate * (q_values - baseline)
combined_q_values = q_values_choices_updated + q_values_outcomes_updated
decayed_q_values = q_values - decay_rate * (q_values - baseline)
scaled_q_values = combined_q_values * inverse_temperature
decayed_q_values = q_values - decay_rate * (q_values - baseline)
decayed_q_values = q_values - decay_rate * (q_values - baseline)
update_amount = learning_rate * prediction_error
−0.2
−0.1
0.0
