# Supplementary material for: AI-Discovered Cognitive Models Reveal Novel Insights into Human and Animal Learning
Source: bioRxiv. 2026 May 21:2026.05.18.725921. Preprint. [Version 1] doi: 10.64898/2026.05.18.725921 (PMC13228651; doi:10.64898/2026.05.18.725921)
Supplement: Supplement 2 [file media-2.zip › ablation_performance_rat_twostep_run1_low_floor_20260420.pdf]

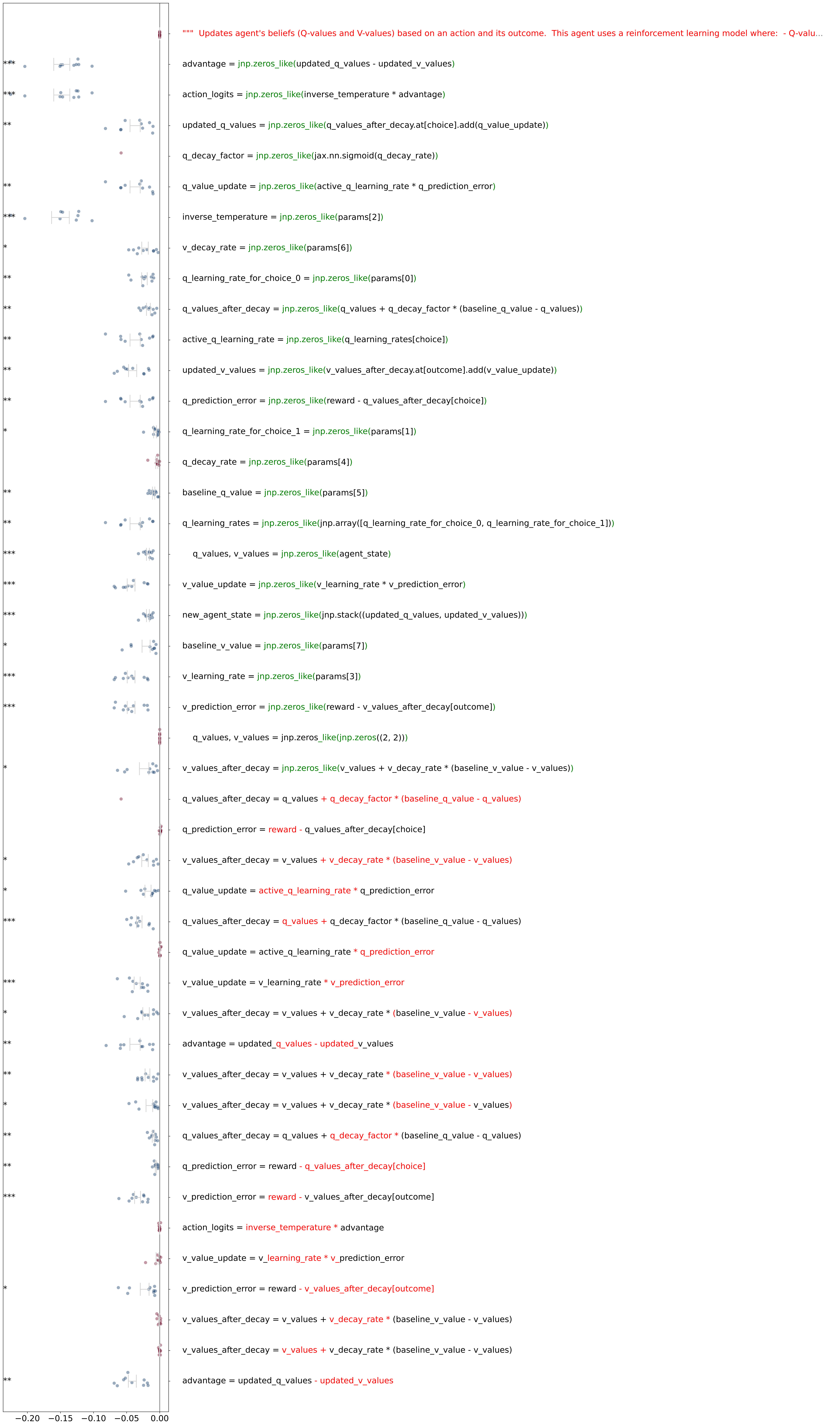
""" Updates agent's beliefs (Q-values and V-values) based on an action and its outcome. This agent uses a reinforcement learning model where: - Q-valu...
advantage = jnp.zeros_like(updated_q_values - updated_v_values)
action_logits = jnp.zeros_like(inverse_temperature * advantage)
updated_q_values = jnp.zeros_like(q_values_after_decay.at[choice].add(q_value_update))
q_decay_factor = jnp.zeros_like(jax.nn.sigmoid(q_decay_rate))
q_value_update = jnp.zeros_like(active_q_learning_rate * q_prediction_error)
inverse_temperature = jnp.zeros_like(params[2])
v_decay_rate = jnp.zeros_like(params[6])
q_learning_rate_for_choice_0 = jnp.zeros_like(params[0])
q_values_after_decay = jnp.zeros_like(q_values + q_decay_factor * (baseline_q_value - q_values))
active_q_learning_rate = jnp.zeros_like(q_learning_rates[choice])
updated_v_values = jnp.zeros_like(v_values_after_decay.at[outcome].add(v_value_update))
q_prediction_error = jnp.zeros_like(reward - q_values_after_decay[choice])
q_learning_rate_for_choice_1 = jnp.zeros_like(params[1])
q_decay_rate = jnp.zeros_like(params[4])
baseline_q_value = jnp.zeros_like(params[5])
q_learning_rates = jnp.zeros_like(jnp.array([q_learning_rate_for_choice_0, q_learning_rate_for_choice_1]))
q_values, v_values = jnp.zeros_like(agent_state)
v_value_update = jnp.zeros_like(v_learning_rate * v_prediction_error)
new_agent_state = jnp.zeros_like(jnp.stack((updated_q_values, updated_v_values)))
baseline_v_value = jnp.zeros_like(params[7])
v_learning_rate = jnp.zeros_like(params[3])
v_prediction_error = jnp.zeros_like(reward - v_values_after_decay[outcome])
q_values, v_values = jnp.zeros_like(jnp.zeros((2, 2)))
v_values_after_decay = jnp.zeros_like(v_values + v_decay_rate * (baseline_v_value - v_values))
q_values_after_decay = q_values + q_decay_factor * (baseline_q_value - q_values)
q_prediction_error = reward - q_values_after_decay[choice]
v_values_after_decay = v_values + v_decay_rate * (baseline_v_value - v_values)
q_value_update = active_q_learning_rate * q_prediction_error
q_values_after_decay = q_values + q_decay_factor * (baseline_q_value - q_values)
q_value_update = active_q_learning_rate * q_prediction_error
v_value_update = v_learning_rate * v_prediction_error
v_values_after_decay = v_values + v_decay_rate * (baseline_v_value - v_values)
advantage = updated_q_values - updated_v_values
v_values_after_decay = v_values + v_decay_rate * (baseline_v_value - v_values)
v_values_after_decay = v_values + v_decay_rate * (baseline_v_value - v_values)
q_values_after_decay = q_values + q_decay_factor * (baseline_q_value - q_values)
q_prediction_error = reward - q_values_after_decay[choice]
v_prediction_error = reward - v_values_after_decay[outcome]
action_logits = inverse_temperature * advantage
v_value_update = v_learning_rate * v_prediction_error
v_prediction_error = reward - v_values_after_decay[outcome]
v_values_after_decay = v_values + v_decay_rate * (baseline_v_value - v_values)
v_values_after_decay = v_values + v_decay_rate * (baseline_v_value - v_values)
advantage = updated_q_values - updated_v_values
−0.20 −0.15 −0.10 −0.05 0.00
