# Supplementary material for: AI-Discovered Cognitive Models Reveal Novel Insights into Human and Animal Learning
Source: bioRxiv. 2026 May 21:2026.05.18.725921. Preprint. [Version 1] doi: 10.64898/2026.05.18.725921 (PMC13228651; doi:10.64898/2026.05.18.725921)
Supplement: Supplement 2 [file media-2.zip › ablation_performance_fly_bandit_run3_low_floor_20260420.pdf]

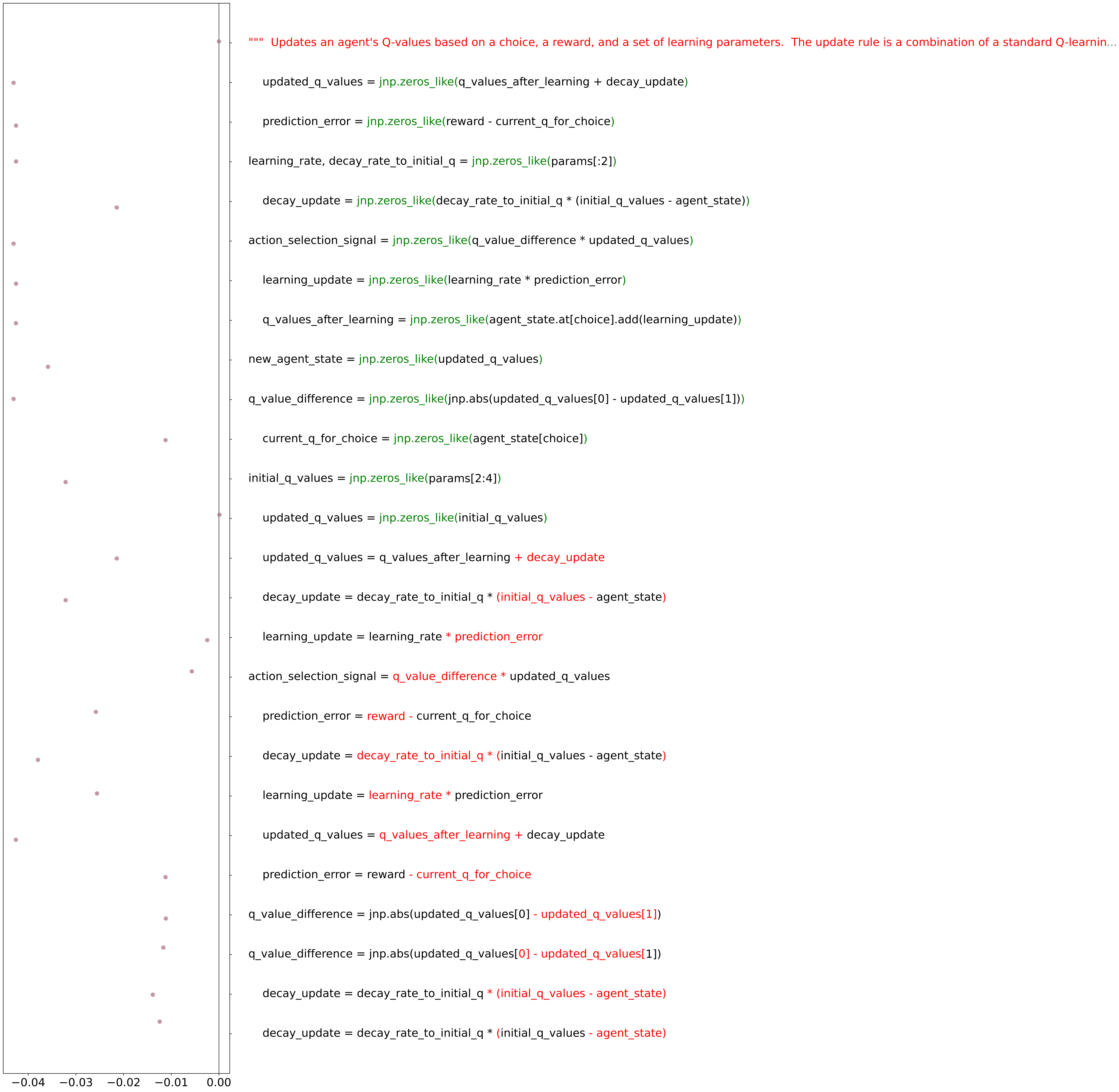

""" Updates an agent's Q-values based on a choice, a reward, and a set of learning parameters. The update rule is a combination of a standard Q-learnin...
updated_q_values = jnp.zeros_like(q_values_after_learning + decay_update)
prediction_error = jnp.zeros_like(reward - current_q_for_choice)
learning_rate, decay_rate_to_initial_q = jnp.zeros_like(params[:2])
decay_update = jnp.zeros_like(decay_rate_to_initial_q * (initial_q_values - agent_state))
action_selection_signal = jnp.zeros_like(q_value_difference * updated_q_values)
learning_update = jnp.zeros_like(learning_rate * prediction_error)
q_values_after_learning = jnp.zeros_like(agent_state.at[choice].add(learning_update))
new_agent_state = jnp.zeros_like(updated_q_values)
q_value_difference = jnp.zeros_like(jnp.abs(updated_q_values[0] - updated_q_values[1]))
current_q_for_choice = jnp.zeros_like(agent_state[choice])
initial_q_values = jnp.zeros_like(params[2:4])
updated_q_values = jnp.zeros_like(initial_q_values)
updated_q_values = q_values_after_learning + decay_update
decay_update = decay_rate_to_initial_q * (initial_q_values - agent_state)
learning_update = learning_rate * prediction_error
action_selection_signal = q_value_difference * updated_q_values
prediction_error = reward - current_q_for_choice
decay_update = decay_rate_to_initial_q * (initial_q_values - agent_state)
learning_update = learning_rate * prediction_error
updated_q_values = q_values_after_learning + decay_update
prediction_error = reward - current_q_for_choice
q_value_difference = jnp.abs(updated_q_values[0] - updated_q_values[1])
q_value_difference = jnp.abs(updated_q_values[0] - updated_q_values[1])
decay_update = decay_rate_to_initial_q * (initial_q_values - agent_state)
decay_update = decay_rate_to_initial_q * (initial_q_values - agent_state)
−0.04
−0.03
−0.02
−0.01
0.00
