# Supplementary material for: AI-Discovered Cognitive Models Reveal Novel Insights into Human and Animal Learning
Source: bioRxiv. 2026 May 21:2026.05.18.725921. Preprint. [Version 1] doi: 10.64898/2026.05.18.725921 (PMC13228651; doi:10.64898/2026.05.18.725921)
Supplement: Supplement 2 [file media-2.zip › ablation_performance_fly_bandit_run1_low_floor_20260420.pdf]

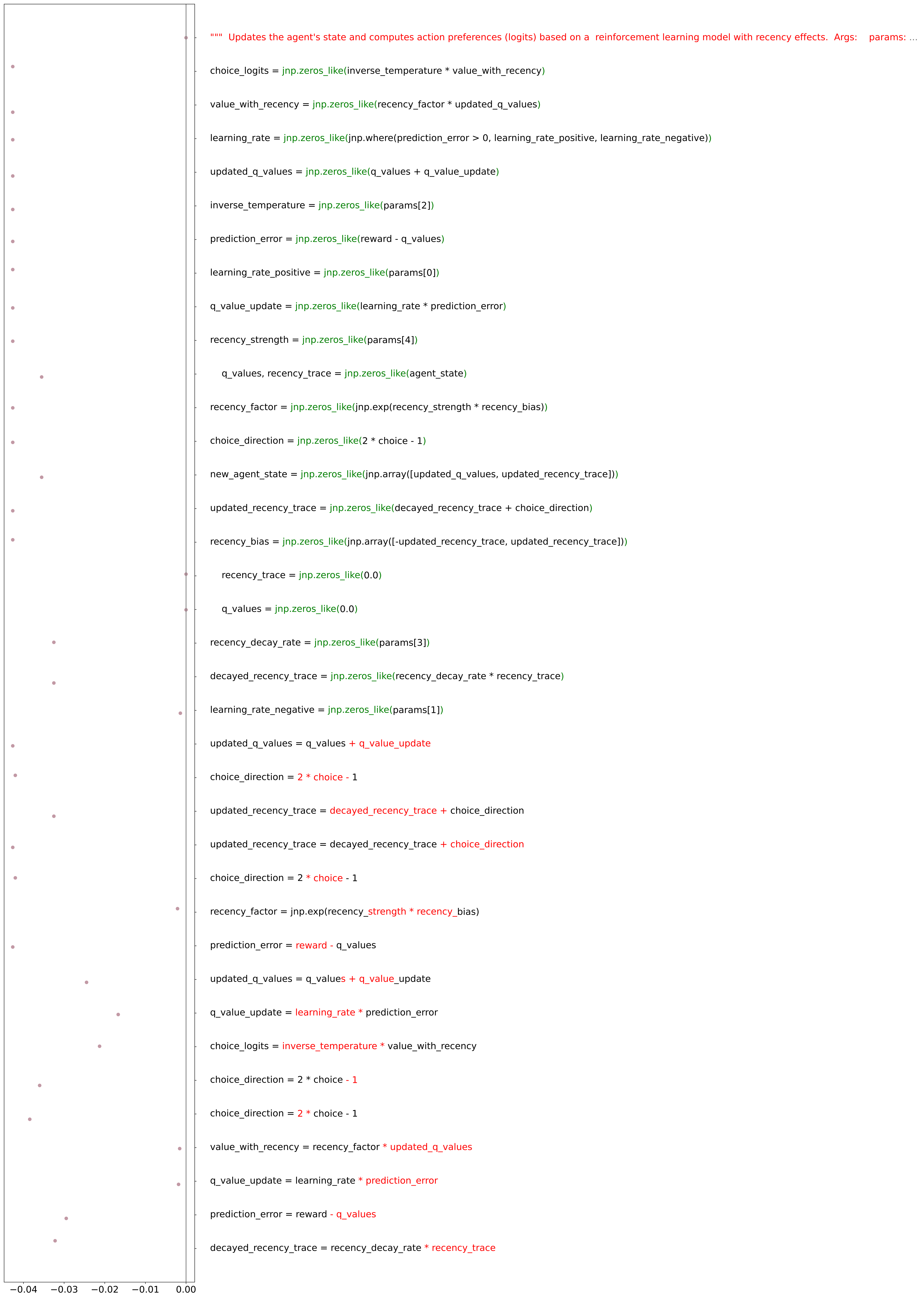

""" Updates the agent's state and computes action preferences (logits) based on a reinforcement learning model with recency effects. Args: params: ...
choice_logits = jnp.zeros_like(inverse_temperature * value_with_recency)
value_with_recency = jnp.zeros_like(recency_factor * updated_q_values)
learning_rate = jnp.zeros_like(jnp.where(prediction_error > 0, learning_rate_positive, learning_rate_negative))
updated_q_values = jnp.zeros_like(q_values + q_value_update)
inverse_temperature = jnp.zeros_like(params[2])
prediction_error = jnp.zeros_like(reward - q_values)
learning_rate_positive = jnp.zeros_like(params[0])
q_value_update = jnp.zeros_like(learning_rate * prediction_error)
recency_strength = jnp.zeros_like(params[4])
q_values, recency_trace = jnp.zeros_like(agent_state)
recency_factor = jnp.zeros_like(jnp.exp(recency_strength * recency_bias))
choice_direction = jnp.zeros_like(2 * choice - 1)
new_agent_state = jnp.zeros_like(jnp.array([updated_q_values, updated_recency_trace]))
updated_recency_trace = jnp.zeros_like(decayed_recency_trace + choice_direction)
recency_bias = jnp.zeros_like(jnp.array([-updated_recency_trace, updated_recency_trace]))
recency_trace = jnp.zeros_like(0.0)
q_values = jnp.zeros_like(0.0)
recency_decay_rate = jnp.zeros_like(params[3])
decayed_recency_trace = jnp.zeros_like(recency_decay_rate * recency_trace)
learning_rate_negative = jnp.zeros_like(params[1])
updated_q_values = q_values + q_value_update
choice_direction = 2 * choice - 1
updated_recency_trace = decayed_recency_trace + choice_direction
updated_recency_trace = decayed_recency_trace + choice_direction
choice_direction = 2 * choice - 1
recency_factor = jnp.exp(recency_strength * recency_bias)
prediction_error = reward - q_values
updated_q_values = q_values + q_value_update
q_value_update = learning_rate * prediction_error
choice_logits = inverse_temperature * value_with_recency
choice_direction = 2 * choice - 1
choice_direction = 2 * choice - 1
value_with_recency = recency_factor * updated_q_values
q_value_update = learning_rate * prediction_error
prediction_error = reward - q_values
decayed_recency_trace = recency_decay_rate * recency_trace
−0.04
−0.03
−0.02
−0.01
0.00
