# Supplementary material for: AI-Discovered Cognitive Models Reveal Novel Insights into Human and Animal Learning
Source: bioRxiv. 2026 May 21:2026.05.18.725921. Preprint. [Version 1] doi: 10.64898/2026.05.18.725921 (PMC13228651; doi:10.64898/2026.05.18.725921)
Supplement: Supplement 2 [file media-2.zip › ablation_performance_monkey_bandit_run1_high_floor_refactored_20260420.pdf]

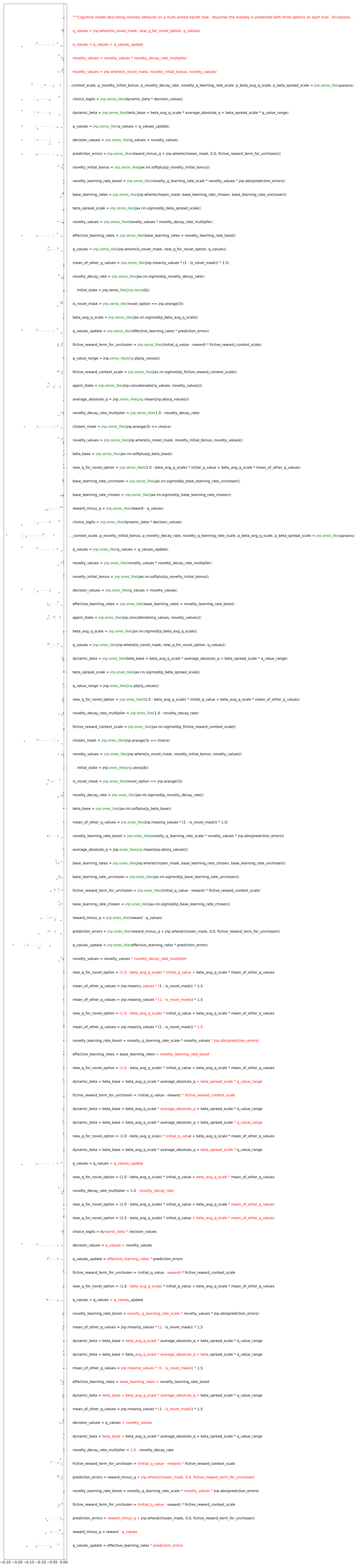
"""Cognitive model describing monkey behavior on a multi-armed bandit task. Assumes the monkey is presented with three options on each trial. Occasiona...
q_values = jnp.where(is_novel_mask, new_q_for_novel_option, q_values)
q_values = q_values + q_values_update
novelty_values = novelty_values * novelty_decay_rate_multiplier
novelty_values = jnp.where(is_novel_mask, novelty_initial_bonus, novelty_values)
context_scale, p_novelty_initial_bonus, p_novelty_decay_rate, novelty_q_learning_rate_scale, p_beta_avg_q_scale, p_beta_spread_scale = jnp.zeros_like(params)
choice_logits = jnp.zeros_like(dynamic_beta * decision_values)
dynamic_beta = jnp.zeros_like(beta_base + beta_avg_q_scale * average_absolute_q + beta_spread_scale * q_value_range)
q_values = jnp.zeros_like(q_values + q_values_update)
decision_values = jnp.zeros_like(q_values + novelty_values)
prediction_errors = jnp.zeros_like(reward_minus_q + jnp.where(chosen_mask, 0.0, fictive_reward_term_for_unchosen))
novelty_initial_bonus = jnp.zeros_like(jax.nn.softplus(p_novelty_initial_bonus))
novelty_learning_rate_boost = jnp.zeros_like(novelty_q_learning_rate_scale * novelty_values * jnp.abs(prediction_errors))
base_learning_rates = jnp.zeros_like(jnp.where(chosen_mask, base_learning_rate_chosen, base_learning_rate_unchosen))
beta_spread_scale = jnp.zeros_like(jax.nn.sigmoid(p_beta_spread_scale))
novelty_values = jnp.zeros_like(novelty_values * novelty_decay_rate_multiplier)
effective_learning_rates = jnp.zeros_like(base_learning_rates + novelty_learning_rate_boost)
q_values = jnp.zeros_like(jnp.where(is_novel_mask, new_q_for_novel_option, q_values))
mean_of_other_q_values = jnp.zeros_like(jnp.mean(q_values * (1 - is_novel_mask)) * 1.5)
novelty_decay_rate = jnp.zeros_like(jax.nn.sigmoid(p_novelty_decay_rate))
initial_state = jnp.zeros_like(jnp.zeros(6))
is_novel_mask = jnp.zeros_like(novel_option == jnp.arange(3))
beta_avg_q_scale = jnp.zeros_like(jax.nn.sigmoid(p_beta_avg_q_scale))
q_values_update = jnp.zeros_like(effective_learning_rates * prediction_errors)
fictive_reward_term_for_unchosen = jnp.zeros_like((initial_q_value - reward) * fictive_reward_context_scale)
q_value_range = jnp.zeros_like(jnp.ptp(q_values))
fictive_reward_context_scale = jnp.zeros_like(jax.nn.sigmoid(p_fictive_reward_context_scale))
agent_state = jnp.zeros_like(jnp.concatenate((q_values, novelty_values)))
average_absolute_q = jnp.zeros_like(jnp.mean(jnp.abs(q_values)))
novelty_decay_rate_multiplier = jnp.zeros_like(1.0 - novelty_decay_rate)
chosen_mask = jnp.zeros_like(jnp.arange(3) == choice)
novelty_values = jnp.zeros_like(jnp.where(is_novel_mask, novelty_initial_bonus, novelty_values))
beta_base = jnp.zeros_like(jax.nn.softplus(p_beta_base))
new_q_for_novel_option = jnp.zeros_like((1.0 - beta_avg_q_scale) * initial_q_value + beta_avg_q_scale * mean_of_other_q_values)
base_learning_rate_unchosen = jnp.zeros_like(jax.nn.sigmoid(p_base_learning_rate_unchosen))
base_learning_rate_chosen = jnp.zeros_like(jax.nn.sigmoid(p_base_learning_rate_chosen))
reward_minus_q = jnp.zeros_like(reward - q_values)
choice_logits = jnp.ones_like(dynamic_beta * decision_values)
context_scale, p_novelty_initial_bonus, p_novelty_decay_rate, novelty_q_learning_rate_scale, p_beta_avg_q_scale, p_beta_spread_scale = jnp.ones_like(params)
q_values = jnp.ones_like(q_values + q_values_update)
novelty_values = jnp.ones_like(novelty_values * novelty_decay_rate_multiplier)
novelty_initial_bonus = jnp.ones_like(jax.nn.softplus(p_novelty_initial_bonus))
decision_values = jnp.ones_like(q_values + novelty_values)
effective_learning_rates = jnp.ones_like(base_learning_rates + novelty_learning_rate_boost)
agent_state = jnp.ones_like(jnp.concatenate((q_values, novelty_values)))
beta_avg_q_scale = jnp.ones_like(jax.nn.sigmoid(p_beta_avg_q_scale))
q_values = jnp.ones_like(jnp.where(is_novel_mask, new_q_for_novel_option, q_values))
dynamic_beta = jnp.ones_like(beta_base + beta_avg_q_scale * average_absolute_q + beta_spread_scale * q_value_range)
beta_spread_scale = jnp.ones_like(jax.nn.sigmoid(p_beta_spread_scale))
q_value_range = jnp.ones_like(jnp.ptp(q_values))
new_q_for_novel_option = jnp.ones_like((1.0 - beta_avg_q_scale) * initial_q_value + beta_avg_q_scale * mean_of_other_q_values)
novelty_decay_rate_multiplier = jnp.ones_like(1.0 - novelty_decay_rate)
fictive_reward_context_scale = jnp.ones_like(jax.nn.sigmoid(p_fictive_reward_context_scale))
chosen_mask = jnp.ones_like(jnp.arange(3) == choice)
novelty_values = jnp.ones_like(jnp.where(is_novel_mask, novelty_initial_bonus, novelty_values))
initial_state = jnp.ones_like(jnp.zeros(6))
is_novel_mask = jnp.ones_like(novel_option == jnp.arange(3))
novelty_decay_rate = jnp.ones_like(jax.nn.sigmoid(p_novelty_decay_rate))
beta_base = jnp.ones_like(jax.nn.softplus(p_beta_base))
mean_of_other_q_values = jnp.ones_like(jnp.mean(q_values * (1 - is_novel_mask)) * 1.5)
novelty_learning_rate_boost = jnp.ones_like(novelty_q_learning_rate_scale * novelty_values * jnp.abs(prediction_errors))
average_absolute_q = jnp.ones_like(jnp.mean(jnp.abs(q_values)))
base_learning_rates = jnp.ones_like(jnp.where(chosen_mask, base_learning_rate_chosen, base_learning_rate_unchosen))
base_learning_rate_unchosen = jnp.ones_like(jax.nn.sigmoid(p_base_learning_rate_unchosen))
fictive_reward_term_for_unchosen = jnp.ones_like((initial_q_value - reward) * fictive_reward_context_scale)
base_learning_rate_chosen = jnp.ones_like(jax.nn.sigmoid(p_base_learning_rate_chosen))
reward_minus_q = jnp.ones_like(reward - q_values)
prediction_errors = jnp.ones_like(reward_minus_q + jnp.where(chosen_mask, 0.0, fictive_reward_term_for_unchosen))
q_values_update = jnp.ones_like(effective_learning_rates * prediction_errors)
novelty_values = novelty_values * novelty_decay_rate_multiplier
new_q_for_novel_option = (1.0 - beta_avg_q_scale) * initial_q_value + beta_avg_q_scale * mean_of_other_q_values
mean_of_other_q_values = jnp.mean(q_values * (1 - is_novel_mask)) * 1.5
mean_of_other_q_values = jnp.mean(q_values * (1 - is_novel_mask)) * 1.5
new_q_for_novel_option = (1.0 - beta_avg_q_scale) * initial_q_value + beta_avg_q_scale * mean_of_other_q_values
mean_of_other_q_values = jnp.mean(q_values * (1 - is_novel_mask)) * 1.5
novelty_learning_rate_boost = novelty_q_learning_rate_scale * novelty_values * jnp.abs(prediction_errors)
effective_learning_rates = base_learning_rates + novelty_learning_rate_boost
new_q_for_novel_option = (1.0 - beta_avg_q_scale) * initial_q_value + beta_avg_q_scale * mean_of_other_q_values
dynamic_beta = beta_base + beta_avg_q_scale * average_absolute_q + beta_spread_scale * q_value_range
fictive_reward_term_for_unchosen = (initial_q_value - reward) * fictive_reward_context_scale
dynamic_beta = beta_base + beta_avg_q_scale * average_absolute_q + beta_spread_scale * q_value_range
dynamic_beta = beta_base + beta_avg_q_scale * average_absolute_q + beta_spread_scale * q_value_range
new_q_for_novel_option = (1.0 - beta_avg_q_scale) * initial_q_value + beta_avg_q_scale * mean_of_other_q_values
dynamic_beta = beta_base + beta_avg_q_scale * average_absolute_q + beta_spread_scale * q_value_range
q_values = q_values + q_values_update
new_q_for_novel_option = (1.0 - beta_avg_q_scale) * initial_q_value + beta_avg_q_scale * mean_of_other_q_values
novelty_decay_rate_multiplier = 1.0 - novelty_decay_rate
new_q_for_novel_option = (1.0 - beta_avg_q_scale) * initial_q_value + beta_avg_q_scale * mean_of_other_q_values
new_q_for_novel_option = (1.0 - beta_avg_q_scale) * initial_q_value + beta_avg_q_scale * mean_of_other_q_values
choice_logits = dynamic_beta * decision_values
decision_values = q_values + novelty_values
q_values_update = effective_learning_rates * prediction_errors
fictive_reward_term_for_unchosen = (initial_q_value - reward) * fictive_reward_context_scale
new_q_for_novel_option = (1.0 - beta_avg_q_scale) * initial_q_value + beta_avg_q_scale * mean_of_other_q_values
q_values = q_values + q_values_update
novelty_learning_rate_boost = novelty_q_learning_rate_scale * novelty_values * jnp.abs(prediction_errors)
mean_of_other_q_values = jnp.mean(q_values * (1 - is_novel_mask)) * 1.5
dynamic_beta = beta_base + beta_avg_q_scale * average_absolute_q + beta_spread_scale * q_value_range
dynamic_beta = beta_base + beta_avg_q_scale * average_absolute_q + beta_spread_scale * q_value_range
mean_of_other_q_values = jnp.mean(q_values * (1 - is_novel_mask)) * 1.5
effective_learning_rates = base_learning_rates + novelty_learning_rate_boost
dynamic_beta = beta_base + beta_avg_q_scale * average_absolute_q + beta_spread_scale * q_value_range
mean_of_other_q_values = jnp.mean(q_values * (1 - is_novel_mask)) * 1.5
decision_values = q_values + novelty_values
dynamic_beta = beta_base + beta_avg_q_scale * average_absolute_q + beta_spread_scale * q_value_range
novelty_decay_rate_multiplier = 1.0 - novelty_decay_rate
fictive_reward_term_for_unchosen = (initial_q_value - reward) * fictive_reward_context_scale
prediction_errors = reward_minus_q + jnp.where(chosen_mask, 0.0, fictive_reward_term_for_unchosen)
novelty_learning_rate_boost = novelty_q_learning_rate_scale * novelty_values * jnp.abs(prediction_errors)
fictive_reward_term_for_unchosen = (initial_q_value - reward) * fictive_reward_context_scale
prediction_errors = reward_minus_q + jnp.where(chosen_mask, 0.0, fictive_reward_term_for_unchosen)
reward_minus_q = reward - q_values
q_values_update = effective_learning_rates * prediction_errors
−0.25
−0.20
−0.15
−0.10
−0.05
0.00
