# Supplementary material for: AI-Discovered Cognitive Models Reveal Novel Insights into Human and Animal Learning
Source: bioRxiv. 2026 May 21:2026.05.18.725921. Preprint. [Version 1] doi: 10.64898/2026.05.18.725921 (PMC13228651; doi:10.64898/2026.05.18.725921)
Supplement: Supplement 2 [file media-2.zip › ablation_performance_rat_bandit_run3_low_floor_20260420.pdf]

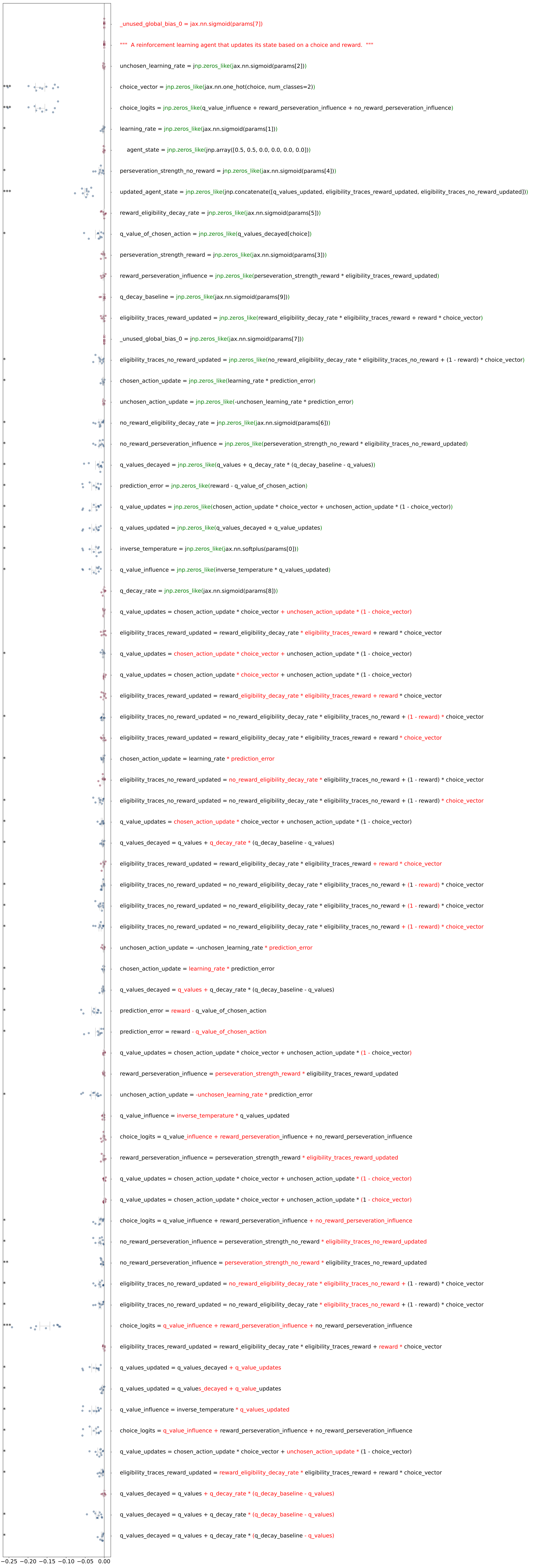

_unused_global_bias_0 = jax.nn.sigmoid(params[7])
""" A reinforcement learning agent that updates its state based on a choice and reward. """
unchosen_learning_rate = jnp.zeros_like(jax.nn.sigmoid(params[2]))
*** choice_vector = jnp.zeros_like(jax.nn.one_hot(choice, num_classes=2))
*** choice_logits = jnp.zeros_like(q_value_influence + reward_perseveration_influence + no_reward_perseveration_influence)
* learning_rate = jnp.zeros_like(jax.nn.sigmoid(params[1]))
agent_state = jnp.zeros_like(jnp.array([0.5, 0.5, 0.0, 0.0, 0.0, 0.0]))
* perseveration_strength_no_reward = jnp.zeros_like(jax.nn.sigmoid(params[4]))
*** updated_agent_state = jnp.zeros_like(jnp.concatenate([q_values_updated, eligibility_traces_reward_updated, eligibility_traces_no_reward_updated]))
reward_eligibility_decay_rate = jnp.zeros_like(jax.nn.sigmoid(params[5]))
* q_value_of_chosen_action = jnp.zeros_like(q_values_decayed[choice])
perseveration_strength_reward = jnp.zeros_like(jax.nn.sigmoid(params[3]))
reward_perseveration_influence = jnp.zeros_like(perseveration_strength_reward * eligibility_traces_reward_updated)
q_decay_baseline = jnp.zeros_like(jax.nn.sigmoid(params[9]))
eligibility_traces_reward_updated = jnp.zeros_like(reward_eligibility_decay_rate * eligibility_traces_reward + reward * choice_vector)
_unused_global_bias_0 = jnp.zeros_like(jax.nn.sigmoid(params[7]))
* eligibility_traces_no_reward_updated = jnp.zeros_like(no_reward_eligibility_decay_rate * eligibility_traces_no_reward + (1 - reward) * choice_vector)
* chosen_action_update = jnp.zeros_like(learning_rate * prediction_error)
unchosen_action_update = jnp.zeros_like(-unchosen_learning_rate * prediction_error)
* no_reward_eligibility_decay_rate = jnp.zeros_like(jax.nn.sigmoid(params[6]))
* no_reward_perseveration_influence = jnp.zeros_like(perseveration_strength_no_reward * eligibility_traces_no_reward_updated)
* q_values_decayed = jnp.zeros_like(q_values + q_decay_rate * (q_decay_baseline - q_values))
* prediction_error = jnp.zeros_like(reward - q_value_of_chosen_action)
* q_value_updates = jnp.zeros_like(chosen_action_update * choice_vector + unchosen_action_update * (1 - choice_vector))
* q_values_updated = jnp.zeros_like(q_values_decayed + q_value_updates)
* inverse_temperature = jnp.zeros_like(jax.nn.softplus(params[0]))
* q_value_influence = jnp.zeros_like(inverse_temperature * q_values_updated)
q_decay_rate = jnp.zeros_like(jax.nn.sigmoid(params[8]))
q_value_updates = chosen_action_update * choice_vector + unchosen_action_update * (1 - choice_vector)
eligibility_traces_reward_updated = reward_eligibility_decay_rate * eligibility_traces_reward + reward * choice_vector
* q_value_updates = chosen_action_update * choice_vector + unchosen_action_update * (1 - choice_vector)
q_value_updates = chosen_action_update * choice_vector + unchosen_action_update * (1 - choice_vector)
eligibility_traces_reward_updated = reward_eligibility_decay_rate * eligibility_traces_reward + reward * choice_vector
* eligibility_traces_no_reward_updated = no_reward_eligibility_decay_rate * eligibility_traces_no_reward + (1 - reward) * choice_vector
eligibility_traces_reward_updated = reward_eligibility_decay_rate * eligibility_traces_reward + reward * choice_vector
* chosen_action_update = learning_rate * prediction_error
eligibility_traces_no_reward_updated = no_reward_eligibility_decay_rate * eligibility_traces_no_reward + (1 - reward) * choice_vector
* eligibility_traces_no_reward_updated = no_reward_eligibility_decay_rate * eligibility_traces_no_reward + (1 - reward) * choice_vector
* q_value_updates = chosen_action_update * choice_vector + unchosen_action_update * (1 - choice_vector)
* q_values_decayed = q_values + q_decay_rate * (q_decay_baseline - q_values)
eligibility_traces_reward_updated = reward_eligibility_decay_rate * eligibility_traces_reward + reward * choice_vector
* eligibility_traces_no_reward_updated = no_reward_eligibility_decay_rate * eligibility_traces_no_reward + (1 - reward) * choice_vector
* eligibility_traces_no_reward_updated = no_reward_eligibility_decay_rate * eligibility_traces_no_reward + (1 - reward) * choice_vector
* eligibility_traces_no_reward_updated = no_reward_eligibility_decay_rate * eligibility_traces_no_reward + (1 - reward) * choice_vector
unchosen_action_update = -unchosen_learning_rate * prediction_error
* chosen_action_update = learning_rate * prediction_error
* q_values_decayed = q_values + q_decay_rate * (q_decay_baseline - q_values)
* prediction_error = reward - q_value_of_chosen_action
* prediction_error = reward - q_value_of_chosen_action
q_value_updates = chosen_action_update * choice_vector + unchosen_action_update * (1 - choice_vector)
reward_perseveration_influence = perseveration_strength_reward * eligibility_traces_reward_updated
* unchosen_action_update = -unchosen_learning_rate * prediction_error
q_value_influence = inverse_temperature * q_values_updated
choice_logits = q_value_influence + reward_perseveration_influence + no_reward_perseveration_influence
reward_perseveration_influence = perseveration_strength_reward * eligibility_traces_reward_updated
q_value_updates = chosen_action_update * choice_vector + unchosen_action_update * (1 - choice_vector)
q_value_updates = chosen_action_update * choice_vector + unchosen_action_update * (1 - choice_vector)
* choice_logits = q_value_influence + reward_perseveration_influence + no_reward_perseveration_influence
* no_reward_perseveration_influence = perseveration_strength_no_reward * eligibility_traces_no_reward_updated
** no_reward_perseveration_influence = perseveration_strength_no_reward * eligibility_traces_no_reward_updated
* eligibility_traces_no_reward_updated = no_reward_eligibility_decay_rate * eligibility_traces_no_reward + (1 - reward) * choice_vector
* eligibility_traces_no_reward_updated = no_reward_eligibility_decay_rate * eligibility_traces_no_reward + (1 - reward) * choice_vector
*** choice_logits = q_value_influence + reward_perseveration_influence + no_reward_perseveration_influence
eligibility_traces_reward_updated = reward_eligibility_decay_rate * eligibility_traces_reward + reward * choice_vector
* q_values_updated = q_values_decayed + q_value_updates
* q_values_updated = q_values_decayed + q_value_updates
* q_value_influence = inverse_temperature * q_values_updated
* choice_logits = q_value_influence + reward_perseveration_influence + no_reward_perseveration_influence
* q_value_updates = chosen_action_update * choice_vector + unchosen_action_update * (1 - choice_vector)
* eligibility_traces_reward_updated = reward_eligibility_decay_rate * eligibility_traces_reward + reward * choice_vector
q_values_decayed = q_values + q_decay_rate * (q_decay_baseline - q_values)
* q_values_decayed = q_values + q_decay_rate * (q_decay_baseline - q_values)
* q_values_decayed = q_values + q_decay_rate * (q_decay_baseline - q_values)
−0.25 −0.20 −0.15 −0.10 −0.05 0.00
