# Supplementary material for: AI-Discovered Cognitive Models Reveal Novel Insights into Human and Animal Learning
Source: bioRxiv. 2026 May 21:2026.05.18.725921. Preprint. [Version 1] doi: 10.64898/2026.05.18.725921 (PMC13228651; doi:10.64898/2026.05.18.725921)
Supplement: Supplement 2 [file media-2.zip › ablation_performance_monkey_bandit_run1_medium_floor_refactored_20260428.pdf]

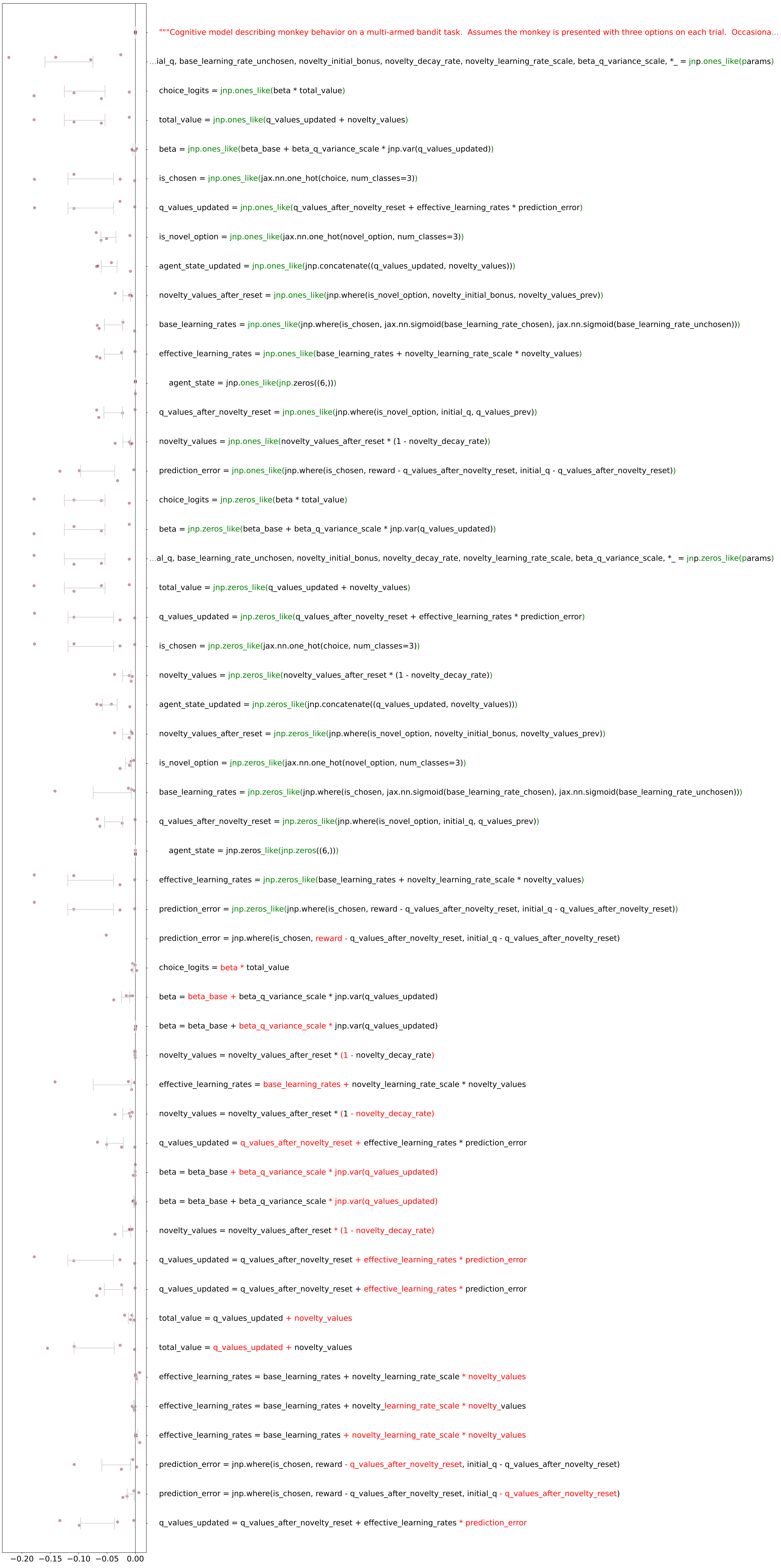
"""Cognitive model describing monkey behavior on a multi-armed bandit task. Assumes the monkey is presented with three options on each trial. Occasiona...
...ial_q, base_learning_rate_unchosen, novelty_initial_bonus, novelty_decay_rate, novelty_learning_rate_scale, beta_q_variance_scale, *_ = jnp.ones_like(params)
choice_logits = jnp.ones_like(beta * total_value)
total_value = jnp.ones_like(q_values_updated + novelty_values)
beta = jnp.ones_like(beta_base + beta_q_variance_scale * jnp.var(q_values_updated))
is_chosen = jnp.ones_like(jax.nn.one_hot(choice, num_classes=3))
q_values_updated = jnp.ones_like(q_values_after_novelty_reset + effective_learning_rates * prediction_error)
is_novel_option = jnp.ones_like(jax.nn.one_hot(novel_option, num_classes=3))
agent_state_updated = jnp.ones_like(jnp.concatenate((q_values_updated, novelty_values)))
novelty_values_after_reset = jnp.ones_like(jnp.where(is_novel_option, novelty_initial_bonus, novelty_values_prev))
base_learning_rates = jnp.ones_like(jnp.where(is_chosen, jax.nn.sigmoid(base_learning_rate_chosen), jax.nn.sigmoid(base_learning_rate_unchosen)))
effective_learning_rates = jnp.ones_like(base_learning_rates + novelty_learning_rate_scale * novelty_values)
agent_state = jnp.ones_like(jnp.zeros((6,)))
q_values_after_novelty_reset = jnp.ones_like(jnp.where(is_novel_option, initial_q, q_values_prev))
novelty_values = jnp.ones_like(novelty_values_after_reset * (1 - novelty_decay_rate))
prediction_error = jnp.ones_like(jnp.where(is_chosen, reward - q_values_after_novelty_reset, initial_q - q_values_after_novelty_reset))
choice_logits = jnp.zeros_like(beta * total_value)
beta = jnp.zeros_like(beta_base + beta_q_variance_scale * jnp.var(q_values_updated))
...al_q, base_learning_rate_unchosen, novelty_initial_bonus, novelty_decay_rate, novelty_learning_rate_scale, beta_q_variance_scale, *_ = jnp.zeros_like(params)
total_value = jnp.zeros_like(q_values_updated + novelty_values)
q_values_updated = jnp.zeros_like(q_values_after_novelty_reset + effective_learning_rates * prediction_error)
is_chosen = jnp.zeros_like(jax.nn.one_hot(choice, num_classes=3))
novelty_values = jnp.zeros_like(novelty_values_after_reset * (1 - novelty_decay_rate))
agent_state_updated = jnp.zeros_like(jnp.concatenate((q_values_updated, novelty_values)))
novelty_values_after_reset = jnp.zeros_like(jnp.where(is_novel_option, novelty_initial_bonus, novelty_values_prev))
is_novel_option = jnp.zeros_like(jax.nn.one_hot(novel_option, num_classes=3))
base_learning_rates = jnp.zeros_like(jnp.where(is_chosen, jax.nn.sigmoid(base_learning_rate_chosen), jax.nn.sigmoid(base_learning_rate_unchosen)))
q_values_after_novelty_reset = jnp.zeros_like(jnp.where(is_novel_option, initial_q, q_values_prev))
agent_state = jnp.zeros_like(jnp.zeros((6,)))
effective_learning_rates = jnp.zeros_like(base_learning_rates + novelty_learning_rate_scale * novelty_values)
prediction_error = jnp.zeros_like(jnp.where(is_chosen, reward - q_values_after_novelty_reset, initial_q - q_values_after_novelty_reset))
prediction_error = jnp.where(is_chosen, reward - q_values_after_novelty_reset, initial_q - q_values_after_novelty_reset)
choice_logits = beta * total_value
beta = beta_base + beta_q_variance_scale * jnp.var(q_values_updated)
beta = beta_base + beta_q_variance_scale * jnp.var(q_values_updated)
novelty_values = novelty_values_after_reset * (1 - novelty_decay_rate)
effective_learning_rates = base_learning_rates + novelty_learning_rate_scale * novelty_values
novelty_values = novelty_values_after_reset * (1 - novelty_decay_rate)
q_values_updated = q_values_after_novelty_reset + effective_learning_rates * prediction_error
beta = beta_base + beta_q_variance_scale * jnp.var(q_values_updated)
beta = beta_base + beta_q_variance_scale * jnp.var(q_values_updated)
novelty_values = novelty_values_after_reset * (1 - novelty_decay_rate)
q_values_updated = q_values_after_novelty_reset + effective_learning_rates * prediction_error
q_values_updated = q_values_after_novelty_reset + effective_learning_rates * prediction_error
total_value = q_values_updated + novelty_values
total_value = q_values_updated + novelty_values
effective_learning_rates = base_learning_rates + novelty_learning_rate_scale * novelty_values
effective_learning_rates = base_learning_rates + novelty_learning_rate_scale * novelty_values
effective_learning_rates = base_learning_rates + novelty_learning_rate_scale * novelty_values
prediction_error = jnp.where(is_chosen, reward - q_values_after_novelty_reset, initial_q - q_values_after_novelty_reset)
prediction_error = jnp.where(is_chosen, reward - q_values_after_novelty_reset, initial_q - q_values_after_novelty_reset)
q_values_updated = q_values_after_novelty_reset + effective_learning_rates * prediction_error
−0.20
−0.15
−0.10
−0.05
0.00
