# Supplementary material for: AI-Discovered Cognitive Models Reveal Novel Insights into Human and Animal Learning
Source: bioRxiv. 2026 May 21:2026.05.18.725921. Preprint. [Version 1] doi: 10.64898/2026.05.18.725921 (PMC13228651; doi:10.64898/2026.05.18.725921)
Supplement: Supplement 2 [file media-2.zip › ablation_performance_fly_bandit_run2_medium_floor_20260420.pdf]

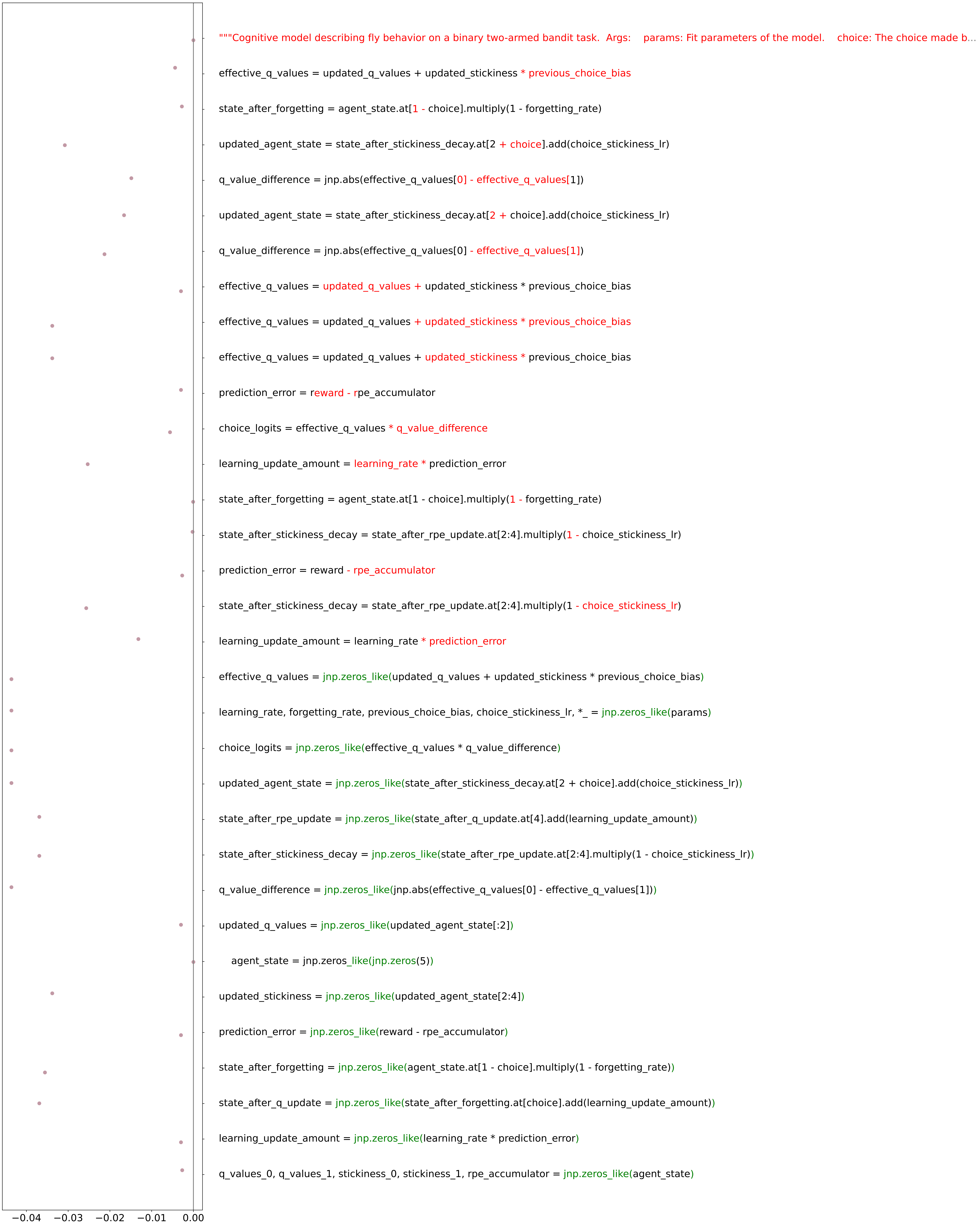
"""Cognitive model describing fly behavior on a binary two-armed bandit task. Args: params: Fit parameters of the model. choice: The choice made b...
effective_q_values = updated_q_values + updated_stickiness * previous_choice_bias
state_after_forgetting = agent_state.at[1 - choice].multiply(1 - forgetting_rate)
updated_agent_state = state_after_stickiness_decay.at[2 + choice].add(choice_stickiness_lr)
q_value_difference = jnp.abs(effective_q_values[0] - effective_q_values[1])
updated_agent_state = state_after_stickiness_decay.at[2 + choice].add(choice_stickiness_lr)
q_value_difference = jnp.abs(effective_q_values[0] - effective_q_values[1])
effective_q_values = updated_q_values + updated_stickiness * previous_choice_bias
effective_q_values = updated_q_values + updated_stickiness * previous_choice_bias
effective_q_values = updated_q_values + updated_stickiness * previous_choice_bias
prediction_error = reward - rpe_accumulator
choice_logits = effective_q_values * q_value_difference
learning_update_amount = learning_rate * prediction_error
state_after_forgetting = agent_state.at[1 - choice].multiply(1 - forgetting_rate)
state_after_stickiness_decay = state_after_rpe_update.at[2:4].multiply(1 - choice_stickiness_lr)
prediction_error = reward - rpe_accumulator
state_after_stickiness_decay = state_after_rpe_update.at[2:4].multiply(1 - choice_stickiness_lr)
learning_update_amount = learning_rate * prediction_error
effective_q_values = jnp.zeros_like(updated_q_values + updated_stickiness * previous_choice_bias)
learning_rate, forgetting_rate, previous_choice_bias, choice_stickiness_lr, *_ = jnp.zeros_like(params)
choice_logits = jnp.zeros_like(effective_q_values * q_value_difference)
updated_agent_state = jnp.zeros_like(state_after_stickiness_decay.at[2 + choice].add(choice_stickiness_lr))
state_after_rpe_update = jnp.zeros_like(state_after_q_update.at[4].add(learning_update_amount))
state_after_stickiness_decay = jnp.zeros_like(state_after_rpe_update.at[2:4].multiply(1 - choice_stickiness_lr))
q_value_difference = jnp.zeros_like(jnp.abs(effective_q_values[0] - effective_q_values[1]))
updated_q_values = jnp.zeros_like(updated_agent_state[:2])
agent_state = jnp.zeros_like(jnp.zeros(5))
updated_stickiness = jnp.zeros_like(updated_agent_state[2:4])
prediction_error = jnp.zeros_like(reward - rpe_accumulator)
state_after_forgetting = jnp.zeros_like(agent_state.at[1 - choice].multiply(1 - forgetting_rate))
state_after_q_update = jnp.zeros_like(state_after_forgetting.at[choice].add(learning_update_amount))
learning_update_amount = jnp.zeros_like(learning_rate * prediction_error)
q_values_0, q_values_1, stickiness_0, stickiness_1, rpe_accumulator = jnp.zeros_like(agent_state)
−0.04
−0.03
−0.02
−0.01
0.00
