# Supplementary material for: AI-Discovered Cognitive Models Reveal Novel Insights into Human and Animal Learning
Source: bioRxiv. 2026 May 21:2026.05.18.725921. Preprint. [Version 1] doi: 10.64898/2026.05.18.725921 (PMC13228651; doi:10.64898/2026.05.18.725921)
Supplement: Supplement 2 [file media-2.zip › ablation_performance_human_bandit_run1_low_floor_refactored_20260420.pdf]

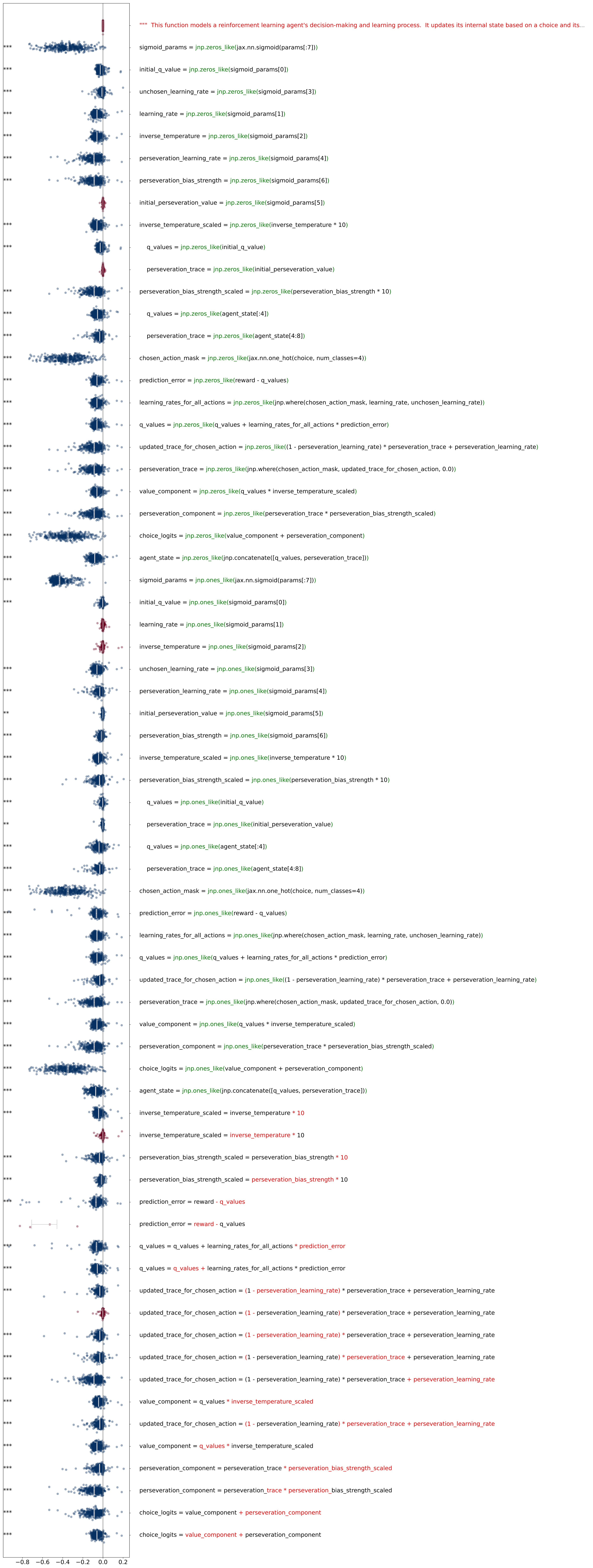

""" This function models a reinforcement learning agent's decision-making and learning process. It updates its internal state based on a choice and its...
*** sigmoid_params = jnp.zeros_like(jax.nn.sigmoid(params[:7]))
*** initial_q_value = jnp.zeros_like(sigmoid_params[0])
*** unchosen_learning_rate = jnp.zeros_like(sigmoid_params[3])
*** learning_rate = jnp.zeros_like(sigmoid_params[1])
*** inverse_temperature = jnp.zeros_like(sigmoid_params[2])
*** perseveration_learning_rate = jnp.zeros_like(sigmoid_params[4])
*** perseveration_bias_strength = jnp.zeros_like(sigmoid_params[6])
initial_perseveration_value = jnp.zeros_like(sigmoid_params[5])
*** inverse_temperature_scaled = jnp.zeros_like(inverse_temperature * 10)
*** q_values = jnp.zeros_like(initial_q_value)
perseveration_trace = jnp.zeros_like(initial_perseveration_value)
*** perseveration_bias_strength_scaled = jnp.zeros_like(perseveration_bias_strength * 10)
*** q_values = jnp.zeros_like(agent_state[:4])
*** perseveration_trace = jnp.zeros_like(agent_state[4:8])
*** chosen_action_mask = jnp.zeros_like(jax.nn.one_hot(choice, num_classes=4))
*** prediction_error = jnp.zeros_like(reward - q_values)
*** learning_rates_for_all_actions = jnp.zeros_like(jnp.where(chosen_action_mask, learning_rate, unchosen_learning_rate))
*** q_values = jnp.zeros_like(q_values + learning_rates_for_all_actions * prediction_error)
*** updated_trace_for_chosen_action = jnp.zeros_like((1 - perseveration_learning_rate) * perseveration_trace + perseveration_learning_rate)
*** perseveration_trace = jnp.zeros_like(jnp.where(chosen_action_mask, updated_trace_for_chosen_action, 0.0))
*** value_component = jnp.zeros_like(q_values * inverse_temperature_scaled)
*** perseveration_component = jnp.zeros_like(perseveration_trace * perseveration_bias_strength_scaled)
*** choice_logits = jnp.zeros_like(value_component + perseveration_component)
*** agent_state = jnp.zeros_like(jnp.concatenate([q_values, perseveration_trace]))
*** sigmoid_params = jnp.ones_like(jax.nn.sigmoid(params[:7]))
*** initial_q_value = jnp.ones_like(sigmoid_params[0])
learning_rate = jnp.ones_like(sigmoid_params[1])
inverse_temperature = jnp.ones_like(sigmoid_params[2])
*** unchosen_learning_rate = jnp.ones_like(sigmoid_params[3])
*** perseveration_learning_rate = jnp.ones_like(sigmoid_params[4])
** initial_perseveration_value = jnp.ones_like(sigmoid_params[5])
*** perseveration_bias_strength = jnp.ones_like(sigmoid_params[6])
*** inverse_temperature_scaled = jnp.ones_like(inverse_temperature * 10)
*** perseveration_bias_strength_scaled = jnp.ones_like(perseveration_bias_strength * 10)
*** q_values = jnp.ones_like(initial_q_value)
** perseveration_trace = jnp.ones_like(initial_perseveration_value)
*** q_values = jnp.ones_like(agent_state[:4])
*** perseveration_trace = jnp.ones_like(agent_state[4:8])
*** chosen_action_mask = jnp.ones_like(jax.nn.one_hot(choice, num_classes=4))
*** prediction_error = jnp.ones_like(reward - q_values)
*** learning_rates_for_all_actions = jnp.ones_like(jnp.where(chosen_action_mask, learning_rate, unchosen_learning_rate))
*** q_values = jnp.ones_like(q_values + learning_rates_for_all_actions * prediction_error)
*** updated_trace_for_chosen_action = jnp.ones_like((1 - perseveration_learning_rate) * perseveration_trace + perseveration_learning_rate)
*** perseveration_trace = jnp.ones_like(jnp.where(chosen_action_mask, updated_trace_for_chosen_action, 0.0))
*** value_component = jnp.ones_like(q_values * inverse_temperature_scaled)
*** perseveration_component = jnp.ones_like(perseveration_trace * perseveration_bias_strength_scaled)
*** choice_logits = jnp.ones_like(value_component + perseveration_component)
*** agent_state = jnp.ones_like(jnp.concatenate([q_values, perseveration_trace]))
*** inverse_temperature_scaled = inverse_temperature * 10
inverse_temperature_scaled = inverse_temperature * 10
*** perseveration_bias_strength_scaled = perseveration_bias_strength * 10
*** perseveration_bias_strength_scaled = perseveration_bias_strength * 10
*** prediction_error = reward - q_values
prediction_error = reward - q_values
*** q_values = q_values + learning_rates_for_all_actions * prediction_error
*** q_values = q_values + learning_rates_for_all_actions * prediction_error
*** updated_trace_for_chosen_action = (1 - perseveration_learning_rate) * perseveration_trace + perseveration_learning_rate
updated_trace_for_chosen_action = (1 - perseveration_learning_rate) * perseveration_trace + perseveration_learning_rate
*** updated_trace_for_chosen_action = (1 - perseveration_learning_rate) * perseveration_trace + perseveration_learning_rate
*** updated_trace_for_chosen_action = (1 - perseveration_learning_rate) * perseveration_trace + perseveration_learning_rate
*** updated_trace_for_chosen_action = (1 - perseveration_learning_rate) * perseveration_trace + perseveration_learning_rate
*** value_component = q_values * inverse_temperature_scaled
*** updated_trace_for_chosen_action = (1 - perseveration_learning_rate) * perseveration_trace + perseveration_learning_rate
*** value_component = q_values * inverse_temperature_scaled
*** perseveration_component = perseveration_trace * perseveration_bias_strength_scaled
*** perseveration_component = perseveration_trace * perseveration_bias_strength_scaled
*** choice_logits = value_component + perseveration_component
*** choice_logits = value_component + perseveration_component
−0.8 −0.6 −0.4 −0.2 0.0 0.2
