# Supplementary material for: AI-Discovered Cognitive Models Reveal Novel Insights into Human and Animal Learning
Source: bioRxiv. 2026 May 21:2026.05.18.725921. Preprint. [Version 1] doi: 10.64898/2026.05.18.725921 (PMC13228651; doi:10.64898/2026.05.18.725921)
Supplement: Supplement 2 [file media-2.zip › ablation_performance_human_bandit_run3_low_floor_refactored_20260420.pdf]

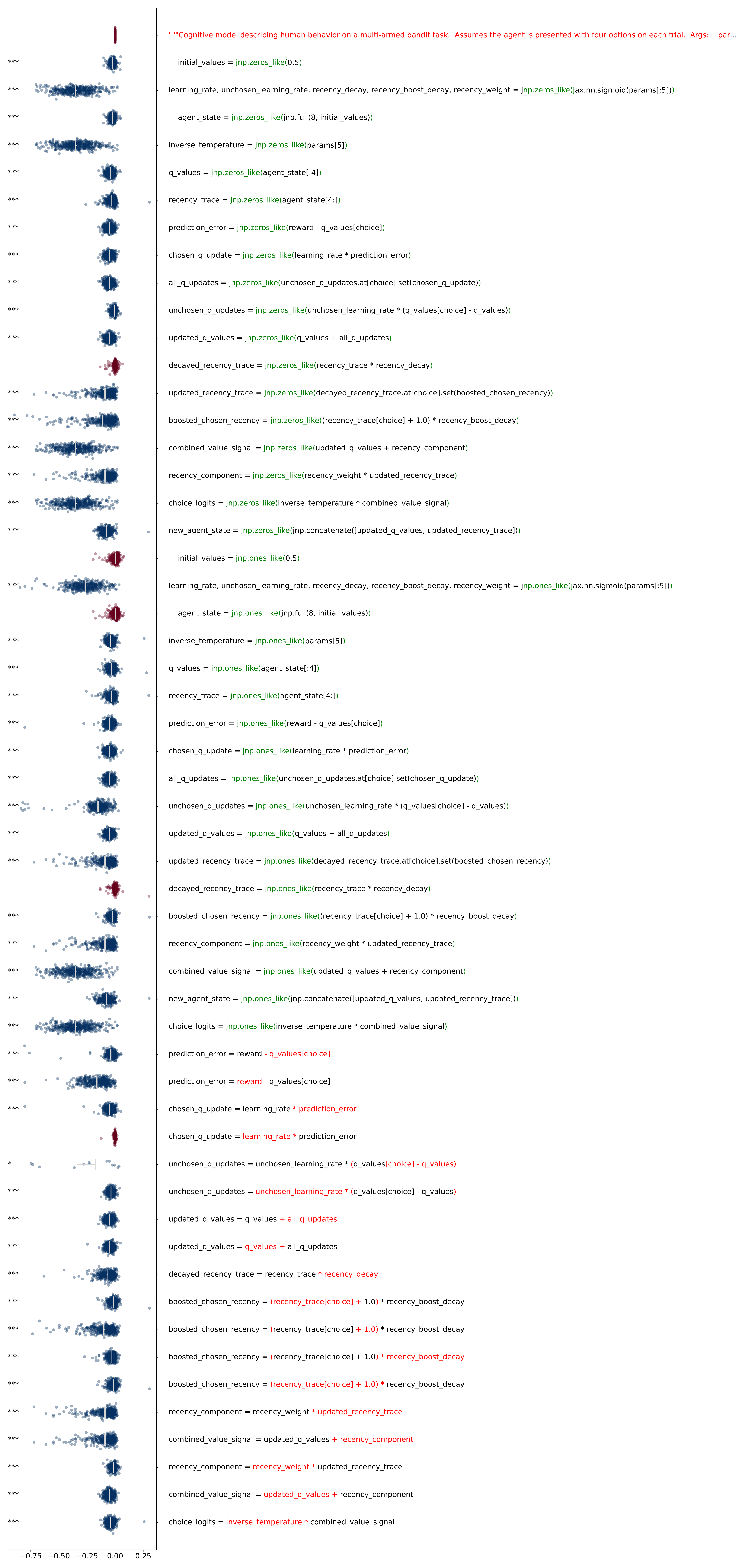
"""Cognitive model describing human behavior on a multi-armed bandit task. Assumes the agent is presented with four options on each trial. Args: par...
initial_values = jnp.zeros_like(0.5)
learning_rate, unchosen_learning_rate, recency_decay, recency_boost_decay, recency_weight = jnp.zeros_like(jax.nn.sigmoid(params[:5]))
agent_state = jnp.zeros_like(jnp.full(8, initial_values))
inverse_temperature = jnp.zeros_like(params[5])
q_values = jnp.zeros_like(agent_state[:4])
recency_trace = jnp.zeros_like(agent_state[4:])
prediction_error = jnp.zeros_like(reward - q_values[choice])
chosen_q_update = jnp.zeros_like(learning_rate * prediction_error)
all_q_updates = jnp.zeros_like(unchosen_q_updates.at[choice].set(chosen_q_update))
unchosen_q_updates = jnp.zeros_like(unchosen_learning_rate * (q_values[choice] - q_values))
updated_q_values = jnp.zeros_like(q_values + all_q_updates)
decayed_recency_trace = jnp.zeros_like(recency_trace * recency_decay)
updated_recency_trace = jnp.zeros_like(decayed_recency_trace.at[choice].set(boosted_chosen_recency))
boosted_chosen_recency = jnp.zeros_like((recency_trace[choice] + 1.0) * recency_boost_decay)
combined_value_signal = jnp.zeros_like(updated_q_values + recency_component)
recency_component = jnp.zeros_like(recency_weight * updated_recency_trace)
choice_logits = jnp.zeros_like(inverse_temperature * combined_value_signal)
new_agent_state = jnp.zeros_like(jnp.concatenate([updated_q_values, updated_recency_trace]))
initial_values = jnp.ones_like(0.5)
learning_rate, unchosen_learning_rate, recency_decay, recency_boost_decay, recency_weight = jnp.ones_like(jax.nn.sigmoid(params[:5]))
agent_state = jnp.ones_like(jnp.full(8, initial_values))
inverse_temperature = jnp.ones_like(params[5])
q_values = jnp.ones_like(agent_state[:4])
recency_trace = jnp.ones_like(agent_state[4:])
prediction_error = jnp.ones_like(reward - q_values[choice])
chosen_q_update = jnp.ones_like(learning_rate * prediction_error)
all_q_updates = jnp.ones_like(unchosen_q_updates.at[choice].set(chosen_q_update))
unchosen_q_updates = jnp.ones_like(unchosen_learning_rate * (q_values[choice] - q_values))
updated_q_values = jnp.ones_like(q_values + all_q_updates)
updated_recency_trace = jnp.ones_like(decayed_recency_trace.at[choice].set(boosted_chosen_recency))
decayed_recency_trace = jnp.ones_like(recency_trace * recency_decay)
boosted_chosen_recency = jnp.ones_like((recency_trace[choice] + 1.0) * recency_boost_decay)
recency_component = jnp.ones_like(recency_weight * updated_recency_trace)
combined_value_signal = jnp.ones_like(updated_q_values + recency_component)
new_agent_state = jnp.ones_like(jnp.concatenate([updated_q_values, updated_recency_trace]))
choice_logits = jnp.ones_like(inverse_temperature * combined_value_signal)
prediction_error = reward - q_values[choice]
prediction_error = reward - q_values[choice]
chosen_q_update = learning_rate * prediction_error
chosen_q_update = learning_rate * prediction_error
unchosen_q_updates = unchosen_learning_rate * (q_values[choice] - q_values)
unchosen_q_updates = unchosen_learning_rate * (q_values[choice] - q_values)
updated_q_values = q_values + all_q_updates
updated_q_values = q_values + all_q_updates
decayed_recency_trace = recency_trace * recency_decay
boosted_chosen_recency = (recency_trace[choice] + 1.0) * recency_boost_decay
boosted_chosen_recency = (recency_trace[choice] + 1.0) * recency_boost_decay
boosted_chosen_recency = (recency_trace[choice] + 1.0) * recency_boost_decay
boosted_chosen_recency = (recency_trace[choice] + 1.0) * recency_boost_decay
recency_component = recency_weight * updated_recency_trace
combined_value_signal = updated_q_values + recency_component
recency_component = recency_weight * updated_recency_trace
combined_value_signal = updated_q_values + recency_component
choice_logits = inverse_temperature * combined_value_signal
−0.75 −0.50 −0.25 0.00 0.25
