# Supplementary material for: AI-Discovered Cognitive Models Reveal Novel Insights into Human and Animal Learning
Source: bioRxiv. 2026 May 21:2026.05.18.725921. Preprint. [Version 1] doi: 10.64898/2026.05.18.725921 (PMC13228651; doi:10.64898/2026.05.18.725921)
Supplement: Supplement 2 [file media-2.zip › ablation_performance_human_bandit_run3_medium_floor_refactored_20260420.pdf]

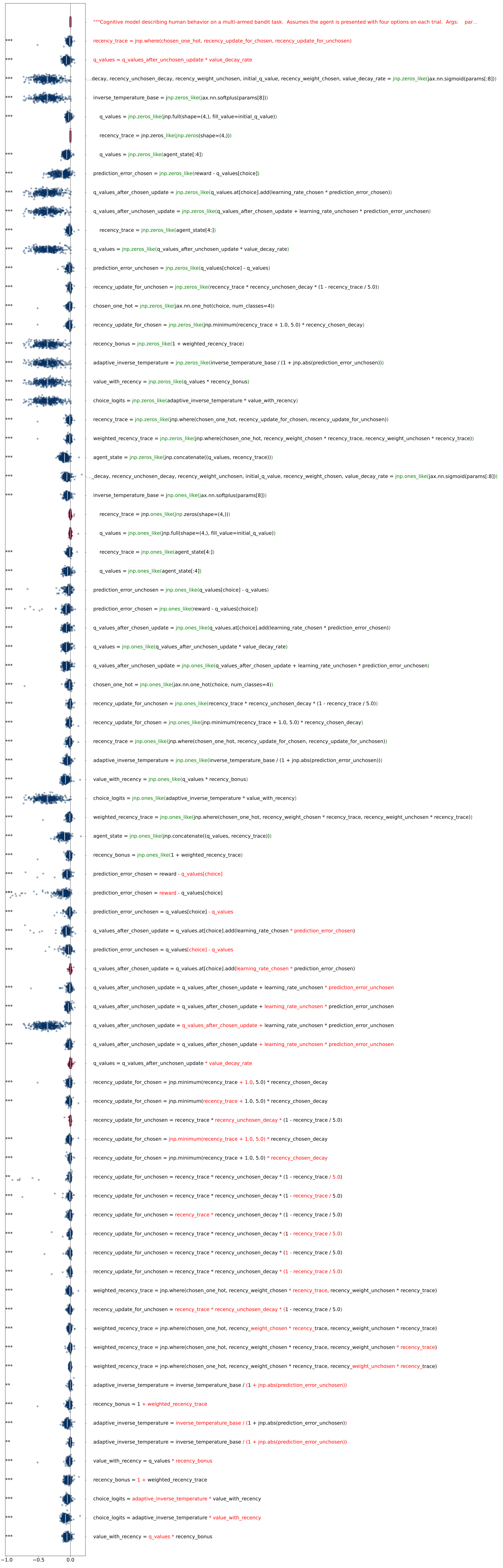

"""Cognitive model describing human behavior on a multi-armed bandit task. Assumes the agent is presented with four options on each trial. Args: par...
*** recency_trace = jnp.where(chosen_one_hot, recency_update_for_chosen, recency_update_for_unchosen)
*** q_values = q_values_after_unchosen_update * value_decay_rate
*** ...decay, recency_unchosen_decay, recency_weight_unchosen, initial_q_value, recency_weight_chosen, value_decay_rate = jnp.zeros_like(jax.nn.sigmoid(params[:8]))
*** inverse_temperature_base = jnp.zeros_like(jax.nn.softplus(params[8]))
*** q_values = jnp.zeros_like(jnp.full(shape=(4,), fill_value=initial_q_value))
recency_trace = jnp.zeros_like(jnp.zeros(shape=(4,)))
*** q_values = jnp.zeros_like(agent_state[:4])
*** prediction_error_chosen = jnp.zeros_like(reward - q_values[choice])
*** q_values_after_chosen_update = jnp.zeros_like(q_values.at[choice].add(learning_rate_chosen * prediction_error_chosen))
*** q_values_after_unchosen_update = jnp.zeros_like(q_values_after_chosen_update + learning_rate_unchosen * prediction_error_unchosen)
*** recency_trace = jnp.zeros_like(agent_state[4:])
*** q_values = jnp.zeros_like(q_values_after_unchosen_update * value_decay_rate)
*** prediction_error_unchosen = jnp.zeros_like(q_values[choice] - q_values)
*** recency_update_for_unchosen = jnp.zeros_like(recency_trace * recency_unchosen_decay * (1 - recency_trace / 5.0))
*** chosen_one_hot = jnp.zeros_like(jax.nn.one_hot(choice, num_classes=4))
*** recency_update_for_chosen = jnp.zeros_like(jnp.minimum(recency_trace + 1.0, 5.0) * recency_chosen_decay)
*** recency_bonus = jnp.zeros_like(1 + weighted_recency_trace)
*** adaptive_inverse_temperature = jnp.zeros_like(inverse_temperature_base / (1 + jnp.abs(prediction_error_unchosen)))
*** value_with_recency = jnp.zeros_like(q_values * recency_bonus)
*** choice_logits = jnp.zeros_like(adaptive_inverse_temperature * value_with_recency)
*** recency_trace = jnp.zeros_like(jnp.where(chosen_one_hot, recency_update_for_chosen, recency_update_for_unchosen))
*** weighted_recency_trace = jnp.zeros_like(jnp.where(chosen_one_hot, recency_weight_chosen * recency_trace, recency_weight_unchosen * recency_trace))
*** agent_state = jnp.zeros_like(jnp.concatenate((q_values, recency_trace)))
*** ..._decay, recency_unchosen_decay, recency_weight_unchosen, initial_q_value, recency_weight_chosen, value_decay_rate = jnp.ones_like(jax.nn.sigmoid(params[:8]))
*** inverse_temperature_base = jnp.ones_like(jax.nn.softplus(params[8]))
recency_trace = jnp.ones_like(jnp.zeros(shape=(4,)))
q_values = jnp.ones_like(jnp.full(shape=(4,), fill_value=initial_q_value))
*** recency_trace = jnp.ones_like(agent_state[4:])
*** q_values = jnp.ones_like(agent_state[:4])
*** prediction_error_unchosen = jnp.ones_like(q_values[choice] - q_values)
*** prediction_error_chosen = jnp.ones_like(reward - q_values[choice])
*** q_values_after_chosen_update = jnp.ones_like(q_values.at[choice].add(learning_rate_chosen * prediction_error_chosen))
*** q_values = jnp.ones_like(q_values_after_unchosen_update * value_decay_rate)
*** q_values_after_unchosen_update = jnp.ones_like(q_values_after_chosen_update + learning_rate_unchosen * prediction_error_unchosen)
*** chosen_one_hot = jnp.ones_like(jax.nn.one_hot(choice, num_classes=4))
*** recency_update_for_unchosen = jnp.ones_like(recency_trace * recency_unchosen_decay * (1 - recency_trace / 5.0))
*** recency_update_for_chosen = jnp.ones_like(jnp.minimum(recency_trace + 1.0, 5.0) * recency_chosen_decay)
*** recency_trace = jnp.ones_like(jnp.where(chosen_one_hot, recency_update_for_chosen, recency_update_for_unchosen))
*** adaptive_inverse_temperature = jnp.ones_like(inverse_temperature_base / (1 + jnp.abs(prediction_error_unchosen)))
*** value_with_recency = jnp.ones_like(q_values * recency_bonus)
*** choice_logits = jnp.ones_like(adaptive_inverse_temperature * value_with_recency)
*** weighted_recency_trace = jnp.ones_like(jnp.where(chosen_one_hot, recency_weight_chosen * recency_trace, recency_weight_unchosen * recency_trace))
*** agent_state = jnp.ones_like(jnp.concatenate((q_values, recency_trace)))
*** recency_bonus = jnp.ones_like(1 + weighted_recency_trace)
*** prediction_error_chosen = reward - q_values[choice]
*** prediction_error_chosen = reward - q_values[choice]
*** prediction_error_unchosen = q_values[choice] - q_values
*** q_values_after_chosen_update = q_values.at[choice].add(learning_rate_chosen * prediction_error_chosen)
*** prediction_error_unchosen = q_values[choice] - q_values
q_values_after_chosen_update = q_values.at[choice].add(learning_rate_chosen * prediction_error_chosen)
*** q_values_after_unchosen_update = q_values_after_chosen_update + learning_rate_unchosen * prediction_error_unchosen
*** q_values_after_unchosen_update = q_values_after_chosen_update + learning_rate_unchosen * prediction_error_unchosen
*** q_values_after_unchosen_update = q_values_after_chosen_update + learning_rate_unchosen * prediction_error_unchosen
*** q_values_after_unchosen_update = q_values_after_chosen_update + learning_rate_unchosen * prediction_error_unchosen
q_values = q_values_after_unchosen_update * value_decay_rate
*** recency_update_for_chosen = jnp.minimum(recency_trace + 1.0, 5.0) * recency_chosen_decay
*** recency_update_for_chosen = jnp.minimum(recency_trace + 1.0, 5.0) * recency_chosen_decay
recency_update_for_unchosen = recency_trace * recency_unchosen_decay * (1 - recency_trace / 5.0)
*** recency_update_for_chosen = jnp.minimum(recency_trace + 1.0, 5.0) * recency_chosen_decay
*** recency_update_for_chosen = jnp.minimum(recency_trace + 1.0, 5.0) * recency_chosen_decay
** recency_update_for_unchosen = recency_trace * recency_unchosen_decay * (1 - recency_trace / 5.0)
*** recency_update_for_unchosen = recency_trace * recency_unchosen_decay * (1 - recency_trace / 5.0)
*** recency_update_for_unchosen = recency_trace * recency_unchosen_decay * (1 - recency_trace / 5.0)
*** recency_update_for_unchosen = recency_trace * recency_unchosen_decay * (1 - recency_trace / 5.0)
*** recency_update_for_unchosen = recency_trace * recency_unchosen_decay * (1 - recency_trace / 5.0)
*** recency_update_for_unchosen = recency_trace * recency_unchosen_decay * (1 - recency_trace / 5.0)
*** weighted_recency_trace = jnp.where(chosen_one_hot, recency_weight_chosen * recency_trace, recency_weight_unchosen * recency_trace)
*** recency_update_for_unchosen = recency_trace * recency_unchosen_decay * (1 - recency_trace / 5.0)
*** weighted_recency_trace = jnp.where(chosen_one_hot, recency_weight_chosen * recency_trace, recency_weight_unchosen * recency_trace)
*** weighted_recency_trace = jnp.where(chosen_one_hot, recency_weight_chosen * recency_trace, recency_weight_unchosen * recency_trace)
*** weighted_recency_trace = jnp.where(chosen_one_hot, recency_weight_chosen * recency_trace, recency_weight_unchosen * recency_trace)
** adaptive_inverse_temperature = inverse_temperature_base / (1 + jnp.abs(prediction_error_unchosen))
*** recency_bonus = 1 + weighted_recency_trace
*** adaptive_inverse_temperature = inverse_temperature_base / (1 + jnp.abs(prediction_error_unchosen))
** adaptive_inverse_temperature = inverse_temperature_base / (1 + jnp.abs(prediction_error_unchosen))
*** value_with_recency = q_values * recency_bonus
*** recency_bonus = 1 + weighted_recency_trace
*** choice_logits = adaptive_inverse_temperature * value_with_recency
*** choice_logits = adaptive_inverse_temperature * value_with_recency
*** value_with_recency = q_values * recency_bonus
−1.0
−0.5
0.0
