# Supplementary figures and images for: AI-Discovered Cognitive Models Reveal Novel Insights into Human and Animal Learning
Source: bioRxiv. 2026 May 21:2026.05.18.725921. Preprint. [Version 1] doi: 10.64898/2026.05.18.725921 (PMC13228651; doi:10.64898/2026.05.18.725921)

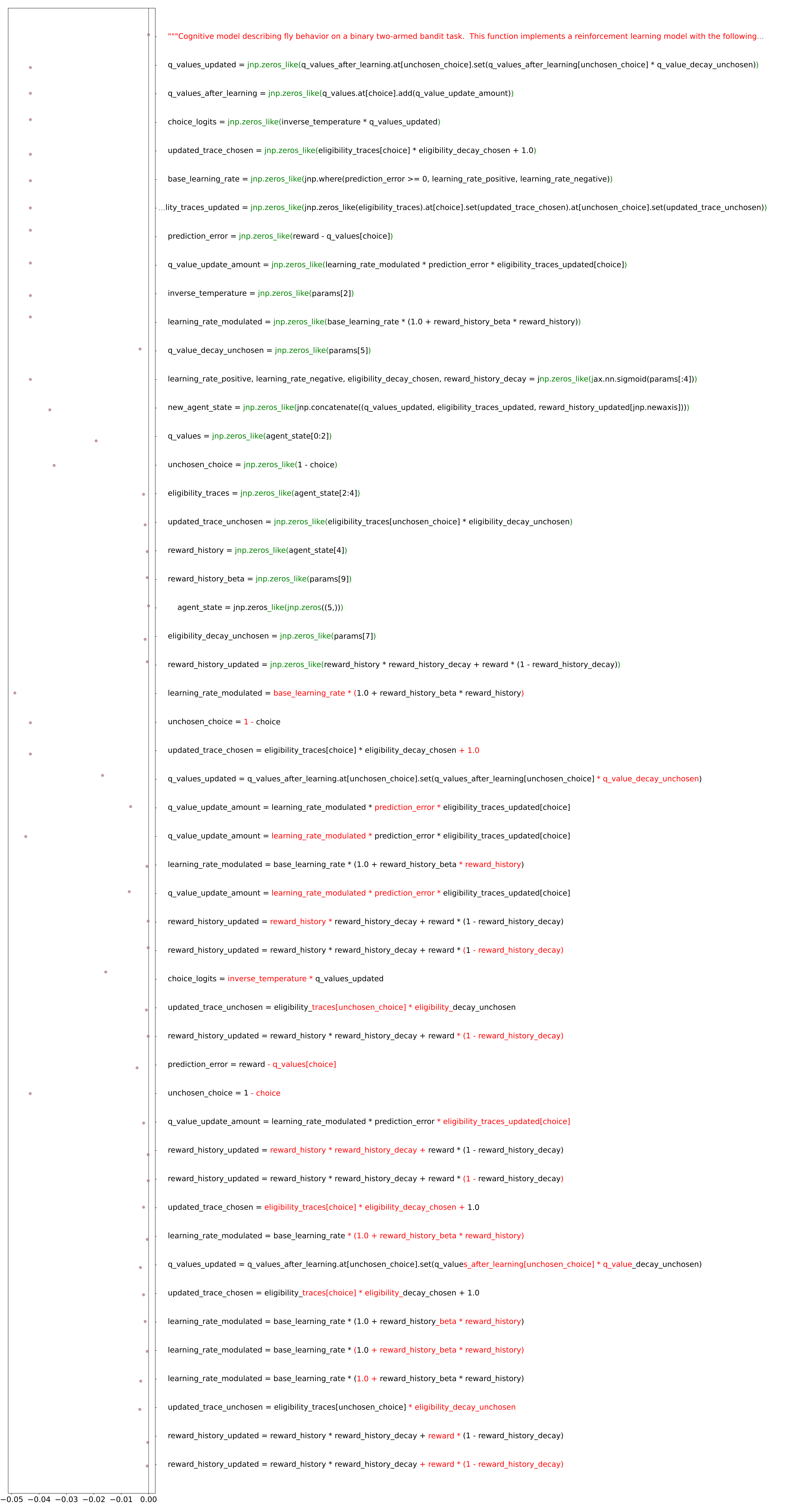

Supplement: Supplement 2 [file media-2.zip › ablation_performance_fly_bandit_run1_medium_floor_20260420.pdf]

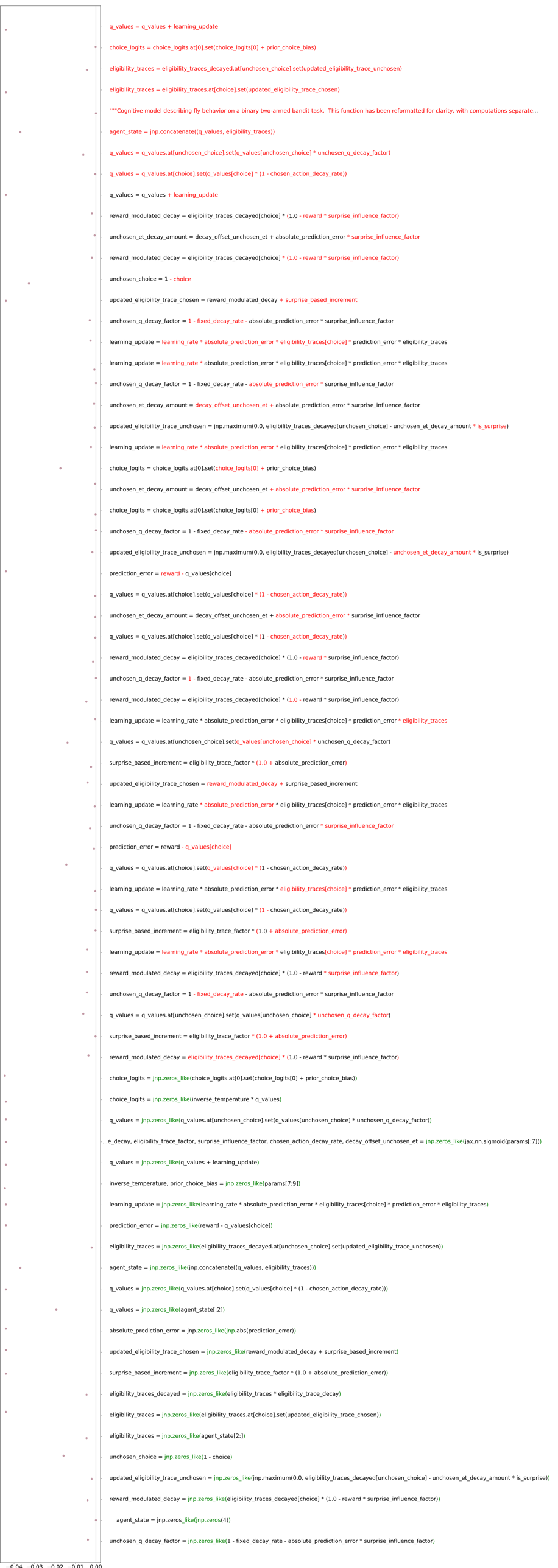

Supplement: Supplement 2 [file media-2.zip › ablation_performance_fly_bandit_run3_high_floor_20260420.pdf]

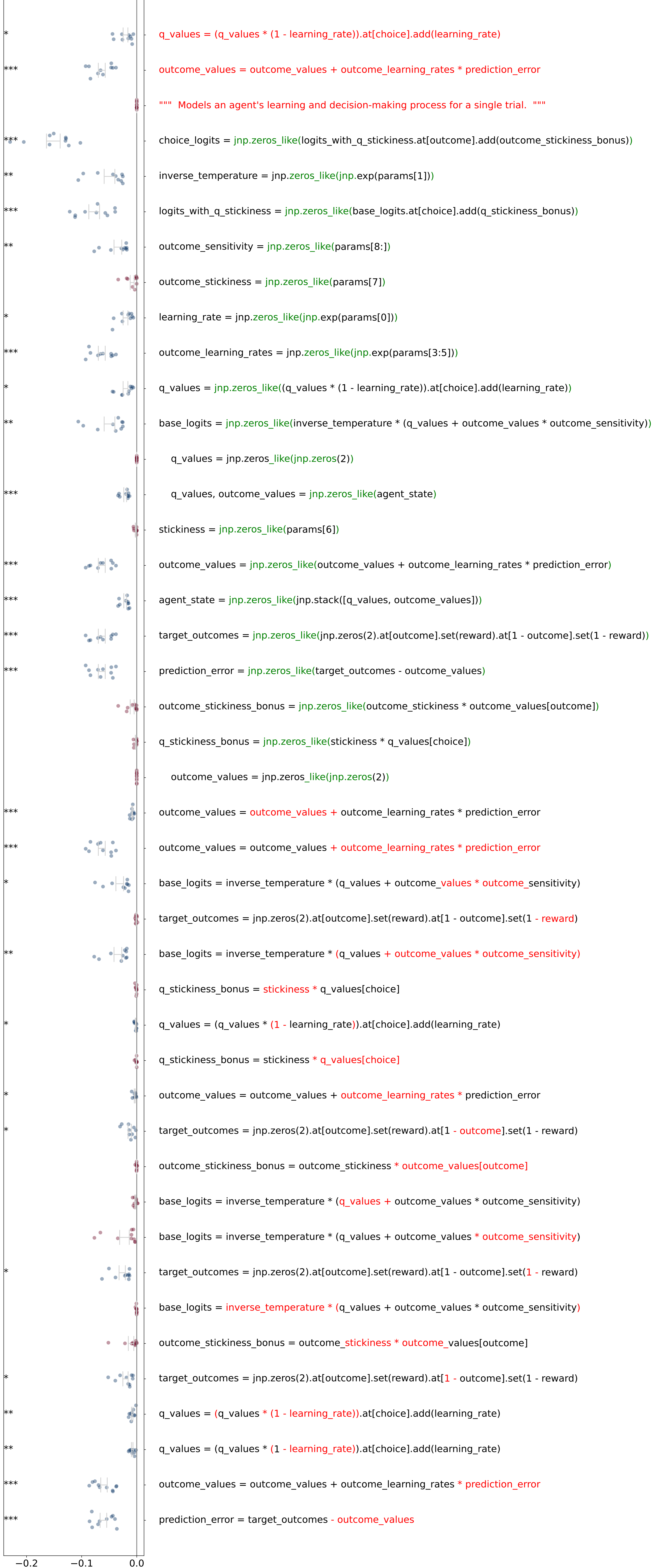

Supplement: Supplement 2 [file media-2.zip › ablation_performance_rat_twostep_run2_medium_floor_20260420.pdf]

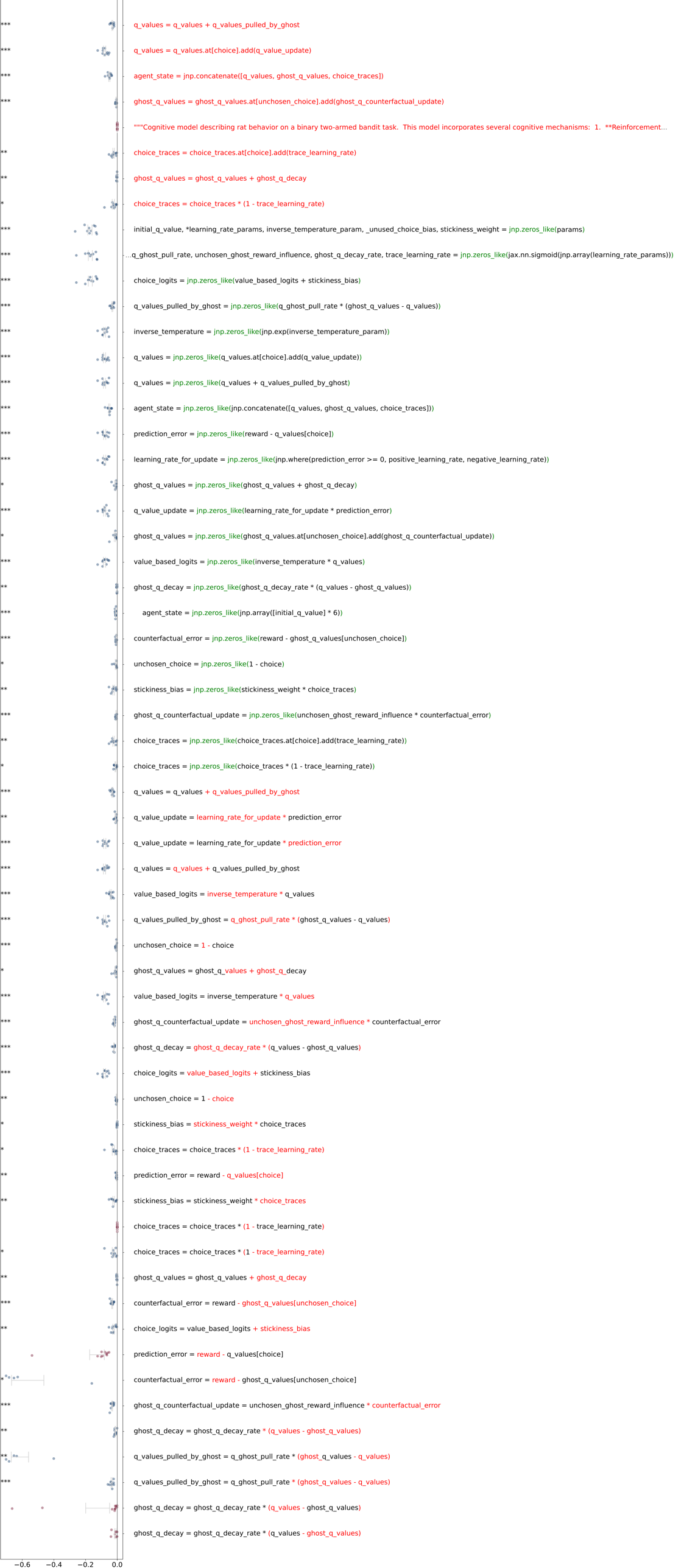

Supplement: Supplement 2 [file media-2.zip › ablation_performance_rat_bandit_run2_low_floor_20260420.pdf]

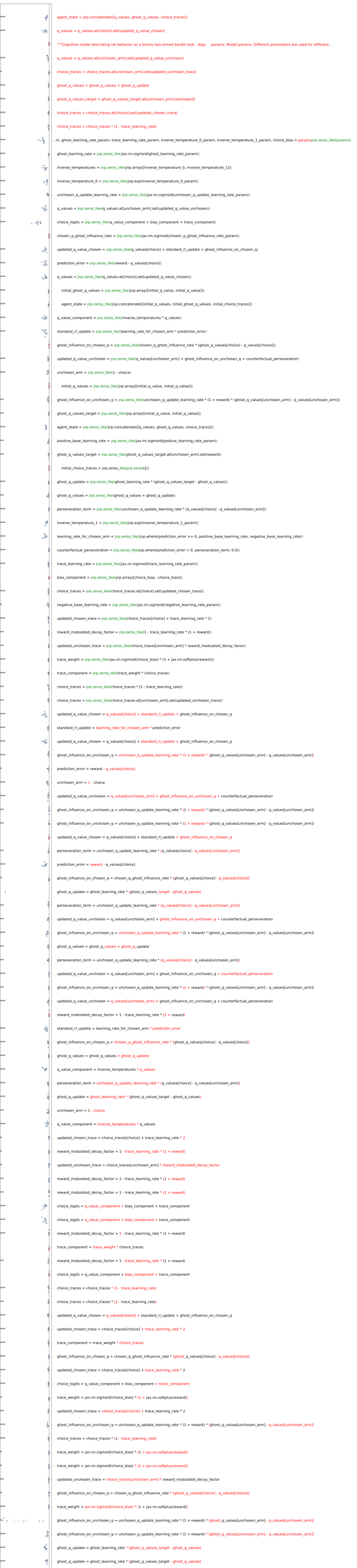

Supplement: Supplement 2 [file media-2.zip › ablation_performance_rat_bandit_run2_high_floor_20260420.pdf]

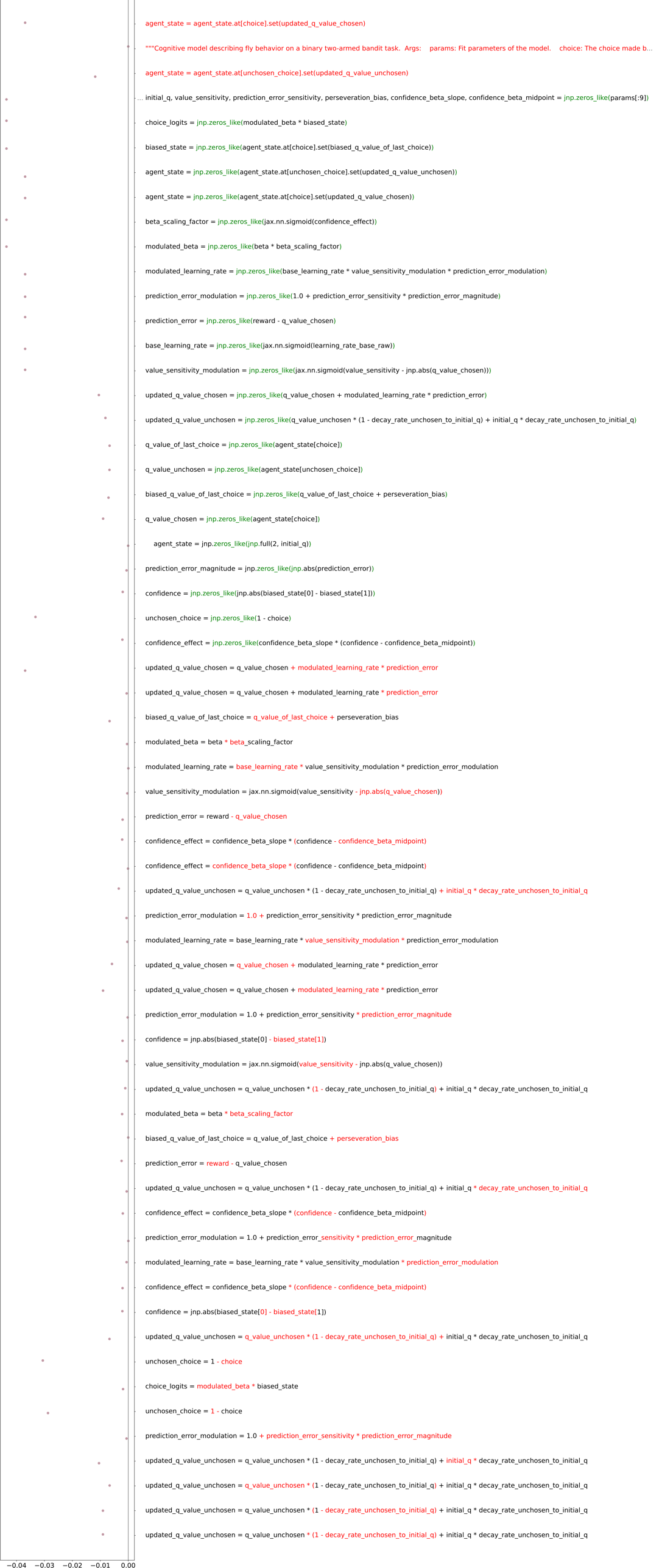

Supplement: Supplement 2 [file media-2.zip › ablation_performance_fly_bandit_run3_medium_floor_20260420.pdf]

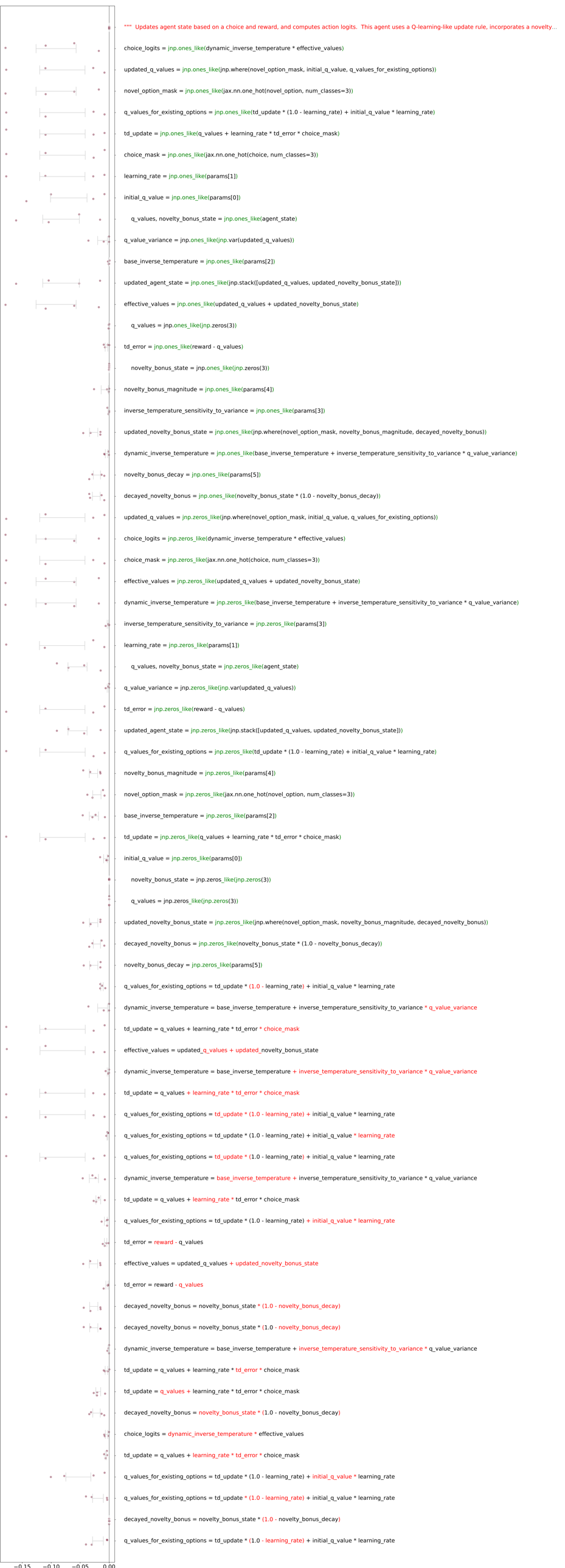

Supplement: Supplement 2 [file media-2.zip › ablation_performance_monkey_bandit_run3_low_floor_refactored_20260420.pdf]

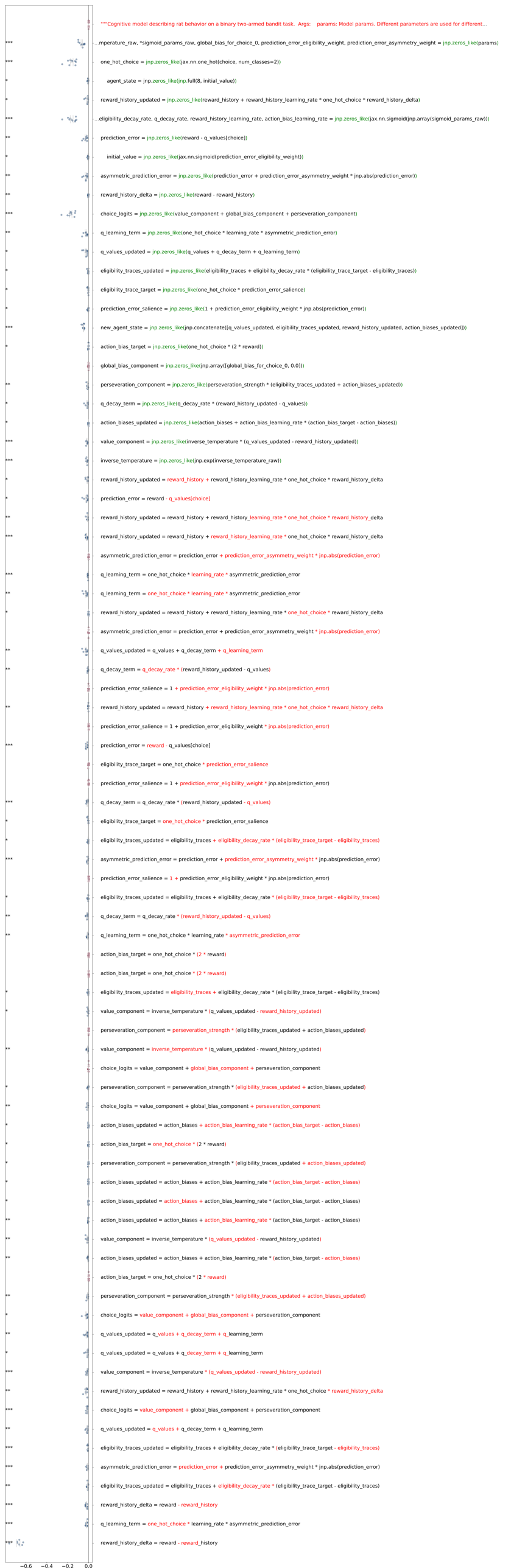

Supplement: Supplement 2 [file media-2.zip › ablation_performance_rat_bandit_run3_medium_floor_20260420.pdf]

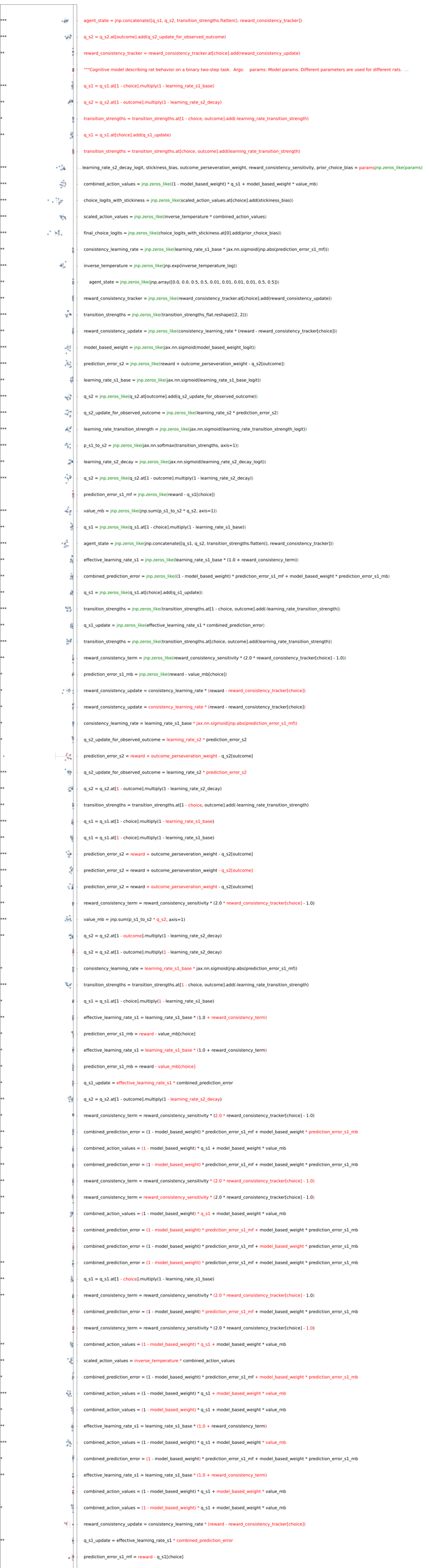

Supplement: Supplement 2 [file media-2.zip › ablation_performance_rat_twostep_run1_high_floor_20260420.pdf]

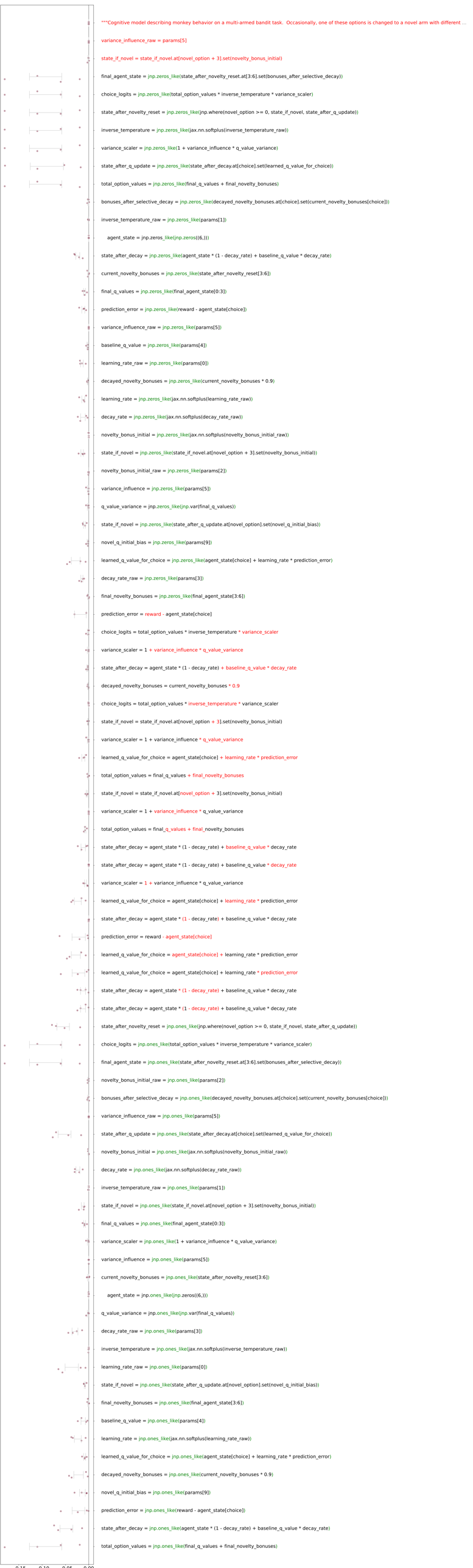

Supplement: Supplement 2 [file media-2.zip › ablation_performance_monkey_bandit_run2_medium_floor_refactored_20260420.pdf]

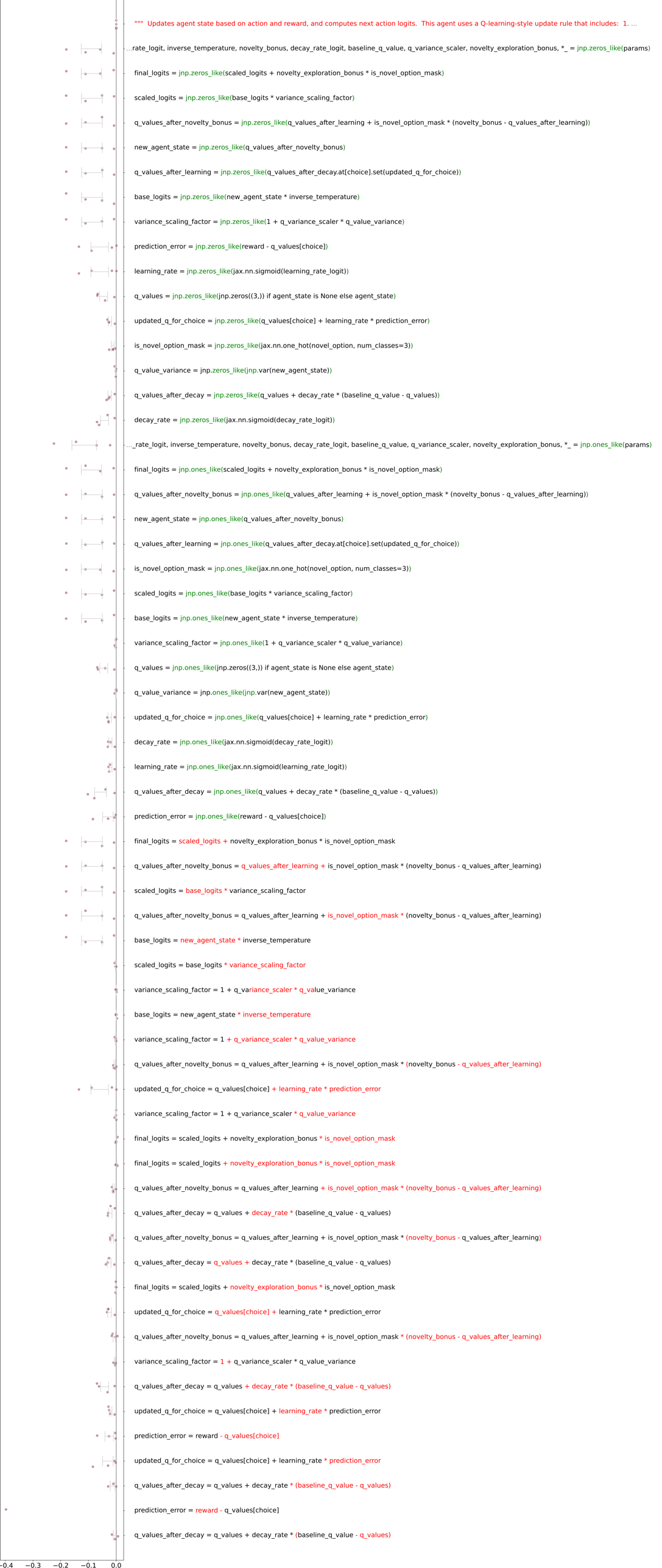

Supplement: Supplement 2 [file media-2.zip › ablation_performance_monkey_bandit_run2_low_floor_refactored_20260428.pdf]

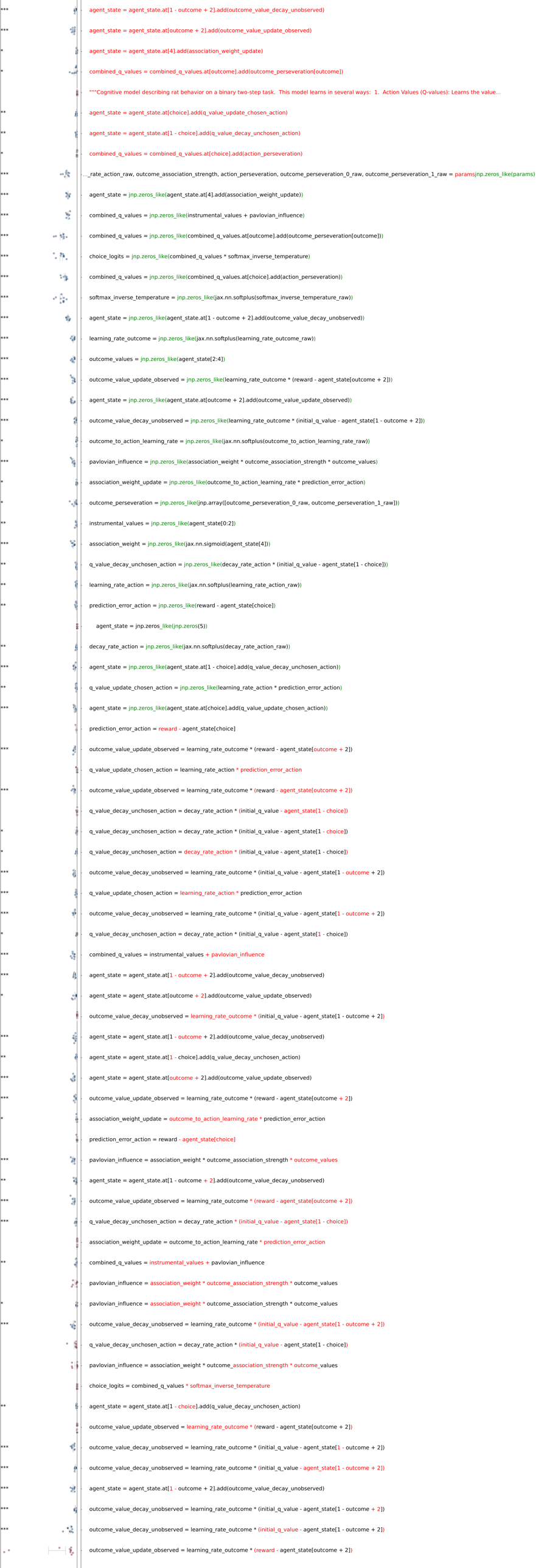

Supplement: Supplement 2 [file media-2.zip › ablation_performance_rat_twostep_run1_medium_floor_20260420.pdf]

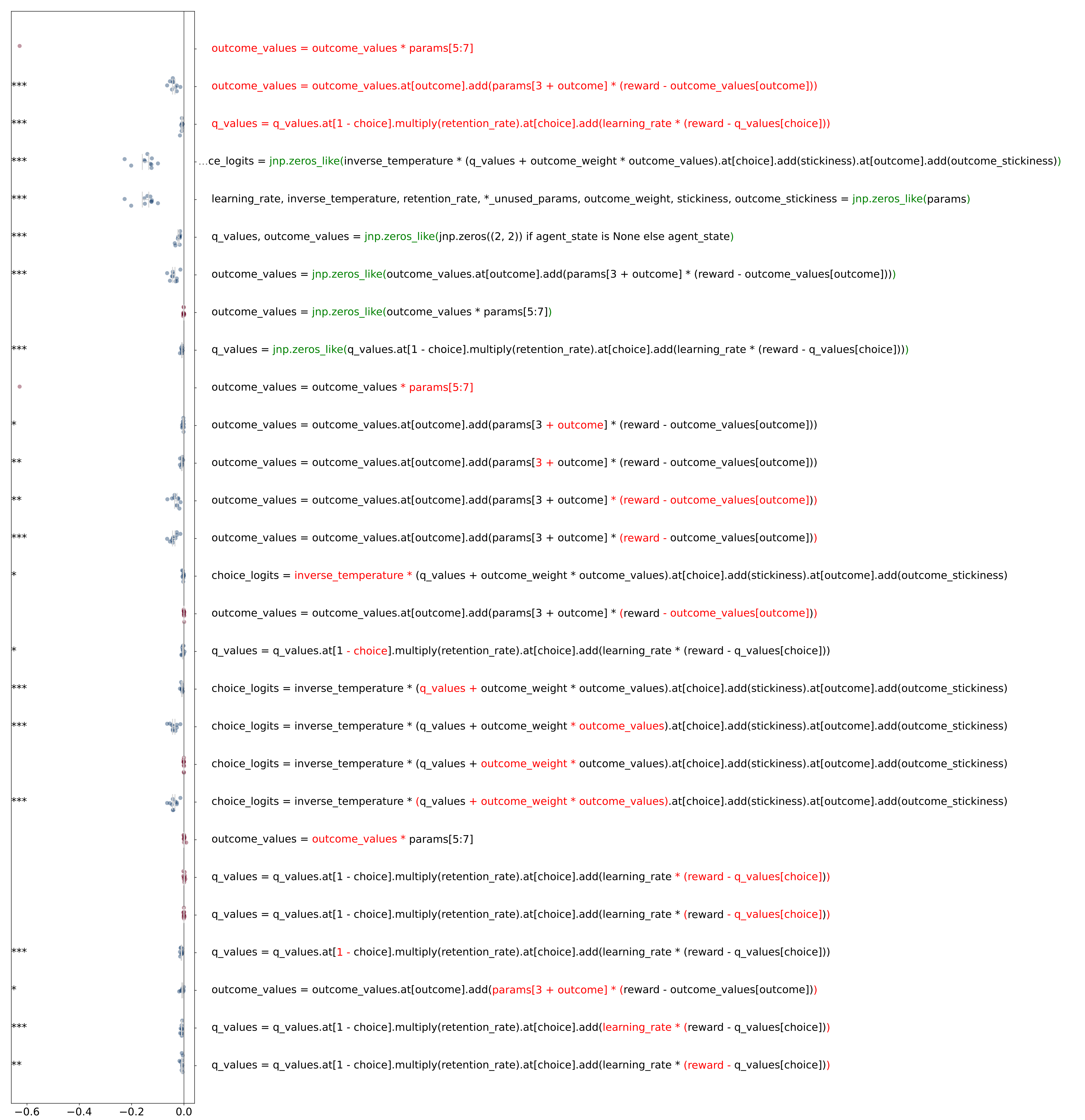

Supplement: Supplement 2 [file media-2.zip › ablation_performance_rat_twostep_run2_low_floor_shortenonly_20260420.pdf]

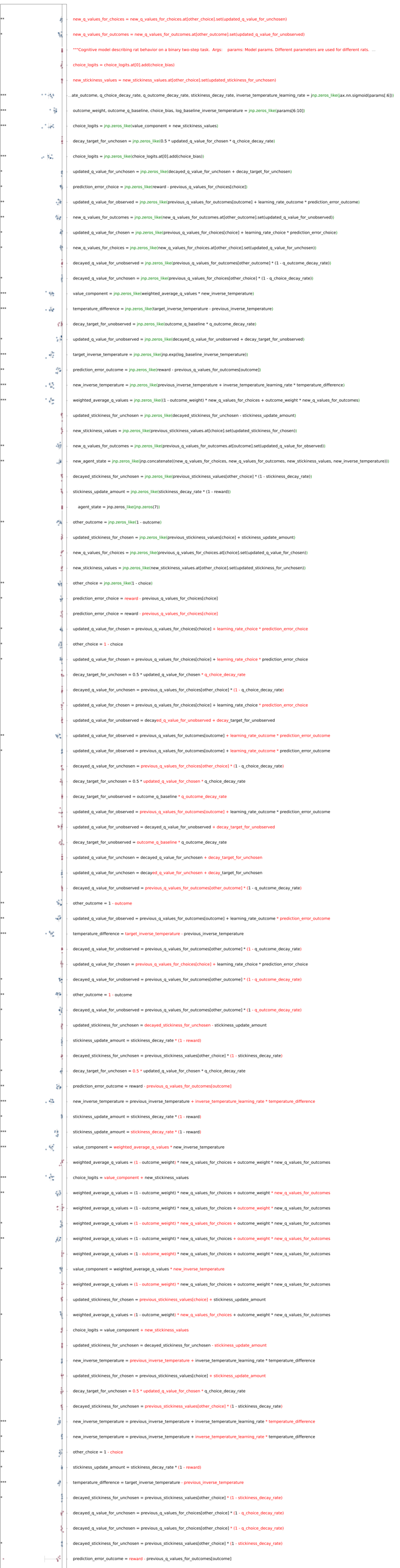

Supplement: Supplement 2 [file media-2.zip › ablation_performance_rat_twostep_run3_high_floor_20260420.pdf]

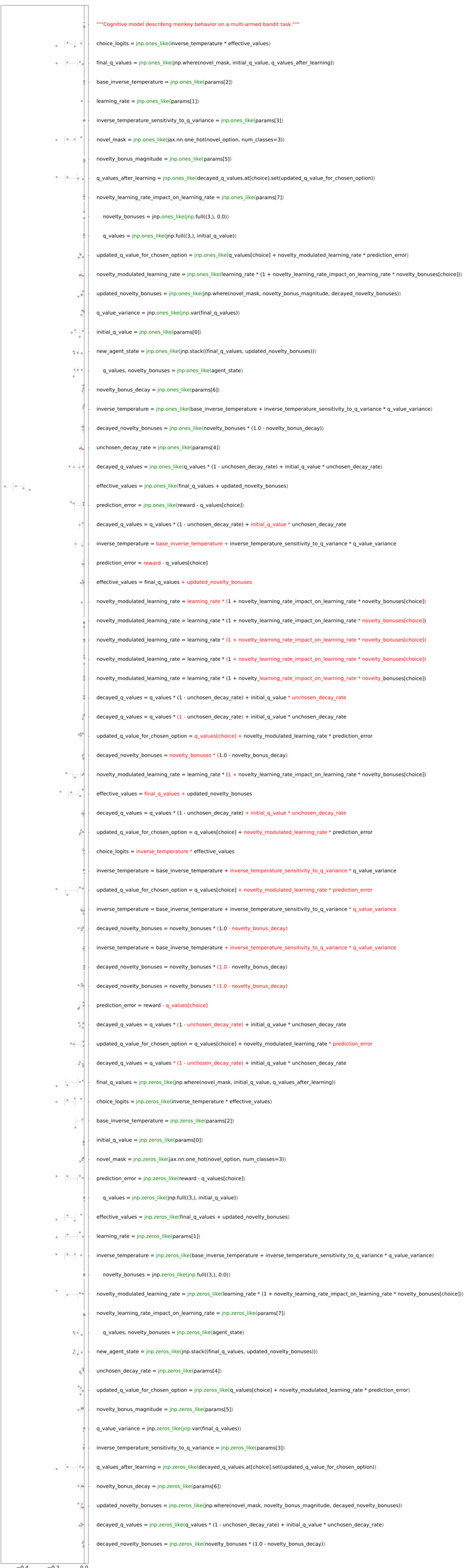

Supplement: Supplement 2 [file media-2.zip › ablation_performance_monkey_bandit_run3_medium_floor_refactored_20260420.pdf]

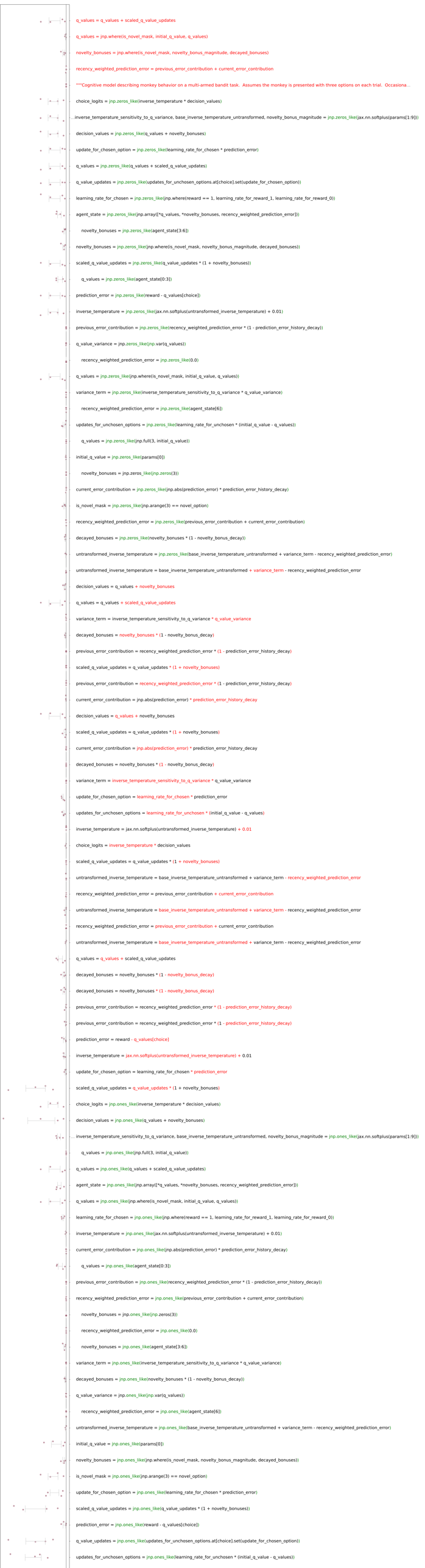

Supplement: Supplement 2 [file media-2.zip › ablation_performance_monkey_bandit_run3_high_floor_refactored_20260420.pdf]

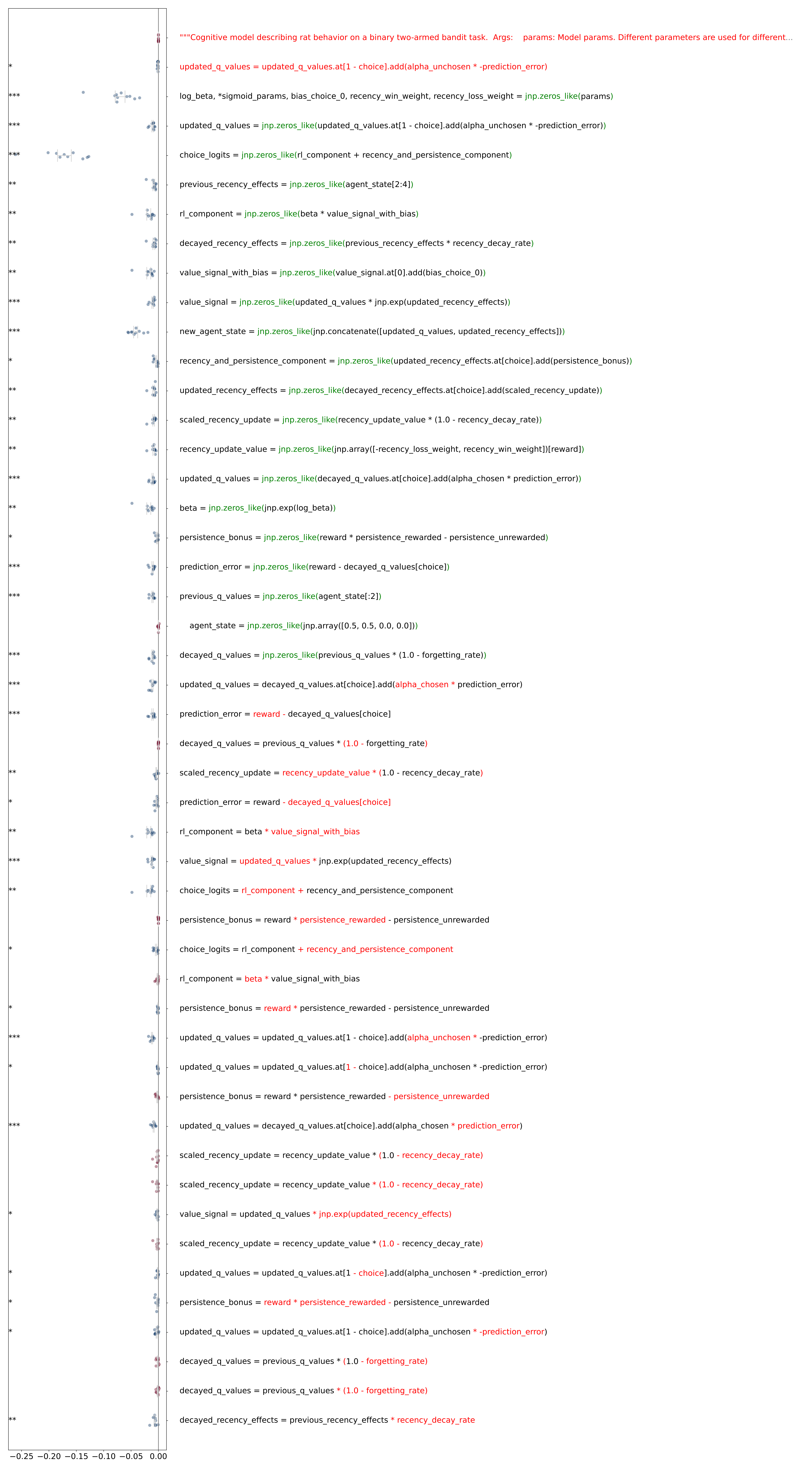

Supplement: Supplement 2 [file media-2.zip › ablation_performance_rat_bandit_run1_medium_floor_20260420.pdf]

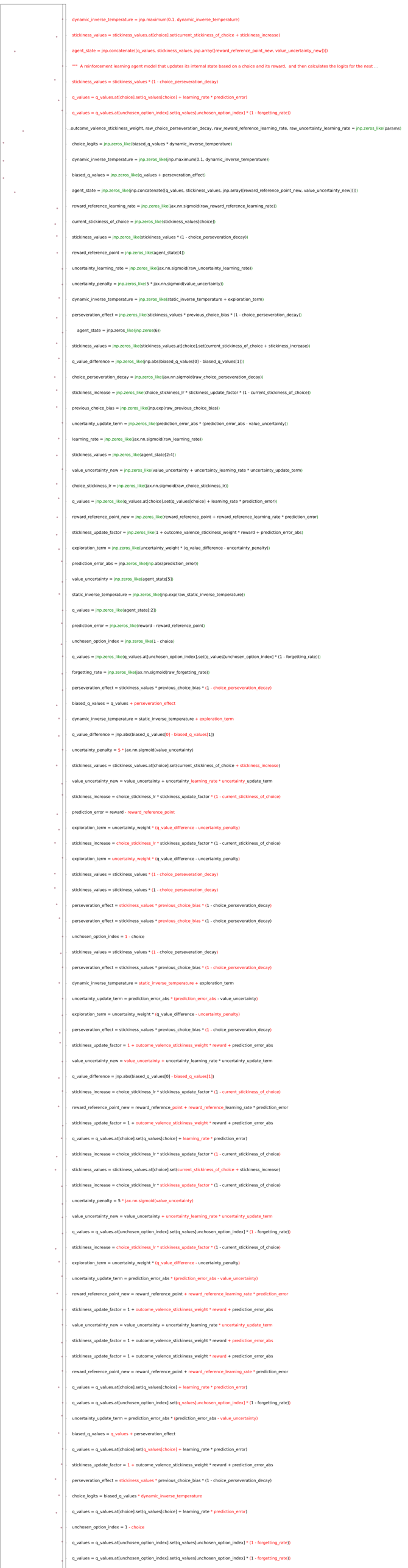

Supplement: Supplement 2 [file media-2.zip › ablation_performance_fly_bandit_run2_high_floor_20260420.pdf]

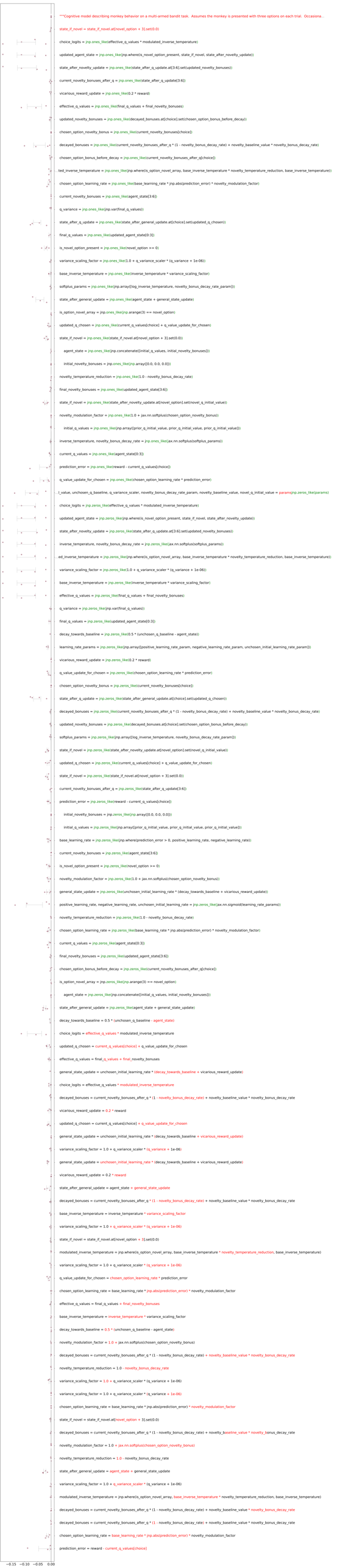

Supplement: Supplement 2 [file media-2.zip › ablation_performance_monkey_bandit_run2_high_floor_refactored_20260420.pdf]

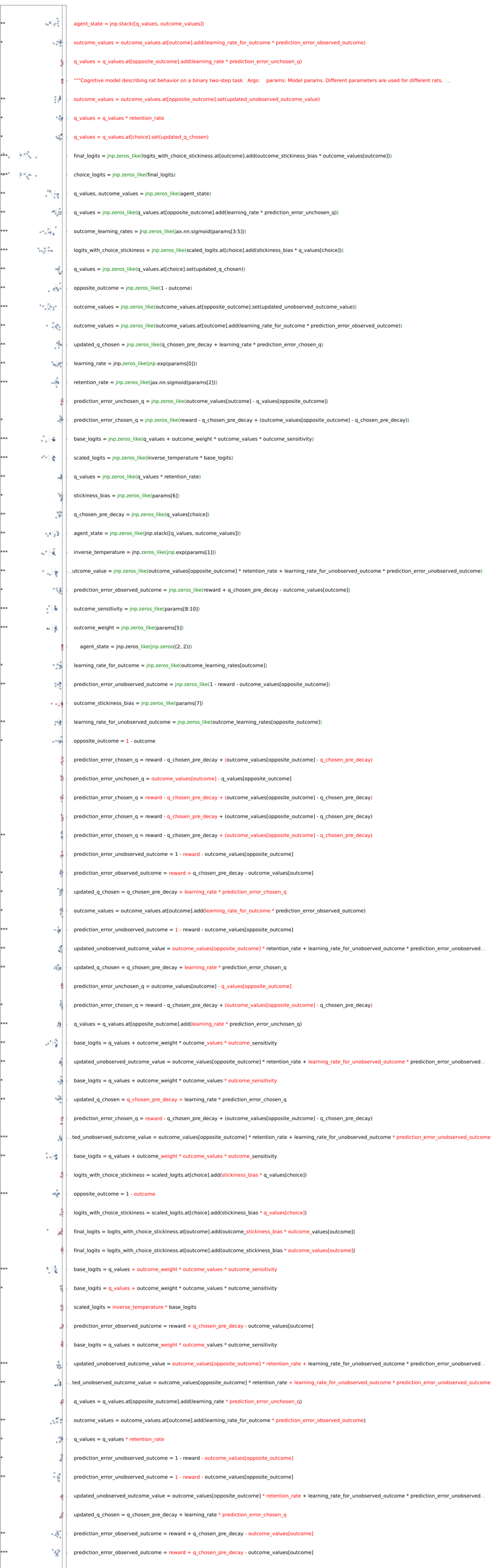

Supplement: Supplement 2 [file media-2.zip › ablation_performance_rat_twostep_run2_high_floor_20260420.pdf]

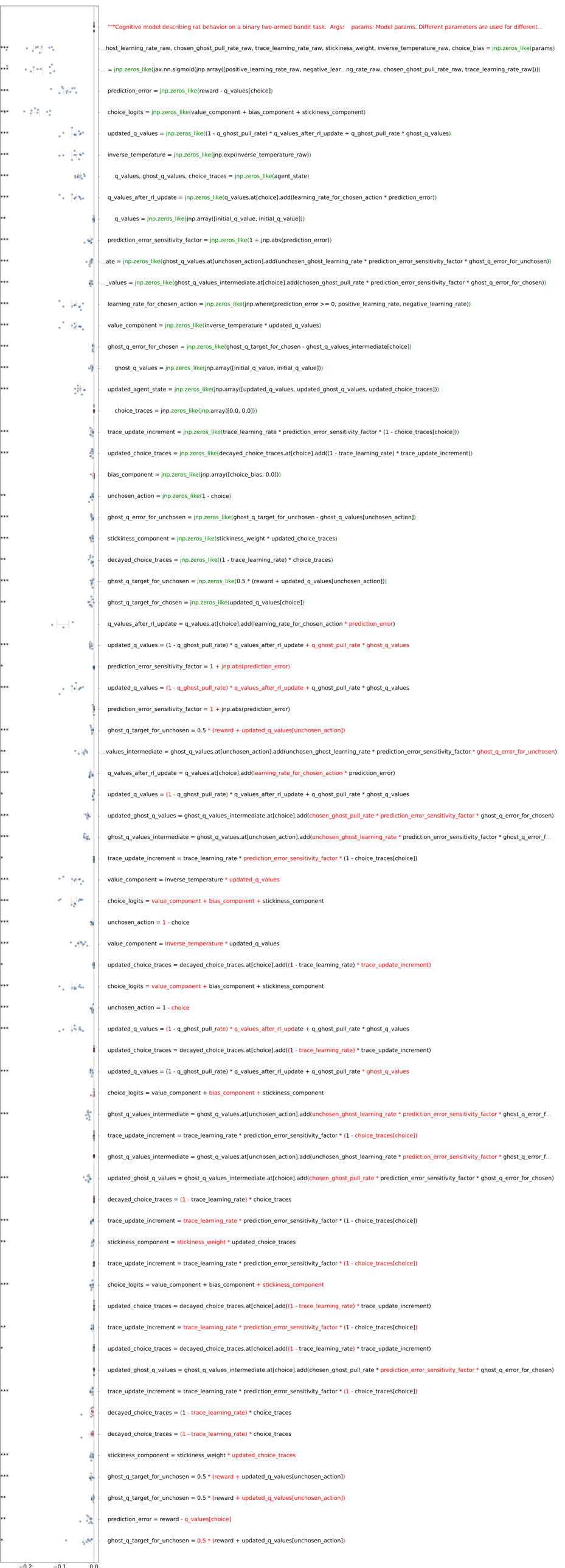

Supplement: Supplement 2 [file media-2.zip › ablation_performance_rat_bandit_run2_medium_floor_20260420.pdf]

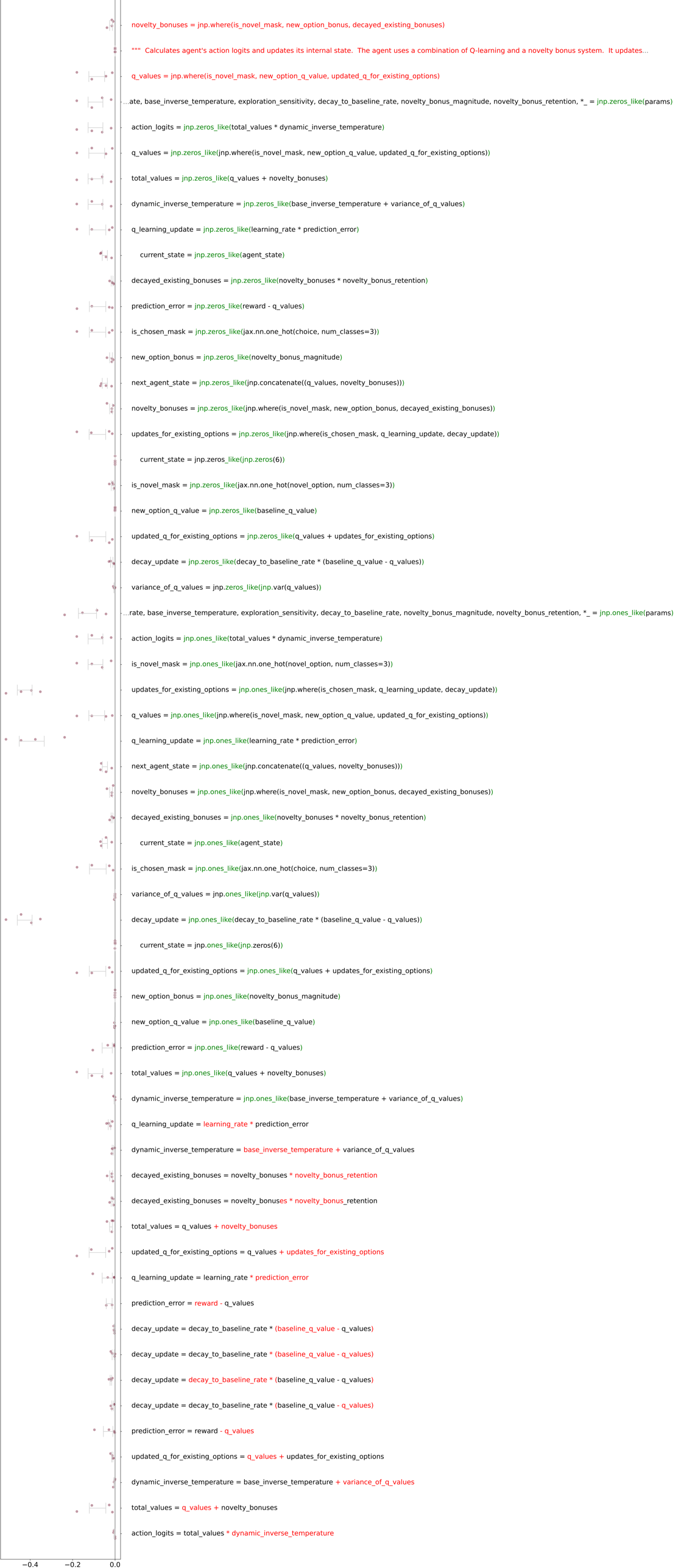

Supplement: Supplement 2 [file media-2.zip › ablation_performance_monkey_bandit_run1_low_floor_refactored_20260428.pdf]

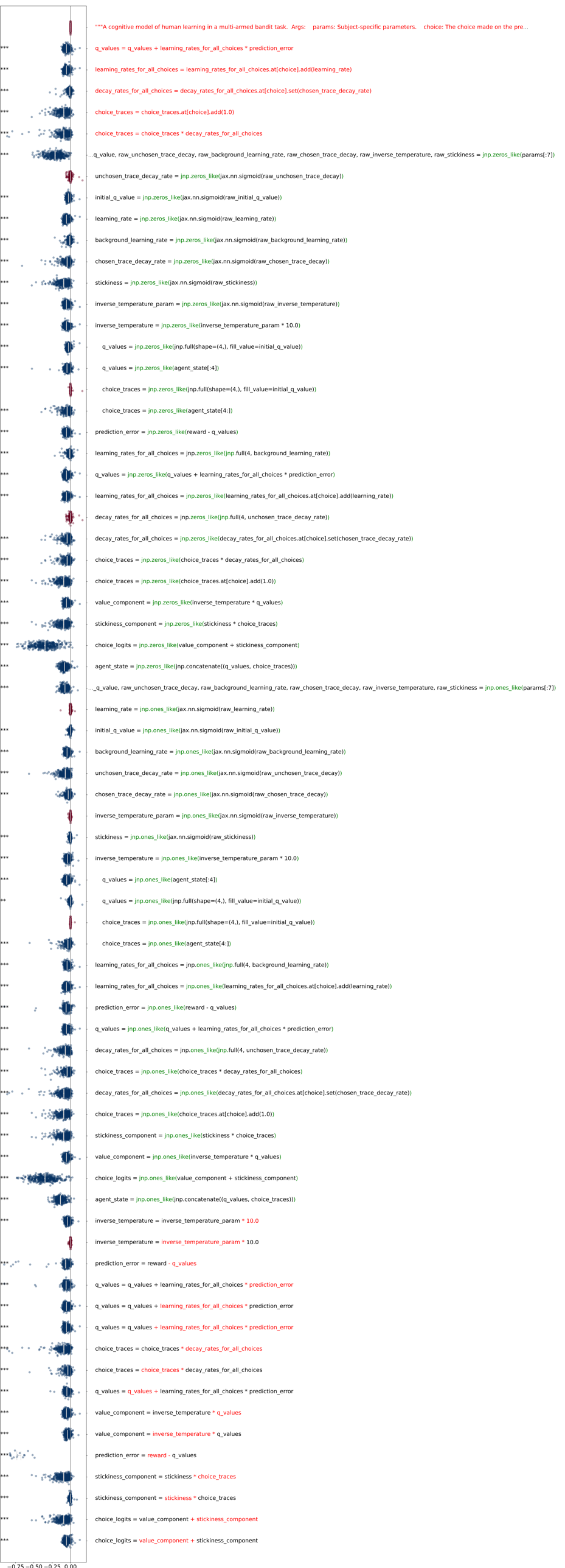

Supplement: Supplement 2 [file media-2.zip › ablation_performance_human_bandit_run2_low_floor_refactored_20260420.pdf]

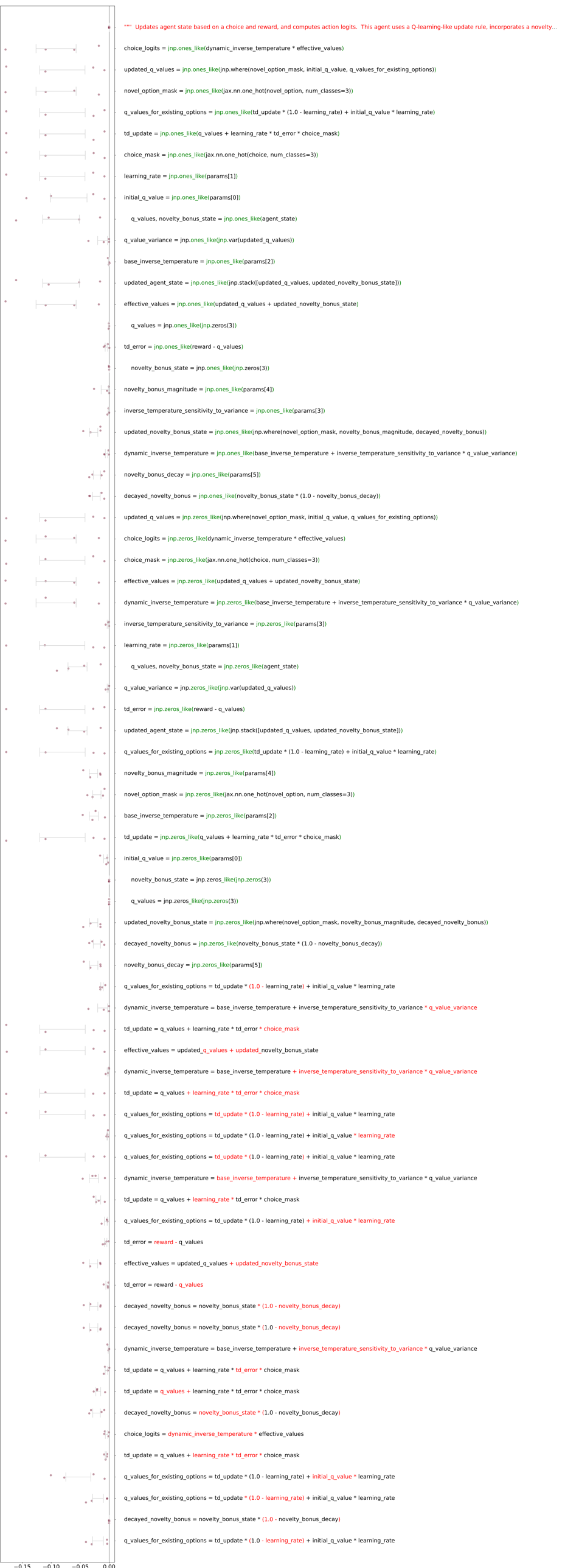

Supplement: Supplement 2 [file media-2.zip › ablation_performance_monkey_bandit_run3_low_floor_20260420.pdf]

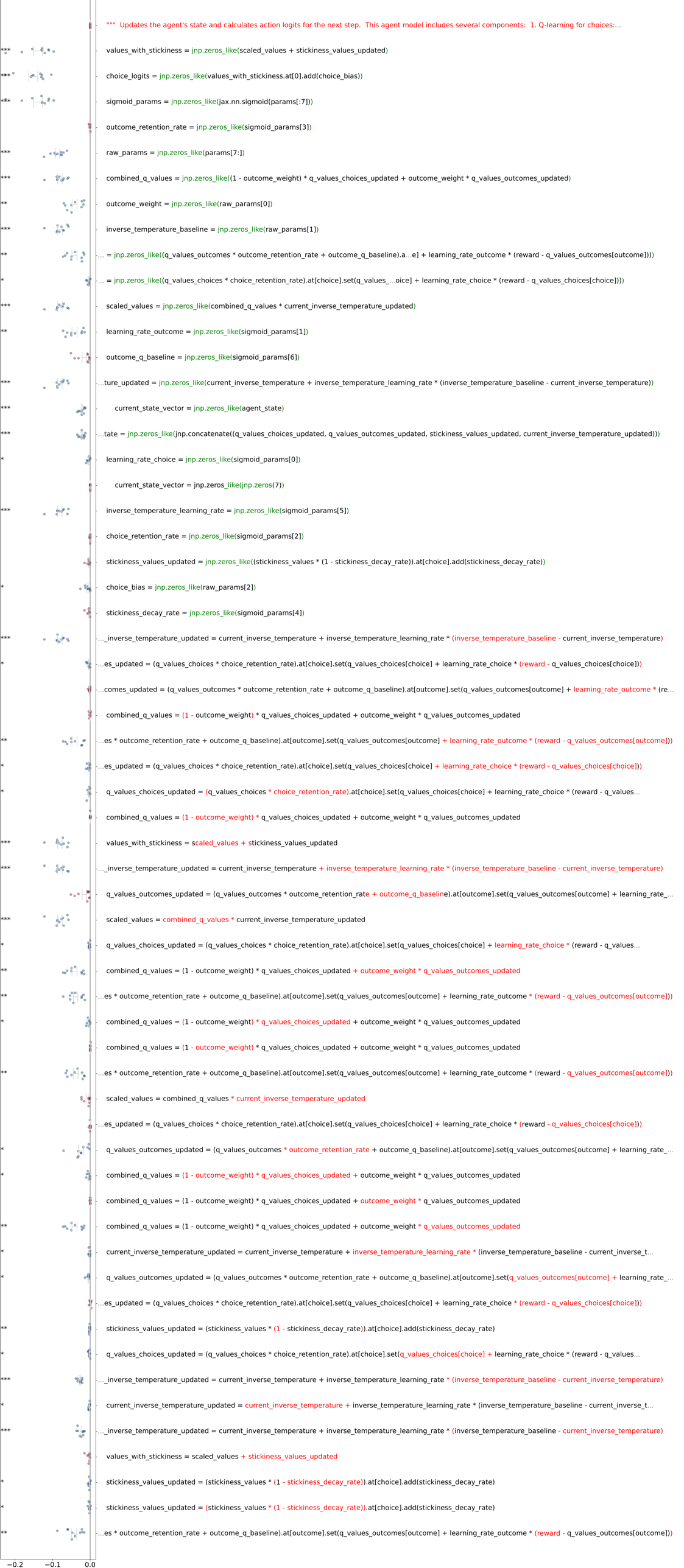

Supplement: Supplement 2 [file media-2.zip › ablation_performance_rat_twostep_run3_medium_floor_20260420.pdf]

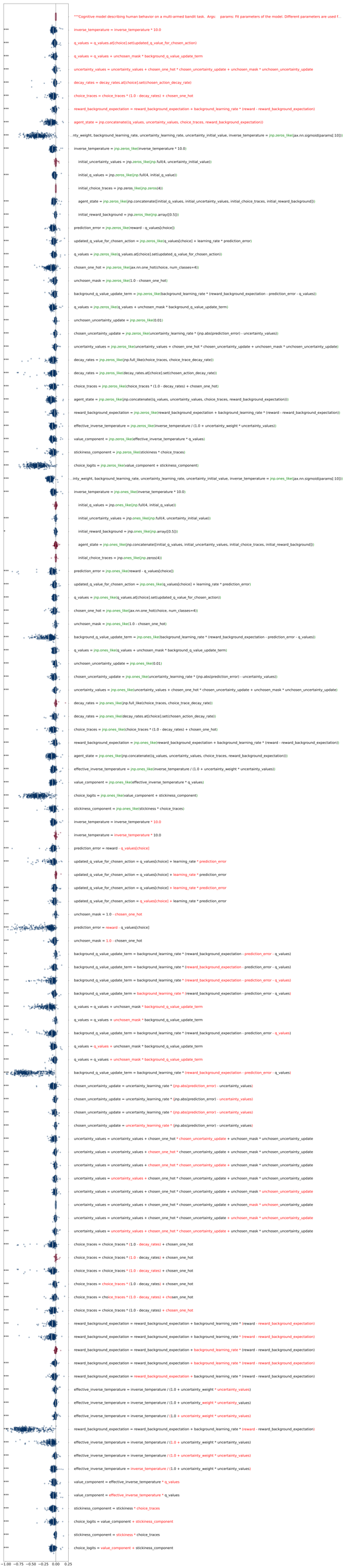

Supplement: Supplement 2 [file media-2.zip › ablation_performance_human_bandit_run2_medium_floor_refactored_20260420.pdf]

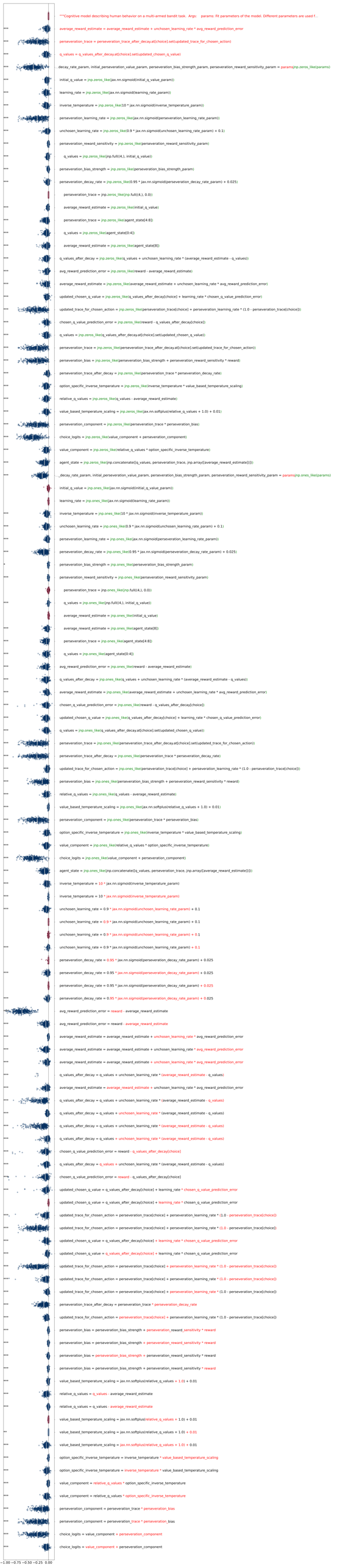

Supplement: Supplement 2 [file media-2.zip › ablation_performance_human_bandit_run1_medium_floor_refactored_20260420.pdf]
