# Supplementary material for: AI-Discovered Cognitive Models Reveal Novel Insights into Human and Animal Learning
Source: bioRxiv. 2026 May 21:2026.05.18.725921. Preprint. [Version 1] doi: 10.64898/2026.05.18.725921 (PMC13228651; doi:10.64898/2026.05.18.725921)
Supplement: Supplement 3 [file NIHPP2026.05.18.725921v1-supplement-3.pdf]

bioRxiv preprint doi: https://doi.org/10.64898/2026.05.18.725921; this version posted May 21, 2026. The copyright holder for this preprint (which was not certified by peer review) is the author/funder, who has granted bioRxiv a license to display the preprint in perpetuity. It is made available under a CC-BY 4.0 International license.

Appendix A Further Analysis of Individual Datasets

bioRxiv preprint doi: https://doi.org/10.64898/2026.05.18.725921; this version posted May 21, 2026. The copyright holder for this preprint (which was not certified by peer review) is the author/funder, who has granted bioRxiv a license to display the preprint in perpetuity. It is made available under a CC-BY 4.0 International license.

A.1 Human Bandit

A.1.1 Details of the dataset

Eckstein et al. [10] consider human participants performing a four-alternative task with graded rewards. Participants performed the task online, and indicated their choice on each trial by pressing either 'D', 'F', 'J', or 'K' on their keyboard. Reward was indicated by displaying an integer number of 'points' between 0 and 100, which subjects were asked to maximize. Available rewards followed independent bounded random walks with additional trial-unique noise. Each participant performed up to five back-to-back sessions of up to 150 trials each. The dataset contains choices from 862 participants performing 4,134 total sessions and 617,871 total trials.

We obtained this dataset from the following URL, where it is freely available under a permissive open-source license: https://osf.io/8xz3w/

A.1.2 Handcrafted Baseline Program

Eckstein et al. [10] performed an extensive comparison of a wide variety of computational cognitive models on this dataset. Following that, we adopt a model we refer to as "Perseverative Forgetting Q-Learning" as the human-discovered benchmark model. Note that we normalize reward to be between 0.0 and 1.0 as input to all baseline and DataDIVER models.

A.1.3 Discussion of evolved programs

Subjectively, we found that the low- and medium-floor programs could be understood with low or moderate effort. The high-floor programs were far more complex, owing to their greater length and diminished modularity, and yielded fewer discernible insights. Example low, medium, and high-floor programs are provided in Supplement A.1.7.

Cognitive Variables

Two cognitive variables that consistently arose across discovered programs were action values (or "Q-values"), which tracked expected rewards for each choice, and a "perseveration trace", which tracked choice history independent of reward (and went by a variety of different terms, e.g. "choice trace", "recency trace"). In many discovered programs, these were the only cognitive variables defined (all low-floor programs, one medium-floor program (run 3), and one high-floor (run 3) program). Another cognitive variable that arose in two out of three medium-floor programs tracked average reward (independent of choice), which interacted with the Q-value updates and the decision variable (described in more detail below). An additional discovered cognitive variable was found in one medium-floor program (run 2) and used average prediction errors per choice to update Q-values. However, this program had a low quality-of-fit and performed poorly on diagnostics. Two of the three high-floor programs did define additional cognitive variables, although their role and contribution to quality of fit was not straightforward due to the complexity of these programs.

For all low and medium-floor programs, action values and Perseveration Trace were updated modularly, with no interactions between the reward-dependent action

bioRxiv preprint doi: https://doi.org/10.64898/2026.05.18.725921; this version posted May 21, 2026. The copyright holder for this preprint (which was not certified by peer review) is the author/funder, who has granted bioRxiv a license to display the preprint in perpetuity. It is made available under a CC-BY 4.0 International license.

value update and reward-independent Perseveration Trace updates. This decomposition into modules parallels the modular hybrid neural network architecture identified by Eckstein et al. [10], who showed that neural network modules organized accordingly predicted behavior on this task better than other architectures. Here, we have the added benefit of being able to inspect these modules. This modularity broke down in high-floor programs, where reward-dependent terms are used to modulate perseveration updates.

Reward learning

Across all discovered programs, learning on the chosen action value $Q(c)$ presented in the form of a standard reward prediction error driven update given reward r: $Q(c) = Q(c) + \phi_i(r - Q(c))$. However, this proved misleading: ablations revealed that for most programs, the equation can be reduced further into a less conventional but much more simple update, $Q(c) = r$ with no loss in quality-of-fit. This is what we use for our synthesis program and discuss more in Section 4.1. Again, this parallels the observation from Eckstein et al. [10] that action values are not updated incrementally.

All discovered programs showed an update on all unchosen action values in which they slowly decayed toward some target that captured recent reward. While the particular statistic varied, this update generally has the effect of decaying $Q(c')$ toward the average over the recent reward history. For a given target $\bar{r}$ and an unchosen option c', the updates had the form $Q(c') = Q(c') + \phi(\bar{r} - Q(c'))$. Across programs, discovered targets included the previous reward r (run 1 and 2, low-floor), in others toward $Q(c)$ (run 3, all floors), and in others, toward a separate cognitive variable which tracked the average reward r_{avg} (run 1 and 2, medium- and high-floors).

Perseveration Updates

In many of our programs, as in many models in the literature, choice is not driven by past rewards (summarized by action values) alone, but also by a separate set of reward-independent mechanisms that capture a tendency to repeat actions that have been taken recently regardless of their outcome (perseveration).

The particular form of the discovered perseveration traces that implement this across our discovered programs showed interesting variations. Like the action values, the perseveration traces are 4-dimensional vectors in which each element corresponds to a different choice. All programs included some update that drives up the perseveration trace for the choice selected on the previous trial by incrementing it or setting it to 1. All programs implemented some kind of forgetting for the unchosen options, usually by decaying their perseveration traces towards 0 (7/9 programs). Collectively, this implements a tendency to repeat actions that were taken recently.

However, the remaining two discovered programs included an unusual forgetting pattern on the perseveration trace P for each unchosen option c' in which $P(c')$ was *reset* to 0 rather than gradually *decayed* under certain conditions. This interrupts the perseverative bout if even a single different action is taken.

In low-floor (run 1), this amounted to simply resetting $P(c')$ to 0, erasing the value that had been accumulated for this variable when it had been chosen. This suggests that there is no extra perseverative momentum on an action once even a single different

bioRxiv preprint doi: https://doi.org/10.64898/2026.05.18.725921; this version posted May 21, 2026. The copyright holder for this preprint (which was not certified by peer review) is the author/funder, who has granted bioRxiv a license to display the preprint in perpetuity. It is made available under a CC-BY 4.0 International license.

action is taken, even if that action has been taken many times prior to the deviation. There still might be some above chance likelihood of returning to that action, but it would have to be driven by the action values.

In medium-floor (run 3), the perseverative update had a more complex form of reset. The perseveration update for chosen options involved incrementing $P(c)$, clipping it at 5.0, and scaling by a positive parameter ϕ_c: $P(c) \leftarrow \phi_c \min\{(P(c)+1), 5.0\}$. The update on unchosen options is $P(c') \leftarrow \phi_u P(c')(5.0 - P(c'))$ for positive parameter ϕ_u. This means that $P(c')$ will be reset to 0 when $P(c')$ is close to 5, and remain positive if it is between 0 and 5. Thus, this reset only occurs for very long runs, where $P(c)$ has accumulated to the clipped value of 5. Intuitively, this means that after the subject has been making the same choice for a long time, their making a different choice means that they have stopped perseverating on that run: there is no extra perseverative momentum on that choice. However, for short runs (<5), some perseverative momentum remains.

Nonlinear, Nonstationary Exploration

In the medium- and high-floor programs, action values undergo a nonlinear, nonstationary transformation before merging with the perseveration component. This has the effect of making behavior more deterministic when action values are further from 0 or from the nonstationary average recent reward. This means that the nonlinearity mapping action values to decision variables is not merely a softmax.

A.1.4 Discussion of synthesis program

The synthesis program included versions of each of the motifs described above. It defined three cognitive variables: action values, a perseveration trace, and a term which tracked the recent average reward. The chosen action value was updated by being overwritten by the received reward; as such, there was no learning rate parameter needed to update the chosen action's value. The unchosen action values were updated by decaying toward the recent average reward. The nonlinearity applied to the action values before their combination with the perseveration trace was

$$f(x) = \phi[\ln(1 + e^{Q - r_{\text{avg}}}) + 0.01]$$

which was the particular nonlinearity from the medium-floor, run 1 program.

The perseveration trace was updated using the nonlinear forgetting rule from medium-floor, run 3, as this program exhibited the best performance at recovering the run return diagnostic .

The synthesis program is shown in Supplement A.1.5.

A.1.5 Code: synthesis program

```
def human_bandit_synthesis(
    params: chex.Array,
    choice: int,
    reward: float,
    agent_state: chex.Array | None,
) -> tuple[chex.Array, chex.Array]:
```

bioRxiv preprint doi: https://doi.org/10.64898/2026.05.18.725921; this version posted May 21, 2026. The copyright holder for this preprint (which was not certified by peer review) is the author/funder, who has granted bioRxiv a license to display the preprint in perpetuity. It is made available under a CC-BY 4.0 International license.

Table A1: Evaluation performance and program complexity for models in the *Human Bandit* dataset. For programs generated by the "Simplify" stage, Floor represents the quality-of-fit threshold below which programs are discarded (see Section 7.6.2). Score indicates the average normalized likelihood across evaluation subjects (see Section 7.3); Effort is Halstead effort. State, Params, and Lines indicate the number of state variables, per-subject parameters, and lines of code respectively.

Model type	Floor	Run	Score	Effort	State	Params	Lines
Handcrafted Baseline	–	–	0.5610	16,197	4	4	59
RNN Baseline	–	–	0.6274	–	–	–	–
Stage 1: "Maximize Quality-of-Fit"	–	1	0.6188	703,382	20	10	350
		2	0.6209	780,146	30	10	555
		3	0.6175	112,167	8	10	169
Stage 2: "Simplify"	50%	1	0.5988	9,645	8	7	108
		2	0.5998	13,660	8	7	114
		3	0.5929	7,484	8	6	99
	75%	1	0.6075	21,296	9	10	141
		2	0.6103	31,285	13	10	154
		3	0.6111	22,647	8	9	138
	90%	1	0.6178	122,938	13	9	210
		2	0.6166	157,612	22	10	307
		3	0.6170	85,125	8	9	184
Synthesis Program	–	–	0.6068	25,047	9	7	106

```
"""Handcrafted agent for the Human Bandit task based on insights from discovered
  programs.

Has the following elements
* Maintains separate q_values, choice_trace, and average_reward state variables.
* Updates q_values on chosen by replacing with recent reward.
* Updates q_values with decay toward average reward on unchosen
* Increment choice_trace for choice, clip at 5. Quadratic reset on unchosen.
* Nonlinearity on q-values: rel_q * softplus(rel_q + 1)
"""

# === 1. Unpack and Transform Model Parameters ===
# Apply a sigmoid function to the first 8 parameters to constrain them
# between 0 and 1. This is common for rates, weights, and probabilities.

(raw_initial_q_value,
 raw_inverse_temperature_base,
 learning_rate_unchosen_logit,
 recency_chosen_decay_logit,
 recency_unchosen_decay_logit,
 recency_weight_chosen,
 *_) = params

initial_q_value = jax.nn.sigmoid(raw_initial_q_value)
inverse_temperature_base = 10 * jax.nn.sigmoid(raw_inverse_temperature_base)
learning_rate_unchosen = 0.9 * jax.nn.sigmoid(learning_rate_unchosen_logit) + 0.1
recency_chosen_decay = jax.nn.sigmoid(recency_chosen_decay_logit)
recency_unchosen_decay = 0.95 * jax.nn.sigmoid(recency_unchosen_decay_logit) +
  0.025

```

bioRxiv preprint doi: https://doi.org/10.64898/2026.05.18.725921; this version posted May 21, 2026. The copyright holder for this preprint (which was not certified by peer review) is the author/funder, who has granted bioRxiv a license to display the preprint in perpetuity. It is made available under a CC-BY 4.0 International license.

```
# === 2. Initialize or Load Agent's Internal State ===
# The agent's state consists of Q-values (expected reward for each option),
# a recency trace (memory of recent choices), and average reward
if agent_state is None:
  # If this is the first trial, initialize Q-values and recency trace.
  q_values = jnp.full(shape=(4,), fill_value=initial_q_value)
  choice_trace = jnp.zeros(shape=(4,))
  average_reward = initial_q_value
else:
  # If not the first trial, load the state from the previous trial.
  q_values = agent_state[:4]       # First 4 elements are Q-values
  choice_trace = agent_state[4:8]  # Last 4 elements are recency traces
  average_reward = agent_state[8]

# === 3. Update Q-Values Based on Last Trial's Outcome ===

# 3a. Update the estimate of the average reward.
# This tracks the overall reward rate of the environment.

avg_reward_prediction_error = reward - average_reward
average_reward += learning_rate_unchosen * avg_reward_prediction_error

q_values += (
    learning_rate_unchosen * (average_reward - q_values)
)

# Update the Q-value for the chosen option by replacing with recent reward
q_values = q_values.at[choice].set(reward)

# === 4. Update Recency Trace ===
# The recency trace tracks which options were chosen recently.

# Create a one-hot vector to easily distinguish the chosen option.
chosen_one_hot = jax.nn.one_hot(choice, num_classes=4)

# Update recency trace.
recency_update_for_chosen = (
    jnp.minimum(choice_trace + 1.0, 5.0) * recency_chosen_decay
)
recency_update_for_unchosen = (
    choice_trace * recency_unchosen_decay * (1 - choice_trace / 5.0)
)

# Apply the appropriate update rule to each element of the recency trace.
choice_trace = jnp.where(
    chosen_one_hot,
    recency_update_for_chosen,
    recency_update_for_unchosen,
)

# === 5. Compute Choice Logits for the Next Trial ===
# Logits are the raw, unnormalized scores for each choice. A higher logit
# means a higher probability of being chosen.

choice_trace_component = recency_weight_chosen * choice_trace  # one weight on all.

# Nonlinearity on q-values.
relative_q_values = q_values - average_reward
value_based_temperature_scaling = jax.nn.softplus(relative_q_values + 1.0) + 0.01
adaptive_inverse_temperature = inverse_temperature_base * value_based_temperature_scaling
value_component = adaptive_inverse_temperature * relative_q_values

choice_logits = value_component + choice_trace_component

# === 6. Prepare State for the Next Trial and Return ===
```

bioRxiv preprint doi: https://doi.org/10.64898/2026.05.18.725921; this version posted May 21, 2026. The copyright holder for this preprint (which was not certified by peer review) is the author/funder, who has granted bioRxiv a license to display the preprint in perpetuity. It is made available under a CC-BY 4.0 International license.

```
    # Concatenate the updated Q-values and recency trace into a single array.
    agent_state = jnp.concatenate((q_values, choice_trace, average_reward[jnp.newaxis
      ]))

    return choice_logits, agent_state
```

Code 1: Synthesis program for the human bandit dataset.

A.1.6 Code: stage 1 ("Maximize Quality-of-Fit") programs

```
def human_bandit_run1_fitonly(
    params: chex.Array,
    choice: int,
    reward: float,
    agent_state: Optional[chex.Array],
) -> tuple[chex.Array, chex.Array]:
  """Cognitive model describing human behavior on a multi-armed bandit task.

  Assumes the agent is presented with four options on each trial.

  Args:
    params: Fit parameters of the model. Different parameters are used for
      different subjects.
    choice: Choice made by the agent on the previous trial. 0, 1, 2, or 3
    reward: Reward received by the agent on the previous trial. A float between
      0 and 1.
    agent_state: The current state of the cognitive model.

  Returns:
    choice_logits: The probabilities that the agent will choose option 0, 1, 2,
      or 3 on the next trial, expressed as logits.
    agent_state: New agent state
  """
  # Do not remove comments or TODOs from this program.

  # Define parameters. All learning rates and decay rates are transformed with the
    logistic function
  # to be between 0 and 1. Inverse temperature is scaled to be between 0 and 10.
  # Perseveration biases are transformed with softplus to be non-negative.
  initial_q_value = jax.nn.sigmoid(params[0])
  learning_rate = jax.nn.sigmoid(params[1])
  inverse_temperature = 10 * jax.nn.sigmoid(params[2]) # Scales from 0 to 10
  q_value_prior_weight = jax.nn.sigmoid(params[3]) # Weight for combining
    initial_q_value and average_reward_estimate
  unchosen_learning_rate = 0.95 * jax.nn.sigmoid(params[4]) + 0.025 # Constrained
    to be between 0.025 and 0.975 for stability.
  perseveration_learning_rate = jax.nn.sigmoid(params[5])
  perseveration_decay_rate = 0.95 * jax.nn.sigmoid(params[6]) + 0.025 # Constrained
     to be between 0.025 and 0.975 for stability.
  initial_perseveration_value = jax.nn.sigmoid(params[7])
  perseveration_bias_strength = jax.nn.softplus(params[8]) # Non-negative
  perseveration_reward_sensitivity = 5 * jax.nn.softplus(params[9]) # Non-negative,
     allowing for a broader positive range.

  # Initialize Q-values, perseveration trace, average reward estimate, and recency
    trace if state is None.
  if agent_state is None:
    q_values = jnp.full((4,), initial_q_value)
    perseveration_trace = jnp.full((4,), initial_perseveration_value)
    average_reward_estimate = initial_q_value
    recency_trace = jnp.full((4,), 0.0) # New state variable to track recency,
    initialized to zero.
    reward_variance_estimate = jnp.array(0.0) # New state variable for tracking
    reward variance
    q_value_uncertainty = jnp.full((4,), 0.5) # New state variable for tracking Q-
    value uncertainty, initialized to a neutral value.
```

bioRxiv preprint doi: https://doi.org/10.64898/2026.05.18.725921; this version posted May 21, 2026. The copyright holder for this preprint (which was not certified by peer review) is the author/funder, who has granted bioRxiv a license to display the preprint in perpetuity. It is made available under a CC-BY 4.0 International license.

```
        uncertainty_prediction_error_estimate = jnp.array(0.0) # New state variable for tracking meta-level uncertainty prediction error
        agent_state = jnp.concatenate([q_values, perseveration_trace, jnp.array([average_reward_estimate]), recency_trace, jnp.array([reward_variance_estimate]), q_value_uncertainty, jnp.array([uncertainty_prediction_error_estimate])])

    # Unpack Q-values, perseveration trace, average reward estimate, recency trace, reward variance estimate, Q-value uncertainty, and uncertainty prediction error from the agent state.
    q_values = agent_state[:4]
    perseveration_trace = agent_state[4:8]
    average_reward_estimate = agent_state[8]
    recency_trace = agent_state[9:13] # Unpack the new recency_trace
    reward_variance_estimate = agent_state[13] # Unpack the new reward variance estimate
    q_value_uncertainty = agent_state[14:18] # Unpack the new q_value_uncertainty
    uncertainty_prediction_error_estimate = agent_state[18] # Unpack the new uncertainty prediction error estimate
    exploration_bonus_strength = agent_state[19] # Unpack the new general exploration bonus strength.

    # Compute a dynamic prior for Q-values, a weighted average of initial Q and current average reward.
    # Q-values decay towards this dynamic prior, effectively integrating the previous decay and adding an adaptive baseline.
    dynamic_q_prior = q_value_prior_weight * initial_q_value + (1 - q_value_prior_weight) * average_reward_estimate
    # Introduce a global, small decay rate towards the initial_q_value. This acts as a fixed prior.
    global_q_decay_to_initial = unchosen_learning_rate * 0.1 # A small fraction of the unchosen learning rate.
    q_values = q_values * (1 - global_q_decay_to_initial) + initial_q_value * global_q_decay_to_initial # Decay all Q-values towards initial_q_value
    q_values = q_values * (1 - unchosen_learning_rate) + dynamic_q_prior * unchosen_learning_rate # Unchosen learning rate acts as the decay to the dynamic prior

    # Update the average reward estimate using a Rescorla-Wagner-like rule.
    # This tracks the overall rewardingness of the environment.
    reward_prediction_error_avg = reward - average_reward_estimate
    # Modulate the learning rate for the average reward estimate.
    # It now adapts based on the signed prediction error, increasing for large errors in either direction,
    # but also adapting based on the consistency of the error (e.g., if it consistently overestimates, learn faster).
    # The factor is now also scaled by the current average reward, allowing for more dynamic learning in different reward environments.
    avg_reward_error_magnitude_factor = 1.0 + jax.nn.softplus(jnp.abs(reward_prediction_error_avg))
    # Introduce a term that scales learning based on whether the average reward is currently "high" or "low",
    # allowing for different sensitivity depending on the environmental baseline.
    avg_reward_context_scaling = 0.5 + 1.5 * jax.nn.sigmoid(average_reward_estimate - 0.5) # Scale between 0.5 and 2.0
    effective_avg_reward_learning_rate = unchosen_learning_rate * avg_reward_error_magnitude_factor * avg_reward_context_scaling
    effective_avg_reward_learning_rate = jnp.clip(effective_avg_reward_learning_rate, 0.0, 1.0) # Ensure rates are within bounds
    average_reward_estimate = average_reward_estimate + effective_avg_reward_learning_rate * reward_prediction_error_avg
    # Update the reward variance estimate using an exponential moving average.
    # This tracks the volatility of the rewards over time.
    reward_squared_error = (reward - average_reward_estimate)**2
    variance_learning_rate = unchosen_learning_rate * 0.5 # A fraction of the unchosen_learning_rate
    reward_variance_estimate = reward_variance_estimate * (1 - variance_learning_rate) + reward_squared_error * variance_learning_rate

```

bioRxiv preprint doi: https://doi.org/10.64898/2026.05.18.725921; this version posted May 21, 2026. The copyright holder for this preprint (which was not certified by peer review) is the author/funder, who has granted bioRxiv a license to display the preprint in perpetuity. It is made available under a CC-BY 4.0 International license.

```
# Modulate the decay rate for the average reward estimate based on the magnitude of the average reward prediction error
# AND the estimated reward variance. Higher variance suggests greater environmental volatility, demanding faster decay.
adaptive_avg_reward_decay_factor = unchosen_learning_rate * 0.1 * (1.0 + jax.nn.softplus(jnp.abs(reward_prediction_error_avg) * 2.0))
# Introduce variance-based scaling: higher variance means more decay towards initial Q.
variance_decay_scaling = 1.0 + jax.nn.softplus(reward_variance_estimate * 10.0) # Scale up decay based on variance
adaptive_avg_reward_decay_factor = adaptive_avg_reward_decay_factor * variance_decay_scaling
adaptive_avg_reward_decay_factor = jnp.clip(adaptive_avg_reward_decay_factor, 0.0, 1.0) # Ensure rates are within bounds
average_reward_estimate = average_reward_estimate * (1 - adaptive_avg_reward_decay_factor) + initial_q_value * adaptive_avg_reward_decay_factor

unchosen_mask = jnp.arange(4) != choice
# Option-specific uncertainty: higher for Q-values closer to the average reward estimate (less distinct information).
# This factor will modulate learning rates, promoting more learning for uncertain options.
option_uncertainty_factor = 1.0 + (1.0 - jax.nn.sigmoid(jnp.abs(q_values - average_reward_estimate) * 5.0))

# Update the Q-value for the chosen option using a standard Rescorla-Wagner rule.
# The prediction error is simply the difference between the reward and the chosen option's Q-value.
prediction_error = reward - q_values[choice]
# Modulate the learning rate for the chosen option by the absolute magnitude of the prediction error
# (surprise), the option's inherent uncertainty, AND its recency.
# Also introduce a 'meta-learning' component where the learning rate itself adapts.
chosen_prediction_error_salience_factor = 1.0 + jax.nn.softplus(jnp.abs(prediction_error))
volatility_sensitive_learning_rate_scaling = 1.0 - 0.5 * jax.nn.sigmoid(reward_variance_estimate * 10.0) # Scales from 1.0 (low variance) to 0.5 (high variance)
# New: Scale the chosen learning rate by the Q-value uncertainty for that option.
# Higher uncertainty means a higher effective learning rate for the chosen option.
uncertainty_scaled_learning_rate_factor = 0.5 + 1.5 * q_value_uncertainty[choice] # Scales from 0.5 (low uncertainty) to 2.0 (high uncertainty)

# Introduce an 'omission salience' factor: stronger learning when a high Q-value results in a near-zero reward.
# This factor becomes high when reward is near 0 and prediction error is strongly negative.
omission_salience_factor = jnp.where(
    (reward < 0.1) & (prediction_error < -0.2), # Check for near-zero reward and significant negative prediction error
    1.0 + 3.0 * jax.nn.sigmoid(-prediction_error * 5.0), # Amplify learning rate for significant omissions, scales from 1 to 4
    1.0 # No amplification otherwise
)
effective_chosen_learning_rate = learning_rate * chosen_prediction_error_salience_factor * option_uncertainty_factor[choice] * volatility_sensitive_learning_rate_scaling * uncertainty_scaled_learning_rate_factor * omission_salience_factor
effective_chosen_learning_rate = jnp.clip(effective_chosen_learning_rate, 0.0, 1.0) # Ensure rates are within bounds
q_values = q_values.at[choice].set(
    q_values[choice] + effective_chosen_learning_rate * prediction_error
)

# For unchosen options, Q-values decay towards the average reward estimate.
```

bioRxiv preprint doi: https://doi.org/10.64898/2026.05.18.725921; this version posted May 21, 2026. The copyright holder for this preprint (which was not certified by peer review) is the author/funder, who has granted bioRxiv a license to display the preprint in perpetuity. It is made available under a CC-BY 4.0 International license.

```
# This decay rate is modulated by the option's uncertainty and its recency,
# and also by the global surprise signal from the chosen option's outcome.
# Higher uncertainty, lower recency, and higher global surprise lead to faster decay.
global_surprise_factor = 1.0 + jax.nn.softplus(jnp.abs(prediction_error)) # Global surprise based on chosen option's prediction error
recency_amplified_decay_factor = 1.0 + (1.0 - recency_trace) * 0.5 # Amplify decay for less recent, scales from 1.0 to 1.5
uncertainty_amplified_decay_factor = 1.0 + option_uncertainty_factor * 0.5 # Amplify decay based on higher option uncertainty
global_surprise_amplified_decay_factor = 1.0 + global_surprise_factor * 0.5 # Amplify decay based on global surprise
effective_unchosen_decay_rate = unchosen_learning_rate * recency_amplified_decay_factor * uncertainty_amplified_decay_factor * global_surprise_amplified_decay_factor
effective_unchosen_decay_rate = jnp.clip(effective_unchosen_decay_rate, 0.0, 1.0)  # Ensure rates are within bounds

q_values = jnp.where(
    unchosen_mask,
    q_values * (1 - effective_unchosen_decay_rate) + average_reward_estimate * effective_unchosen_decay_rate,
    q_values,  # Chosen option's Q-value has already been updated.
)

# Update the perseveration trace for the chosen option. It increases towards 1.0
# with a learning rate modulated by the prediction error (stronger for positive errors).
# Modulate the perseveration learning rate based on the average reward estimate.
# When average reward is low, perseveration learning rate is boosted, and vice-versa.
average_reward_modulated_perseveration_lr_factor = 1.0 + jax.nn.tanh(0.5 - average_reward_estimate) # Scales from ~0.5 (high avg reward) to ~1.5 (low avg reward)
chosen_perseveration_learning_rate = perseveration_learning_rate * jax.nn.sigmoid(prediction_error * 5) * average_reward_modulated_perseveration_lr_factor
perseveration_trace_update_chosen = perseveration_trace[choice] + chosen_perseveration_learning_rate * (1 - perseveration_trace[choice])

# For unchosen options, apply decay. The decay rate is modulated by prediction error,
# causing faster decay when outcomes are surprising (higher uncertainty).
# For unchosen options, apply decay. The decay rate is now modulated by the unchosen option's own
# uncertainty (how far its Q-value is from the average reward estimate) AND the magnitude of the
# prediction error for the chosen option, reflecting overall environmental surprise.
jnp.abs(q_values - average_reward_estimate)
# Stronger overall prediction error (surprise) can lead to faster decay of unchosen perseveration
# For unchosen options, apply decay. The decay rate is modulated by:
# 1. How 'bad' the unchosen option would have been (Q-value relative to current reward).
# 2. The global context of average reward (faster decay in low-reward environments).
# 3. The overall surprise (magnitude of chosen option's prediction error).
# 4. The 'uncertainty' of the unchosen perseveration trace itself (how far it is from its initial value).

# Factor based on how 'bad' the unchosen option might have been. Higher if reward was much better than unchosen Q.
unchosen_opportunity_cost_factor = jax.nn.sigmoid((reward - q_values) * 5.0)

# Factor based on the overall environmental rewardingness (amplify decay if average reward is below 0.5).
global_average_reward_context_factor = 1.0 + jax.nn.relu(average_reward_estimate - 0.5)
```

bioRxiv preprint doi: https://doi.org/10.64898/2026.05.18.725921; this version posted May 21, 2026. The copyright holder for this preprint (which was not certified by peer review) is the author/funder, who has granted bioRxiv a license to display the preprint in perpetuity. It is made available under a CC-BY 4.0 International license.

```

# Factor based on the global surprise from the chosen option's outcome.
global_outcome_surprise_factor = 1.0 + jax.nn.softplus(jnp.abs(prediction_error))

# Factor based on how 'uncertain' or 'outdated' the unchosen perseveration trace is.
perseveration_uncertainty_factor = 1.0 + jax.nn.softplus(jnp.abs(perseveration_trace - initial_perseveration_value))

# Modulate the perseveration decay rate by the recency trace for unchosen options.
# Lower recency (i.e., less recently chosen) should lead to faster decay of perseveration.
recency_modulated_decay_factor = 1.0 + (1.0 - recency_trace) * 2.0 # Scales from 1.0 (recency=1) to 3.0 (recency=0)

effective_perseveration_decay_unchosen = perseveration_decay_rate * (
    1.0 + unchosen_opportunity_cost_factor + global_average_reward_context_factor
    + global_outcome_surprise_factor + perseveration_uncertainty_factor
) * recency_modulated_decay_factor
effective_perseveration_decay_unchosen = jnp.clip(effective_perseveration_decay_unchosen, 0.0, 1.0) # Ensure rates are within bounds
perseveration_trace_update_unchosen = perseveration_trace * (1 - effective_perseveration_decay_unchosen) + initial_perseveration_value * effective_perseveration_decay_unchosen

# Update the perseveration trace by combining updates for chosen and unchosen options.
perseveration_trace = jnp.where(
    unchosen_mask,
    perseveration_trace_update_unchosen,
    perseveration_trace_update_chosen,
)

# Update the recency trace. It increases for the chosen option and decays for unchosen options.
# Modulate the recency trace update for the chosen option. A positive prediction error leads to stronger accumulation,
# while a negative prediction error weakens it, or even promotes decay.
recency_update_factor = 1.0 + jax.nn.tanh(prediction_error * 2.0) # Scales from ~0 to ~2
reward_modulated_recency_learning_rate = perseveration_learning_rate * recency_update_factor
reward_modulated_recency_learning_rate = jnp.clip(reward_modulated_recency_learning_rate, 0.0, 1.0)
recency_trace_update_chosen = recency_trace[choice] + reward_modulated_recency_learning_rate * (1 - recency_trace[choice])
# Modulate the recency trace decay for unchosen options based on their Q-value similarity to the chosen option.
# Options with Q-values closer to the chosen option's Q-value decay slower.
jax.nn.sigmoid(5.0 * (1.0 - jnp.abs(q_values - q_values[choice]))) # Higher for more similar Q-values, range 0-1
# Decay rate is inverse of similarity, so higher similarity means lower decay rate
# Modulate the recency trace decay for unchosen options based on their Q-value similarity to the chosen option
# AND their inherent Q-value uncertainty. Higher uncertainty should lead to faster decay.
q_value_similarity_to_chosen = jax.nn.sigmoid(5.0 * (1.0 - jnp.abs(q_values - q_values[choice]))) # Higher for more similar Q-values, range 0-1
uncertainty_amplified_recency_decay_factor = 1.0 + q_value_uncertainty * 2.0 # Higher uncertainty -> faster decay, scales from 1.0 (uncertainty=0) to 3.0 (uncertainty=1)
# Decay rate is inverse of similarity, so higher similarity means lower decay rate, but amplified by uncertainty.
recency_decay_scaling_factor = (1.0 - 0.9 * q_value_similarity_to_chosen) * uncertainty_amplified_recency_decay_factor # Scales from ~0.1 to ~3.0
```

bioRxiv preprint doi: https://doi.org/10.64898/2026.05.18.725921; this version posted May 21, 2026. The copyright holder for this preprint (which was not certified by peer review) is the author/funder, who has granted bioRxiv a license to display the preprint in perpetuity. It is made available under a CC-BY 4.0 International license.

```
effective_recency_decay_rate = perseveration_decay_rate *
  recency_decay_scaling_factor
effective_recency_decay_rate = jnp.clip(effective_recency_decay_rate, 0.0, 1.0) #
   Ensure rates are within bounds
recency_trace_update_unchosen = recency_trace * (1 - effective_recency_decay_rate
  )

recency_trace = jnp.where(
    unchosen_mask,
    recency_trace_update_unchosen,
    recency_trace_update_chosen,
)
# Ensure the recency trace remains within reasonable bounds
recency_trace = jnp.clip(recency_trace, 0.0, 1.0) # Bounds 0 to 1 as it's a '
  trace'

# Update the Q-value uncertainty trace.
# For the chosen option, uncertainty decreases if prediction error is small (more
   certain),
# and increases if prediction error is large (less certain).
# For unchosen options, uncertainty slowly increases over time (decay towards
  maximum uncertainty).
uncertainty_decay_rate = unchosen_learning_rate * 0.5 # A fraction of the
  unchosen learning rate for decay.
# Modulate the uncertainty learning rate for the chosen option by the inverse of
  reward variance.
# Lower variance (more stable environment) leads to a higher learning rate for
  uncertainty.
volatility_sensitive_uncertainty_lr_scaling = 1.0 - 0.9 * jax.nn.sigmoid(
  reward_variance_estimate * 10.0) # Scales from 1.0 (low variance) to 0.1 (high
   variance)
# For the chosen option, uncertainty decreases as new evidence is gathered.
# The reduction in uncertainty is stronger when the prediction error is small,
# indicating more reliable information gain.
# If the prediction error is large, the reduction in uncertainty is smaller,
# as the Q-value estimate was less accurate.
# Scale this reduction by the volatility-sensitive learning rate.
# For the chosen option, uncertainty decreases as new evidence is gathered.
# The reduction in uncertainty is stronger when the prediction error is small,
# indicating more reliable information gain.
# If the prediction error is large, the reduction in uncertainty is smaller,
# as the Q-value estimate was less accurate.
uncertainty_reduction_magnitude = 1.0 - jax.nn.sigmoid(jnp.abs(prediction_error)
  * 3.0) # Larger reduction for smaller errors.
# Scale this reduction by the volatility-sensitive learning rate.
effective_uncertainty_reduction = uncertainty_reduction_magnitude *
  volatility_sensitive_uncertainty_lr_scaling

# Calculate the actual change in uncertainty for the chosen option.
# Predict the change in uncertainty. A simple prediction could be proportional to
   the effective_uncertainty_reduction.

# Update the meta-level uncertainty prediction error estimate.
# For the chosen option, uncertainty decreases as new evidence is gathered.
# The reduction in uncertainty is stronger when the prediction error is small.
# For the chosen option, uncertainty decreases as new evidence is gathered.
# The reduction in uncertainty is stronger when the prediction error is small.
# Predict the uncertainty *before* update for UPE calculation.
predicted_uncertainty_after_update = q_value_uncertainty[choice] * (1.0 -
  effective_uncertainty_reduction)

# Introduce a 'confidence bonus' for chosen options that lead to very low
  prediction error and high reward.
# This makes the agent more certain about high-value, predictable options.
confidence_bonus_factor = jnp.where(
    (jnp.abs(prediction_error) < 0.1) & (reward > 0.8), # Low error, high reward
    0.2, # Significant confidence boost (reduction in uncertainty)
    0.0 # No additional confidence bonus otherwise
```

bioRxiv preprint doi: https://doi.org/10.64898/2026.05.18.725921; this version posted May 21, 2026. The copyright holder for this preprint (which was not certified by peer review) is the author/funder, who has granted bioRxiv a license to display the preprint in perpetuity. It is made available under a CC-BY 4.0 International license.

```
)
uncertainty_update_chosen = jnp.maximum(0.0, predicted_uncertainty_after_update - confidence_bonus_factor) # Ensure uncertainty doesn't go below 0

# Calculate Uncertainty Prediction Error (UPE) for the chosen option.
# UPE is the difference between the actual updated uncertainty and the predicted uncertainty.
# If the actual updated uncertainty is *lower* than predicted (i.e., less uncertain than expected), UPE is negative.
# If actual updated uncertainty is *higher* than predicted (i.e., more uncertain than expected), UPE is positive.
uncertainty_prediction_error = uncertainty_update_chosen - predicted_uncertainty_after_update

# Update the meta-level uncertainty prediction error estimate.
# Use a simple Rescorla-Wagner rule for UPE learning, modulated by the general uncertainty decay rate.
upe_learning_rate = uncertainty_decay_rate * 2.0 # UPE learns faster
uncertainty_prediction_error_estimate = uncertainty_prediction_error_estimate + upe_learning_rate * uncertainty_prediction_error

# For unchosen options, decay uncertainty towards initial_q_value's uncertainty, modulated by reward variance.
# If reward variance is high (unstable), uncertainty decays slower, reflecting persistent lack of knowledge.
# For unchosen options, uncertainty slowly increases over time (decay towards maximum uncertainty of 1.0)
# BUT it also decays towards a level of uncertainty inversely proportional to how distinct its Q-value is from the average.
# If an option's Q-value is very distinct (far from average), its inherent uncertainty should be lower.
# If an option's Q-value is close to the average, its inherent uncertainty should be higher (more ambiguous).
inherent_uncertainty_from_distinctiveness = 1.0 - jax.nn.sigmoid(jnp.abs(q_values - average_reward_estimate) * 5.0) # Higher for less distinct Q-values.
# Modulate the uncertainty decay rate by reward variance. In high variance, uncertainty decays slower for unchosen options.
variance_modulated_uncertainty_decay_rate = uncertainty_decay_rate * (1.0 - jax.nn.sigmoid(reward_variance_estimate * 5.0))
# The target uncertainty is a blend of inherent uncertainty and a slow decay towards a higher default (e.g., 0.8 to reflect lack of recent info).
target_unchosen_uncertainty = 0.8 * inherent_uncertainty_from_distinctiveness + 0.2 * 1.0 # Bias towards 1.0 but influenced by distinctiveness
uncertainty_update_unchosen = q_value_uncertainty * (1 - variance_modulated_uncertainty_decay_rate) + target_unchosen_uncertainty * variance_modulated_uncertainty_decay_rate

q_value_uncertainty = jnp.where(
    unchosen_mask,
    uncertainty_update_unchosen,
    uncertainty_update_chosen,
)
q_value_uncertainty = jnp.clip(q_value_uncertainty, 0.0, 1.0)

# Compute perseveration bias, modulated by value uncertainty, recency, and prediction error.
# Higher uncertainty (Q-value closer to average) amplifies perseveration.
value_uncertainty = 1.0 - jax.nn.sigmoid(jnp.abs(q_values - average_reward_estimate) * 5.0)
recency_modulated_reward_sensitivity = perseveration_reward_sensitivity * recency_trace[choice]
adaptive_perseveration_bias_strength = perseveration_bias_strength * (1.0 + recency_modulated_reward_sensitivity * jax.nn.sigmoid(prediction_error))
base_perseveration_strength = perseveration_trace * adaptive_perseveration_bias_strength * (0.5 + 0.5 * recency_trace)
perseveration_bias = jax.nn.softplus(base_perseveration_strength * (1.0 + value_uncertainty))

```

bioRxiv preprint doi: https://doi.org/10.64898/2026.05.18.725921; this version posted May 21, 2026. The copyright holder for this preprint (which was not certified by peer review) is the author/funder, who has granted bioRxiv a license to display the preprint in perpetuity. It is made available under a CC-BY 4.0 International license.

```
relative_q_values = q_values - average_reward_estimate

# Dynamically adjust the inverse temperature based on overall Q-value uncertainty and reward variance.
# Higher uncertainty or variance leads to a lower effective inverse temperature (more exploration).
# Dynamically adjust the inverse temperature based on overall Q-value uncertainty, reward variance,
# AND the magnitude of uncertainty prediction error (UPE).
# High UPE magnitude indicates unreliability in uncertainty estimation, promoting more exploration (lower temperature).
upe_magnitude_scaling = 1.0 + jax.nn.softplus(jnp.abs(uncertainty_prediction_error_estimate) * 2.0) # Scales from 1.0 (low UPE) upwards
adaptive_temperature_scaling_factor = 1.0 - 0.5 * jax.nn.sigmoid(jnp.mean(q_value_uncertainty) * 5.0 + reward_variance_estimate * 5.0 - 5.0) * upe_magnitude_scaling
adaptive_temperature_scaling_factor = jnp.clip(adaptive_temperature_scaling_factor, 0.1, 2.0)
adaptive_inverse_temperature = inverse_temperature * adaptive_temperature_scaling_factor

# The option_specific_inverse_temperature now uses this adaptively scaled base temperature.
# It also incorporates Q-value variance for further scaling.
q_value_variance = jnp.var(q_values)
variance_scaling_factor = 0.5 + 1.5 * jax.nn.sigmoid(q_value_variance)
max_relative_q = jnp.max(relative_q_values)
volatility_and_uncertainty_scaling = 1.0 - 0.5 * jax.nn.sigmoid(reward_variance_estimate * 5.0 + jnp.mean(q_value_uncertainty) * 5.0 - 5.0)
volatility_and_uncertainty_scaling = jnp.clip(volatility_and_uncertainty_scaling, 0.1, 2.0)
option_specific_inverse_temperature = adaptive_inverse_temperature * variance_scaling_factor * (jax.nn.relu(1 + (relative_q_values - max_relative_q)) + 0.01) * volatility_and_uncertainty_scaling

# Recalculate Q-value entropy using the new adaptive_inverse_temperature for consistency.
q_value_softmax_probs = jax.nn.softmax(q_values * adaptive_inverse_temperature)
q_value_entropy = -jnp.sum(q_value_softmax_probs * jnp.log(q_value_softmax_probs + 1e-9))
normalized_entropy = q_value_entropy / jnp.log(4.0)
entropy_modulated_perseveration_factor = 0.5 + normalized_entropy

global_surprise_signal = jax.nn.sigmoid(jnp.abs(prediction_error) * 2.0)
global_surprise_perseveration_factor = 1.0 - 0.5 * global_surprise_signal
modulated_perseveration_bias = perseveration_bias * entropy_modulated_perseveration_factor * global_surprise_perseveration_factor

# Incorporate an exploration bonus based on Q-value uncertainty and reward variance.
exploration_bonus = jnp.mean(q_value_uncertainty) * jnp.mean(q_value_uncertainty)
option_exploration_bonus = (q_value_uncertainty + reward_variance_estimate) * exploration_bonus
# Add a general exploration bonus that doesn't rely on uncertainty, potentially encouraging more diverse choices.
# This bonus is higher for options that have not been chosen recently.
general_exploration_bonus = exploration_bonus_strength * (1.0 - recency_trace)
choice_logits = relative_q_values * option_specific_inverse_temperature + perseveration_trace * modulated_perseveration_bias + option_exploration_bonus + general_exploration_bonus

agent_state = jnp.concatenate([q_values, perseveration_trace, jnp.array([average_reward_estimate]), recency_trace, jnp.array([reward_variance_estimate]), q_value_uncertainty, jnp.array([uncertainty_prediction_error_estimate]), jnp.array([exploration_bonus_strength])])

```

bioRxiv preprint doi: https://doi.org/10.64898/2026.05.18.725921; this version posted May 21, 2026. The copyright holder for this preprint (which was not certified by peer review) is the author/funder, who has granted bioRxiv a license to display the preprint in perpetuity. It is made available under a CC-BY 4.0 International license.

```
  return choice_logits, agent_state
```

Code 2: Highest quality-of-fit program from the first independent Stage 1 AlphaEvolve run for the human bandit dataset.

A.1.7 Code: stage 2 ("Simplify") programs

```
def human_bandit_run1_simplified_low_floor(
    params: chex.Array,
    choice: int,
    reward: float,
    agent_state: Optional[chex.Array],
) -> tuple[chex.Array, chex.Array]:
  """
  This function models a reinforcement learning agent's decision-making and learning process.
  It updates its internal state based on a choice and its resulting reward, and then
  calculates the action preferences (logits) for the next decision.
  """

  # === 1. Unpack and Transform Model Parameters ===

  # The raw parameters are passed through a sigmoid function to constrain them to the range [0, 1].
  sigmoid_params = jax.nn.sigmoid(params[:7])

  # Assign the constrained parameters to informative variable names.
  initial_q_value = sigmoid_params[0]
  learning_rate = sigmoid_params[1]
  inverse_temperature = sigmoid_params[2]
  unchosen_learning_rate = sigmoid_params[3]
  perseveration_learning_rate = sigmoid_params[4]
  initial_perseveration_value = sigmoid_params[5]
  perseveration_bias_strength = sigmoid_params[6]

  # Scale some parameters to a more behaviorally meaningful range (e.g., [0, 10]).
  # Inverse temperature controls the exploration-exploitation trade-off.
  inverse_temperature_scaled = inverse_temperature * 10
  # Perseveration bias strength controls the tendency to repeat the last action.
  perseveration_bias_strength_scaled = perseveration_bias_strength * 10

  # === 2. Initialize or Unpack Agent's Internal State ===

  # If this is the first trial (agent_state is None), initialize the agent's internal
  # state with the initial parameter values. Otherwise, unpack the state from the previous trial.
  if agent_state is None:
    # Initialize Q-values (expected reward for each action) and perseveration trace.
    q_values = initial_q_value
    perseveration_trace = initial_perseveration_value
  else:
    # Unpack the Q-values (first 4 elements) and perseveration trace (next 4 elements).
    q_values = agent_state[:4]
    perseveration_trace = agent_state[4:8]

  # === 3. Update Q-values (Action Values) ===

  # This uses a Rescorla-Wagner (delta) learning rule.

  # Create a one-hot encoded vector to identify which action was chosen.
  chosen_action_mask = jax.nn.one_hot(choice, num_classes=4)
```

bioRxiv preprint doi: https://doi.org/10.64898/2026.05.18.725921; this version posted May 21, 2026. The copyright holder for this preprint (which was not certified by peer review) is the author/funder, who has granted bioRxiv a license to display the preprint in perpetuity. It is made available under a CC-BY 4.0 International license.

```

    # Calculate the prediction error: the difference between actual and expected
      reward.
    prediction_error = reward - q_values

    # Determine the learning rate for each action: a different rate is used for the
    # chosen action compared to the unchosen ones.
    learning_rates_for_all_actions = jnp.where(
        chosen_action_mask,
        learning_rate,            # Rate for the chosen action
        unchosen_learning_rate    # Rate for all other actions
    )

    # Update the Q-values by adding the prediction error, scaled by the learning rate
      .
    q_values = q_values + learning_rates_for_all_actions * prediction_error

    # === 4. Update Perseveration Trace ===

    # This trace models the agent's tendency to repeat recent actions.

    # For the chosen action, the trace is updated towards 1.
    updated_trace_for_chosen_action = (
        (1 - perseveration_learning_rate) * perseveration_trace
        + perseveration_learning_rate
    )

    # Update the full perseveration trace array: apply the update for the chosen
    # action and reset the trace to 0.0 for all unchosen actions.
    perseveration_trace = jnp.where(
        chosen_action_mask,
        updated_trace_for_chosen_action,
        0.0,
    )

    # === 5. Calculate Action Preferences (Logits) ===

    # The agent's final choice preference is a combination of learned values and
      perseveration bias.

    # Calculate the component of choice preference driven by the learned Q-values.
    value_component = q_values * inverse_temperature_scaled

    # Calculate the component of choice preference driven by the perseveration trace.
    perseveration_component = perseveration_trace *
      perseveration_bias_strength_scaled

    # The final choice logits are the sum of the two components.
    # A softmax function is typically applied to these logits to get choice
      probabilities.
    choice_logits = value_component + perseveration_component

    # === 6. Prepare the New Agent State for the Next Trial ===

    # Concatenate the updated Q-values and perseveration trace into a single array
    # to be passed as the agent_state in the next iteration.
    agent_state = jnp.concatenate([q_values, perseveration_trace])

    # Return the calculated logits and the updated state.
    return choice_logits, agent_state
```

Code 3: Lowest-complexity program from Stage 2 AlphaEvolve run with 50% threshold for the human bandit dataset, evolved from programs in the first independent Stage 1 AlphaEvolve run and rewritten for readability (Stage 3).

```
def human_bandit_run2_simplified_medium_floor(
    params: chex.Array,
```

bioRxiv preprint doi: https://doi.org/10.64898/2026.05.18.725921; this version posted May 21, 2026. The copyright holder for this preprint (which was not certified by peer review) is the author/funder, who has granted bioRxiv a license to display the preprint in perpetuity. It is made available under a CC-BY 4.0 International license.

```
    choice: int,
    reward: float,
    agent_state: Optional[chex.Array],
) -> tuple[chex.Array, chex.Array]:
  """Cognitive model describing human behavior on a multi-armed bandit task.

  Args:
    params: Fit parameters of the model. Different parameters are used for
      different subjects.
    choice: Choice made by the agent on the previous trial. 0, 1, 2, or 3
    reward: Reward received by the agent on the previous trial. A float between
      0 and 1.
    agent_state: The current state of the cognitive model.

  Returns:
    choice_logits: The probabilities that the agent will choose option 0, 1, 2,
      or 3 on the next trial, expressed as logits.
    agent_state: New agent state
  """
  # --- 1. Unpack and transform model parameters ---

  # Apply a sigmoid function to raw parameters to constrain them between 0 and 1.
  (
      learning_rate,
      initial_q_value,
      choice_trace_decay_rate,
      stickiness,
      chosen_action_decay_rate,
      uncertainty_weight,
      background_learning_rate,
      uncertainty_learning_rate,
      uncertainty_initial_value,
      inverse_temperature,
  ) = jax.nn.sigmoid(params[:10])

  # Scale the inverse_temperature (softmax temperature) for a wider dynamic range.
  inverse_temperature = inverse_temperature * 10.0

  # --- 2. Initialize agent state on the first trial ---

  if agent_state is None:
    # The agent state is a vector containing different cognitive variables.
    # It is initialized here if this is the very first trial.
    initial_q_values = jnp.full(4, initial_q_value)
    initial_uncertainty_values = jnp.full(4, uncertainty_initial_value)
    initial_choice_traces = jnp.zeros(4)
    initial_reward_background = jnp.array([0.5])  # Starts at a neutral 0.5

    agent_state = jnp.concatenate([
        initial_q_values,
        initial_uncertainty_values,
        initial_choice_traces,
        initial_reward_background,
    ])

  # --- 3. Unpack the agent's current state into meaningful variables ---

  # The state vector is split into its constituent parts.
  q_values, uncertainty_values, choice_traces, reward_background_expectation = jnp.
     split(
      agent_state, [4, 8, 12]
  )

  # --- 4. Update Q-values (action value estimates) based on the reward ---

  # Calculate the prediction error: the difference between the received reward
  # and the expected reward for the chosen action.
  prediction_error = reward - q_values[choice]
```

bioRxiv preprint doi: https://doi.org/10.64898/2026.05.18.725921; this version posted May 21, 2026. The copyright holder for this preprint (which was not certified by peer review) is the author/funder, who has granted bioRxiv a license to display the preprint in perpetuity. It is made available under a CC-BY 4.0 International license.

```

# Update the Q-value of the chosen action using the prediction error and learning
  rate.
# This is a standard Rescorla-Wagner update rule.
updated_q_value_for_chosen_action = (
    q_values[choice] + learning_rate * prediction_error
)
q_values = q_values.at[choice].set(updated_q_value_for_chosen_action)

# Create a one-hot vector to easily apply updates to chosen vs. unchosen actions.
chosen_one_hot = jax.nn.one_hot(choice, num_classes=4)
unchosen_mask = 1.0 - chosen_one_hot

# Update the Q-values of the *unchosen* actions. This represents a form of
# background learning or generalization, where unchosen options drift towards
# a modified background expectation.
background_q_value_update_term = background_learning_rate * (
    (reward_background_expectation - prediction_error) - q_values
)
q_values = q_values + unchosen_mask * background_q_value_update_term

# --- 5. Update uncertainty estimates ---

# The update for the chosen action's uncertainty depends on the absolute
  prediction error.
# A large surprise (positive or negative) increases uncertainty about that action
  .
chosen_uncertainty_update = uncertainty_learning_rate * (
    jnp.abs(prediction_error) - uncertainty_values
)

# The uncertainty of unchosen actions increases by a small, fixed amount.
# This models a general increase in uncertainty for options not being sampled.
unchosen_uncertainty_update = 0.01

# Apply the respective updates to the chosen and unchosen actions' uncertainties.
uncertainty_values = uncertainty_values + (
    chosen_one_hot * chosen_uncertainty_update
) + (
    unchosen_mask * unchosen_uncertainty_update
)

# --- 6. Update choice traces to model perseveration/stickiness ---

# Create a vector of decay rates. The chosen action has a different decay rate
# than the unchosen actions.
decay_rates = jnp.full_like(choice_traces, choice_trace_decay_rate)
decay_rates = decay_rates.at[choice].set(chosen_action_decay_rate)

# Apply decay to all traces, then add 1 to the trace of the chosen action.
# This makes recently chosen actions more likely to be chosen again.
choice_traces = choice_traces * (1.0 - decay_rates) + chosen_one_hot

# --- 7. Update the background reward expectation ---

# This is a running average of the rewards received, updated with its own
  learning rate.
reward_background_expectation = reward_background_expectation + (
    background_learning_rate * (reward - reward_background_expectation)
)

# --- 8. Re-assemble the new agent state vector ---

# Concatenate the updated cognitive variables back into a single state vector.
agent_state = jnp.concatenate(
    (q_values, uncertainty_values, choice_traces, reward_background_expectation)
)

```

bioRxiv preprint doi: https://doi.org/10.64898/2026.05.18.725921; this version posted May 21, 2026. The copyright holder for this preprint (which was not certified by peer review) is the author/funder, who has granted bioRxiv a license to display the preprint in perpetuity. It is made available under a CC-BY 4.0 International license.

```
  # --- 9. Calculate choice logits for the next action ---

  # The effective temperature is modulated by uncertainty. Higher uncertainty
    reduces
  # the influence of the Q-values, making choices more random (exploratory).
  effective_inverse_temperature = inverse_temperature / (
      1.0 + uncertainty_weight * uncertainty_values
  )

  # The final logits are a weighted sum of two components:
  # 1. The value-based component (Q-values scaled by effective temperature).
  value_component = effective_inverse_temperature * q_values
  # 2. The stickiness component (choice traces encouraging perseveration).
  stickiness_component = stickiness * choice_traces

  choice_logits = value_component + stickiness_component

  # --- 10. Return the results ---

  return choice_logits, agent_state
```

Code 4: Lowest-complexity program from Stage 2 AlphaEvolve run with 75% threshold for the human bandit dataset, evolved from programs in the second independent Stage 1 AlphaEvolve run and rewritten for readability (Stage 3).

```
def human_bandit_run1_simplified_high_floor(
    params: chex.Array,
    choice: int,
    reward: float,
    agent_state: Optional[chex.Array],
) -> tuple[chex.Array, chex.Array]:
  """Cognitive model describing human behavior on a multi-armed bandit task.

  Assumes the agent is presented with four options on each trial.

  Args:
    params: Fit parameters of the model. Different parameters are used for
      different subjects.
    choice: Choice made by the agent on the previous trial. 0, 1, 2, or 3
    reward: Reward received by the agent on the previous trial. A float between
      0 and 1.
    agent_state: The current state of the cognitive model.

  Returns:
    choice_logits: The probabilities that the agent will choose option 0, 1, 2,
      or 3 on the next trial, expressed as logits.
    agent_state: New agent state
  """

  # --- 1. Unpack Model Parameters ---
  # These parameters are fixed for a given agent and are learned from data.
  # They are transformed using sigmoid or other functions to constrain their range.

  # The initial belief about the value of each option, used for initialization and
    as a decay target.
  initial_q_value = jax.nn.sigmoid(params[0])

  # Learning rate for the Q-value of the *chosen* option.
  learning_rate = jax.nn.sigmoid(params[1])

  # The base inverse temperature for the softmax choice rule, controlling the level
    of exploration.
  inverse_temperature = 10 * jax.nn.sigmoid(params[2])

  # Learning rate for unchosen options, causing them to decay towards a prior.
  unchosen_learning_rate = 0.95 * jax.nn.sigmoid(params[3]) + 0.025

```

bioRxiv preprint doi: https://doi.org/10.64898/2026.05.18.725921; this version posted May 21, 2026. The copyright holder for this preprint (which was not certified by peer review) is the author/funder, who has granted bioRxiv a license to display the preprint in perpetuity. It is made available under a CC-BY 4.0 International license.

```
    # Learning rate for the perseveration trace of the *chosen* option.
    perseveration_learning_rate = jax.nn.sigmoid(params[4])

    # Decay rate for the perseveration traces of *unchosen* options.
    perseveration_decay_rate = jax.nn.sigmoid(params[5])

    # The initial value of the perseveration trace for each option.
    initial_perseveration_value = jax.nn.sigmoid(params[6])

    # The strength of the perseveration bias, influencing how much the perseveration trace affects choices.
    perseveration_bias_strength = jax.nn.sigmoid(params[7]) * 10.0

    # How sensitive the perseveration bias is to the reward outcome.
    perseveration_reward_sensitivity = jax.nn.sigmoid(params[8])


    # --- 2. Initialize or Unpack Agent State ---
    # The agent state holds dynamic variables that are updated on each trial.
    if agent_state is None:
      # If this is the first trial, initialize all state variables.
      num_options = 4
      q_values = jnp.full(num_options, initial_q_value)
      perseveration_trace = jnp.full(num_options, initial_perseveration_value)
      average_reward_estimate = initial_q_value
      recency_trace = jnp.zeros(num_options)
    else:
      # Otherwise, unpack the state from the previous trial.
      q_values = agent_state[0:4]
      perseveration_trace = agent_state[4:8]
      average_reward_estimate = agent_state[8]
      recency_trace = agent_state[9:13]


    # --- 3. Update Q-Values (Action Values) ---

    # 3.1. First, apply a global decay to all Q-values, pulling them towards the initial prior.
    # This represents a forgetting or diffusion process.
    q_values = q_values * (1 - unchosen_learning_rate) + initial_q_value * unchosen_learning_rate

    # 3.2. Update the running estimate of the average reward in the environment.
    # This estimate acts as a baseline for evaluating individual options.
    reward_prediction_error_avg = reward - average_reward_estimate
    avg_reward_error_magnitude_factor = 1.0 + jax.nn.softplus(jnp.abs(reward_prediction_error_avg))
    effective_avg_reward_learning_rate = unchosen_learning_rate * avg_reward_error_magnitude_factor
    average_reward_estimate += effective_avg_reward_learning_rate * reward_prediction_error_avg

    # 3.3. Update the Q-value for the *chosen* option based on the reward received.
    # This is a standard Rescorla-Wagner update rule.
    prediction_error = reward - q_values[choice]
    updated_chosen_q_value = q_values[choice] + learning_rate * prediction_error
    q_values = q_values.at[choice].set(updated_chosen_q_value)

    # 3.4. Update the Q-values for the *unchosen* options.
    # These values decay towards the average reward estimate, allowing them to track the
    # overall richness of the environment. The decay rate is modulated by "global surprise."
    global_surprise_factor = 1.0 + jax.nn.softplus(jnp.abs(prediction_error))
    unchosen_inferred_prediction_error = reward - q_values
    unchosen_surprise_factor = 1.0 + jax.nn.softplus(jnp.abs(unchosen_inferred_prediction_error))
```

bioRxiv preprint doi: https://doi.org/10.64898/2026.05.18.725921; this version posted May 21, 2026. The copyright holder for this preprint (which was not certified by peer review) is the author/funder, who has granted bioRxiv a license to display the preprint in perpetuity. It is made available under a CC-BY 4.0 International license.

```
effective_unchosen_learning_rate = unchosen_learning_rate * global_surprise_factor * unchosen_surprise_factor

updated_unchosen_q_values = (
    q_values * (1 - effective_unchosen_learning_rate) +
    average_reward_estimate * effective_unchosen_learning_rate
)

# Create a mask to apply updates only to unchosen options.
unchosen_mask = jnp.arange(4) != choice
q_values = jnp.where(
    unchosen_mask,
    updated_unchosen_q_values,
    q_values # Keep the already-updated chosen Q-value.
)


# --- 4. Update Perseveration Trace ---
# This trace captures the tendency to repeat previous choices (habit).

# 4.1. For the *chosen* option, increase the perseveration trace towards 1.
# The update is stronger for positive prediction errors (unexpected rewards).
chosen_perseveration_learning_rate = perseveration_learning_rate * jax.nn.sigmoid(prediction_error * 5)
perseveration_trace_update_chosen = perseveration_trace[choice] + chosen_perseveration_learning_rate * (1 - perseveration_trace[choice])

# 4.2. For *unchosen* options, decay the perseveration trace.
# The decay rate is increased by several factors reflecting environmental uncertainty and surprise.
unchosen_relative_badness_factor = jax.nn.sigmoid(q_values - reward)
global_average_reward_context_factor = 1.0 + jax.nn.relu(average_reward_estimate - 0.5)
global_outcome_surprise_factor = 1.0 + jax.nn.softplus(jnp.abs(prediction_error))

decay_multiplier = (
    1.0 + unchosen_relative_badness_factor +
    global_average_reward_context_factor + global_outcome_surprise_factor
)
effective_perseveration_decay_unchosen = jnp.clip(perseveration_decay_rate * decay_multiplier, 0.0, 1.0)

perseveration_trace_update_unchosen = (
    perseveration_trace * (1 - effective_perseveration_decay_unchosen) +
    initial_perseveration_value * effective_perseveration_decay_unchosen
)

# 4.3. Combine the updates for the chosen and unchosen options.
perseveration_trace = jnp.where(
    unchosen_mask,
    perseveration_trace_update_unchosen,
    perseveration_trace_update_chosen,
)


# --- 5. Update Recency Trace ---
# This trace captures more general recency effects.

# 5.1. For the *chosen* option, increase the recency trace towards 1.
# The update is scaled by the prediction error.
recency_update_factor = 1.0 + prediction_error
reward_modulated_recency_learning_rate = perseveration_learning_rate * recency_update_factor
recency_trace_update_chosen = recency_trace[choice] + reward_modulated_recency_learning_rate * (1 - recency_trace[choice])

# 5.2. For *unchosen* options, decay the recency trace.
# The decay is slower for options that had Q-values similar to the chosen one.
```

bioRxiv preprint doi: https://doi.org/10.64898/2026.05.18.725921; this version posted May 21, 2026. The copyright holder for this preprint (which was not certified by peer review) is the author/funder, who has granted bioRxiv a license to display the preprint in perpetuity. It is made available under a CC-BY 4.0 International license.

```
q_value_similarity_to_chosen = jax.nn.sigmoid(5.0 * (1.0 - jnp.abs(q_values - q_values[choice])))
recency_decay_scaling_factor = 1.0 - 0.9 * q_value_similarity_to_chosen
effective_recency_decay_rate = perseveration_decay_rate * recency_decay_scaling_factor
recency_trace_update_unchosen = recency_trace * (1 - effective_recency_decay_rate )

# 5.3. Combine the updates for the chosen and unchosen options.
recency_trace = jnp.where(
    unchosen_mask,
    recency_trace_update_unchosen,
    recency_trace_update_chosen,
)


# --- 6. Compute Choice Logits ---
# Combine the learned values and biases to determine the choice probabilities for the next trial.

# 6.1. Compute an adaptive perseveration bias.
# This bias is modulated by value uncertainty, recency, and reward sensitivity.
value_uncertainty = 1.0 - jax.nn.sigmoid(jnp.abs(q_values - average_reward_estimate) * 5.0)
adaptive_perseveration_bias_strength = perseveration_bias_strength * (1.0 + perseveration_reward_sensitivity * prediction_error)
base_perseveration_strength = perseveration_trace * adaptive_perseveration_bias_strength * (0.5 + 0.5 * recency_trace)
perseveration_bias = jax.nn.softplus(base_perseveration_strength * (1.0 + value_uncertainty))

# 6.2. Compute relative Q-values by centering them around the average reward estimate.
# This makes the choice dependent on how much better an option is than the environment's average.
relative_q_values = q_values - average_reward_estimate

# 6.3. Compute an option-specific inverse temperature.
# This makes the choice policy more exploitative when value estimates are more certain (higher variance).
q_value_variance = jnp.var(q_values)
variance_scaling_factor = 0.5 + 1.5 * jax.nn.sigmoid(q_value_variance)
option_specific_inverse_temperature = inverse_temperature * variance_scaling_factor * (jax.nn.relu(1 + relative_q_values) + 0.01)

# 6.4. Combine the value-based and habit-based components to get the final choice logits.
# This is a hybrid model where choice is driven by both goal-directed values and habitual perseveration.
choice_logits = (
    relative_q_values * option_specific_inverse_temperature +
    perseveration_trace * perseveration_bias
)


# --- 7. Assemble New Agent State for the Next Trial ---
agent_state = jnp.concatenate([
    q_values,
    perseveration_trace,
    jnp.array([average_reward_estimate]),
    recency_trace
])
```

bioRxiv preprint doi: https://doi.org/10.64898/2026.05.18.725921; this version posted May 21, 2026. The copyright holder for this preprint (which was not certified by peer review) is the author/funder, who has granted bioRxiv a license to display the preprint in perpetuity. It is made available under a CC-BY 4.0 International license.

```
  return choice_logits, agent_state
```

Code 5: Lowest-complexity program from Stage 2 AlphaEvolve run with 90% threshold for the human bandit dataset, evolved from programs in the first independent Stage 1 AlphaEvolve run and rewritten for readability (Stage 3).

A.1.8 Additional figures

bioRxiv preprint doi: https://doi.org/10.64898/2026.05.18.725921; this version posted May 21, 2026. The copyright holder for this preprint (which was not certified by peer review) is the author/funder, who has granted bioRxiv a license to display the preprint in perpetuity. It is made available under a CC-BY 4.0 International license.

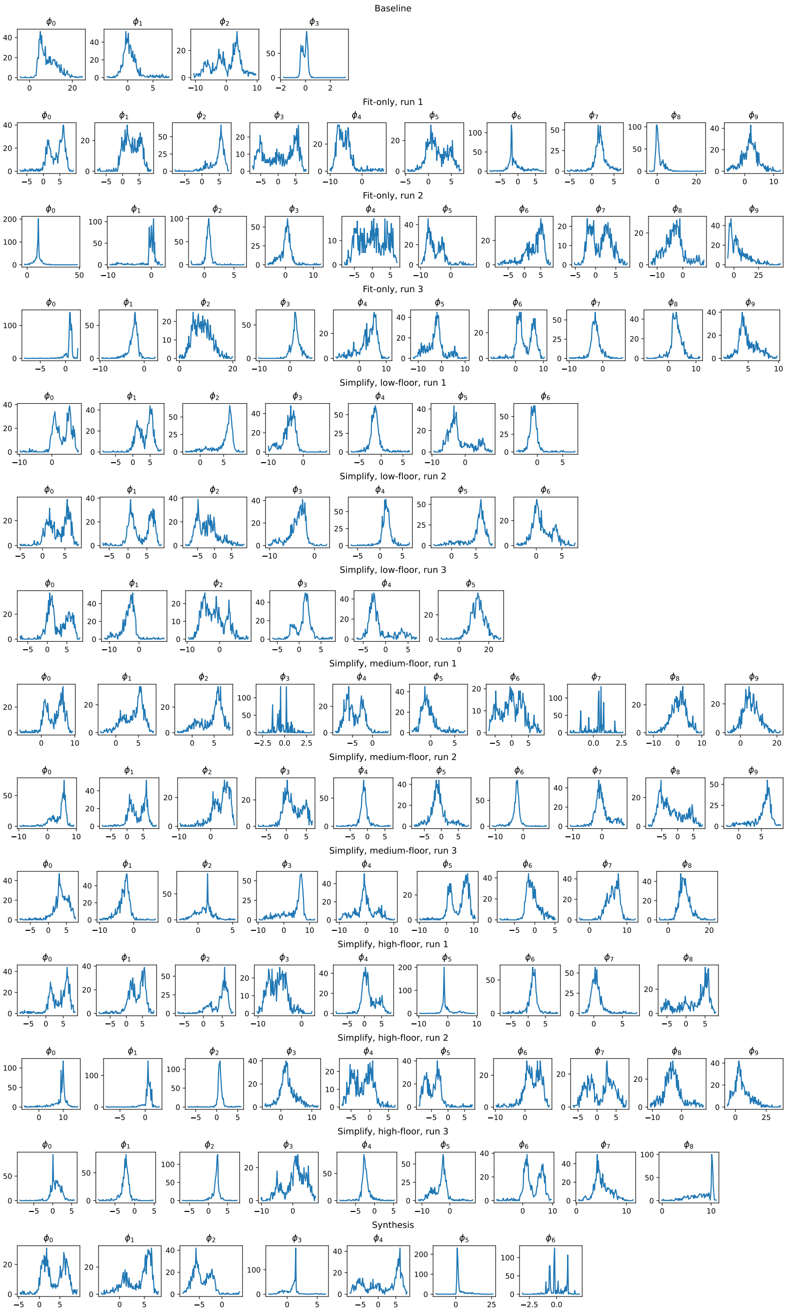

Fig. A1: ***Human Bandit* Dataset: Fit parameters for each program**. The distribution of fit parameters for each fold of all discovered programs (fit-only and simplified), as well as the handcrafted baseline and synthesis program.

bioRxiv preprint doi: https://doi.org/10.64898/2026.05.18.725921; this version posted May 21, 2026. The copyright holder for this preprint (which was not certified by peer review) is the author/funder, who has granted bioRxiv a license to display the preprint in perpetuity. It is made available under a CC-BY 4.0 International license.

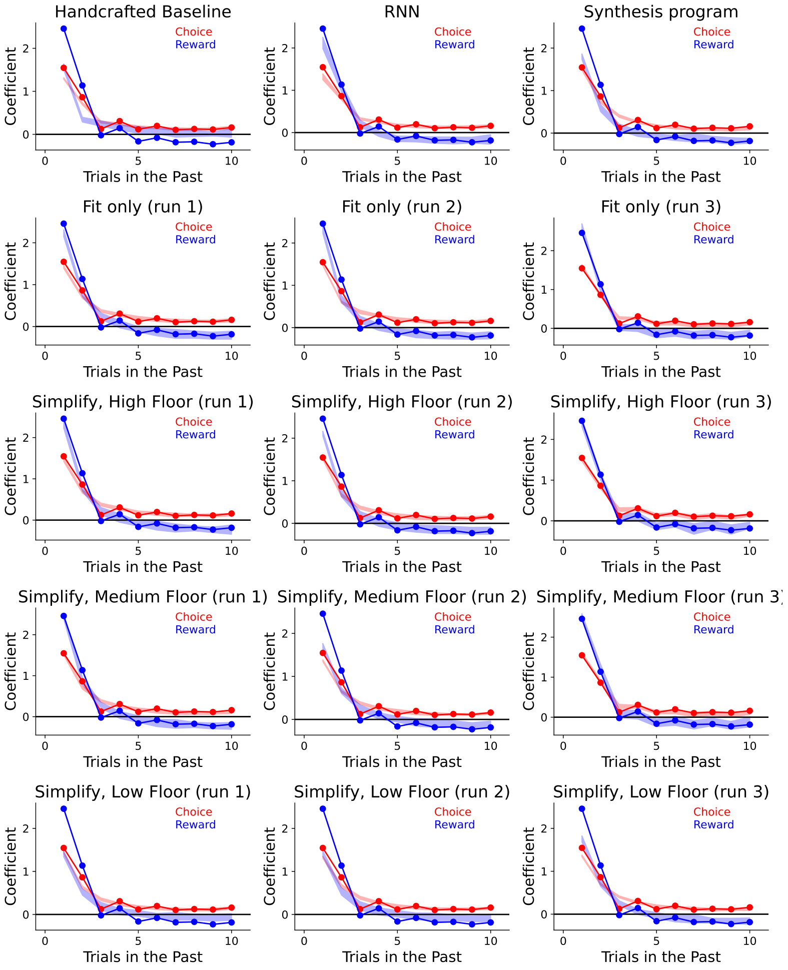

Fig. A2: ***Human Bandit* Dataset: Trial-lagged regression analyses**. Here we see the trial-lagged regression analysis shown in Figure 6 for all discovered programs for this dataset. The coefficients for the real data are shown in solid lines, while the transparent patch shows the 95% prediction interval for the artificial data. We see that most discovered models capture the trial-lagged regression statistics better than the handcrafted model, and that programs obtained with higher floors tend to provide a tighter match. This demonstrates that the evolved programs are strong generative models.

bioRxiv preprint doi: https://doi.org/10.64898/2026.05.18.725921; this version posted May 21, 2026. The copyright holder for this preprint (which was not certified by peer review) is the author/funder, who has granted bioRxiv a license to display the preprint in perpetuity. It is made available under a CC-BY 4.0 International license.

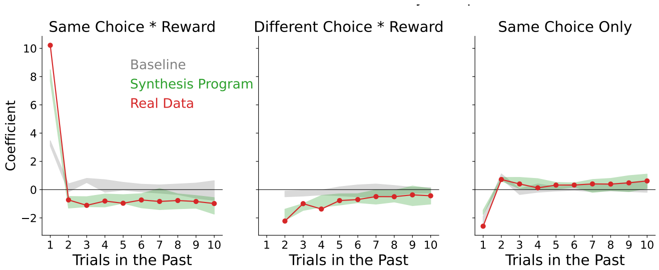

Fig. A3: ***Human Bandit* Dataset: Trial-lagged regression coefficients for tendency to repeat previous choice.**. Trial-lagged regression analysis showing the influence (coefficient, positive promotes repeating) of previous trials (1-10 trials ago) on tendency to repeat the agent's current choice. The The leftmost plot ("Same Choice * Reward") shows the influence of being rewarded in the past for choosing the same action the subject is considering repeating; the middle plot ("Different Choice * Reward") shows the influence of being rewarded in the past for choosing a *different* action from the one the subject is considering repeating; the rightmost plot shows the influence of having performed the same action in the past, regardless of reward.

bioRxiv preprint doi: https://doi.org/10.64898/2026.05.18.725921; this version posted May 21, 2026. The copyright holder for this preprint (which was not certified by peer review) is the author/funder, who has granted bioRxiv a license to display the preprint in perpetuity. It is made available under a CC-BY 4.0 International license.

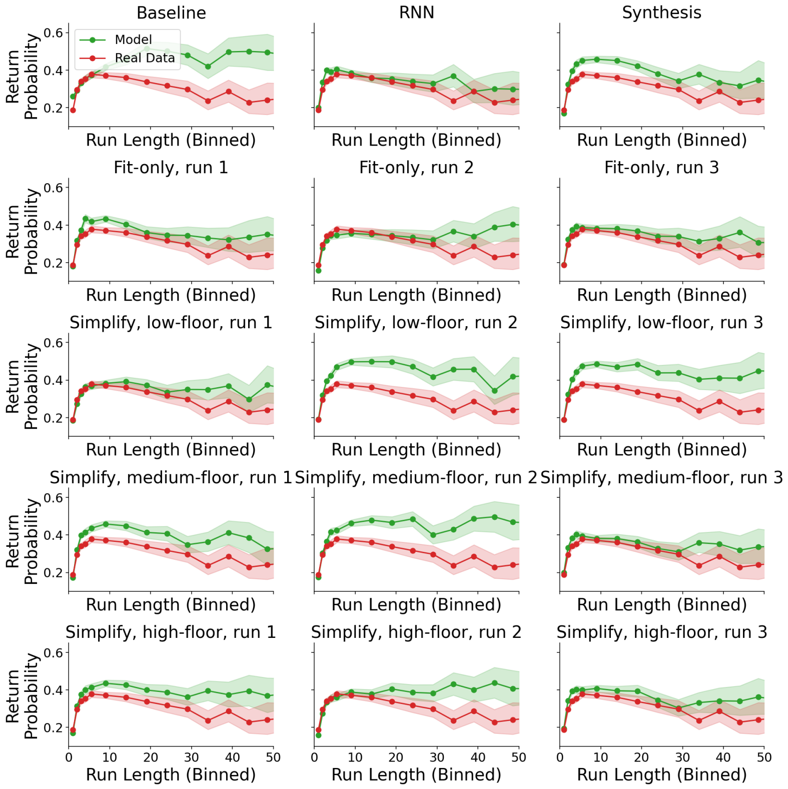

Fig. A4: ***Human Bandit* Dataset: Run Return Probability analysis for all models.**. Here we see the probability of returning to a run of repeated choices, for runs of different length, after a single different choice. Shaded intervals depict 95% confidence intervals.

bioRxiv preprint doi: https://doi.org/10.64898/2026.05.18.725921; this version posted May 21, 2026. The copyright holder for this preprint (which was not certified by peer review) is the author/funder, who has granted bioRxiv a license to display the preprint in perpetuity. It is made available under a CC-BY 4.0 International license.

A.2 Rat Bandit

A.2.1 Dataset

We consider the behavioral dataset from Miller et al. [8], in which rats perform a two-alternative reward-learning task with binary rewards. Rats indicated their choice on each trial by entering one of two available nose ports, which were equipped to deliver small liquid rewards. Reward probabilities followed independent bounded random walks. Rats performed daily sessions of approximately one hour. The dataset contains choices from 20 rats performing 1,946 total sessions and 1,087,140 total trials.

We obtained this dataset from the following URL, where it is freely available under a permissive open-source license:

https://figshare.com/articles/dataset/From_predictive_models_to_cognitive_models_Separable_behavioral_processes_underlying_reward_learning_in_the_rat/20449356

A.2.2 Baseline Model

Miller et al. [8] performed an intensive human process of data-driven model discovery on this dataset. This resulted in a model that we refer to as "Reward-Seeking/Habit/Gambler-Fallacy" (RHG), which we adopt as the handcrafted baseline model for this dataset. For compatibility with our pipeline, we re-implemented this model in jax.

A.2.3 Discussion of evolved programs

One fit-only seed achieved a much higher performance on the training set than the others. Because the floors for simplifying were based on the difference between this best fit-only program and the baseline program, this meant that the highest floor was actually higher than the fit-only performance on the training set for the other two seeds. As a result, there was only one high-floor simplify program for this dataset.

We therefore focused our analysis on the 6 medium- and low-floor programs, of which we had three each. Medium-floor programs had similar scores to one another on the held-out subjects, and similar scores to the fit-only programs from the two lower-scoring seeds. This indicates that the simplifications done did not substantially impact held-out performance. Low-floor programs had substantially worse scores, indicating that the simplifications done to get to this floor did substantially impact held-out performance.

Cognitive variables

All simplified programs defined two sets of variables for fast and slow reward-dependent learning processes respectively. Some programs (3/6 low- and medium-floor programs) also defined action perservation terms that encouraged actions to be repeated independent of whether they had been rewarding. This particular decomposition of cognitive variables was present in the handcrafted baseline model as well, although the particular discovered updates on the terms had slightly different forms and added nonlinearities.

bioRxiv preprint doi: https://doi.org/10.64898/2026.05.18.725921; this version posted May 21, 2026. The copyright holder for this preprint (which was not certified by peer review) is the author/funder, who has granted bioRxiv a license to display the preprint in perpetuity. It is made available under a CC-BY 4.0 International license.

Fast reward-guided learning

The fast learning modules often implemented a "linked" Q-learning variant in which the prediction error $\delta = r - Q(c)$ given the chosen action value was used to update both the chosen and the unchosen Q-values (3/6 programs). The chosen and unchosen action values were updated with different learning rates but otherwise antisymmetrically, with updates $Q(c) \leftarrow Q(c) + \phi_i \delta$ and $Q(c') \leftarrow Q(c') - \phi_j \delta$. We call this linked because the update on the chosen and unchosen action values are linked. Other discovered programs found more complex reward-guided learning.

Slow learning of non-rewarding options

The slow learning modules often exhibited a pattern wherein it accumulated recent non-rewarding choices and ignored rewarding choices. This had the effect of driving choices *toward* choices from which rewards had been absent. This pattern was evident in the baseline RHG model as well, and was referred to as the "Gambler's Fallacy" term. Gambler's Fallacy refers to an often mistaken belief that options that have not returned rewards recently are due to payoff soon.

Mapping cognitive variables onto behavior

One discovered model exhibited the nonlinear pattern of behavior present in the synthesis program. However, this nonlinearity contributed substantially to behavior performance: across the medium-floor models, it is the simplest while maintaining the median quality-of-fit. For each slow learning value x and fast learning values y, the particular nonlinearity is ye^x.

Programs also exhibited a "win-stay/lose-shift" contribution to behavior. This does not reflect "learning" *per se*, as it does not necessitate updating cognitive variables; rather, a bonus is applied to the previous action that depends on the reward.

A.2.4 Discussion of synthesis program

The synthesis program kept the linked Q-learning rule that appeared in 3/6 programs, as this was the most common learning rule to emerge and fit as well as the other more complex rules. The term tracking the slow learning of non-rewarding options was also included, as was the nonlinear rule for combining the fast and slow learning terms and the one-back win-stay/lose-switch bias, as these all contributed positively to behavior. Its slightly improved simplicity over the other medium-floor programs comes from the removal of terms that were revealed to not contribute to score.

A.2.5 Code: stage 2 ("Simplify") programs

```
def rat_bandit_run1_simplified_medium_floor(
    params: chex.Array,
    choice: int,
    reward: int,
    agent_state: Optional[chex.Array],
) -> tuple[chex.Array, chex.Array]:
  """Cognitive model describing rat behavior on a binary two-armed bandit task.

  Args:
```

bioRxiv preprint doi: https://doi.org/10.64898/2026.05.18.725921; this version posted May 21, 2026. The copyright holder for this preprint (which was not certified by peer review) is the author/funder, who has granted bioRxiv a license to display the preprint in perpetuity. It is made available under a CC-BY 4.0 International license.

Table A2: Evaluation performance and program complexity for models in the *Rat Bandit* dataset. For programs generated by the "Simplify" stage, Floor represents the quality-of-fit threshold below which programs are discarded (see Section 7.6.2). Score indicates the average normalized likelihood across evaluation subjects (see Section 7.3); Effort is Halstead effort. State, Params, and Lines indicate the number of state variables, per-subject parameters, and lines of code respectively.

Model type	Floor	Run	Score	Effort	State	Params	Lines
Handcrafted Baseline	–	–	0.6677	18,564	3	7	76
RNN Baseline	–	–	0.6747	–	–	–	–
Stage 1: "Maximize Quality-of-Fit"	–	1	0.6723	48,457	7	10	77
		2	0.6739	227,980	8	10	176
		3	0.6720	53,138	8	10	69
Stage 2: "Simplify"	50%	1	0.6704	14,968	4	10	119
		2	0.6701	17,773	6	5	119
		3	0.6692	20,356	6	10	131
	75%	1	0.6718	13,039	4	5	121
		2	0.6721	25,175	6	10	145
		3	0.6716	19,378	8	5	119
	90%	1	0.6732	62,403	6	10	173
Synthesis Program	–	–	0.6713	11,891	4	7	59

```
    params: Model params. Different parameters are used for different rats.
    choice: Previous choice. Values: 0 or 1.
    reward: Previous reward. Values: 0 or 1.
    agent_state: Previous state of the agent

  Returns:
    choice_logits: Vector of shape (2,) with the probabilities that the rat will
      choose option 0 or 1 on the next trial, expressed as logits.
    agent_state: New state of the agent, after observing the previous choice and
      reward.
  """
  # --- 1. Initialization and Parameter Unpacking ---

  # On the first trial (when agent_state is None), initialize the state.
  # The state consists of [Q-value for choice 0, Q-value for choice 1,
  #                        Recency for choice 0, Recency for choice 1].
  # Q-values are initialized to 0.5 (neutral), recency effects to 0.0.
  if agent_state is None:
    agent_state = jnp.array([0.5, 0.5, 0.0, 0.0])

  # Unpack the model's parameters from the input array.
  log_beta, *sigmoid_params, bias_choice_0, recency_win_weight, recency_loss_weight
     = params

  # Apply the sigmoid function to constrain certain parameters to be between 0 and
     1.
  # This is a standard method for parameters representing rates or probabilities.
  (
      alpha_chosen,           # Learning rate for the chosen option.
      alpha_unchosen,         # Learning rate for the unchosen option.
      persistence_rewarded,   # Weight for repeating a rewarded choice.
      persistence_unrewarded,# Weight for repeating an unrewarded choice.
      forgetting_rate,        # Rate at which Q-values decay over time.
```

bioRxiv preprint doi: https://doi.org/10.64898/2026.05.18.725921; this version posted May 21, 2026. The copyright holder for this preprint (which was not certified by peer review) is the author/funder, who has granted bioRxiv a license to display the preprint in perpetuity. It is made available under a CC-BY 4.0 International license.

```
    recency_decay_rate,    # Rate at which recency effects decay over time.
) = map(jax.nn.sigmoid, sigmoid_params)

# Decompose the agent_state vector into its meaningful components.
previous_q_values = agent_state[:2]
previous_recency_effects = agent_state[2:4]

# --- 2. Update Q-Values (Reinforcement Learning) ---

# Apply a forgetting factor to the previous Q-values.
decayed_q_values = previous_q_values * (1.0 - forgetting_rate)

# Calculate the prediction error: the difference between the actual reward
# and the expected reward (the Q-value of the chosen option).
prediction_error = reward - decayed_q_values[choice]

# Update the Q-value for the chosen option using the prediction error.
# Note: JAX arrays are immutable, so .at[].add() creates a new array.
updated_q_values = decayed_q_values.at[choice].add(
    alpha_chosen * prediction_error
)
# Update the Q-value for the unchosen option. This model assumes it is
# updated in the opposite direction of the chosen option's error.
updated_q_values = updated_q_values.at[1 - choice].add(
    alpha_unchosen * -prediction_error
)

# --- 3. Update Recency Effects ---

# Decay the previous recency effects.
decayed_recency_effects = previous_recency_effects * recency_decay_rate

# Determine the "recency shock" from the last outcome.
# A reward (reward=1) adds recency_win_weight, a non-reward (reward=0)
# adds -recency_loss_weight, modeling a win-stay/lose-shift tendency.
recency_update_value = jnp.array([-recency_loss_weight, recency_win_weight])[
  reward]
scaled_recency_update = recency_update_value * (1.0 - recency_decay_rate)

# Apply this update to the recency trace of the chosen option.
updated_recency_effects = decayed_recency_effects.at[choice].add(
    scaled_recency_update
)

# --- 4. Calculate Choice Logits for the Next Trial ---

# The logits determine the probability of the next choice. They are a sum
# of several cognitive components: value, bias, recency, and persistence.

# Convert log_beta to beta, the inverse temperature parameter that controls
# the level of determinism in the choice. Higher beta means less random choices.
beta = jnp.exp(log_beta)

# Component 1: Value-based term (RL).
# The Q-values are modulated by an exponential of the recency effects.
value_signal = updated_q_values * jnp.exp(updated_recency_effects)
# A static bias towards choice 0 is added to the value signal.
value_signal_with_bias = value_signal.at[0].add(bias_choice_0)
# This entire value component is then scaled by beta.
rl_component = beta * value_signal_with_bias

# Component 2: Persistence and Recency term.
# A persistence bonus is calculated for the action that was just taken.
# The size of the bonus depends on whether the action was rewarded.
persistence_bonus = reward * persistence_rewarded - persistence_unrewarded
# This bonus is added to the recency effect of the chosen action.
recency_and_persistence_component = updated_recency_effects.at[choice].add(
    persistence_bonus
```

bioRxiv preprint doi: https://doi.org/10.64898/2026.05.18.725921; this version posted May 21, 2026. The copyright holder for this preprint (which was not certified by peer review) is the author/funder, who has granted bioRxiv a license to display the preprint in perpetuity. It is made available under a CC-BY 4.0 International license.

```
  )

  # The final choice logits are the sum of the two main components.
  choice_logits = rl_component + recency_and_persistence_component

  # --- 5. Prepare Outputs ---

  # Construct the new agent state for the next trial by combining the
  # updated Q-values and recency effects into a single array.
  new_agent_state = jnp.concatenate([updated_q_values, updated_recency_effects])

  return choice_logits, new_agent_state
```

Code 6: Lowest-complexity program from Stage 2 AlphaEvolve run with 75% threshold for the rat bandit dataset, evolved from programs in the first independent Stage 1 AlphaEvolve run and rewritten for readability (Stage 3).

A.2.6 Code: synthesis program

```
def rat_bandit_synthesis(
    params: chex.Array,
    choice: int,
    reward: int,
    agent_state: Optional[chex.Array],
) -> tuple[chex.Array, chex.Array]:
  """Handcrafted agent for the Rat Bandit task based on insights from discovered
     programs.
  """
  if agent_state is None:
    agent_state = jnp.array([0.5, 0.5, 0.0, 0.0])

  (
    beta,
    one_back_weight,
    recency_loss_weight,
  ) = map(jnp.abs, params[0:3])
  (
      forgetting_rate,    # Rate at which recency effects decay over time.
      alpha_chosen,          # Learning rate for the chosen option.
      alpha_unchosen_fraction, # Fraction of alpha_chosen for unchosen option.
  ) = map(jax.nn.sigmoid, params[3:6])
  bias_choice_0 = params[6]
  alpha_unchosen = alpha_chosen * alpha_unchosen_fraction

  # Apply forgetting to all state variables.
  decayed_agent_state = agent_state * forgetting_rate

  # Update fast learner, which has linked values
  decayed_q_values = decayed_agent_state[:2]
  prediction_error = reward - decayed_q_values[choice]
  updated_q_values = decayed_q_values.at[choice].add(
      alpha_chosen * prediction_error
  )
  updated_q_values = updated_q_values.at[1 - choice].add(
      alpha_unchosen * -prediction_error
  )

  # Update slow learner, which increments values and relies on forgetting
  decayed_recency_effects = decayed_agent_state[2:4]
  updated_recency_effects = decayed_recency_effects.at[choice].add(
      recency_loss_weight * (1 - reward)
  )

  # Compute previous trial win-stay/lose-shift effect
  one_back_bonus = reward - one_back_weight
```

bioRxiv preprint doi: https://doi.org/10.64898/2026.05.18.725921; this version posted May 21, 2026. The copyright holder for this preprint (which was not certified by peer review) is the author/funder, who has granted bioRxiv a license to display the preprint in perpetuity. It is made available under a CC-BY 4.0 International license.

```
  # Update state variables for fast and slow learners.
  new_agent_state = jnp.concatenate([updated_q_values, updated_recency_effects])

  # Compute choice logits.
  # Fast and slow learners
  value_signals = updated_recency_effects + beta * jnp.exp(updated_recency_effects)
     * updated_q_values
  # Add the previous trial win-stay/lose-shift bonus
  value_signals_with_oneback = value_signals.at[choice].add(one_back_bonus)
  # Add bias.
  choice_logits = value_signals_with_oneback.at[0].add(bias_choice_0)

  return choice_logits, new_agent_state
```

Code 7: Synthesis program for the rat bandit dataset.

A.2.7 Additional figures

bioRxiv preprint doi: https://doi.org/10.64898/2026.05.18.725921; this version posted May 21, 2026. The copyright holder for this preprint (which was not certified by peer review) is the author/funder, who has granted bioRxiv a license to display the preprint in perpetuity. It is made available under a CC-BY 4.0 International license.

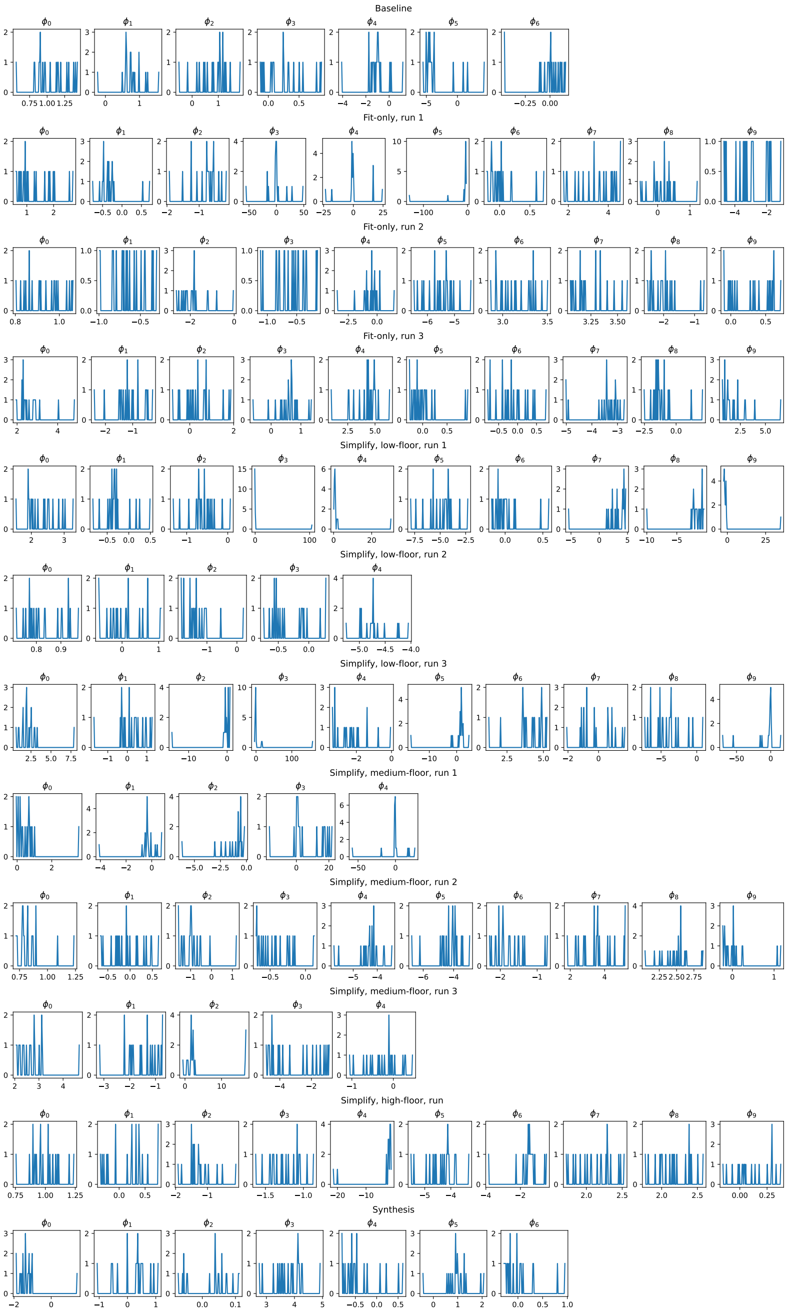

Fig. A5: ***Rat Bandit* Dataset: Fit parameters for each program**. The distribution of fit parameters for each fold of all discovered programs (fit-only and simplified), as well as the handcrafted baseline and synthesis program.

bioRxiv preprint doi: https://doi.org/10.64898/2026.05.18.725921; this version posted May 21, 2026. The copyright holder for this preprint (which was not certified by peer review) is the author/funder, who has granted bioRxiv a license to display the preprint in perpetuity. It is made available under a CC-BY 4.0 International license.

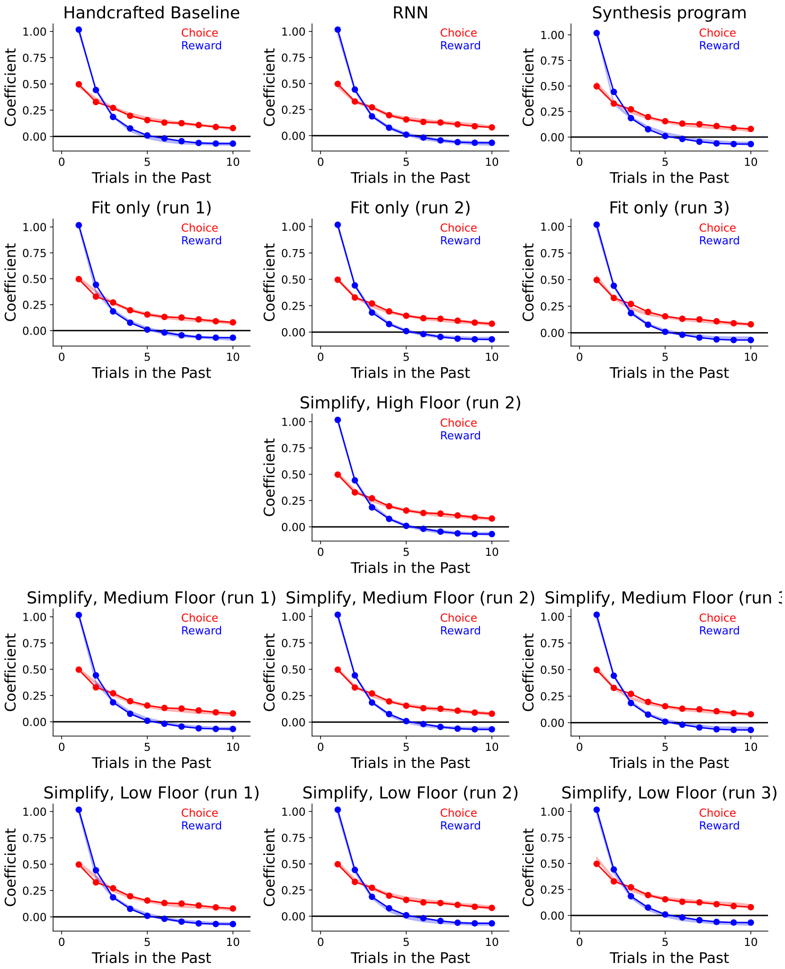

Fig. A6: ***Rat Bandit*** **Dataset: Trial-lagged regression analyses**. Here we see the trial-lagged regression analysis shown in Figure 6 for all discovered programs for this dataset. The coefficients for the real data are shown in solid lines, while the transparent patch shows the 95% prediction interval for the artificial data. We see that all models closely match the data.

bioRxiv preprint doi: https://doi.org/10.64898/2026.05.18.725921; this version posted May 21, 2026. The copyright holder for this preprint (which was not certified by peer review) is the author/funder, who has granted bioRxiv a license to display the preprint in perpetuity. It is made available under a CC-BY 4.0 International license.

A.3 Fly Bandit

A.3.1 Details of the dataset

Mohanta [34] considers fruit flies performing a two-armed bandit task with binary rewards. Flies performed the task in a Y-maze in which separate odors could be delivered to each of the three arms [81]. At the beginning of each trial, the arm containing the fly had no odor, and the other two arms contained one of two odors defining the choices. Flies indicated their choice on each trial by selecting an odor and walking to the end of the associated arm. Reward was delivered via a brief pulse of red light which activated flies' sugar-sensing neurons [82]. Reward probabilities followed a random block structure with randomly sampled reward probabilities and block lengths. Each fly performed one session. The dataset contains choices from 347 flies performing 68,000 total trials. Each fly completed a single session.

This dataset is available from Glenn Turner upon request.

A.3.2 Parameter fitting in *Fly Bandit*

Because each fly completed a single session, it is not possible to fit parameters separately to each subject's "even" and "odd" sessions. Thus, in order to fit parameters, we subdivide the sessions from all flies into a single "training" subject and a single "evaluation" subject. Within each "subject", we perform two-fold cross-validation as described in Section 7.3. When reporting single-subject likelihood scores (as in Figure 3b), we still compute normalized likelihoods separately for each fly, and we still average across flies to compute the final score.

A.3.3 Handcrafted Baseline Program

Rajagopalan et al. [81] and Mohanta [34] have performed extensive model comparison on similar datasets, and identified a popular model known as "Differential Forgetting Q-Learning" (DFQ) as performing at least as well as any other. We adopt DFQ as the handcrafted baseline model for this dataset.

A.3.4 Discussion of evolved programs

Perseveration

Nearly half of the programs included a perseveration, or "stickiness", factor. This models the animal's tendency to repeat previous actions across subsequent trials. The strength of perseveration is determined by trainable parameters. We observe this feature in all simplified programs.

Eligibility traces

A third of the programs made use of *eligibility traces*. While typically considered as a "bridge from temporal-difference (TD) to Monte Carlo methods" [23], they can also serve as a form of memory for the occurrence of certain events (e.g. rewarding arms). They can be useful in bandit settings when rewards are non-stationary, as in our setup. The weight given eligibility traces in the update rule is determined by traininable

bioRxiv preprint doi: https://doi.org/10.64898/2026.05.18.725921; this version posted May 21, 2026. The copyright holder for this preprint (which was not certified by peer review) is the author/funder, who has granted bioRxiv a license to display the preprint in perpetuity. It is made available under a CC-BY 4.0 International license.

parameters. It is worth noting that none of the low-floor programs with the lower included eligibility traces.

Confidence (difference in Q-values)

Over half of the discovered programs use the difference in learned action values as an indicator for *confidence*. Specifically, a higher difference in values is indicative of greater confidence in the higher-valued arm being rewarding. This confidence was sometimes used to drive exploration: less confidence resulted in a higher likelihood of selecting an arm randomly.

Reward history

Two thirds of the medium-and high-floor programs included some form of reward accumulation or history. This could take one of two forms: i) in some models, the choice-contingent reward history was used as a reference signal for computing prediction errors, a canonical feature of reinforcement learning; ii) in others, the total reward accumulated across the session, independent of which choices generated it, was used as gain control for learning, modulating the magnitude of updates. The latter form aligned with the notion of value sensitivity found in one of the discovered programs, where learning is slowed down if Q-values become too large; this is perhaps indicative of flies becoming reward-insensitive after too much stimulation.

Inverse temperature

Most of the medium- and high-floor programs use a parameterized inverse temperature to scale the final logits returned by the model, which helps control the exploration/exploitation tradeoff in arm selection. The value ultimately used to modulate the logits can be influenced by other factors such as confidence, mentioned above.

A.3.5 Discussion of synthesis program

The synthesis program is one of the generated programs (high-floor, run 1) with manual renaming of the variables for clarity. This program maintains action values, eligibility traces, and reward history.

The reward history is updated towards the most recent reward and decayed according to a decay parameter. A reward history weight is then computed by exponentiating the product of the reward history and a scaling parameter. Eligibility traces are first decayed according to a decay parameter; then the chosen option is increased according to a boost parameter, while the unchosen option is decreased by the same amount (with a floor at zero). The prediction error is given by the (signed) difference between the observed reward and the Q-value of the chosen option. The Q-value of the chosen option is then updated by multiplying the prediction error, a learning rate parameter, the reward history weight, and the eligibility trace for the chosen option. The Q-value of the unchosen option is decayed according to a decay parameter. The final decision variables (logits) are the product of the Q-values with an inverse temperature.

To verify the strength of our synthesis program, we evaluated a number of variants, all of which resulted in a reduction in accuracy. Specifically, we examined the following modifications to our synthesis program:

bioRxiv preprint doi: https://doi.org/10.64898/2026.05.18.725921; this version posted May 21, 2026. The copyright holder for this preprint (which was not certified by peer review) is the author/funder, who has granted bioRxiv a license to display the preprint in perpetuity. It is made available under a CC-BY 4.0 International license.

- Removing reward history weighting.
- Adding a surprise factor.
- Adding both a surprise factor and choice bias.
- Adding both a surprise factor and choice bias, and removing reward history weighting.
- Replacing eligibility traces with stickiness factors.

Figure A7 summarizes the comparison and demonstrates that, while all variants improve over the Handcrafted Baseline Program, our chosen synthesis program is the strongest of all variants.

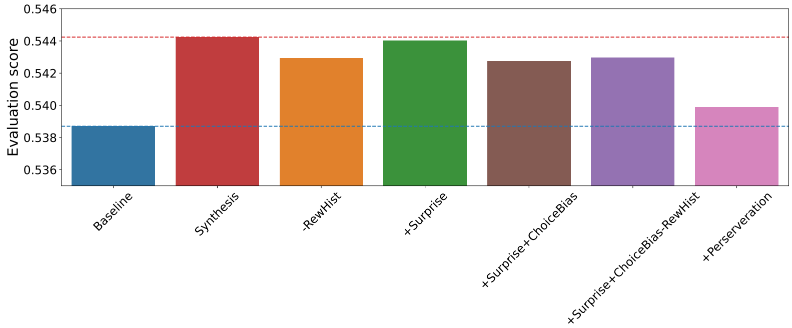

Fig. A7: **Comparison of Synthesis program against variants.**

A.3.6 Code: stage 2 ("Simplify") programs

```
def fly_bandit_run1__simplified_medium_floor(
    params: chex.Array,
    choice: int,
    reward: int,
    agent_state: Optional[chex.Array],
) -> tuple[chex.Array, chex.Array]:
  """Cognitive model describing fly behavior on a binary two-armed bandit task.

  This function implements a reinforcement learning model with the following
    features:
  - Q-learning with separate learning rates for positive and negative feedback.
  - Eligibility traces to attribute rewards to recent choices.
  - A forgetting mechanism for the value of the unchosen option.
  - A reward history mechanism that modulates the learning rate.

  Args:
    params: Fit parameters of the model.
    choice: The choice made by a fly on this trial. 0 or 1
    reward: The reward received by a fly on this trial. 0 or 1
    agent_state: The current state of the cognitive model.

  Returns:
    choice_logits: The probabilities that the fly will choose option 0 or 1 on
      the next trial, expressed as logits.
    agent_state: The updated state of the cognitive model.
  """
  # --- 1. Initialization ---
```

bioRxiv preprint doi: https://doi.org/10.64898/2026.05.18.725921; this version posted May 21, 2026. The copyright holder for this preprint (which was not certified by peer review) is the author/funder, who has granted bioRxiv a license to display the preprint in perpetuity. It is made available under a CC-BY 4.0 International license.

Table A3: Evaluation performance and program complexity for models in the *Fly Bandit* dataset. For programs generated by the "Simplify" stage, Floor represents the quality-of-fit threshold below which programs are discarded (see Section 7.6.2). Score indicates the average normalized likelihood across evaluation subjects (see Section 7.3); Effort is Halstead effort. State, Params, and Lines indicate the number of state variables, per-subject parameters, and lines of code respectively.

Model type	Floor	Run	Score	Effort	State	Params	Lines
Handcrafted Baseline	–	–	0.5404	16,737	2	4	64
RNN Baseline	–	–	0.5443	–	–	–	–
Stage 1: "Maximize Quality-of-Fit"	–	1	0.5451	109,605	7	10	167
		2	0.5451	164,570	7	10	177
		3	0.5449	165,779	9	10	139
Stage 2: "Simplify"	50%	1	0.5426	8,097	2	5	99
		2	0.5423	6,159	4	9	86
		3	0.5431	3,375	2	4	68
	75%	1	0.5432	18,797	5	10	122
		2	0.5436	7,191	5	5	87
		3	0.5437	12,065	2	9	124
	90%	1	0.5443	19,015	5	8	122
		2	0.5439	32,865	6	10	127
		3	0.5445	23,530	4	9	140
Synthesis Program	–	–	0.5443	19,015	5	8	71

```
  # If this is the first trial, initialize the agent's state with zeros.
  # The state vector contains: [q_value_0, q_value_1, trace_0, trace_1, reward_history]
  if agent_state is None:
    agent_state = jnp.zeros((5,))

  # --- 2. Unpack Model Parameters ---
  # Apply a sigmoid function to constrain rate and decay parameters between 0 and 1.
  learning_rate_positive, \
  learning_rate_negative, \
  eligibility_decay_chosen, \
  reward_history_decay = jax.nn.sigmoid(params[:4])

  # Other parameters are used directly without transformation.
  inverse_temperature = params[2]  # Used for choice probability (softmax)
  q_value_decay_unchosen = params[5] # Forgetting factor for the unchosen option's value
  eligibility_decay_unchosen = params[7] # Decay factor for the unchosen option's trace
  reward_history_beta = params[9] # Modulation factor of learning rate by reward history

  # --- 3. Unpack Agent State ---
  # Extract the different components from the agent's state vector.
  q_values = agent_state[0:2]          # Expected value for each choice
  eligibility_traces = agent_state[2:4] # Memory trace for recent choices
  reward_history = agent_state[4]       # Moving average of recent rewards

  # --- 4. Core Computations for Learning ---
```

bioRxiv preprint doi: https://doi.org/10.64898/2026.05.18.725921; this version posted May 21, 2026. The copyright holder for this preprint (which was not certified by peer review) is the author/funder, who has granted bioRxiv a license to display the preprint in perpetuity. It is made available under a CC-BY 4.0 International license.

```
# Determine the unchosen option.
unchosen_choice = 1 - choice

# Calculate the prediction error: the difference between actual and expected
   reward.
prediction_error = reward - q_values[choice]

# Update the reward history using an exponential moving average.
# This tracks the recent rate of rewards.
reward_history_updated = (
    reward_history * reward_history_decay
    + reward * (1 - reward_history_decay)
)

# Select the learning rate based on the sign of the prediction error.
# This allows for different learning speeds from positive vs. negative feedback.
base_learning_rate = jnp.where(
    prediction_error >= 0, learning_rate_positive, learning_rate_negative
)

# Modulate the learning rate by the reward history.
# This can increase learning speed in reward-rich environments.
learning_rate_modulated = base_learning_rate * (
    1.0 + reward_history_beta * reward_history
)

# --- 5. Update Eligibility Traces ---
# The eligibility trace for the unchosen option decays.
updated_trace_unchosen = eligibility_traces[unchosen_choice] *
   eligibility_decay_unchosen

# The trace for the chosen option is updated based on its previous value and a
   decay factor,
# and is incremented by 1 to mark its recent selection.
updated_trace_chosen = eligibility_traces[choice] * eligibility_decay_chosen +
   1.0

# Combine the updated traces.
eligibility_traces_updated = jnp.zeros_like(eligibility_traces).at[choice].set(
    updated_trace_chosen
).at[unchosen_choice].set(
    updated_trace_unchosen
)

# --- 6. Update Q-Values (Action Values) ---
# Calculate the change in value for the chosen option, scaled by the learning
   rate,
# prediction error, and the eligibility trace.
q_value_update_amount = (
    learning_rate_modulated
    * prediction_error
    * eligibility_traces_updated[choice]
)

# Apply the learning update to the chosen option's Q-value.
q_values_after_learning = q_values.at[choice].add(q_value_update_amount)

# Apply a decay (forgetting) factor to the unchosen option's Q-value.
q_values_updated = q_values_after_learning.at[unchosen_choice].set(
    q_values_after_learning[unchosen_choice] * q_value_decay_unchosen
)

# --- 7. Prepare Outputs ---
# Reassemble the updated components into the new agent state vector.
# jnp.newaxis is used to make the scalar reward_history a 1-element array for
   concatenation.
new_agent_state = jnp.concatenate(
```

bioRxiv preprint doi: https://doi.org/10.64898/2026.05.18.725921; this version posted May 21, 2026. The copyright holder for this preprint (which was not certified by peer review) is the author/funder, who has granted bioRxiv a license to display the preprint in perpetuity. It is made available under a CC-BY 4.0 International license.

```
        (q_values_updated, eligibility_traces_updated, reward_history_updated[jnp.
      newaxis])
    )

    # Calculate the choice logits for the next trial using the updated Q-values.
    # The inverse_temperature parameter controls the stochasticity of the choice (
      softmax).
    choice_logits = inverse_temperature * q_values_updated

    return choice_logits, new_agent_state
```

Code 8: Lowest-complexity program from Stage 2 AlphaEvolve run with 75% threshold for the fly bandit dataset, evolved from programs in the first independent Stage 1 AlphaEvolve run and rewritten for readability (Stage 3).

A.3.7 Code: synthesis program

```
def fly_bandit_synthesis(
    params: chex.Array,
    choice: int,
    reward: int,
    agent_state: Optional[chex.Array],
) -> Tuple[chex.Array, chex.Array]:
  """Handcrafted agent for the Fly Bandit task based on insights from discovered
    programs.

  Based on fly_bandit_run1_simplified_low_floor.
  """
  # --- 1. Initialization ---
  if agent_state is None:
    agent_state = jnp.zeros(5)

  # --- 2. Unpack parameters ---
  # Some are sigmoided...
  (
      learning_rate_positive,
      learning_rate_negative,
      eligibility_decay_rate,
      eligibility_boost,
      reward_history_decay,
  ) = jax.nn.sigmoid(params[:5])
  # Some are not.
  unchosen_q_decay_rate, inverse_temperature, reward_history_beta = params[5:8]

  # --- 3. Get agent state ---
  q_values = agent_state[:2]
  eligibility_traces = agent_state[2:4]
  reward_history = agent_state[4]

  # --- 4. Core model computations ---
  unchosen_choice = 1 - choice
  # Compute prediction error
  prediction_error = reward - q_values[choice]
  # Update reward history
  reward_history_updated = (
      reward_history * reward_history_decay +
      reward * (1.0 - reward_history_decay)
  )
  # Update eligibility traces
  traces_decayed = eligibility_traces * eligibility_decay_rate
  traces_boosted = traces_decayed.at[choice].add(eligibility_boost)
  traces_final = traces_boosted.at[unchosen_choice].add(-eligibility_boost)
  eligibility_traces_updated = jnp.maximum(0.0, traces_final)

  # Compute learning rate and learning modulators
```

bioRxiv preprint doi: https://doi.org/10.64898/2026.05.18.725921; this version posted May 21, 2026. The copyright holder for this preprint (which was not certified by peer review) is the author/funder, who has granted bioRxiv a license to display the preprint in perpetuity. It is made available under a CC-BY 4.0 International license.

```
    learning_rate = jnp.where(prediction_error >= 0,
                              learning_rate_positive,
                              learning_rate_negative)
    # Higher recent rewards can amplify learning
    reward_history_weight = jnp.exp(reward_history_beta * reward_history_updated)
    # Update amount depends on prediction error, learning rate, reward history,
    # and eligibility traces.
    q_update_delta = (prediction_error * learning_rate * reward_history_weight *
                      eligibility_traces_updated[choice])
    q_values_after_learning = q_values.at[choice].add(q_update_delta)
    q_values_updated = q_values_after_learning.at[unchosen_choice].set(
        q_values_after_learning[unchosen_choice] * unchosen_q_decay_rate)

    # --- 5. Prepare new state ---
    new_agent_state = jnp.concatenate(
        (q_values_updated, eligibility_traces_updated,
         jnp.array([reward_history_updated]))
    )

    # --- 6. Compute logits and return ---
    logits = inverse_temperature * q_values_updated
    return logits, new_agent_state
```

Code 9: Synthesis program for the fly bandit dataset.

A.3.8 Additional figures

bioRxiv preprint doi: https://doi.org/10.64898/2026.05.18.725921; this version posted May 21, 2026. The copyright holder for this preprint (which was not certified by peer review) is the author/funder, who has granted bioRxiv a license to display the preprint in perpetuity. It is made available under a CC-BY 4.0 International license.

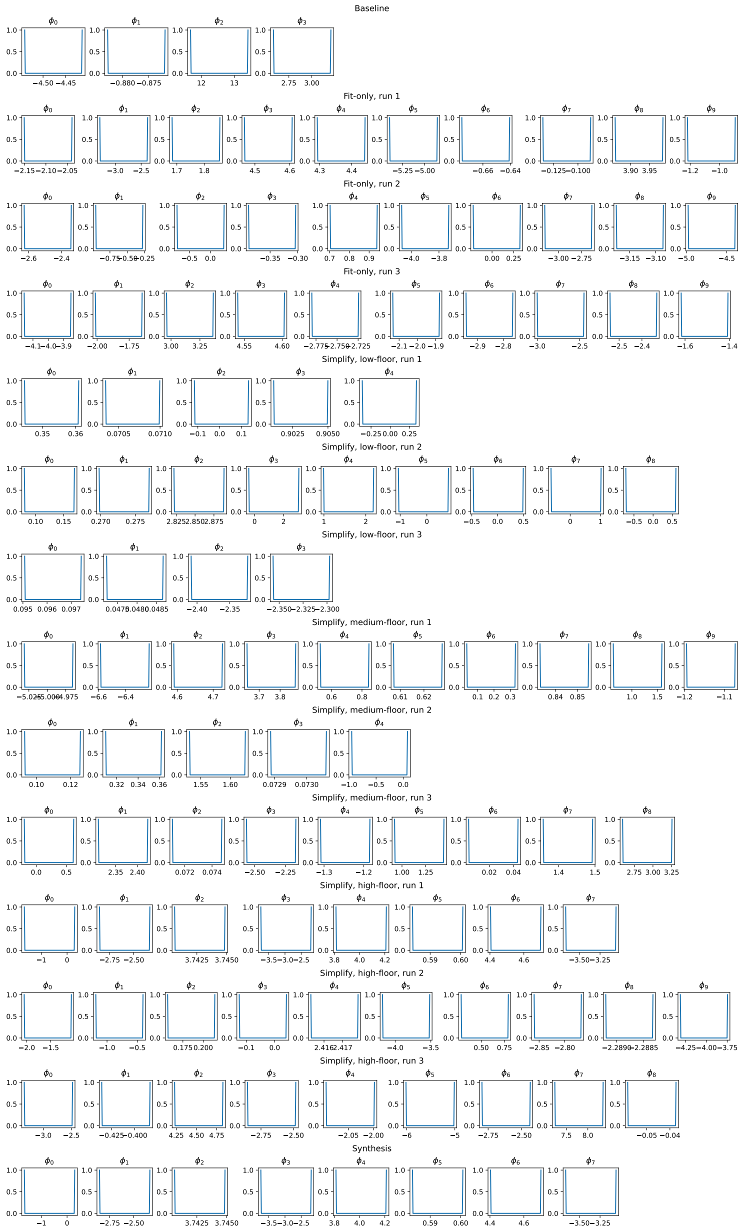

Fig. A8: ***Fly Bandit* Dataset: Fit parameters for each program**. The distribution of fit parameters for each fold of all discovered programs (fit-only and simplified), as well as the handcrafted baseline and synthesis program. For the *Fly Bandit* dataset, the evaluation set consists of all even-indexed sessions, treated as if the data belonged to one subject: thus, there are only two cross-validation folds to which parameters are fit (and thus two data points per histogram).

bioRxiv preprint doi: https://doi.org/10.64898/2026.05.18.725921; this version posted May 21, 2026. The copyright holder for this preprint (which was not certified by peer review) is the author/funder, who has granted bioRxiv a license to display the preprint in perpetuity. It is made available under a CC-BY 4.0 International license.

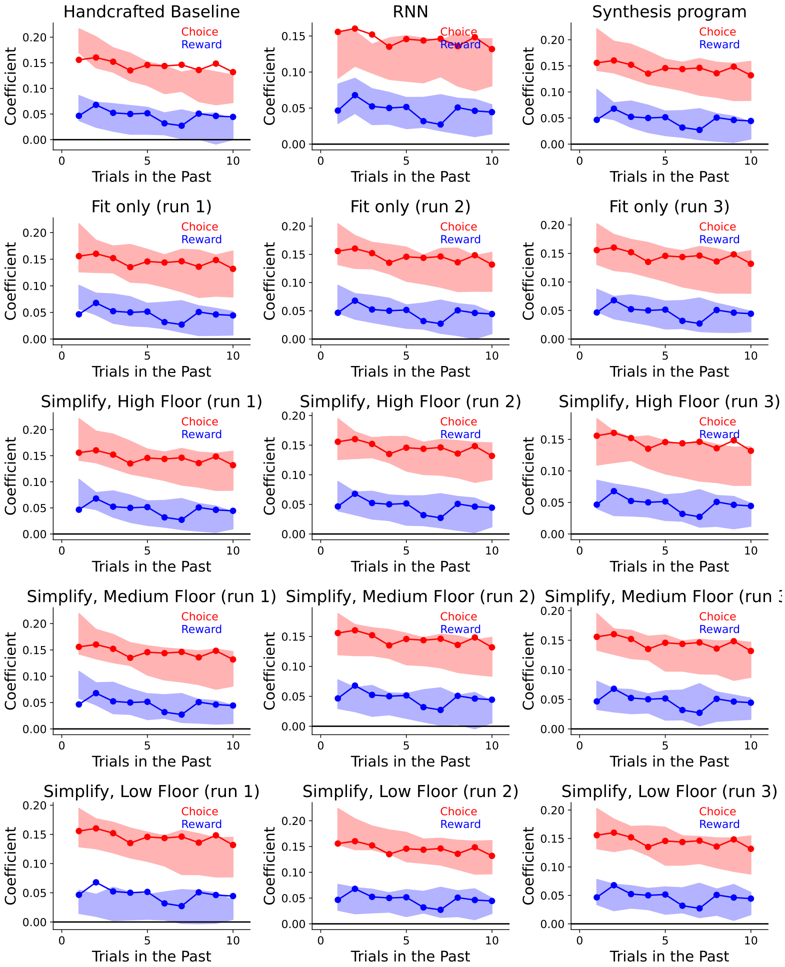

Fig. A9: ***Fly Bandit* Dataset: Trial-lagged regression analyses**. Here we see the trial-lagged regression analysis shown in Figure 6 for all discovered programs for this dataset. The coefficients for the real data are shown in solid lines, while the transparent patch shows the 95% prediction interval for the artificial data. We see that the handcrafted baseline model does not match the reward-independent choice-driven perseveration, overestimating the effect on previous choice for recent trials and underestimating it for more distant trials. The discovered models more often contain the real data within the prediction intervals. We note that this dataset is considerably more stochastic than other datasets, and the coefficients on the y-axis occupy a smaller range than for other datasets.

bioRxiv preprint doi: https://doi.org/10.64898/2026.05.18.725921; this version posted May 21, 2026. The copyright holder for this preprint (which was not certified by peer review) is the author/funder, who has granted bioRxiv a license to display the preprint in perpetuity. It is made available under a CC-BY 4.0 International license.

A.4 Monkey Bandit

A.4.1 Details of the dataset

Costa et al. [35] consider macaque monkeys performing a three-alternative bandit task using eye movements. On each trial three images are presented, and the monkey selects one of the images by making an eye movement to fixate on it. Each image was associated with a fixed probability of reward, either 20%, 50%, or 80%. The same set of three images was presented for a block of 10-30 trials, after which one of the images was switched out for a new image, with a new random reward probability, and a new block was begun. The dataset contains choices from nine monkeys performing 653 sessions and 412,342 total trials. This data can be made available upon publication.

A.4.2 Baseline Program

Costa et al. [35] introduced a Q-learning model which includes a novelty bonus for selecting a new image that has just become available, which we term "Novelty-Q". Subsequent work has explored alternative models [36, 83], but none of these has provided a compelling advantage over Novelty-Q for the considered subjects, so we adopt Novelty-Q as our human-discovered baseline model for this dataset.

A.4.3 Discussion of evolved programs

Cognitive variables

Nearly all programs divided their cognitive variables into two terms: an action value (or "Q-value") term which tracked expected reward for each choice, and a "novelty trace" term which was updated each time a novel option was introduced, and decayed over time. This division of cognitive variables surfaced in every program, across all levels of simplification, except for one (50% floor, run 2), a program which also shows worse performance at capturing the lagged regression novelty patterns (Figure A11). These variables were usually updated in a modular fashion (while some interactions arose in the more complex high-floor programs, these did not survive automated ablation).

These separate modules show a marked departure from the a core structural feature of the baseline model, in which all learning is localized to action values. This mechanism predicts separate neural substrates for reward learning and perceptual novelty. Prior models largely assumed a single action value tracking average reward with an added fixed novelty bonus—suggesting the brain encourages exploration via optimistic initialization [35, 46, 47]. However, this unified approach consistently underestimates the monkeys' initial novelty seeking (bottom panel of Figure 6, Figure A11).

Reward learning

The action values for the chosen option were typically incrementally updated with reward prediction error based learning rule. Additionally, there was often a decay on the unchosen action values toward the initial action value. This parameter is also the value to which action values are initialized following either the beginning of a new session or the introduction of a novel option. Surprisingly, parameter fits resulted in *negative* values for this parameter in programs that also had a novelty trace, despite

bioRxiv preprint doi: https://doi.org/10.64898/2026.05.18.725921; this version posted May 21, 2026. The copyright holder for this preprint (which was not certified by peer review) is the author/funder, who has granted bioRxiv a license to display the preprint in perpetuity. It is made available under a CC-BY 4.0 International license.

monkeys usually showing a preference for novel options. This was possible because novelty preference is represented by a separate variable that can counteract this. The baseline models, which also had a parameter for initial action values, returned positive values for this parameter for all monkeys.

Among the more complex "high floor" programs, two out of three introduced differential learning rates for rewards and omissions, which had a small positive contribution to performance. This was the only motif from the high floor programs that was incorporated into the synthesis program.

Novelty bonus

A recurring motif involved having the novelty trace updated by placing a strongly positive bonus parameter on the novel option, which was then gradually decayed toward zero on each trial. Interestingly, the discovered novelty traces were usually updated independently of both reward and choice: there was no dependence on how frequently the option had actually been sampled, or whether it resulted in reward. This is consistent with perceptual novelty that is driving novelty preference. Two programs from run 2 (medium- and high-floor) did exhibit an additional decay on unchosen options; however, ablations did not reveal that they consistently contributed to model performance.

Nonlinear, Nonstationary Exploration

A consistent motif across programs was a nonstationary, nonlinear exploration function mapping cognitive variables to the decision variables. This is part of a trend we see across discovered programs in this work, in which the function mapping cognitive variables to choice departs from the simple softmax rule often assumed. The generalized form of this mapping was:

$$(\phi_i + \phi_j \text{var}(Q)(N + Q)$$

where Q and N are action value and novelty trace vectors respectively, and ϕ_i and ϕ_j are fittable parameters. This has the effect of increasing choice stochasticity when all action values are similar to each other, and making choices more deterministic when there is higher variance. We note that because novel options cause the corresponding action value to be set to a negative number, which is necessarily lower than the running average because rewards are only 0 and 1, var(Q) will be particularly high following the introduction of novel options as well.

A.4.4 Discussion of synthesis program

The synthesis program combined the elements described above that appeared consistently across the programs and contributed robustly to quality-of-fit. It consisted of two cognitive variables, Action Values (or Q-values) and Novelty Trace, which were updated in a modular fashion. Action values were updated by the learning rule described above (error-driven learning on the chosen action value with differential learning rates for rewards and omissions, decay on unchosen action values toward the initial action value parameter, reset on action value to initial action value parameter for

bioRxiv preprint doi: https://doi.org/10.64898/2026.05.18.725921; this version posted May 21, 2026. The copyright holder for this preprint (which was not certified by peer review) is the author/funder, who has granted bioRxiv a license to display the preprint in perpetuity. It is made available under a CC-BY 4.0 International license.

novel options). Novelty trace was reset to a parametric novelty bonus parameter following the introduction of a novel option, and non-novel options were decayed toward zero, as described above. Cognitive variables for action values (Q) and Novelty trace (N) were mapped onto the decision variables using the expression $(\phi_i + \phi_j \text{var}(Q)(N + Q)$.

Table A4: Evaluation performance and program complexity for models in the *Monkey Bandit* dataset. For programs generated by the "Simplify" stage, Floor represents the quality-of-fit threshold below which programs are discarded (see Section 7.6.2). Score indicates the average normalized likelihood across evaluation subjects (see Section 7.3); Effort is Halstead effort. State, Params, and Lines indicate the number of state variables, per-subject parameters, and lines of code respectively.

Model type	Floor	Run	Score	Effort	State	Params	Lines
Handcrafted Baseline	–	–	0.4220	12,352	3	6	70
RNN Baseline	–	–	0.4166	–	–	–	–
Stage 1: "Maximize Quality-of-Fit"	–	1	0.4264	60,406	6	10	96
		2	0.4267	72,710	6	10	104
		3	0.4270	240,368	12	10	162
Stage 2: "Simplify"	50%	1	0.4258	9,172	6	8	97
		2	0.4250	6,903	3	8	90
		3	0.4255	10,603	6	6	119
	75%	1	0.4255	10,081	6	9	138
		2	0.4258	18,965	6	10	130
		3	0.4258	14,309	6	8	100
	90%	1	0.4264	31,921	6	10	156
		2	0.4267	45,463	6	10	158
		3	0.4269	26,977	7	9	188
Synthesis Program	–	–	0.4267	16,523	6	9	102

A.4.5 Code: stage 2 ("Simplify") programs

```
def monkey_bandit_run1_simplified_medium_floor(
    params: chex.Array,
    choice: int,
    reward: float,
    novel_option: int,
    agent_state: Optional[chex.Array],
) -> tuple[chex.Array, chex.Array]:
  """Cognitive model describing monkey behavior on a multi-armed bandit task.

  Assumes the monkey is presented with three options on each trial.
  Occasionally, one of these options is changed to a novel arm with different
  reward probabilities, when this happens novel_option will indicate the index
  of the newly novel option and otherwise it will be -1.

  Args:
    params: Model params. Different parameters are used for different monkeys.
    choice: The choice made by the subject on the previous trial. An
      integer with values of 0, 1, or 2.
```

bioRxiv preprint doi: https://doi.org/10.64898/2026.05.18.725921; this version posted May 21, 2026. The copyright holder for this preprint (which was not certified by peer review) is the author/funder, who has granted bioRxiv a license to display the preprint in perpetuity. It is made available under a CC-BY 4.0 International license.

```
  reward: The reward received by the subject on this trial. A scalar
    either 0 or 1.
  novel_option: The choice option that is novel to the subject on this trial.
    The number 0, 1, 2 indicates one of the choices, and -1 indicates that
    no option is currently novel.
  agent_state: The current state of the cognitive model.

Returns:
  choice_logits: The probabilities that the subject will choose option
    0, 1, or 2 on the next trial, expressed as logits.
  agent_state: The updated state of the cognitive model.
"""
# --- 1. Initialization and Parameter Unpacking ---

# On the first trial, initialize the agent's state with zeros.
# The state vector holds Q-values and novelty values for the 3 options.
if agent_state is None:
  # State is a vector of size 6: [q0, q1, q2, n0, n1, n2]
  agent_state = jnp.zeros((6,))

# Unpack the model's parameters, which are learned for each subject.
(
    # Learning rate for the value of the chosen option.
    base_learning_rate_chosen,
    # Base inverse temperature for the softmax choice rule.
    beta_base,
    # Initial Q-value assigned to all options at the start or when novel.
    initial_q,
    # Learning rate for the value of the unchosen options.
    base_learning_rate_unchosen,
    # Initial bonus value assigned to a novel option.
    novelty_initial_bonus,
    # Rate at which the novelty bonus decays over time.
    novelty_decay_rate,
    # Factor scaling how much novelty affects the learning rate.
    novelty_learning_rate_scale,
    # Factor scaling how much Q-value variance affects beta.
    beta_q_variance_scale,
    *_  # stachenfeld@ added for unused parameters.
) = params

# --- 2. State Unpacking and Novelty Handling ---

# Split the agent state from the previous trial into Q-values and novelty values.
q_values_prev, novelty_values_prev = jnp.split(agent_state, 2)

# Create a one-hot encoded mask to identify which option is novel (-1 means none).
is_novel_option = jax.nn.one_hot(novel_option, num_classes=3)

# If an option is novel, reset its Q-value to the initial default value.
# Otherwise, keep the Q-value from the previous state.
q_values_after_novelty_reset = jnp.where(is_novel_option, initial_q, q_values_prev)

# If an option is novel, reset its novelty bonus to the initial high value.
novelty_values_after_reset = jnp.where(
    is_novel_option, novelty_initial_bonus, novelty_values_prev
)
# Apply a decay to all novelty values, so they decrease over time.
novelty_values = novelty_values_after_reset * (1 - novelty_decay_rate)

# --- 3. Q-Value Update (Reinforcement Learning) ---

# Create a one-hot encoded mask to identify the action chosen on the last trial.
is_chosen = jax.nn.one_hot(choice, num_classes=3)

# Calculate the prediction error (difference between expected and actual reward).
```

bioRxiv preprint doi: https://doi.org/10.64898/2026.05.18.725921; this version posted May 21, 2026. The copyright holder for this preprint (which was not certified by peer review) is the author/funder, who has granted bioRxiv a license to display the preprint in perpetuity. It is made available under a CC-BY 4.0 International license.

```
    # For the chosen option, error = reward - Q-value.
    # For unchosen options, they are assumed to regress towards the initial value.
    prediction_error = jnp.where(
        is_chosen,
        reward - q_values_after_novelty_reset,
        initial_q - q_values_after_novelty_reset,
    )

    # Determine the base learning rate for each option.
    # Use a higher rate for the chosen option and a lower one for unchosen options.
    # The sigmoid function transforms the raw parameters into a (0, 1) range.
    base_learning_rates = jnp.where(
        is_chosen,
        jax.nn.sigmoid(base_learning_rate_chosen),
        jax.nn.sigmoid(base_learning_rate_unchosen),
    )

    # Increase the learning rate for options that are still considered novel.
    effective_learning_rates = (
        base_learning_rates + novelty_learning_rate_scale * novelty_values
    )

    # Update the Q-values using the prediction error and the learning rates.
    # This is the core Rescorla-Wagner learning rule.
    q_values_updated = (
        q_values_after_novelty_reset
        + effective_learning_rates * prediction_error
    )

    # --- 4. Prepare for Next Choice ---

    # Calculate the softmax inverse temperature (beta).
    # Beta is dynamic: it increases when the Q-values are more spread out (higher
    # variance), leading to more deterministic (exploitative) choices.
    beta = beta_base + beta_q_variance_scale * jnp.var(q_values_updated)

    # Combine the learned Q-values and the current novelty values to get the
    # total value, or "attractiveness," of each option.
    total_value = q_values_updated + novelty_values

    # Calculate the choice logits for the next trial. These are the inputs to a
    # softmax function that determines the choice probabilities.
    # A higher logit means a higher probability of being chosen.
    choice_logits = beta * total_value

    # --- 5. Update and Return State ---

    # Concatenate the updated Q-values and novelty values to form the new agent state
      .
    agent_state_updated = jnp.concatenate((q_values_updated, novelty_values))

    # Return the choice logits and the new state for the next trial.
    return choice_logits, agent_state_updated
```

Code 10: Lowest-complexity program from Stage 2 AlphaEvolve run with 75% threshold for the monkey bandit dataset, evolved from programs in the first independent Stage 1 AlphaEvolve run and rewritten for readability (Stage 3).

A.4.6 Code: synthesis program

```
def monkey_bandit_synthesis(
    params: chex.Array,
    choice: int,
    reward: float,
    novel_option: int,
```

bioRxiv preprint doi: https://doi.org/10.64898/2026.05.18.725921; this version posted May 21, 2026. The copyright holder for this preprint (which was not certified by peer review) is the author/funder, who has granted bioRxiv a license to display the preprint in perpetuity. It is made available under a CC-BY 4.0 International license.

```
    agent_state: chex.Array | None,
) -> tuple[chex.Array, chex.Array]:
  """Handcrafted agent for the Monkey Bandit task based on insights from discovered
     programs.

  Has the following elements
  * Maintains separate q_values and novelty_trace state variables.
  * Updates q_values with Q-learning on chosen and decay toward baseline on
    unchosen
  * Updates novelty with bonus for novel options, decay on all other options
  * Differential learning on rewards & omissions
  """
  # --- 1. Unpack Parameters and State ---

  # Unpack the agent's fixed parameters.
  baseline_q_value, reward_learning_rate_logit, omission_learning_rate_logit, \
  base_inverse_temperature, novelty_decay_rate_logit, novelty_initial_value, \
  q_unchosen_decay_rate_logit, q_var_scalar, *_ = params

  reward_learning_rate = jax.nn.sigmoid(reward_learning_rate_logit)
  omission_learning_rate = jax.nn.sigmoid(omission_learning_rate_logit)
  base_inverse_temperature = jnp.abs(base_inverse_temperature)
  novelty_decay_rate = jax.nn.sigmoid(novelty_decay_rate_logit)
  q_unchosen_decay_rate = jax.nn.sigmoid(q_unchosen_decay_rate_logit)
  q_var_scalar = jnp.abs(q_var_scalar)

  # Initialize the agent's state if this is the first step (state is None).
  # The state vector contains Q-values and novelty bonuses for 3 options.
  if agent_state is None:
    agent_state = jnp.zeros(6)  # 3 Q-values and 3 novelty bonuses

  # Unpack the state into separate arrays for Q-values and novelty bonuses.
  q_values, novelty_trace = jnp.split(agent_state, 2)

  # --- 2. Update Novelty Bonuses ---

  # Create a one-hot mask to identify which option, if any, is newly introduced.
  # A value of -1 for `novel_option` means no new option was introduced,
  # and jax.nn.one_hot will correctly produce a vector of all zeros.
  is_novel_mask = jax.nn.one_hot(novel_option, num_classes=3)

  # For all existing options, their novelty bonuses are decayed.
  decayed_existing_bonuses = novelty_trace * novelty_decay_rate

  # Use the mask to apply the correct update: reset for the novel option, decay for
     others.
  novelty_bonuses = jnp.where(
      is_novel_mask, novelty_initial_value, decayed_existing_bonuses)

  # --- 3. Update Q-Values ---

  # This update logic has three distinct cases handled by `where` clauses:
  #   a) The option was newly introduced.
  #   b) The option was chosen.
  #   c) The option was available but not chosen.

  # Different learning rate depending on reward.
  learning_rate = jnp.where(
      reward > 0.0, reward_learning_rate, omission_learning_rate)

  # First, calculate the updates for existing options (cases b and c).
  is_chosen_mask = jax.nn.one_hot(choice, num_classes=3)

  # For the CHOSEN option (b), apply a standard Q-learning update.
  prediction_error = reward - q_values
  q_learning_update = learning_rate * prediction_error

  # For UNCHOSEN options (c), decay their values back towards the baseline.
```

bioRxiv preprint doi: https://doi.org/10.64898/2026.05.18.725921; this version posted May 21, 2026. The copyright holder for this preprint (which was not certified by peer review) is the author/funder, who has granted bioRxiv a license to display the preprint in perpetuity. It is made available under a CC-BY 4.0 International license.

```
    decay_update = q_unchosen_decay_rate * (baseline_q_value - q_values)

    # Combine updates for chosen and unchosen options.
    updates_for_existing_options = jnp.where(is_chosen_mask, q_learning_update,
        decay_update)
    updated_q_for_existing_options = q_values + updates_for_existing_options


    # Use the novelty mask to select the final Q-values:
    # - If an option is new, set its Q-value to the baseline.
    # - Otherwise, use the updated value for existing options calculated above.
    q_values = jnp.where(is_novel_mask, baseline_q_value,
        updated_q_for_existing_options)

    # --- 4. Calculate Outputs and Next State ---

    # The final value of an option is its Q-value plus its novelty bonus.
    total_values = q_values + novelty_bonuses

    # Calculate a dynamic inverse temperature for the softmax calculation.
    # This makes the agent's choices more exploratory when Q-values are similar.
    variance_of_q_values = jnp.var(q_values)
    dynamic_inverse_temperature = base_inverse_temperature + q_var_scalar *
        variance_of_q_values

    # Action logits are the inputs to a softmax function for choice probability.
    action_logits = total_values * dynamic_inverse_temperature

    # The next agent state is the concatenation of the newly updated values.
    next_agent_state = jnp.concatenate((q_values, novelty_bonuses))

    return action_logits, next_agent_state
```

Code 11: Synthesis program for the monkey bandit dataset.

A.4.7 Additional figures

bioRxiv preprint doi: https://doi.org/10.64898/2026.05.18.725921; this version posted May 21, 2026. The copyright holder for this preprint (which was not certified by peer review) is the author/funder, who has granted bioRxiv a license to display the preprint in perpetuity. It is made available under a CC-BY 4.0 International license.

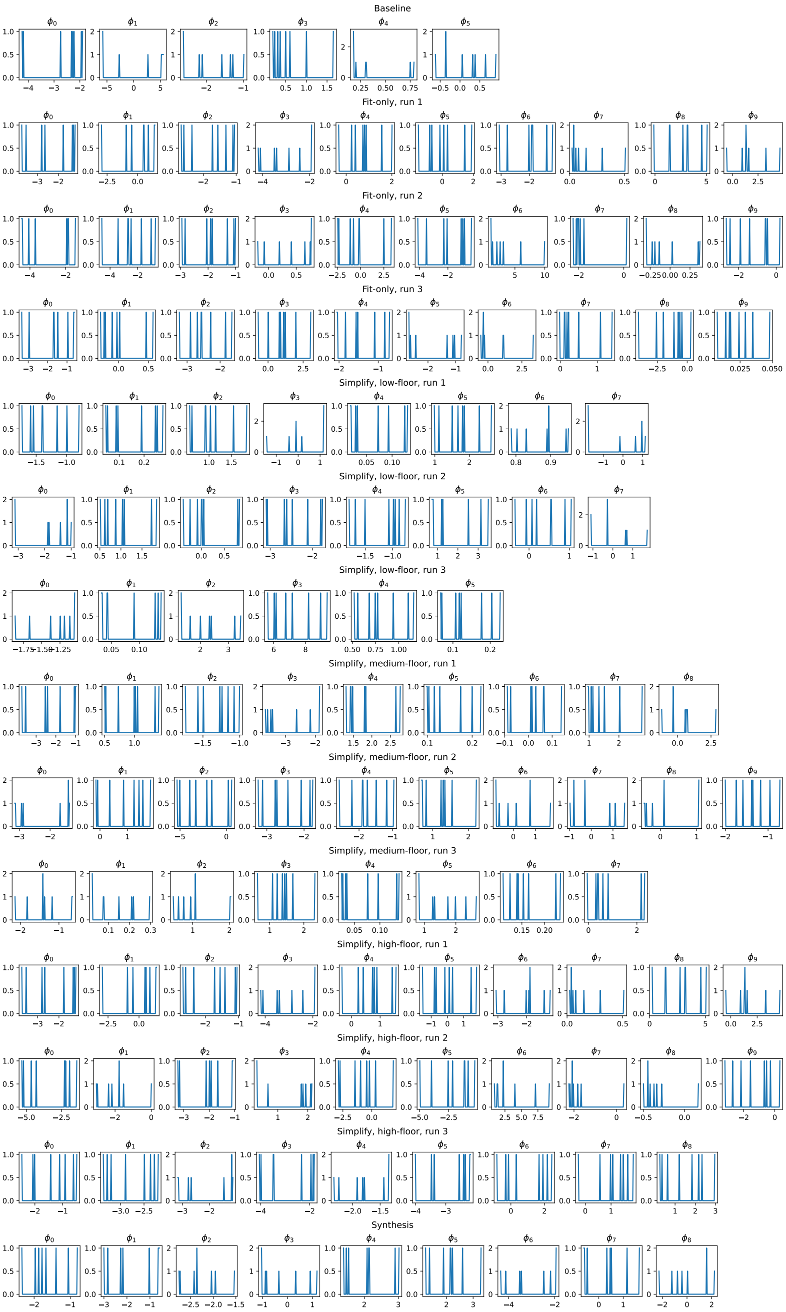

Fig. A10: ***Monkey Bandit* Dataset: Fit parameters for each program**. The distribution of fit parameters for each fold of all discovered programs (fit-only and simplified), as well as the handcrafted baseline and synthesis program.

bioRxiv preprint doi: https://doi.org/10.64898/2026.05.18.725921; this version posted May 21, 2026. The copyright holder for this preprint (which was not certified by peer review) is the author/funder, who has granted bioRxiv a license to display the preprint in perpetuity. It is made available under a CC-BY 4.0 International license.

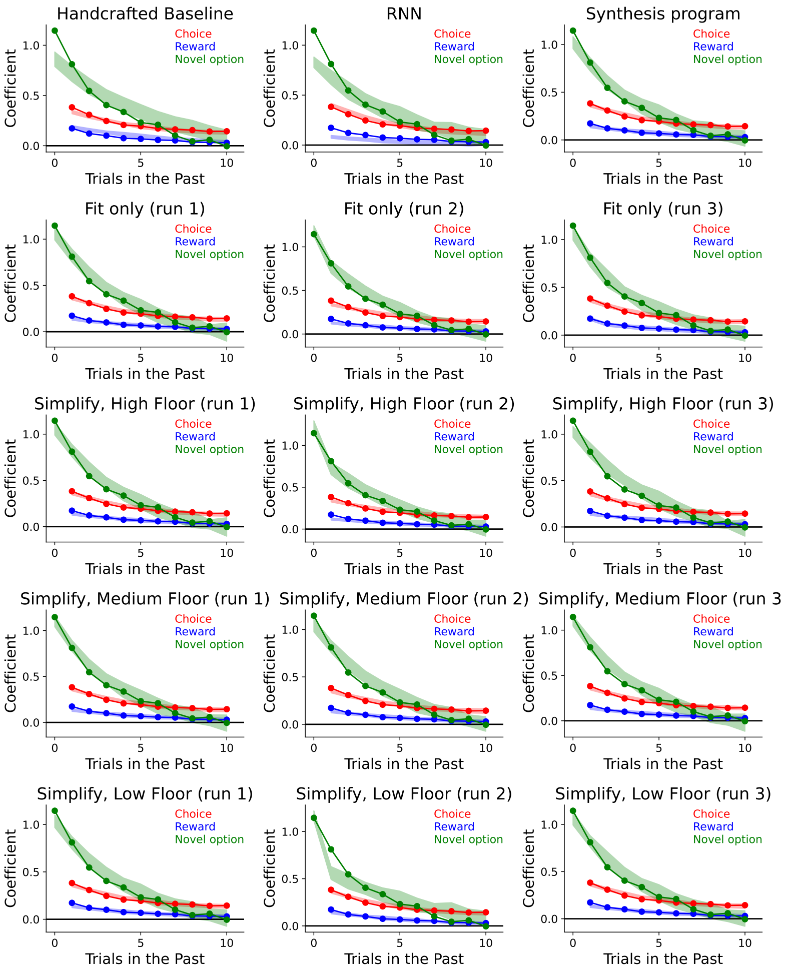

Fig. A11: ***Monkey Bandit* Dataset: Trial-lagged regression analyses**. Here we see the trial-lagged regression analysis shown in Figure 6 for all discovered programs for this dataset. The coefficients for the real data are shown in solid lines, while the transparent patch shows the 95% prediction interval for the artificial data. We see that the handcrafted baseline model does not match the timecourse of novelty-seeking, while the other models do.

bioRxiv preprint doi: https://doi.org/10.64898/2026.05.18.725921; this version posted May 21, 2026. The copyright holder for this preprint (which was not certified by peer review) is the author/funder, who has granted bioRxiv a license to display the preprint in perpetuity. It is made available under a CC-BY 4.0 International license.

A.5 Rat Twostep

A.5.1 Dataset

The rat two-step dataset [37] considers rats performing a two-step decision-making task that is commonly used to study model-based learning and decision-making. In the first step of each trial, the rat indicated its choice by entering one of two available "choice" ports. This was followed by one of two possible "outcome" ports becoming available. Each choice port was associated with one of the outcome ports, which became available following choices to that port with probability 80% (with probability 20% the other outcome port because available instead). The rat then entered the available outcome port and received a reward with probability that depended on the outcome port (but not the choice port). Reward probabilities were 80% and 20% for the two outcome ports and changed unpredictably in blocks. The dataset contains choices from 21 rats performing 1,960 sessions and 542,195 total trials.

We obtained this dataset from the following URL, where it is freely available under a permissive open-source license:
https://github.com/kevin-j-miller/MBB2017-rat-two-step-task

A.5.2 Baseline Model

The paper which introduced this dataset also introduced a computational model, which was improved upon in several subsequent papers [38, 84]. The best-fitting cognitive model is a mixture of three agents: model-based reward learning, model-based perseveration, and model-free perseveration. This model is different from others in the literature in that it contains no influence of model-free reward learning, as model comparisons have shown that this does not improve quality of fit on this dataset [37, 38, 84]. It is also unusual in that each agent controls the update of just one decision variable, which expresses a relative preference between the two choice ports, rather than a pair of them expressing an absolute value for each port. We adopt this model as the human-discovered baseline for the rat two-step dataset. For compatibility with our pipeline, we re-implemented this model in Jax.

A.5.3 Discussion of evolved programs

Model-free and model-based learning

All but two simplified programs compute a weighted sum between action values (model-free learning) and outcome values (model-based learning). Of the remaining two programs, one has something referred to as "Q-values" for actions but which actually resemble a recency trace (perseveration), and computes a weighted sum between these and the model-based values (see "Recency traces"); the other computes a weighted sum over two systems, but neither system is purely model-free (the "model-free" system updates Q-values using both model-free and and model-based prediction errors).

bioRxiv preprint doi: https://doi.org/10.64898/2026.05.18.725921; this version posted May 21, 2026. The copyright holder for this preprint (which was not certified by peer review) is the author/funder, who has granted bioRxiv a license to display the preprint in perpetuity. It is made available under a CC-BY 4.0 International license.

Inverse temperature

All simplified programs scale the weighted sums described above by an inverse temperature. In the majority of programs, inverse temperatures are given by a fixed parameter. In two programs (a medium- and a high-floor program, simplified from the same fit-only program), this inverse temperature changes over time, beginning at zero and grows over time, asymptoting at a final value specified by a fit parameter. This is expected to cause the models to make more random choices at the beginning of each session, and to slowly become more deterministic throughout the session. This pattern may be similar to the patterns identified in [38] using statistical models. The evolved models here represent an advance on this in that they are generative, runnable models.

Connecting actions and outcomes

In the underlying experiment, rats are assigned to one of two conditions: the congruent condition, in which the action matches the outcome 80% of the time, and the incongruent condition, in which the choice matches the outcome 20% of the time. In all simplified programs but one, the experimental condition (and thus the relationship between actions and outcomes) is implicitly encoded in the sign of a product of per-subject parameters (typically the model-based learning rate, the weight of the outcome values relative to the action values, and the inverse temperature). The remaining program explicitly learns the transition function over the course of a session, and thus does not use parameters to encode the relationship between actions and outcomes.

Update of unchosen values

All simplified programs update the (model-free) values for the unchosen action (three programs also decay the *chosen* action value before updating it). In all but one program this takes the form of decay towards a fixed target, either zero (five programs) or a per-subject parameter (three programs). The remaining program decays the value of the unchosen action towards half the value of the chosen action. Each of these processes can be thought of as a different model of forgetting.

Similarly, all programs decay their (model-free) values for the unobserved outcome (the *low floor* programs also decay the *observed* outcome value before updating it). In all programs but two, these updates take the form of decay, either to a fixed per-subject parameter (six programs) or to zero (one program). The remaining two programs do a form of counterfactual learning: one program decays the unobserved outcome value towards $1 - r$ where r is the reward observed in this trial (using the assumption that if outcome o has reward r, then outcome $1 - o$ would have reward $1 - r$). The other program decays first to zero, and then to $1 - r$.

Decay towards zero [41] and towards a fit parameter [39] are known motifs from the literature on related tasks. Counterfactual learning is a known motif in modeling human decision-making in related tasks, though it is usually deployed in situations where participants were aware of the counterfactual outcome (what would have happened had they chosen the alternative option) [85, 86]. Notably, "counterfactual" learning for the unchosen action, using the same learning rate as for the chosen action, is equivalent to tracking only a single decision variable which expresses a relative

bioRxiv preprint doi: https://doi.org/10.64898/2026.05.18.725921; this version posted May 21, 2026. The copyright holder for this preprint (which was not certified by peer review) is the author/funder, who has granted bioRxiv a license to display the preprint in perpetuity. It is made available under a CC-BY 4.0 International license.

preference [8, 37]. These motifs have not, to our knowledge, been deployed in computational models of the rat two-step task. Decay towards the current value of the chosen action is to our knowledge an entirely novel computational motif, which occurs in our evolved models of the human bandit dataset as well.

Bias and stickiness terms

All simplified programs except for one have at least one of the following: a direct bias towards one action or another, specified by a per-subject parameter (four programs); an "action stickiness" term directly rewarding or penalizing the immediately previous action (five programs); and/or an "outcome stickiness" term directly rewarding the choice with the same index as the current outcome (four programs; one of these has a separate outcome stickiness term for each outcome). No program has all three.

Habit traces

Three programs (none of which include an "action stickiness" term as described above) use something resembling an habit trace for action perseveration [45]. Namely, each of these three programs maintain per-action values which decay when the action is unchosen, and increase when the action is chosen. These can be thought of as maintaining a running average of how often each action has been chosen in the recent past. As mentioned above, one of these programs misleadingly refers to its recency trace as "Q-values"; the others call their recency traces "stickiness values".

A.5.4 Discussion of synthesis program

Due to both prior literature and our analysis of discovered programs revealing that ablating the model-free component of these programs (e.g., by replacing them with an eligibility trace) generally has a minimal effect on quality of fit, the synthesis program specifically omits model-free (action) Q-values. Aside from this, the program follows the same basic skeleton as most of the discovered programs, with the final choice logits a weighted sum of a habit trace and model-based (outcome) values, where the sign of the model-based weight is positive for subjects in the congruent condition, and negative in the incongruent condition. The outcome-based values decay towards a per-subject fitted parameter; the habit trace, which only requires one state variable to store (its learning target is $+1$ when the rat chooses right, and -1 when the rat chooses left), decays towards 0.

The synthesis model includes both a direct left-right bias and an "action stickiness" bias favoring the last action performed by the rat. Thus, there are two separate perseveration pathways: one favoring repeating the immediately preceding action (the action stickiness bias), and one favoring repeating whichever action was performed most frequently in the recent past (the habit trace).

Finally, the synthesis program incorporates the dynamic inverse temperature calculation present in two of the discovered programs, in which the inverse temperature gradually decays from zero to one at a fixed rate (resulting in the stochasticity of the program's choices gradually decreasing). This perhaps models the rat's re-acclimatization to the task at the beginning of each session.

bioRxiv preprint doi: https://doi.org/10.64898/2026.05.18.725921; this version posted May 21, 2026. The copyright holder for this preprint (which was not certified by peer review) is the author/funder, who has granted bioRxiv a license to display the preprint in perpetuity. It is made available under a CC-BY 4.0 International license.

Table A5: Evaluation performance and program complexity for models in the *Rat Two-step* dataset. For programs generated by the "Simplify" stage, Floor represents the quality-of-fit threshold below which programs are discarded (see Section 7.6.2). Score indicates the average normalized likelihood across evaluation subjects (see Section 7.3); Effort is Halstead effort. State, Params, and Lines indicate the number of state variables, per-subject parameters, and lines of code respectively.

Model type	Floor	Run	Score	Effort	State	Params	Lines
Handcrafted Baseline	–	–	0.6405	26,560	4	9	84
RNN Baseline	–	–	0.6528	–	–	–	–
Stage 1: "Maximize Quality-of-Fit"	–	1	0.6543	241,747	10	10	178
		2	0.6550	105,438	4	10	96
		3	0.6540	108,168	11	10	172
Stage 2: "Simplify"	50%	1	0.6491	10,892	4	8	122
		2	0.6477	5,180	4	7	29
		3	0.6489	8,522	4	9	137
	75%	1	0.6511	20,260	5	10	138
		2	0.6515	12,291	4	9	76
		3	0.6520	18,386	7	8	114
	90%	1	0.6533	51,371	10	10	152
		2	0.6539	26,072	4	10	132
		3	0.6533	28,584	7	10	131
Synthesis Program	–	–	0.6510	17,003	4	9	97

A.5.5 Code: stage 2 ("Simplify") programs

```
def rat_twostep_run1_simplified_medium_floor(
    params: chex.Array,
    choice: int,
    reward: int,
    outcome: int,
    agent_state: Optional[chex.Array],
) -> tuple[chex.Array, chex.Array]:
  """Cognitive model describing rat behavior on a binary two-step task.

  This model learns in several ways:
  1.  Action Values (Q-values): Learns the value of taking each action.
  2.  Outcome Values: Learns the value of arriving at each outcome.
  3.  Pavlovian-Instrumental Transfer (PIT): Learns an association between
      actions and outcomes, allowing outcome values to influence action choice.
  4.  Perseveration: Includes biases to repeat the previous action or the
      action associated with the previous outcome.

  Args:
    params: Model params. Different parameters are used for different rats.
    choice: Previous choice. Values: 0 or 1.
    reward: Previous reward. Values: 0 or 1.
    outcome: Previous outcome observed following the choice. Values: 0 or 1.
    agent_state: Previous state of the agent. A vector containing the agent's
      internal learned values.

  Returns:
    choice_logits: Vector of shape (2,) with the probabilities that the rat will
      choose option 0 or 1 on the next trial, expressed as logits.
```

bioRxiv preprint doi: https://doi.org/10.64898/2026.05.18.725921; this version posted May 21, 2026. The copyright holder for this preprint (which was not certified by peer review) is the author/funder, who has granted bioRxiv a license to display the preprint in perpetuity. It is made available under a CC-BY 4.0 International license.

```
    agent_state: New state of the agent, after observing the previous choice and
      reward.
  """
  # --- 1. Unpack and Transform Model Parameters ---

  # Unpack all raw (pre-transformation) parameters from the input array.
  (
      initial_q_value,
      learning_rate_action_raw,
      learning_rate_outcome_raw,
      softmax_inverse_temperature_raw,
      outcome_to_action_learning_rate_raw,
      decay_rate_action_raw,
      outcome_association_strength,
      action_perseveration,
      outcome_perseveration_0_raw,
      outcome_perseveration_1_raw,
  ) = params

  # Ensure certain parameters (learning rates, temperature) are positive
  # by applying the softplus function. This is a standard way to handle
  # constrained parameters during optimization.
  learning_rate_action = jax.nn.softplus(learning_rate_action_raw)
  learning_rate_outcome = jax.nn.softplus(learning_rate_outcome_raw)
  softmax_inverse_temperature = jax.nn.softplus(softmax_inverse_temperature_raw)
  outcome_to_action_learning_rate = jax.nn.softplus(
     outcome_to_action_learning_rate_raw)
  decay_rate_action = jax.nn.softplus(decay_rate_action_raw)

  # Combine the two outcome perseveration parameters into a single array.
  outcome_perseveration = jnp.array([outcome_perseveration_0_raw,
     outcome_perseveration_1_raw])


  # --- 2. Initialize or Retrieve Agent State ---

  # If this is the first trial (agent_state is None), initialize a new state.
  # The state vector tracks the following learned values:
  # agent_state[0]: Q-value of action 0
  # agent_state[1]: Q-value of action 1
  # agent_state[2]: Value of outcome 0
  # agent_state[3]: Value of outcome 1
  # agent_state[4]: Learned association weight between actions and outcomes (PIT)
  if agent_state is None:
    agent_state = jnp.zeros(5)


  # --- 3. Update Agent State Based on Previous Trial's Experience ---

  # Calculate the prediction error for the chosen action.
  # This is the difference between the reward received and the expected value (Q-
     value).
  prediction_error_action = reward - agent_state[choice]

  # Update the Q-value for the CHOSEN action using the prediction error.
  # This is a standard Rescorla-Wagner update rule.
  q_value_update_chosen_action = learning_rate_action * prediction_error_action

  # Update the Q-value for the UNCHOSEN action by decaying it towards the initial
     value.
  # This represents forgetting or a belief that unchosen options revert to a mean
     value.
  q_value_decay_unchosen_action = decay_rate_action * (initial_q_value -
     agent_state[1 - choice])

  # Update the value of the OBSERVED outcome based on the reward received.
  outcome_value_update_observed = learning_rate_outcome * (reward - agent_state[
     outcome + 2])
```

bioRxiv preprint doi: https://doi.org/10.64898/2026.05.18.725921; this version posted May 21, 2026. The copyright holder for this preprint (which was not certified by peer review) is the author/funder, who has granted bioRxiv a license to display the preprint in perpetuity. It is made available under a CC-BY 4.0 International license.

```

    # Decay the value of the UNOBSERVED outcome towards the initial value.
    outcome_value_decay_unobserved = learning_rate_outcome * (initial_q_value -
        agent_state[1 - outcome + 2])

    # Update the outcome-to-action association weight. This weight determines how
        strongly
    # outcome values influence action choices.
    association_weight_update = outcome_to_action_learning_rate *
        prediction_error_action

    # Apply all the calculated updates to the agent's state vector.
    agent_state = agent_state.at[choice].add(q_value_update_chosen_action)
    agent_state = agent_state.at[1 - choice].add(q_value_decay_unchosen_action)
    agent_state = agent_state.at[outcome + 2].add(outcome_value_update_observed)
    agent_state = agent_state.at[1 - outcome + 2].add(outcome_value_decay_unobserved)
    agent_state = agent_state.at[4].add(association_weight_update)


    # --- 4. Calculate Action Preferences (Combined Q-values) for the Next Choice ---

    # Start with the learned Q-values for each action (the "instrumental" component).
    instrumental_values = agent_state[0:2]

    # Calculate the influence from outcome values (the "Pavlovian" component).
    # The learned association weight is passed through a sigmoid to be between 0 and
        1.
    outcome_values = agent_state[2:4]
    association_weight = jax.nn.sigmoid(agent_state[4])
    pavlovian_influence = association_weight * outcome_association_strength *
        outcome_values

    # Combine the instrumental and Pavlovian values.
    combined_q_values = instrumental_values + pavlovian_influence

    # Add a perseveration bonus to the action that was just chosen, making it more
        likely to be repeated.
    combined_q_values = combined_q_values.at[choice].add(action_perseveration)

    # Add a perseveration bonus for the outcome that was just observed. This promotes
    # choosing the action that is associated with that outcome.
    # Note: This assumes a mapping where choice `i` is associated with outcome `i`.
    combined_q_values = combined_q_values.at[outcome].add(outcome_perseveration[
        outcome])


    # --- 5. Convert Action Preferences to Choice Logits ---

    # Scale the final Q-values by the inverse temperature. A higher value (lower "
        temperature")
    # leads to more deterministic choices, while a lower value leads to more random
        choices.
    # The result is the choice logits, which can be passed to a softmax function to
        get probabilities.
    choice_logits = combined_q_values * softmax_inverse_temperature

    return choice_logits, agent_state
```

Code 12: Lowest-complexity program from Stage 2 AlphaEvolve run with 75% threshold for the rat two-step dataset, evolved from programs in the first independent Stage 1 AlphaEvolve run and rewritten for readability (Stage 3).

A.5.6 Code: synthesis program

```
def rat_twostep_synthesis(
```

bioRxiv preprint doi: https://doi.org/10.64898/2026.05.18.725921; this version posted May 21, 2026. The copyright holder for this preprint (which was not certified by peer review) is the author/funder, who has granted bioRxiv a license to display the preprint in perpetuity. It is made available under a CC-BY 4.0 International license.

```
    params: chex.Array,
    choice: int,
    reward: int,
    outcome: int,
    agent_state: Optional[chex.Array],
) -> tuple[chex.Array, chex.Array]:
  """
  A manual implementation of a rat two-step task model.

  This model combines a recency trace for perseveration, outcome-based
  Q-values, and a dynamic inverse temperature to predict choice behavior.

  Args:
    params: An array of model parameters:
      - params[0]: model_based_weight (weight of outcome values)
      - params[1]: model_based_learning_rate (learning rate for outcome values)
      - params[2]: model_based_forgetting_rate (decay rate for outcome values)
      - params[3]: model_based_forgetting_target (target for outcome value decay)
      - params[4]: perseveration_weight (weight of recency trace)
      - params[5]: perseveration_forgetting_rate (decay rate for recency trace)
      - params[6]: bias (choice bias)
      - params[7]: prev_choice_stickiness (bonus added to the logit of the
                   previous choice)
      - params[8]: inverse_temperature_convergence_rate (rate at which inverse
                   temperature approaches its final value)
    choice: The action taken by the agent in the previous step (0 or 1).
    reward: The reward received after the previous choice (e.g., 0 or 1).
    outcome: The outcome state observed after the previous choice (0 or 1).
    agent_state: The agent's internal state from the previous step,
                 consisting of [recency_trace (1,), outcome_values (2,),
                 prev_inverse_temperature (1,)]. Initialized to zeros if None.

  Returns:
    A tuple containing:
      - choice_logits: Logits for the next choice (shape 2).
      - new_agent_state: The updated agent state for the next step.
  """

  # The agent state consists of a recency trace on actions, and outcome-based'
  # Q-values.
  if agent_state is None:
    agent_state = jnp.zeros(4)
  recency_trace, outcome_values, prev_inverse_temperature = jnp.split(
      agent_state, [1, 3]
  )

  # Outcome-value weight can be positive or negative, and its sign encodes
  # whether this rat is in the congruent or incongruent condition.
  model_based_weight = params[0]
  # Learning and decay rates are bounded between 0 and 1.
  model_based_learning_rate = jax.nn.sigmoid(params[1])
  model_based_forgetting_rate = jax.nn.sigmoid(params[2])
  model_based_forgetting_target = params[3]

  # Empirically, there should be a positive perseveration effect.
  perseveration_weight = jax.nn.softplus(params[4])
  perseveration_forgetting_rate = jax.nn.sigmoid(params[5])
  bias = params[6]
  prev_choice_stickiness = params[7]

  inverse_temperature_convergence_rate = jax.nn.sigmoid(params[8])

  # First, decay the outcome values towards the baseline, then update the value
  # of the chosen outcome.
  outcome_values_after_decay = outcome_values + model_based_forgetting_rate * (
      model_based_forgetting_target - outcome_values
  )
  outcome_prediction_error = reward - outcome_values_after_decay[outcome]
```

bioRxiv preprint doi: https://doi.org/10.64898/2026.05.18.725921; this version posted May 21, 2026. The copyright holder for this preprint (which was not certified by peer review) is the author/funder, who has granted bioRxiv a license to display the preprint in perpetuity. It is made available under a CC-BY 4.0 International license.

```python
outcome_update = model_based_learning_rate * outcome_prediction_error
updated_outcome_values = outcome_values_after_decay.at[outcome].add(
    outcome_update
)

# Decay the recency trace, then add 1 to the trace of the chosen action.
updated_recency_trace = recency_trace * perseveration_forgetting_rate + (2 *
   choice - 1)

# Update the inverse temperature.
inverse_temperature = (
    prev_inverse_temperature
    + inverse_temperature_convergence_rate
    * (1 - prev_inverse_temperature)
)

# Ultimately we're just taking a weighted sum of the recency trace and the
# outcome values, and applying a bias term.
choice_logits = inverse_temperature * (
    perseveration_weight * updated_recency_trace * jnp.array([-1.0, 1.0])
    + model_based_weight * updated_outcome_values
) + bias * jnp.array([-1.0, 1.0])
choice_logits = choice_logits.at[choice].add(prev_choice_stickiness)

return choice_logits, jnp.concatenate(
    [updated_recency_trace, updated_outcome_values, inverse_temperature]
)
```

Code 13: Synthesis program for the rat two-step dataset.

A.5.7 Additional figures

bioRxiv preprint doi: https://doi.org/10.64898/2026.05.18.725921; this version posted May 21, 2026. The copyright holder for this preprint (which was not certified by peer review) is the author/funder, who has granted bioRxiv a license to display the preprint in perpetuity. It is made available under a CC-BY 4.0 International license.

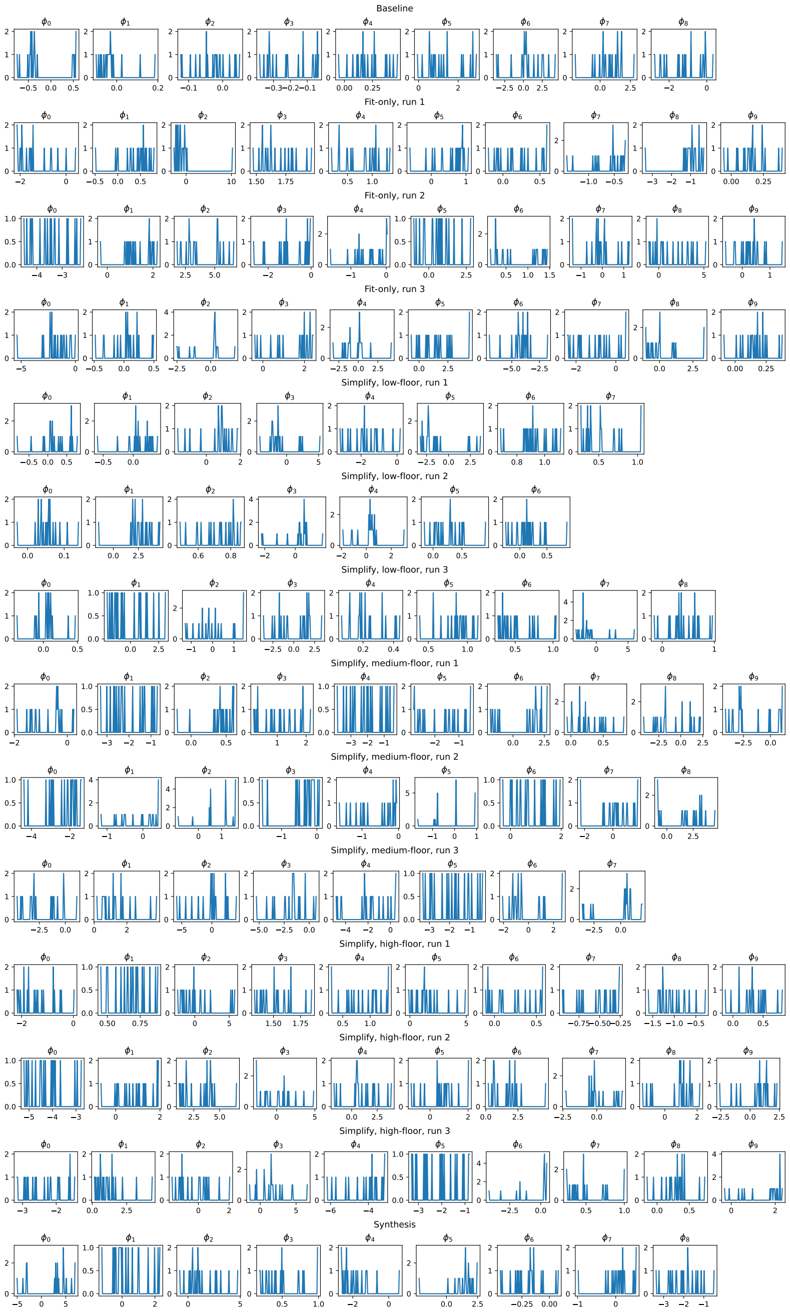

Fig. A12: ***Rat Two-step* Dataset: Fit parameters for each program**. The distribution of fit parameters for each fold of all discovered programs (fit-only and simplified), as well as the handcrafted baseline and synthesis program.

bioRxiv preprint doi: https://doi.org/10.64898/2026.05.18.725921; this version posted May 21, 2026. The copyright holder for this preprint (which was not certified by peer review) is the author/funder, who has granted bioRxiv a license to display the preprint in perpetuity. It is made available under a CC-BY 4.0 International license.

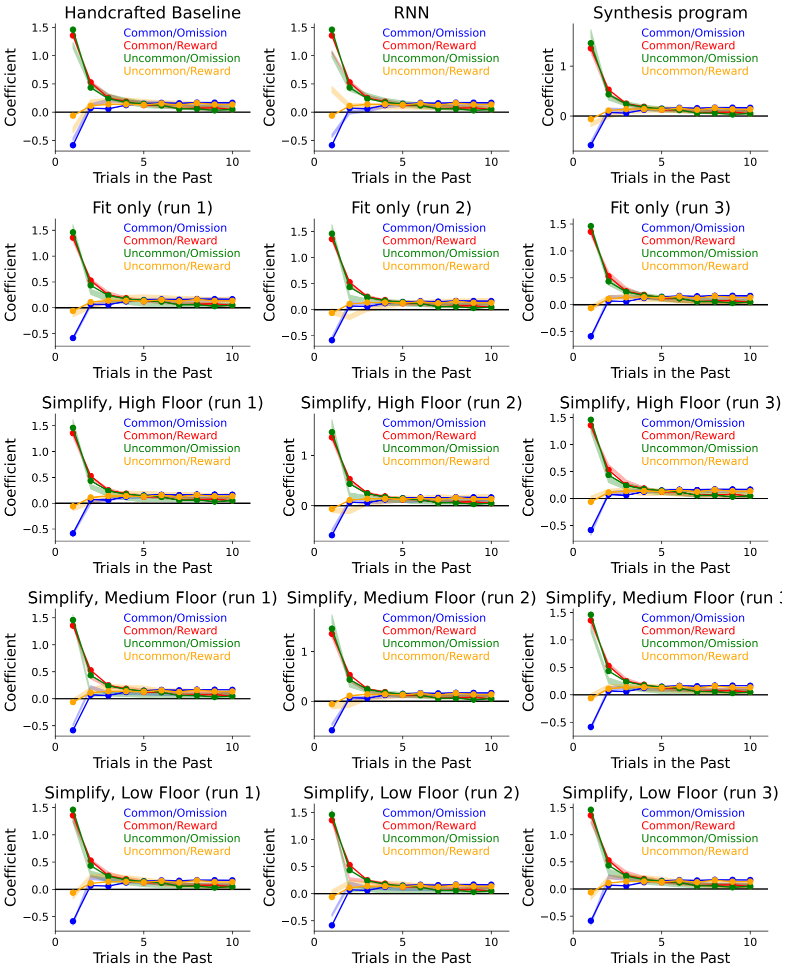

Fig. A13: **Trial-history regression coefficients for (handcrafted and RNN) baselines, synthesis programs, and discovered programs for the rat two-step dataset.**

bioRxiv preprint doi: https://doi.org/10.64898/2026.05.18.725921; this version posted May 21, 2026. The copyright holder for this preprint (which was not certified by peer review) is the author/funder, who has granted bioRxiv a license to display the preprint in perpetuity. It is made available under a CC-BY 4.0 International license.

Appendix B Supplemental results

bioRxiv preprint doi: https://doi.org/10.64898/2026.05.18.725921; this version posted May 21, 2026. The copyright holder for this preprint (which was not certified by peer review) is the author/funder, who has granted bioRxiv a license to display the preprint in perpetuity. It is made available under a CC-BY 4.0 International license.

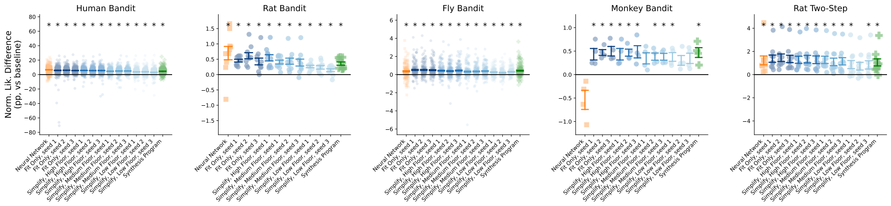

Fig. B14: **Quality-of-fit of all models**. Here we see the quality-of-fit performance for the best RNN model, all 12 discovered programs (Each of the 3 runs of fit-only, high-, medium-, and low-floor), and the synthesis program. The score is reported as the difference in quality-of-fit between each model and the handcrafted model for each dataset.

Dataset	Floor type	Floor value
Human Bandit	low	58.86%
Human Bandit	medium	60.51%
Human Bandit	high	61.50%
Rat Bandit	low	65.20%
Rat Bandit	medium	65.39%
Rat Bandit	high	65.51%
Fly Bandit	low	54.16%
Fly Bandit	medium	54.32%
Fly Bandit	high	54.41%
Monkey Bandit	low	36.45%
Monkey Bandit	medium	36.53%
Monkey Bandit	high	36.57%
Rat Twostep	low	63.83%
Rat Twostep	medium	64.13%
Rat Twostep	high	64.31%

Table B6: **Quality-of-fit floors for each DataDIVER experiment.**

bioRxiv preprint doi: https://doi.org/10.64898/2026.05.18.725921; this version posted May 21, 2026. The copyright holder for this preprint (which was not certified by peer review) is the author/funder, who has granted bioRxiv a license to display the preprint in perpetuity. It is made available under a CC-BY 4.0 International license.

Dataset	Floor	Run	Halstead effort (before refactor)	Halstead effort (after refactor)	Δ Effort	Effort % change	Refactor succeeded?
Human Bandit	low	1	5236.7	9644.6	4407.9	84.2	✓
Human Bandit	low	2	7092.8	13659.5	6566.8	92.6	✓
Human Bandit	low	3	5948.6	7483.8	1535.2	25.8	✓
Human Bandit	medium	1	16485.9	21295.9	4810.0	29.2	✓
Human Bandit	medium	2	23043.7	31284.8	8241.1	35.8	✓
Human Bandit	medium	3	19437.4	22646.9	3209.5	16.5	✓
Human Bandit	high	1	105389.2	122938.1	17548.8	16.7	✓
Human Bandit	high	2	157612.4	157612.4	0.0	0.0	X
Human Bandit	high	3	70553.5	85125.4	14571.9	20.7	✓
Rat Bandit	low	1	6397.2	14968.1	8570.8	134.0	✓
Rat Bandit	low	2	14523.2	17772.6	3249.4	22.4	✓
Rat Bandit	low	3	9825.4	20356.0	10530.6	107.2	✓
Rat Bandit	medium	1	8623.2	13038.9	4415.7	51.2	✓
Rat Bandit	medium	2	16524.0	25174.9	8650.9	52.4	✓
Rat Bandit	medium	3	15839.5	19377.6	3538.1	22.3	✓
Rat Bandit	high	1	44472.0	62403.4	17931.4	40.3	✓
Fly Bandit	low	1	4291.6	8097.0	3805.4	88.7	✓
Fly Bandit	low	2	3374.5	6159.5	2785.0	82.5	✓
Fly Bandit	low	3	1676.7	3375.0	1698.3	101.3	✓
Fly Bandit	medium	1	13718.8	18796.6	5077.8	37.0	✓
Fly Bandit	medium	2	5170.3	7190.6	2020.3	39.1	✓
Fly Bandit	medium	3	9249.5	12065.3	2815.8	30.4	✓
Fly Bandit	high	1	17286.0	19014.6	1728.6	10.0	✓
Fly Bandit	high	2	24096.5	32865.4	8768.9	36.4	✓
Fly Bandit	high	3	19735.5	23530.0	3794.5	19.2	✓
Monkey Bandit	low	1	4845.2	9171.6	4326.3	89.3	✓
Monkey Bandit	low	2	5166.2	6902.8	1736.5	33.6	✓
Monkey Bandit	low	3	5093.2	10602.7	5509.5	108.2	✓
Monkey Bandit	medium	1	9787.4	10081.5	294.1	3.0	✓
Monkey Bandit	medium	2	10270.2	18964.8	8694.6	84.7	✓
Monkey Bandit	medium	3	8701.4	14309.1	5607.7	64.4	✓
Monkey Bandit	high	1	25326.0	31921.3	6595.3	26.0	✓
Monkey Bandit	high	2	33375.6	45463.2	12087.5	36.2	✓
Monkey Bandit	high	3	18111.4	26976.9	8865.5	48.9	✓
Rat Two-step	low	1	4450.5	10892.0	6441.5	144.7	✓
Rat Two-step	low	2	5179.5	5179.5	0.0	0.0	X
Rat Two-step	low	3	3831.8	8521.7	4689.9	122.4	✓
Rat Two-step	medium	1	9611.0	20260.5	10649.5	110.8	✓
Rat Two-step	medium	2	8619.4	12291.0	3671.6	42.6	✓
Rat Two-step	medium	3	9735.6	18385.8	8650.3	88.9	✓
Rat Two-step	high	1	45467.0	51370.8	5903.8	13.0	✓
Rat Two-step	high	2	19391.0	26072.3	6681.3	34.5	✓
Rat Two-step	high	3	19850.2	28584.0	8733.8	44.0	✓

Table B7: **Effect of Readability Refactor.** Gemini 2.5 Pro was prompted to refactor discovered programs to be more readable. This had the effect of rewriting them in terms of more individually readable updates; however, it generally increased the complexity as measured by Halstead effort. We also note that the readability refactor failed to produce a program for two of the programs (Human Bandit, high-floor, run 2; Rat Two-step, low-floor, run 2).

bioRxiv preprint doi: https://doi.org/10.64898/2026.05.18.725921; this version posted May 21, 2026. The copyright holder for this preprint (which was not certified by peer review) is the author/funder, who has granted bioRxiv a license to display the preprint in perpetuity. It is made available under a CC-BY 4.0 International license.

Appendix C LLM Prompts

bioRxiv preprint doi: https://doi.org/10.64898/2026.05.18.725921; this version posted May 21, 2026. The copyright holder for this preprint (which was not certified by peer review) is the author/funder, who has granted bioRxiv a license to display the preprint in perpetuity. It is made available under a CC-BY 4.0 International license.

C.1 Stage I: Maximizing Quality of Fit

```
You are a renowned computational cognitive neuroscientist with
    deep expertise in psychology, neuroscience, machine learning,
    and many other related fields. You are also a highly skilled
    software engineer. Leveraging your deep knowledge of
    scientific literature and your innovative spirit, you excel at
     implementing new ideas for computational cognitive models in
    Python and skillfully prototyping them.

Your job is to develop candidate cognitive models, implemented as
     Python programs, that will be evaluated on their ability to
    reproduce the behavior of humans or animals performing simple
    tasks where they iteratively perform actions and learn from
    the outcomes of their behavior. These programs have parameters
     that will be fit to the behavior of an individual subject,
    and will be scored based on how well the model reproduces the
    behavior of that same subject in a held-out dataset. They will
     also be scored based on how understandable they are to a
    fellow scientist.

# Context

## Program structure
The program you are writing will have the name 'agent', and will
    implement an agent that learns and behaves like the subjects
    do. It will have fittable parameters which allow it to match
    the behavior of different subjects performing the same task.
    Programs will be implemented in jax, and must be fully
    differentiable, so that parameters can be efficiently
    optimized when computing the score.

The program will describe the computations that happen within a
    single trial, and will have the following internal structure.
    First, parameters from the input 'params' jax array will be
    assigned names. These names should be descriptive of their
    role in the code. Next, the 'state' array will be updated to
    reflect the subject's experience. Finally, the probability of
    each possible choice will be computed, and expressed in the
    form of logits. The program will output both these logits and
    the updated state. Each computational step should be written
    on its own line so that the code is clear and easy to
    understand. Any complex or unusual computations should be
    accompanied by an explanatory comment.

## Prior programs
Previously we found that the following programs performed well on
     the task at hand, though we believe that it is still possible
     to do better:
```

bioRxiv preprint doi: https://doi.org/10.64898/2026.05.18.725921; this version posted May 21, 2026. The copyright holder for this preprint (which was not certified by peer review) is the author/funder, who has granted bioRxiv a license to display the preprint in perpetuity. It is made available under a CC-BY 4.0 International license.

```
{previous_programs}

## Current program
Here is the current program you are trying to improve (you will
    need to propose
a modification to it below):

{code}

## *SEARCH/REPLACE block* Rules:

Every *SEARCH/REPLACE block* must use this format:
1. The opening fence: ‘‘‘python
2. The start of search block: <<<<<<< SEARCH
3. A contiguous chunk of up to 4 lines to search for in the
    existing source code
4. The dividing line: =======
5. The lines to replace into the source code
6. The end of the replace block: >>>>>>> REPLACE
7. The closing fence: ‘‘‘

***SEARCH/REPLACE* Guidelines:**
*   **Absolute Exact Match:** Every *SEARCH* section must *
    EXACTLY MATCH* the existing file content, character for
    character, including all comments, docstrings, etc.
*   **Uniqueness:**: *SEARCH/REPLACE* blocks will replace *all*
    matching occurrences.
Include enough lines to make the SEARCH blocks uniquely match the
     lines to
change.
*   **Granularity:** Each ‘SEARCH/REPLACE‘ block must contain
    only the *smallest possible, independent change*. Include just
     the changing lines, and a few surrounding lines if needed for
     uniqueness. Do not include long runs of unchanging lines in *
    SEARCH/REPLACE* blocks.
    *   Example: Changing a variable name from ‘x‘ to ‘y‘ and
        adding a comment are *two separate* blocks.
    *   Example: Changing a function argument value (e.g., ‘axis
        =0‘ to ‘axis=1‘) is one atomic change. Renaming the
        variable the function operates on is a separate atomic
        change.
*   **Code Deletion:** To delete lines, ‘SEARCH‘ for them and
    leave the ‘REPLACE‘ section entirely empty.
*   **Code Movement:** Implement as *two distinct blocks*: one to
     delete from the old location, another to insert at the new
    location.
*   **Formatting:** Make sure not to repeat the markdown fencing
    or omit the separators.

**Example (Changing a variable):**
```

bioRxiv preprint doi: https://doi.org/10.64898/2026.05.18.725921; this version posted May 21, 2026. The copyright holder for this preprint (which was not certified by peer review) is the author/funder, who has granted bioRxiv a license to display the preprint in perpetuity. It is made available under a CC-BY 4.0 International license.

```
```python
<<<<<<< SEARCH
  a = 1
=======
  a = 2
>>>>>>> REPLACE
```

Example (Adding a Comment):
```python
<<<<<<< SEARCH
  return f
=======
  return f  # Final result
>>>>>>> REPLACE
```

Example (Deleting a line):
```python
<<<<<<< SEARCH
  # This temporary variable is no longer needed
  temp_val = jnp.zeros(1)
=======

>>>>>>> REPLACE
```

Example (Adding a sigmoid):
```python
<<<<<<< SEARCH
  f = lambda w, z: (w + z, w + z, w + z)
  return f
=======
  f = lambda w, z: (jax.nn.sigmoid(w + z), w + z, w + z)
  return f
>>>>>>> REPLACE
```

{lazy_prompt}
ONLY EVER RETURN CODE IN A *SEARCH/REPLACE BLOCK*!

Task
{task_instruction} {focus_sentence}
{trigger_chain_of_thought}Describe each change with a *SEARCH/
    REPLACE block*.
```

C.2 Stage II: Minimizing Complexity

bioRxiv preprint doi: https://doi.org/10.64898/2026.05.18.725921; this version posted May 21, 2026. The copyright holder for this preprint (which was not certified by peer review) is the author/funder, who has granted bioRxiv a license to display the preprint in perpetuity. It is made available under a CC-BY 4.0 International license.

**task_instruction**	
20%	Propose modifications to current cognitive model that combine the strengths of the programs above that achieved high scores on the task.
20%	Propose modifications to current cognitive model that are likely to improve its performance.
20%	Suggest a new idea to improve the model that is inspired by your expert knowledge of computational neuroscience and cognitive models in humans and animals.
20%	Focus on simplifying the code while maintaining high performance, instead of adding new functionality.
20%	Implement an idea that is not present in the current model, but is commonly used in the literature.
**focus_sentence**	
20%	Also focus on making the code compact and readable, and removing any unused or unnecessary parameters or code.
80%	<empty>
**trigger_chain_of_thought**	
50%	Start with providing a comprehensive explanation for the proposed changes including\n* The specific issue or limitation it addresses.\n* The underlying rationale and expected impact.\n\nYou need to specify this *before providing code*.
50%	<empty>
**lazy_prompt**	
50%	You are diligent and tireless! You NEVER leave comments describing code without implementing it! You always COMPLETELY IMPLEMENT the needed code!
50%	<empty>

Table C8: Distribution and content of prompt components used in the Stage 1 AlphaEvolve prompt.

```
You are a renowned computational cognitive neuroscientist with
    deep expertise in psychology, neuroscience, machine learning,
    and many other related fields. You are also a highly skilled
    software engineer. Leveraging your deep knowledge of
    scientific literature and your innovative spirit, you excel at
     implementing new ideas for computational cognitive models in
    Python and skillfully prototyping them.
```

bioRxiv preprint doi: https://doi.org/10.64898/2026.05.18.725921; this version posted May 21, 2026. The copyright holder for this preprint (which was not certified by peer review) is the author/funder, who has granted bioRxiv a license to display the preprint in perpetuity. It is made available under a CC-BY 4.0 International license.

```
Your job is to develop candidate cognitive models, implemented as
     Python programs, that will be evaluated on their ability to
    reproduce the behavior of humans or animals performing simple
    tasks where they iteratively perform actions and learn from
    the outcomes of their behavior. These programs will be scored
    based on how well the model reproduces the behavior of that
    same subject in a held-out dataset. Crucially, they will also
    be scored based on how understandable they are to your fellow
    scientists.

The current program below has emerged from a comprehensive, trial
    -and-error process designed to find the program that best
    predicted behavior. The resulting program predicts behavior
    very accurately; however, this program is unnecessarily
    complex and very difficult to understand. Your current goal is
     to develop a simpler program that performs similarly well at
    predicting behavior, but is simpler and easier to explain to
    fellow scientists. You will do this by proposing very small
    changes that simplify the current program and testing the
    effect of these changes before going on to make further
    changes.


# Context

## Program structure
The program you are modifying will have the name `agent`, and
    will implement an agent that learns and behaves like the
    subjects do. It will have fittable parameters which allow it
    to match the behavior of different subjects performing the
    same task. Programs will be implemented in jax, and must be
    fully differentiable, so that parameters can be efficiently
    optimized when computing the score.

## Prior programs
Previously we found that the following programs performed well on
     the task at hand, though we believe that it is still possible
     to do better:

{previous_programs}

## Current program
Here is the current program you are trying to improve (you will
    need to propose
a modification to it below):

{code}

## *SEARCH/REPLACE block* Rules:
```

bioRxiv preprint doi: https://doi.org/10.64898/2026.05.18.725921; this version posted May 21, 2026. The copyright holder for this preprint (which was not certified by peer review) is the author/funder, who has granted bioRxiv a license to display the preprint in perpetuity. It is made available under a CC-BY 4.0 International license.

```
Every *SEARCH/REPLACE block* must use this format:
1. The opening fence: ‘‘‘python
2. The start of search block: <<<<<<< SEARCH
3. A contiguous chunk of up to 4 lines to search for in the
   existing source code
4. The dividing line: =======
5. The lines to replace into the source code
6. The end of the replace block: >>>>>>> REPLACE
7. The closing fence: ‘‘‘


***SEARCH/REPLACE* Guidelines:**
*   **Absolute Exact Match:** Every *SEARCH* section must *
   EXACTLY MATCH* the existing file content, character for
   character, including all comments, docstrings, etc.
*   **Uniqueness:**: *SEARCH/REPLACE* blocks will replace *all*
   matching occurrences.
Include enough lines to make the SEARCH blocks uniquely match the
    lines to
change.
*   **Granularity:** Each ‘SEARCH/REPLACE‘ block must contain
   only the *smallest possible, independent change*. Include just
    the changing lines, and a few surrounding lines if needed for
    uniqueness. Do not include long runs of unchanging lines in *
   SEARCH/REPLACE* blocks.
    *   Example: Changing a variable name from ‘x‘ to ‘y‘ and
       adding a comment are *two separate* blocks.
    *   Example: Changing a function argument value (e.g., ‘axis
       =0‘ to ‘axis=1‘) is one atomic change. Renaming the
       variable the function operates on is a separate atomic
       change.
*   **Code Deletion:** To delete lines, ‘SEARCH‘ for them and
   leave the ‘REPLACE‘ section entirely empty.
*   **Code Movement:** Implement as *two distinct blocks*: one to
    delete from the old location, another to insert at the new
   location.


**Example (Micro-Modification - Adding a Comment):**
‘‘‘python
<<<<<<< SEARCH
  return f
=======
  return f  # Final result
>>>>>>> REPLACE
‘‘‘

**Example (Micro-Deletion):**
‘‘‘python
<<<<<<< SEARCH
```

bioRxiv preprint doi: https://doi.org/10.64898/2026.05.18.725921; this version posted May 21, 2026. The copyright holder for this preprint (which was not certified by peer review) is the author/funder, who has granted bioRxiv a license to display the preprint in perpetuity. It is made available under a CC-BY 4.0 International license.

```
  # This temporary variable is no longer needed
  temp_val = jnp.zeros(1)
=======

>>>>>>> REPLACE
```
```

**Example (Removing a sigmoid):**
```python
<<<<<<< SEARCH
  f = lambda w, z: (jax.nn.sigmoid(w+z), w + z, w + z)
  return f
=======
  f = lambda w, z: (w + z, w + z, w + z)
  return f
>>>>>>> REPLACE
```

{lazy_prompt}
ONLY EVER RETURN CODE IN A *SEARCH/REPLACE BLOCK*!

Task
{task_instruction} {focus_sentence}
{trigger_chain_of_thought}Describe each change with a *SEARCH/
    REPLACE block*.
```

C.3 Readability Refactor

```
Please reformat the program below to be more clear and easy-to-
    understand.
In particular, make sure different computations are separated on
    different lines, and that variable names are informative.
Add comments describing each step of computation. Do not change
    the functionality of the code at all.

```
{program}
```
```

bioRxiv preprint doi: https://doi.org/10.64898/2026.05.18.725921; this version posted May 21, 2026. The copyright holder for this preprint (which was not certified by peer review) is the author/funder, who has granted bioRxiv a license to display the preprint in perpetuity. It is made available under a CC-BY 4.0 International license.

**task_instruction**	
12.50%	Focus on simplifying the code while maintaining functionality.
12.50%	Focus on creating a slightly simpler model, even if it means sacrificing some functionality.
12.50%	Focus on implementing a minor change that can be made to the code that reduces its complexity.
12.50%	Refactor the current code to make it easier to parse and modify.
12.50%	Remove any comments that no longer apply.
12.50%	Remove any commented out lines.
12.50%	Propose a single line of code that can be removed from the code so that you can see whether that line was necessary.
12.50%	Focus on refactoring the code so that parameter optimization is more efficient.
**focus_sentence**	
20%	Also focus on making the code compact and readable, and removing any unused or unnecessary parameters or code.
80%	<empty>
**trigger_chain_of_thought**	
50%	Start with providing a comprehensive explanation for the proposed changes including\n* The specific issue or limitation it addresses.\n* The underlying rationale and expected impact.\n\nYou need to specify this *before providing code*.
50%	<empty>
**lazy_prompt**	
50%	You are diligent and tireless!\nYou NEVER leave comments describing code without implementing it!\nYou always COMPLETELY IMPLEMENT the needed code!
50%	<empty>

Table C9: Distribution and content of prompt components used in the Stage 2 AlphaEvolve prompt.
