# Supplementary material for: AI-Discovered Cognitive Models Reveal Novel Insights into Human and Animal Learning
Source: bioRxiv. 2026 May 21:2026.05.18.725921. Preprint. [Version 1] doi: 10.64898/2026.05.18.725921 (PMC13228651; doi:10.64898/2026.05.18.725921)
Supplement: Supplement 2 [file ablation_performance_human_bandit_run3_high_floor_refactored_20260420.pdf › ablation_performance_human_bandit_run3_high_floor_refactored_20260420.pdf]

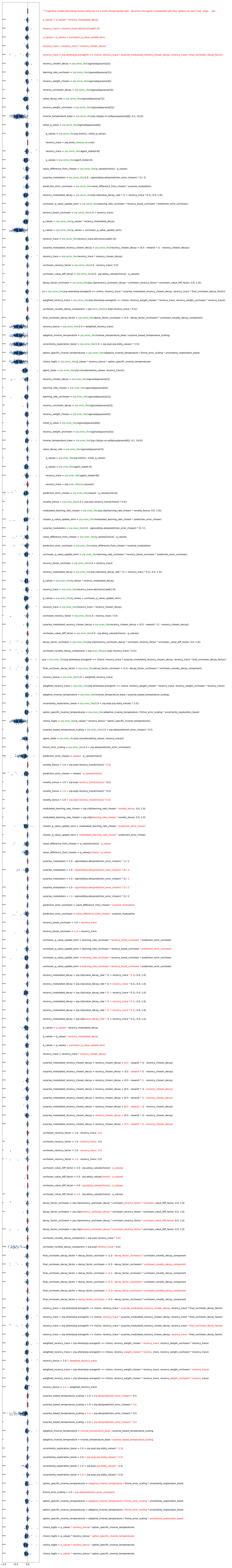

−1.0
−0.5
0.0
