# Supplementary material for: AI-Discovered Cognitive Models Reveal Novel Insights into Human and Animal Learning
Source: bioRxiv. 2026 May 21:2026.05.18.725921. Preprint. [Version 1] doi: 10.64898/2026.05.18.725921 (PMC13228651; doi:10.64898/2026.05.18.725921)
Supplement: Supplement 2 [file ablation_performance_human_bandit_run1_high_floor_refactored_20260420.pdf › ablation_performance_human_bandit_run1_high_floor_refactored_20260420.pdf]

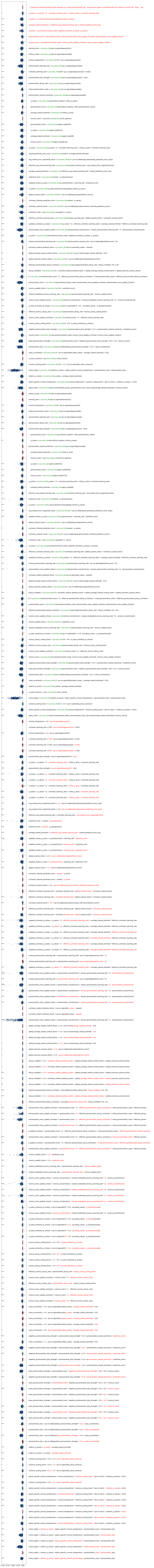
"""Cognitive model describing human behavior on a multi-armed bandit task. Assumes the agent is presented with four options on each trial. Args: par…
q_values = q_values * (1 - unchosen_learning_rate) + initial_q_value * unchosen_learning_rate
q_values = q_values.at[choice].set(updated_chosen_q_value)
average_reward_estimate += effective_avg_reward_learning_rate * reward_prediction_error_avg
q_values = jnp.where(unchosen_mask, updated_unchosen_q_values, q_values)
perseveration_trace = jnp.where(unchosen_mask, perseveration_trace_update_unchosen, perseveration_trace_update_chosen)
recency_trace = jnp.where(unchosen_mask, recency_trace_update_unchosen, recency_trace_update_chosen)
learning_rate = jnp.zeros_like(jax.nn.sigmoid(params[1]))
initial_q_value = jnp.zeros_like(jax.nn.sigmoid(params[0]))
inverse_temperature = jnp.zeros_like(10 * jax.nn.sigmoid(params[2]))
perseveration_learning_rate = jnp.zeros_like(jax.nn.sigmoid(params[4]))
unchosen_learning_rate = jnp.zeros_like(0.95 * jax.nn.sigmoid(params[3]) + 0.025)
perseveration_bias_strength = jnp.zeros_like(jax.nn.sigmoid(params[7]) * 10.0)
perseveration_decay_rate = jnp.zeros_like(jax.nn.sigmoid(params[5]))
initial_perseveration_value = jnp.zeros_like(jax.nn.sigmoid(params[6]))
perseveration_reward_sensitivity = jnp.zeros_like(jax.nn.sigmoid(params[8]))
q_values = jnp.zeros_like(jnp.full(num_options, initial_q_value))
perseveration_trace = jnp.zeros_like(jnp.full(num_options, initial_perseveration_value))
average_reward_estimate = jnp.zeros_like(initial_q_value)
recency_trace = jnp.zeros_like(jnp.zeros(num_options))
perseveration_trace = jnp.zeros_like(agent_state[4:8])
q_values = jnp.zeros_like(agent_state[0:4])
average_reward_estimate = jnp.zeros_like(agent_state[8])
recency_trace = jnp.zeros_like(agent_state[9:13])
q_values = jnp.zeros_like(q_values * (1 - unchosen_learning_rate) + initial_q_value * unchosen_learning_rate)
reward_prediction_error_avg = jnp.zeros_like(reward - average_reward_estimate)
avg_reward_error_magnitude_factor = jnp.zeros_like(1.0 + jax.nn.softplus(jnp.abs(reward_prediction_error_avg)))
effective_avg_reward_learning_rate = jnp.zeros_like(unchosen_learning_rate * avg_reward_error_magnitude_factor)
average_reward_estimate += jnp.zeros_like(effective_avg_reward_learning_rate * reward_prediction_error_avg)
prediction_error = jnp.zeros_like(reward - q_values[choice])
updated_chosen_q_value = jnp.zeros_like(q_values[choice] + learning_rate * prediction_error)
q_values = jnp.zeros_like(q_values.at[choice].set(updated_chosen_q_value))
global_surprise_factor = jnp.zeros_like(1.0 + jax.nn.softplus(jnp.abs(prediction_error)))
unchosen_inferred_prediction_error = jnp.zeros_like(reward - q_values)
unchosen_surprise_factor = jnp.zeros_like(1.0 + jax.nn.softplus(jnp.abs(unchosen_inferred_prediction_error)))
unchosen_mask = jnp.zeros_like(jnp.arange(4) != choice)
effective_unchosen_learning_rate = jnp.zeros_like(unchosen_learning_rate * global_surprise_factor * unchosen_surprise_factor)
updated_unchosen_q_values = jnp.zeros_like(q_values * (1 - effective_unchosen_learning_rate) + average_reward_estimate * effective_unchosen_learning_rate)
perseveration_trace_update_chosen = jnp.zeros_like(perseveration_trace[choice] + chosen_perseveration_learning_rate * (1 - perseveration_trace[choice]))
q_values = jnp.zeros_like(jnp.where(unchosen_mask, updated_unchosen_q_values, q_values))
chosen_perseveration_learning_rate = jnp.zeros_like(perseveration_learning_rate * jax.nn.sigmoid(prediction_error * 5))
unchosen_relative_badness_factor = jnp.zeros_like(jax.nn.sigmoid(q_values - reward))
global_average_reward_context_factor = jnp.zeros_like(1.0 + jax.nn.relu(average_reward_estimate - 0.5))
global_outcome_surprise_factor = jnp.zeros_like(1.0 + jax.nn.softplus(jnp.abs(prediction_error)))
effective_perseveration_decay_unchosen = jnp.zeros_like(jnp.clip(perseveration_decay_rate * decay_multiplier, 0.0, 1.0))
decay_multiplier = jnp.zeros_like(1.0 + unchosen_relative_badness_factor + global_average_reward_context_factor + global_outcome_surprise_factor)
…n = jnp.zeros_like(perseveration_trace * (1 - effective_perseveration_decay_unchosen) + initial_perseveration_value * effective_perseveration_decay_unchosen)
perseveration_trace = jnp.zeros_like(jnp.where(unchosen_mask, perseveration_trace_update_unchosen, perseveration_trace_update_chosen))
recency_update_factor = jnp.zeros_like(1.0 + prediction_error)
reward_modulated_recency_learning_rate = jnp.zeros_like(perseveration_learning_rate * recency_update_factor)
recency_trace_update_chosen = jnp.zeros_like(recency_trace[choice] + reward_modulated_recency_learning_rate * (1 - recency_trace[choice]))
q_value_similarity_to_chosen = jnp.zeros_like(jax.nn.sigmoid(5.0 * (1.0 - jnp.abs(q_values - q_values[choice]))))
effective_recency_decay_rate = jnp.zeros_like(perseveration_decay_rate * recency_decay_scaling_factor)
recency_trace_update_unchosen = jnp.zeros_like(recency_trace * (1 - effective_recency_decay_rate))
recency_decay_scaling_factor = jnp.zeros_like(1.0 - 0.9 * q_value_similarity_to_chosen)
adaptive_perseveration_bias_strength = jnp.zeros_like(perseveration_bias_strength * (1.0 + perseveration_reward_sensitivity * prediction_error))
recency_trace = jnp.zeros_like(jnp.where(unchosen_mask, recency_trace_update_unchosen, recency_trace_update_chosen))
base_perseveration_strength = jnp.zeros_like(perseveration_trace * adaptive_perseveration_bias_strength * (0.5 + 0.5 * recency_trace))
perseveration_bias = jnp.zeros_like(jax.nn.softplus(base_perseveration_strength * (1.0 + value_uncertainty)))
value_uncertainty = jnp.zeros_like(1.0 - jax.nn.sigmoid(jnp.abs(q_values - average_reward_estimate) * 5.0))
q_value_variance = jnp.zeros_like(jnp.var(q_values))
variance_scaling_factor = jnp.zeros_like(0.5 + 1.5 * jax.nn.sigmoid(q_value_variance))
choice_logits = jnp.zeros_like(relative_q_values * option_specific_inverse_temperature + perseveration_trace * perseveration_bias)
relative_q_values = jnp.zeros_like(q_values - average_reward_estimate)
option_specific_inverse_temperature = jnp.zeros_like(inverse_temperature * variance_scaling_factor * (jax.nn.relu(1 + relative_q_values) + 0.01))
agent_state = jnp.zeros_like(jnp.concatenate([q_values, perseveration_trace, jnp.array([average_reward_estimate]), recency_trace]))
initial_q_value = jnp.ones_like(jax.nn.sigmoid(params[0]))
learning_rate = jnp.ones_like(jax.nn.sigmoid(params[1]))
inverse_temperature = jnp.ones_like(10 * jax.nn.sigmoid(params[2]))
initial_perseveration_value = jnp.ones_like(jax.nn.sigmoid(params[6]))
perseveration_learning_rate = jnp.ones_like(jax.nn.sigmoid(params[4]))
perseveration_decay_rate = jnp.ones_like(jax.nn.sigmoid(params[5]))
perseveration_bias_strength = jnp.ones_like(jax.nn.sigmoid(params[7]) * 10.0)
perseveration_trace = jnp.ones_like(jnp.full(num_options, initial_perseveration_value))
q_values = jnp.ones_like(jnp.full(num_options, initial_q_value))
perseveration_reward_sensitivity = jnp.ones_like(jax.nn.sigmoid(params[8]))
average_reward_estimate = jnp.ones_like(initial_q_value)
recency_trace = jnp.ones_like(jnp.zeros(num_options))
q_values = jnp.ones_like(agent_state[0:4])
perseveration_trace = jnp.ones_like(agent_state[4:8])
recency_trace = jnp.ones_like(agent_state[9:13])
q_values = jnp.ones_like(q_values * (1 - unchosen_learning_rate) + initial_q_value * unchosen_learning_rate)
average_reward_estimate = jnp.ones_like(agent_state[8])
effective_avg_reward_learning_rate = jnp.ones_like(unchosen_learning_rate * avg_reward_error_magnitude_factor)
prediction_error = jnp.ones_like(reward - q_values[choice])
q_values = jnp.ones_like(q_values.at[choice].set(updated_chosen_q_value))
avg_reward_error_magnitude_factor = jnp.ones_like(1.0 + jax.nn.softplus(jnp.abs(reward_prediction_error_avg)))
updated_chosen_q_value = jnp.ones_like(q_values[choice] + learning_rate * prediction_error)
global_surprise_factor = jnp.ones_like(1.0 + jax.nn.softplus(jnp.abs(prediction_error)))
unchosen_inferred_prediction_error = jnp.ones_like(reward - q_values)
unchosen_surprise_factor = jnp.ones_like(1.0 + jax.nn.softplus(jnp.abs(unchosen_inferred_prediction_error)))
unchosen_mask = jnp.ones_like(jnp.arange(4) != choice)
q_values = jnp.ones_like(jnp.where(unchosen_mask, updated_unchosen_q_values, q_values))
effective_unchosen_learning_rate = jnp.ones_like(unchosen_learning_rate * global_surprise_factor * unchosen_surprise_factor)
updated_unchosen_q_values = jnp.ones_like(q_values * (1 - effective_unchosen_learning_rate) + average_reward_estimate * effective_unchosen_learning_rate)
chosen_perseveration_learning_rate = jnp.ones_like(perseveration_learning_rate * jax.nn.sigmoid(prediction_error * 5))
perseveration_trace_update_chosen = jnp.ones_like(perseveration_trace[choice] + chosen_perseveration_learning_rate * (1 - perseveration_trace[choice]))
unchosen_relative_badness_factor = jnp.ones_like(jax.nn.sigmoid(q_values - reward))
global_average_reward_context_factor = jnp.ones_like(1.0 + jax.nn.relu(average_reward_estimate - 0.5))
global_outcome_surprise_factor = jnp.ones_like(1.0 + jax.nn.softplus(jnp.abs(prediction_error)))
decay_multiplier = jnp.ones_like(1.0 + unchosen_relative_badness_factor + global_average_reward_context_factor + global_outcome_surprise_factor)
…en = jnp.ones_like(perseveration_trace * (1 - effective_perseveration_decay_unchosen) + initial_perseveration_value * effective_perseveration_decay_unchosen)
perseveration_trace = jnp.ones_like(jnp.where(unchosen_mask, perseveration_trace_update_unchosen, perseveration_trace_update_chosen))
effective_perseveration_decay_unchosen = jnp.ones_like(jnp.clip(perseveration_decay_rate * decay_multiplier, 0.0, 1.0))
recency_trace_update_chosen = jnp.ones_like(recency_trace[choice] + reward_modulated_recency_learning_rate * (1 - recency_trace[choice]))
recency_update_factor = jnp.ones_like(1.0 + prediction_error)
reward_modulated_recency_learning_rate = jnp.ones_like(perseveration_learning_rate * recency_update_factor)
q_value_similarity_to_chosen = jnp.ones_like(jax.nn.sigmoid(5.0 * (1.0 - jnp.abs(q_values - q_values[choice]))))
recency_decay_scaling_factor = jnp.ones_like(1.0 - 0.9 * q_value_similarity_to_chosen)
effective_recency_decay_rate = jnp.ones_like(perseveration_decay_rate * recency_decay_scaling_factor)
recency_trace_update_unchosen = jnp.ones_like(recency_trace * (1 - effective_recency_decay_rate))
recency_trace = jnp.ones_like(jnp.where(unchosen_mask, recency_trace_update_unchosen, recency_trace_update_chosen))
adaptive_perseveration_bias_strength = jnp.ones_like(perseveration_bias_strength * (1.0 + perseveration_reward_sensitivity * prediction_error))
base_perseveration_strength = jnp.ones_like(perseveration_trace * adaptive_perseveration_bias_strength * (0.5 + 0.5 * recency_trace))
perseveration_bias = jnp.ones_like(jax.nn.softplus(base_perseveration_strength * (1.0 + value_uncertainty)))
value_uncertainty = jnp.ones_like(1.0 - jax.nn.sigmoid(jnp.abs(q_values - average_reward_estimate) * 5.0))
relative_q_values = jnp.ones_like(q_values - average_reward_estimate)
q_value_variance = jnp.ones_like(jnp.var(q_values))
choice_logits = jnp.ones_like(relative_q_values * option_specific_inverse_temperature + perseveration_trace * perseveration_bias)
variance_scaling_factor = jnp.ones_like(0.5 + 1.5 * jax.nn.sigmoid(q_value_variance))
option_specific_inverse_temperature = jnp.ones_like(inverse_temperature * variance_scaling_factor * (jax.nn.relu(1 + relative_q_values) + 0.01))
agent_state = jnp.ones_like(jnp.concatenate([q_values, perseveration_trace, jnp.array([average_reward_estimate]), recency_trace]))
inverse_temperature = 10 * jax.nn.sigmoid(params[2])
unchosen_learning_rate = 0.95 * jax.nn.sigmoid(params[3]) + 0.025
inverse_temperature = 10 * jax.nn.sigmoid(params[2])
unchosen_learning_rate = 0.95 * jax.nn.sigmoid(params[3]) + 0.025
unchosen_learning_rate = 0.95 * jax.nn.sigmoid(params[3]) + 0.025
unchosen_learning_rate = 0.95 * jax.nn.sigmoid(params[3]) + 0.025
perseveration_bias_strength = jax.nn.sigmoid(params[7]) * 10.0
q_values = q_values * (1 - unchosen_learning_rate) + initial_q_value * unchosen_learning_rate
perseveration_bias_strength = jax.nn.sigmoid(params[7]) * 10.0
q_values = q_values * (1 - unchosen_learning_rate) + initial_q_value * unchosen_learning_rate
q_values = q_values * (1 - unchosen_learning_rate) + initial_q_value * unchosen_learning_rate
q_values = q_values * (1 - unchosen_learning_rate) + initial_q_value * unchosen_learning_rate
q_values = q_values * (1 - unchosen_learning_rate) + initial_q_value * unchosen_learning_rate
q_values = q_values * (1 - unchosen_learning_rate) + initial_q_value * unchosen_learning_rate
avg_reward_error_magnitude_factor = 1.0 + jax.nn.softplus(jnp.abs(reward_prediction_error_avg))
avg_reward_error_magnitude_factor = 1.0 + jax.nn.softplus(jnp.abs(reward_prediction_error_avg))
effective_avg_reward_learning_rate = unchosen_learning_rate * avg_reward_error_magnitude_factor
prediction_error = reward - q_values[choice]
prediction_error = reward - q_values[choice]
average_reward_estimate += effective_avg_reward_learning_rate * reward_prediction_error_avg
updated_chosen_q_value = q_values[choice] + learning_rate * prediction_error
updated_chosen_q_value = q_values[choice] + learning_rate * prediction_error
updated_chosen_q_value = q_values[choice] + learning_rate * prediction_error
global_surprise_factor = 1.0 + jax.nn.softplus(jnp.abs(prediction_error))
updated_chosen_q_value = q_values[choice] + learning_rate * prediction_error
global_surprise_factor = 1.0 + jax.nn.softplus(jnp.abs(prediction_error))
unchosen_inferred_prediction_error = reward - q_values
unchosen_inferred_prediction_error = reward - q_values
unchosen_surprise_factor = 1.0 + jax.nn.softplus(jnp.abs(unchosen_inferred_prediction_error))
effective_unchosen_learning_rate = unchosen_learning_rate * global_surprise_factor * unchosen_surprise_factor
effective_unchosen_learning_rate = unchosen_learning_rate * global_surprise_factor * unchosen_surprise_factor
unchosen_surprise_factor = 1.0 + jax.nn.softplus(jnp.abs(unchosen_inferred_prediction_error))
effective_unchosen_learning_rate = unchosen_learning_rate * global_surprise_factor * unchosen_surprise_factor
effective_unchosen_learning_rate = unchosen_learning_rate * global_surprise_factor * unchosen_surprise_factor
updated_unchosen_q_values = q_values * (1 - effective_unchosen_learning_rate) + average_reward_estimate * effective_unchosen_learning_rate
updated_unchosen_q_values = q_values * (1 - effective_unchosen_learning_rate) + average_reward_estimate * effective_unchosen_learning_rate
updated_unchosen_q_values = q_values * (1 - effective_unchosen_learning_rate) + average_reward_estimate * effective_unchosen_learning_rate
updated_unchosen_q_values = q_values * (1 - effective_unchosen_learning_rate) + average_reward_estimate * effective_unchosen_learning_rate
updated_unchosen_q_values = q_values * (1 - effective_unchosen_learning_rate) + average_reward_estimate * effective_unchosen_learning_rate
updated_unchosen_q_values = q_values * (1 - effective_unchosen_learning_rate) + average_reward_estimate * effective_unchosen_learning_rate
chosen_perseveration_learning_rate = perseveration_learning_rate * jax.nn.sigmoid(prediction_error * 5)
chosen_perseveration_learning_rate = perseveration_learning_rate * jax.nn.sigmoid(prediction_error * 5)
updated_unchosen_q_values = q_values * (1 - effective_unchosen_learning_rate) + average_reward_estimate * effective_unchosen_learning_rate
perseveration_trace_update_chosen = perseveration_trace[choice] + chosen_perseveration_learning_rate * (1 - perseveration_trace[choice])
chosen_perseveration_learning_rate = perseveration_learning_rate * jax.nn.sigmoid(prediction_error * 5)
perseveration_trace_update_chosen = perseveration_trace[choice] + chosen_perseveration_learning_rate * (1 - perseveration_trace[choice])
perseveration_trace_update_chosen = perseveration_trace[choice] + chosen_perseveration_learning_rate * (1 - perseveration_trace[choice])
perseveration_trace_update_chosen = perseveration_trace[choice] + chosen_perseveration_learning_rate * (1 - perseveration_trace[choice])
perseveration_trace_update_chosen = perseveration_trace[choice] + chosen_perseveration_learning_rate * (1 - perseveration_trace[choice])
unchosen_relative_badness_factor = jax.nn.sigmoid(q_values - reward)
unchosen_relative_badness_factor = jax.nn.sigmoid(q_values - reward)
perseveration_trace_update_chosen = perseveration_trace[choice] + chosen_perseveration_learning_rate * (1 - perseveration_trace[choice])
global_average_reward_context_factor = 1.0 + jax.nn.relu(average_reward_estimate - 0.5)
global_average_reward_context_factor = 1.0 + jax.nn.relu(average_reward_estimate - 0.5)
global_average_reward_context_factor = 1.0 + jax.nn.relu(average_reward_estimate - 0.5)
global_average_reward_context_factor = 1.0 + jax.nn.relu(average_reward_estimate - 0.5)
global_outcome_surprise_factor = 1.0 + jax.nn.softplus(jnp.abs(prediction_error))
global_outcome_surprise_factor = 1.0 + jax.nn.softplus(jnp.abs(prediction_error))
decay_multiplier = 1.0 + unchosen_relative_badness_factor + global_average_reward_context_factor + global_outcome_surprise_factor
decay_multiplier = 1.0 + unchosen_relative_badness_factor + global_average_reward_context_factor + global_outcome_surprise_factor
decay_multiplier = 1.0 + unchosen_relative_badness_factor + global_average_reward_context_factor + global_outcome_surprise_factor
decay_multiplier = 1.0 + unchosen_relative_badness_factor + global_average_reward_context_factor + global_outcome_surprise_factor
decay_multiplier = 1.0 + unchosen_relative_badness_factor + global_average_reward_context_factor + global_outcome_surprise_factor
effective_perseveration_decay_unchosen = jnp.clip(perseveration_decay_rate * decay_multiplier, 0.0, 1.0)
decay_multiplier = 1.0 + unchosen_relative_badness_factor + global_average_reward_context_factor + global_outcome_surprise_factor
effective_perseveration_decay_unchosen = jnp.clip(perseveration_decay_rate * decay_multiplier, 0.0, 1.0)
perseveration_trace_update_unchosen = perseveration_trace * (1 - effective_perseveration_decay_unchosen) + initial_perseveration_value * effective_persev…
perseveration_trace_update_unchosen = perseveration_trace * (1 - effective_perseveration_decay_unchosen) + initial_perseveration_value * effective_persev…
perseveration_trace_update_unchosen = perseveration_trace * (1 - effective_perseveration_decay_unchosen) + initial_perseveration_value * effective_persev…
perseveration_trace_update_unchosen = perseveration_trace * (1 - effective_perseveration_decay_unchosen) + initial_perseveration_value * effective_persev…
…e_update_unchosen = perseveration_trace * (1 - effective_perseveration_decay_unchosen) + initial_perseveration_value * effective_perseveration_decay_unchosen
perseveration_trace_update_unchosen = perseveration_trace * (1 - effective_perseveration_decay_unchosen) + initial_perseveration_value * effective_persev…
…e_update_unchosen = perseveration_trace * (1 - effective_perseveration_decay_unchosen) + initial_perseveration_value * effective_perseveration_decay_unchosen
perseveration_trace_update_unchosen = perseveration_trace * (1 - effective_perseveration_decay_unchosen) + initial_perseveration_value * effective_persev…
recency_update_factor = 1.0 + prediction_error
recency_update_factor = 1.0 + prediction_error
reward_modulated_recency_learning_rate = perseveration_learning_rate * recency_update_factor
reward_modulated_recency_learning_rate = perseveration_learning_rate * recency_update_factor
recency_trace_update_chosen = recency_trace[choice] + reward_modulated_recency_learning_rate * (1 - recency_trace[choice])
recency_trace_update_chosen = recency_trace[choice] + reward_modulated_recency_learning_rate * (1 - recency_trace[choice])
recency_trace_update_chosen = recency_trace[choice] + reward_modulated_recency_learning_rate * (1 - recency_trace[choice])
recency_trace_update_chosen = recency_trace[choice] + reward_modulated_recency_learning_rate * (1 - recency_trace[choice])
recency_trace_update_chosen = recency_trace[choice] + reward_modulated_recency_learning_rate * (1 - recency_trace[choice])
q_value_similarity_to_chosen = jax.nn.sigmoid(5.0 * (1.0 - jnp.abs(q_values - q_values[choice])))
recency_trace_update_chosen = recency_trace[choice] + reward_modulated_recency_learning_rate * (1 - recency_trace[choice])
q_value_similarity_to_chosen = jax.nn.sigmoid(5.0 * (1.0 - jnp.abs(q_values - q_values[choice])))
q_value_similarity_to_chosen = jax.nn.sigmoid(5.0 * (1.0 - jnp.abs(q_values - q_values[choice])))
q_value_similarity_to_chosen = jax.nn.sigmoid(5.0 * (1.0 - jnp.abs(q_values - q_values[choice])))
q_value_similarity_to_chosen = jax.nn.sigmoid(5.0 * (1.0 - jnp.abs(q_values - q_values[choice])))
recency_decay_scaling_factor = 1.0 - 0.9 * q_value_similarity_to_chosen
q_value_similarity_to_chosen = jax.nn.sigmoid(5.0 * (1.0 - jnp.abs(q_values - q_values[choice])))
recency_decay_scaling_factor = 1.0 - 0.9 * q_value_similarity_to_chosen
recency_decay_scaling_factor = 1.0 - 0.9 * q_value_similarity_to_chosen
recency_decay_scaling_factor = 1.0 - 0.9 * q_value_similarity_to_chosen
effective_recency_decay_rate = perseveration_decay_rate * recency_decay_scaling_factor
recency_trace_update_unchosen = recency_trace * (1 - effective_recency_decay_rate)
effective_recency_decay_rate = perseveration_decay_rate * recency_decay_scaling_factor
recency_trace_update_unchosen = recency_trace * (1 - effective_recency_decay_rate)
recency_trace_update_unchosen = recency_trace * (1 - effective_recency_decay_rate)
recency_trace_update_unchosen = recency_trace * (1 - effective_recency_decay_rate)
value_uncertainty = 1.0 - jax.nn.sigmoid(jnp.abs(q_values - average_reward_estimate) * 5.0)
value_uncertainty = 1.0 - jax.nn.sigmoid(jnp.abs(q_values - average_reward_estimate) * 5.0)
value_uncertainty = 1.0 - jax.nn.sigmoid(jnp.abs(q_values - average_reward_estimate) * 5.0)
value_uncertainty = 1.0 - jax.nn.sigmoid(jnp.abs(q_values - average_reward_estimate) * 5.0)
value_uncertainty = 1.0 - jax.nn.sigmoid(jnp.abs(q_values - average_reward_estimate) * 5.0)
adaptive_perseveration_bias_strength = perseveration_bias_strength * (1.0 + perseveration_reward_sensitivity * prediction_error)
value_uncertainty = 1.0 - jax.nn.sigmoid(jnp.abs(q_values - average_reward_estimate) * 5.0)
adaptive_perseveration_bias_strength = perseveration_bias_strength * (1.0 + perseveration_reward_sensitivity * prediction_error)
adaptive_perseveration_bias_strength = perseveration_bias_strength * (1.0 + perseveration_reward_sensitivity * prediction_error)
adaptive_perseveration_bias_strength = perseveration_bias_strength * (1.0 + perseveration_reward_sensitivity * prediction_error)
adaptive_perseveration_bias_strength = perseveration_bias_strength * (1.0 + perseveration_reward_sensitivity * prediction_error)
base_perseveration_strength = perseveration_trace * adaptive_perseveration_bias_strength * (0.5 + 0.5 * recency_trace)
adaptive_perseveration_bias_strength = perseveration_bias_strength * (1.0 + perseveration_reward_sensitivity * prediction_error)
base_perseveration_strength = perseveration_trace * adaptive_perseveration_bias_strength * (0.5 + 0.5 * recency_trace)
base_perseveration_strength = perseveration_trace * adaptive_perseveration_bias_strength * (0.5 + 0.5 * recency_trace)
base_perseveration_strength = perseveration_trace * adaptive_perseveration_bias_strength * (0.5 + 0.5 * recency_trace)
base_perseveration_strength = perseveration_trace * adaptive_perseveration_bias_strength * (0.5 + 0.5 * recency_trace)
base_perseveration_strength = perseveration_trace * adaptive_perseveration_bias_strength * (0.5 + 0.5 * recency_trace)
base_perseveration_strength = perseveration_trace * adaptive_perseveration_bias_strength * (0.5 + 0.5 * recency_trace)
base_perseveration_strength = perseveration_trace * adaptive_perseveration_bias_strength * (0.5 + 0.5 * recency_trace)
perseveration_bias = jax.nn.softplus(base_perseveration_strength * (1.0 + value_uncertainty))
perseveration_bias = jax.nn.softplus(base_perseveration_strength * (1.0 + value_uncertainty))
perseveration_bias = jax.nn.softplus(base_perseveration_strength * (1.0 + value_uncertainty))
perseveration_bias = jax.nn.softplus(base_perseveration_strength * (1.0 + value_uncertainty))
relative_q_values = q_values - average_reward_estimate
relative_q_values = q_values - average_reward_estimate
variance_scaling_factor = 0.5 + 1.5 * jax.nn.sigmoid(q_value_variance)
variance_scaling_factor = 0.5 + 1.5 * jax.nn.sigmoid(q_value_variance)
variance_scaling_factor = 0.5 + 1.5 * jax.nn.sigmoid(q_value_variance)
option_specific_inverse_temperature = inverse_temperature * variance_scaling_factor * (jax.nn.relu(1 + relative_q_values) + 0.01)
variance_scaling_factor = 0.5 + 1.5 * jax.nn.sigmoid(q_value_variance)
option_specific_inverse_temperature = inverse_temperature * variance_scaling_factor * (jax.nn.relu(1 + relative_q_values) + 0.01)
option_specific_inverse_temperature = inverse_temperature * variance_scaling_factor * (jax.nn.relu(1 + relative_q_values) + 0.01)
option_specific_inverse_temperature = inverse_temperature * variance_scaling_factor * (jax.nn.relu(1 + relative_q_values) + 0.01)
option_specific_inverse_temperature = inverse_temperature * variance_scaling_factor * (jax.nn.relu(1 + relative_q_values) + 0.01)
option_specific_inverse_temperature = inverse_temperature * variance_scaling_factor * (jax.nn.relu(1 + relative_q_values) + 0.01)
option_specific_inverse_temperature = inverse_temperature * variance_scaling_factor * (jax.nn.relu(1 + relative_q_values) + 0.01)
option_specific_inverse_temperature = inverse_temperature * variance_scaling_factor * (jax.nn.relu(1 + relative_q_values) + 0.01)
choice_logits = relative_q_values * option_specific_inverse_temperature + perseveration_trace * perseveration_bias
choice_logits = relative_q_values * option_specific_inverse_temperature + perseveration_trace * perseveration_bias
choice_logits = relative_q_values * option_specific_inverse_temperature + perseveration_trace * perseveration_bias
choice_logits = relative_q_values * option_specific_inverse_temperature + perseveration_trace * perseveration_bias
choice_logits = relative_q_values * option_specific_inverse_temperature + perseveration_trace * perseveration_bias
choice_logits = relative_q_values * option_specific_inverse_temperature + perseveration_trace * perseveration_bias
−1.00 −0.75 −0.50 −0.25 0.00
