# Supplementary material for: AI-Discovered Cognitive Models Reveal Novel Insights into Human and Animal Learning
Source: bioRxiv. 2026 May 21:2026.05.18.725921. Preprint. [Version 1] doi: 10.64898/2026.05.18.725921 (PMC13228651; doi:10.64898/2026.05.18.725921)
Supplement: Supplement 2 [file ablation_performance_human_bandit_run2_high_floor_shortenonly_20260420.pdf › ablation_performance_human_bandit_run2_high_floor_shortenonly_20260420.pdf]

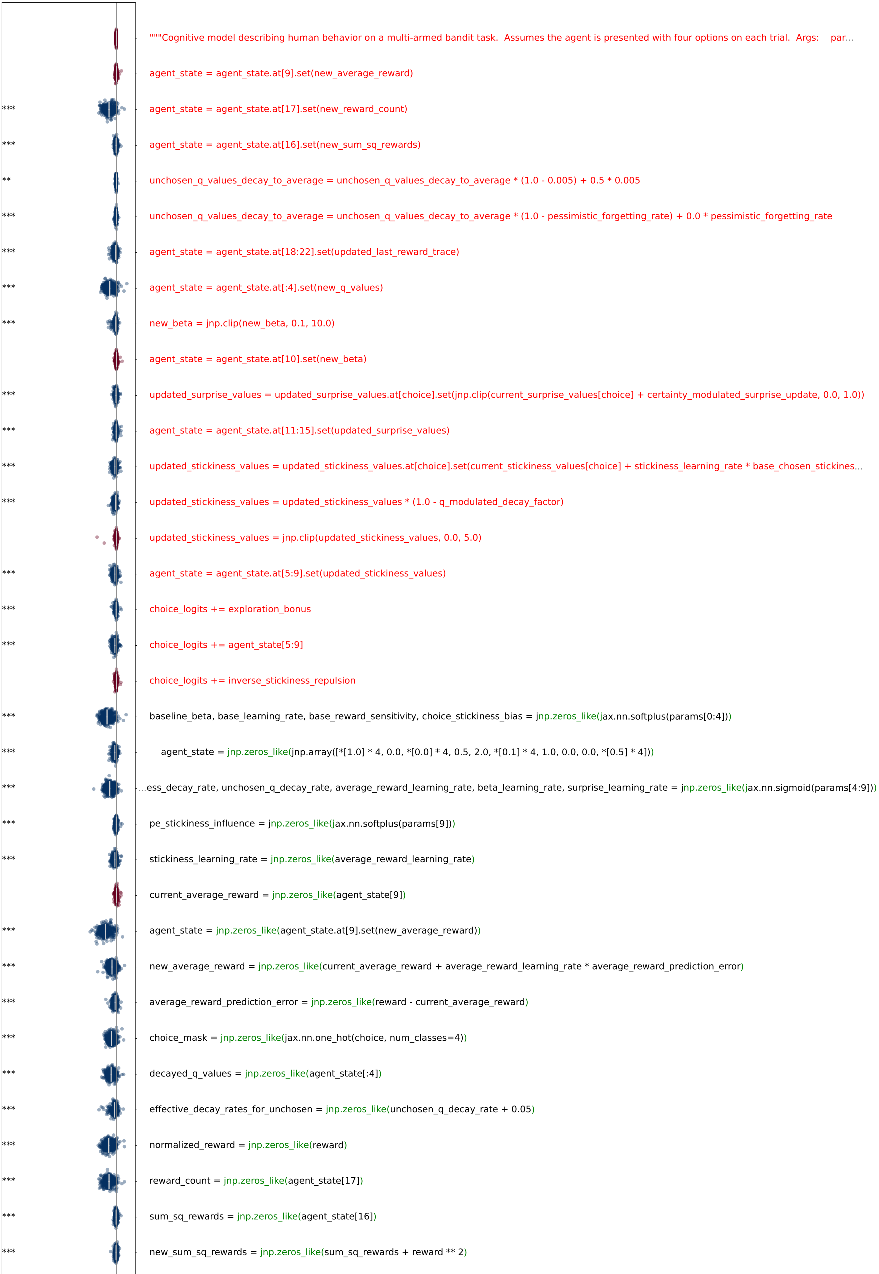

"""Cognitive model describing human behavior on a multi-armed bandit task. Assumes the agent is presented with four options on each trial. Args: par...
agent_state = agent_state.at[9].set(new_average_reward)
*** agent_state = agent_state.at[17].set(new_reward_count)
*** agent_state = agent_state.at[16].set(new_sum_sq_rewards)
** unchosen_q_values_decay_to_average = unchosen_q_values_decay_to_average * (1.0 - 0.005) + 0.5 * 0.005
*** unchosen_q_values_decay_to_average = unchosen_q_values_decay_to_average * (1.0 - pessimistic_forgetting_rate) + 0.0 * pessimistic_forgetting_rate
*** agent_state = agent_state.at[18:22].set(updated_last_reward_trace)
*** agent_state = agent_state.at[:4].set(new_q_values)
*** new_beta = jnp.clip(new_beta, 0.1, 10.0)
agent_state = agent_state.at[10].set(new_beta)
*** updated_surprise_values = updated_surprise_values.at[choice].set(jnp.clip(current_surprise_values[choice] + certainty_modulated_surprise_update, 0.0, 1.0))
*** agent_state = agent_state.at[11:15].set(updated_surprise_values)
*** updated_stickiness_values = updated_stickiness_values.at[choice].set(current_stickiness_values[choice] + stickiness_learning_rate * base_chosen_stickines...
*** updated_stickiness_values = updated_stickiness_values * (1.0 - q_modulated_decay_factor)
updated_stickiness_values = jnp.clip(updated_stickiness_values, 0.0, 5.0)
*** agent_state = agent_state.at[5:9].set(updated_stickiness_values)
*** choice_logits += exploration_bonus
*** choice_logits += agent_state[5:9]
choice_logits += inverse_stickiness_repulsion
*** baseline_beta, base_learning_rate, base_reward_sensitivity, choice_stickiness_bias = jnp.zeros_like(jax.nn.softplus(params[0:4]))
*** agent_state = jnp.zeros_like(jnp.array([*[1.0] * 4, 0.0, *[0.0] * 4, 0.5, 2.0, *[0.1] * 4, 1.0, 0.0, 0.0, *[0.5] * 4]))
*** ...ess_decay_rate, unchosen_q_decay_rate, average_reward_learning_rate, beta_learning_rate, surprise_learning_rate = jnp.zeros_like(jax.nn.sigmoid(params[4:9]))
*** pe_stickiness_influence = jnp.zeros_like(jax.nn.softplus(params[9]))
*** stickiness_learning_rate = jnp.zeros_like(average_reward_learning_rate)
current_average_reward = jnp.zeros_like(agent_state[9])
*** agent_state = jnp.zeros_like(agent_state.at[9].set(new_average_reward))
*** new_average_reward = jnp.zeros_like(current_average_reward + average_reward_learning_rate * average_reward_prediction_error)
*** average_reward_prediction_error = jnp.zeros_like(reward - current_average_reward)
*** choice_mask = jnp.zeros_like(jax.nn.one_hot(choice, num_classes=4))
*** decayed_q_values = jnp.zeros_like(agent_state[:4])
*** effective_decay_rates_for_unchosen = jnp.zeros_like(unchosen_q_decay_rate + 0.05)
*** normalized_reward = jnp.zeros_like(reward)
*** reward_count = jnp.zeros_like(agent_state[17])
*** sum_sq_rewards = jnp.zeros_like(agent_state[16])
*** new_sum_sq_rewards = jnp.zeros_like(sum_sq_rewards + reward ** 2)

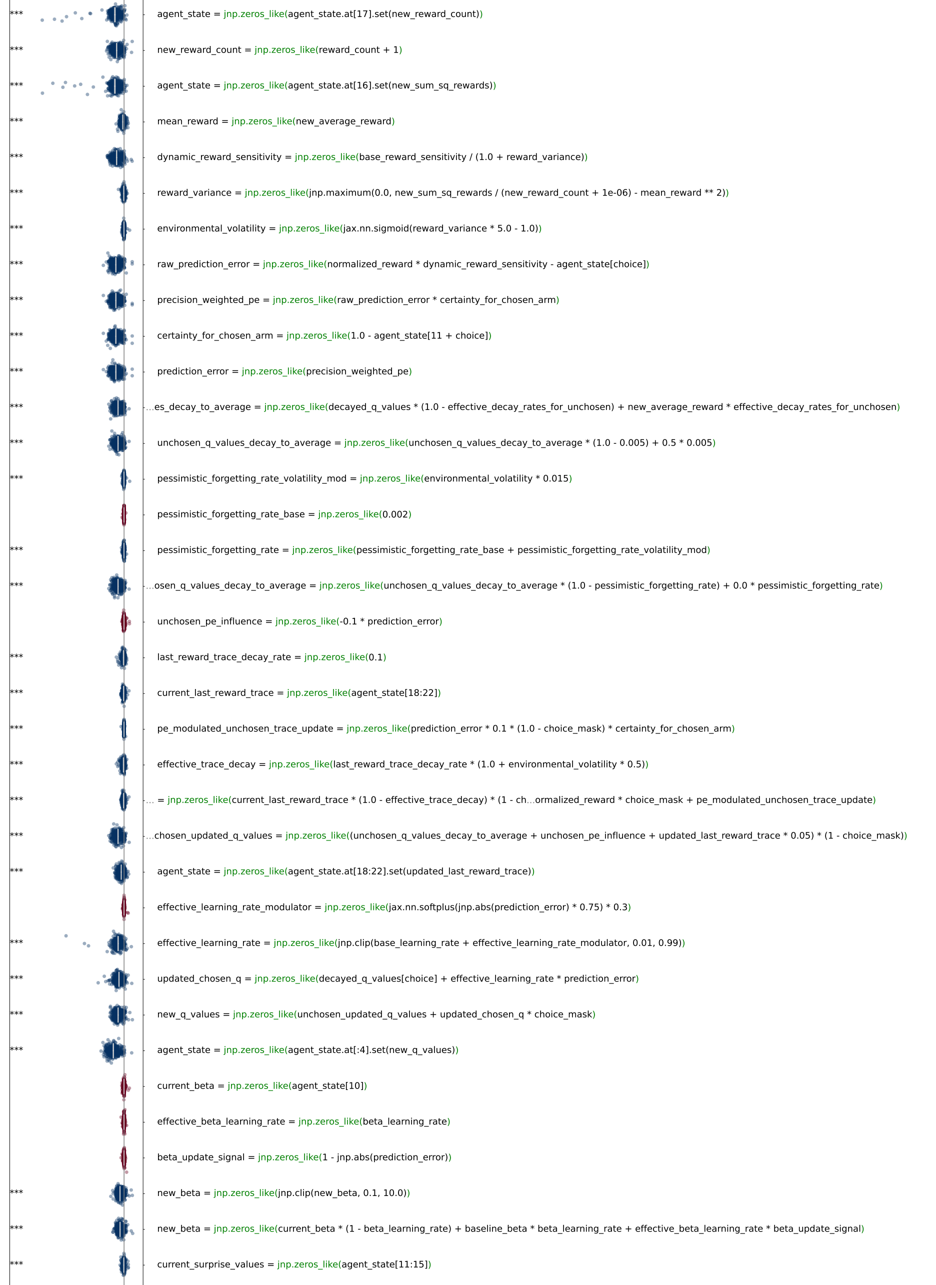

*** agent_state = jnp.zeros_like(agent_state.at[17].set(new_reward_count))
*** new_reward_count = jnp.zeros_like(reward_count + 1)
*** agent_state = jnp.zeros_like(agent_state.at[16].set(new_sum_sq_rewards))
*** mean_reward = jnp.zeros_like(new_average_reward)
*** dynamic_reward_sensitivity = jnp.zeros_like(base_reward_sensitivity / (1.0 + reward_variance))
*** reward_variance = jnp.zeros_like(jnp.maximum(0.0, new_sum_sq_rewards / (new_reward_count + 1e-06) - mean_reward ** 2))
*** environmental_volatility = jnp.zeros_like(jax.nn.sigmoid(reward_variance * 5.0 - 1.0))
*** raw_prediction_error = jnp.zeros_like(normalized_reward * dynamic_reward_sensitivity - agent_state[choice])
*** precision_weighted_pe = jnp.zeros_like(raw_prediction_error * certainty_for_chosen_arm)
*** certainty_for_chosen_arm = jnp.zeros_like(1.0 - agent_state[11 + choice])
*** prediction_error = jnp.zeros_like(precision_weighted_pe)
*** ...es_decay_to_average = jnp.zeros_like(decayed_q_values * (1.0 - effective_decay_rates_for_unchosen) + new_average_reward * effective_decay_rates_for_unchosen)
*** unchosen_q_values_decay_to_average = jnp.zeros_like(unchosen_q_values_decay_to_average * (1.0 - 0.005) + 0.5 * 0.005)
*** pessimistic_forgetting_rate_volatility_mod = jnp.zeros_like(environmental_volatility * 0.015)
pessimistic_forgetting_rate_base = jnp.zeros_like(0.002)
*** pessimistic_forgetting_rate = jnp.zeros_like(pessimistic_forgetting_rate_base + pessimistic_forgetting_rate_volatility_mod)
*** ...osen_q_values_decay_to_average = jnp.zeros_like(unchosen_q_values_decay_to_average * (1.0 - pessimistic_forgetting_rate) + 0.0 * pessimistic_forgetting_rate)
unchosen_pe_influence = jnp.zeros_like(-0.1 * prediction_error)
*** last_reward_trace_decay_rate = jnp.zeros_like(0.1)
*** current_last_reward_trace = jnp.zeros_like(agent_state[18:22])
*** pe_modulated_unchosen_trace_update = jnp.zeros_like(prediction_error * 0.1 * (1.0 - choice_mask) * certainty_for_chosen_arm)
*** effective_trace_decay = jnp.zeros_like(last_reward_trace_decay_rate * (1.0 + environmental_volatility * 0.5))
*** ... = jnp.zeros_like(current_last_reward_trace * (1.0 - effective_trace_decay) * (1 - ch...ormalized_reward * choice_mask + pe_modulated_unchosen_trace_update)
*** ...chosen_updated_q_values = jnp.zeros_like((unchosen_q_values_decay_to_average + unchosen_pe_influence + updated_last_reward_trace * 0.05) * (1 - choice_mask))
*** agent_state = jnp.zeros_like(agent_state.at[18:22].set(updated_last_reward_trace))
effective_learning_rate_modulator = jnp.zeros_like(jax.nn.softplus(jnp.abs(prediction_error) * 0.75) * 0.3)
*** effective_learning_rate = jnp.zeros_like(jnp.clip(base_learning_rate + effective_learning_rate_modulator, 0.01, 0.99))
*** updated_chosen_q = jnp.zeros_like(decayed_q_values[choice] + effective_learning_rate * prediction_error)
*** new_q_values = jnp.zeros_like(unchosen_updated_q_values + updated_chosen_q * choice_mask)
*** agent_state = jnp.zeros_like(agent_state.at[:4].set(new_q_values))
current_beta = jnp.zeros_like(agent_state[10])
effective_beta_learning_rate = jnp.zeros_like(beta_learning_rate)
beta_update_signal = jnp.zeros_like(1 - jnp.abs(prediction_error))
*** new_beta = jnp.zeros_like(jnp.clip(new_beta, 0.1, 10.0))
*** new_beta = jnp.zeros_like(current_beta * (1 - beta_learning_rate) + baseline_beta * beta_learning_rate + effective_beta_learning_rate * beta_update_signal)
*** current_surprise_values = jnp.zeros_like(agent_state[11:15])

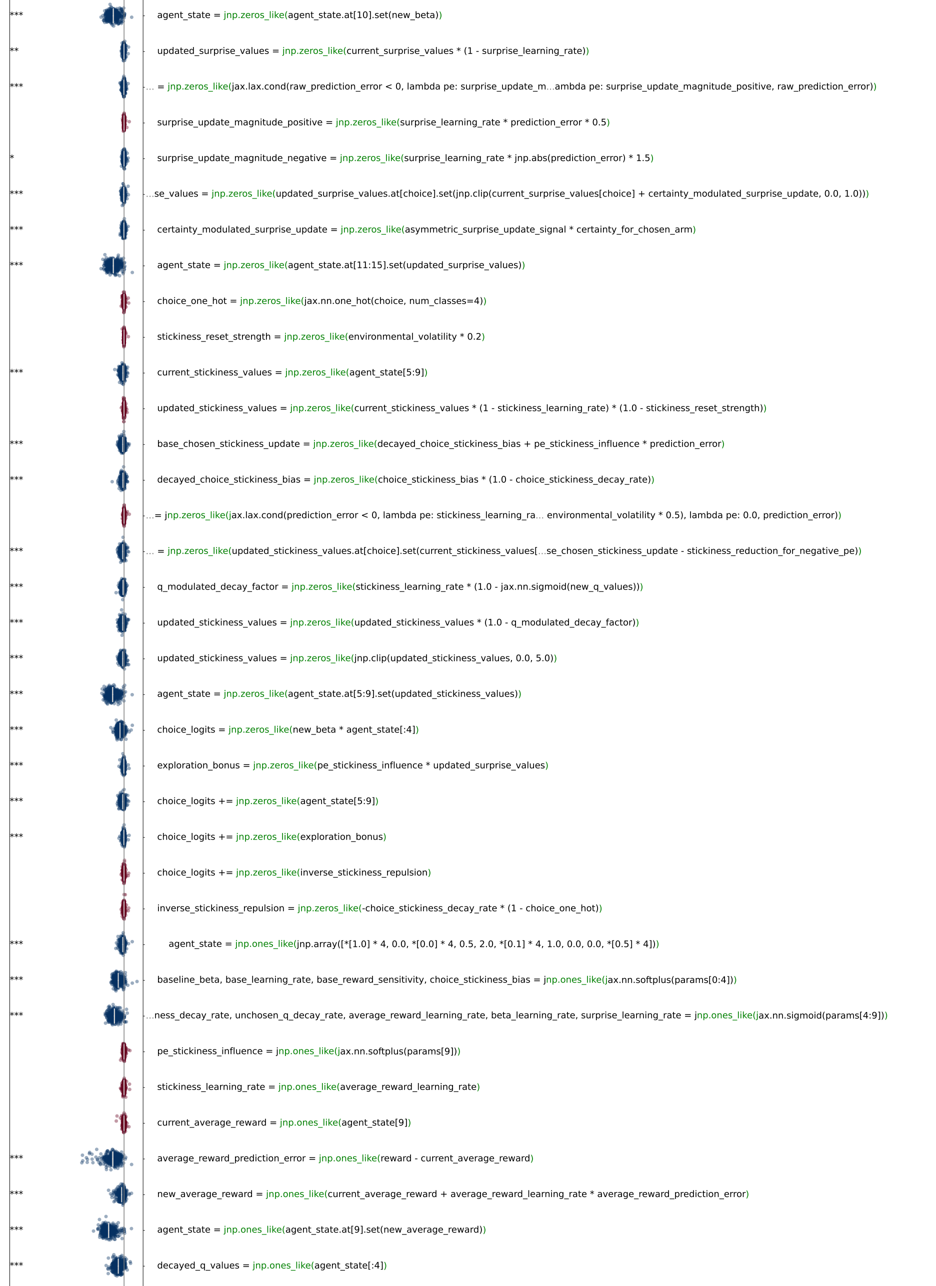

*** agent_state = jnp.zeros_like(agent_state.at[10].set(new_beta))
** updated_surprise_values = jnp.zeros_like(current_surprise_values * (1 - surprise_learning_rate))
*** … = jnp.zeros_like(jax.lax.cond(raw_prediction_error < 0, lambda pe: surprise_update_m…ambda pe: surprise_update_magnitude_positive, raw_prediction_error))
surprise_update_magnitude_positive = jnp.zeros_like(surprise_learning_rate * prediction_error * 0.5)
* surprise_update_magnitude_negative = jnp.zeros_like(surprise_learning_rate * jnp.abs(prediction_error) * 1.5)
*** …se_values = jnp.zeros_like(updated_surprise_values.at[choice].set(jnp.clip(current_surprise_values[choice] + certainty_modulated_surprise_update, 0.0, 1.0)))
*** certainty_modulated_surprise_update = jnp.zeros_like(asymmetric_surprise_update_signal * certainty_for_chosen_arm)
*** agent_state = jnp.zeros_like(agent_state.at[11:15].set(updated_surprise_values))
choice_one_hot = jnp.zeros_like(jax.nn.one_hot(choice, num_classes=4))
stickiness_reset_strength = jnp.zeros_like(environmental_volatility * 0.2)
*** current_stickiness_values = jnp.zeros_like(agent_state[5:9])
updated_stickiness_values = jnp.zeros_like(current_stickiness_values * (1 - stickiness_learning_rate) * (1.0 - stickiness_reset_strength))
*** base_chosen_stickiness_update = jnp.zeros_like(decayed_choice_stickiness_bias + pe_stickiness_influence * prediction_error)
*** decayed_choice_stickiness_bias = jnp.zeros_like(choice_stickiness_bias * (1.0 - choice_stickiness_decay_rate))
…= jnp.zeros_like(jax.lax.cond(prediction_error < 0, lambda pe: stickiness_learning_ra… environmental_volatility * 0.5), lambda pe: 0.0, prediction_error))
*** … = jnp.zeros_like(updated_stickiness_values.at[choice].set(current_stickiness_values[…se_chosen_stickiness_update - stickiness_reduction_for_negative_pe))
*** q_modulated_decay_factor = jnp.zeros_like(stickiness_learning_rate * (1.0 - jax.nn.sigmoid(new_q_values)))
*** updated_stickiness_values = jnp.zeros_like(updated_stickiness_values * (1.0 - q_modulated_decay_factor))
*** updated_stickiness_values = jnp.zeros_like(jnp.clip(updated_stickiness_values, 0.0, 5.0))
*** agent_state = jnp.zeros_like(agent_state.at[5:9].set(updated_stickiness_values))
*** choice_logits = jnp.zeros_like(new_beta * agent_state[:4])
*** exploration_bonus = jnp.zeros_like(pe_stickiness_influence * updated_surprise_values)
*** choice_logits += jnp.zeros_like(agent_state[5:9])
*** choice_logits += jnp.zeros_like(exploration_bonus)
choice_logits += jnp.zeros_like(inverse_stickiness_repulsion)
inverse_stickiness_repulsion = jnp.zeros_like(-choice_stickiness_decay_rate * (1 - choice_one_hot))
*** agent_state = jnp.ones_like(jnp.array([*[1.0] * 4, 0.0, *[0.0] * 4, 0.5, 2.0, *[0.1] * 4, 1.0, 0.0, 0.0, *[0.5] * 4]))
*** baseline_beta, base_learning_rate, base_reward_sensitivity, choice_stickiness_bias = jnp.ones_like(jax.nn.softplus(params[0:4]))
*** …ness_decay_rate, unchosen_q_decay_rate, average_reward_learning_rate, beta_learning_rate, surprise_learning_rate = jnp.ones_like(jax.nn.sigmoid(params[4:9]))
pe_stickiness_influence = jnp.ones_like(jax.nn.softplus(params[9]))
stickiness_learning_rate = jnp.ones_like(average_reward_learning_rate)
current_average_reward = jnp.ones_like(agent_state[9])
*** average_reward_prediction_error = jnp.ones_like(reward - current_average_reward)
*** new_average_reward = jnp.ones_like(current_average_reward + average_reward_learning_rate * average_reward_prediction_error)
*** agent_state = jnp.ones_like(agent_state.at[9].set(new_average_reward))
*** decayed_q_values = jnp.ones_like(agent_state[:4])

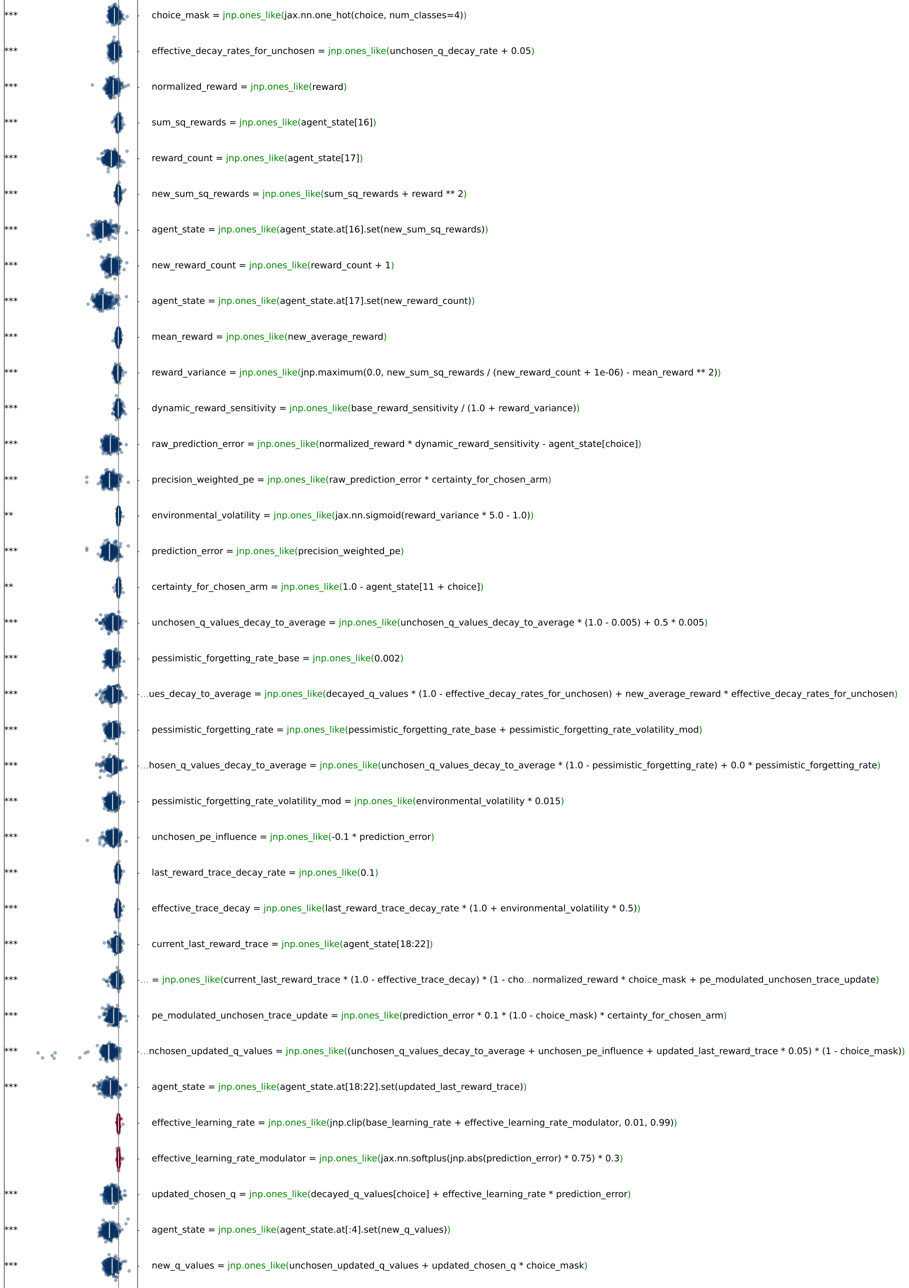
*** choice_mask = jnp.ones_like(jax.nn.one_hot(choice, num_classes=4))
*** effective_decay_rates_for_unchosen = jnp.ones_like(unchosen_q_decay_rate + 0.05)
*** normalized_reward = jnp.ones_like(reward)
*** sum_sq_rewards = jnp.ones_like(agent_state[16])
*** reward_count = jnp.ones_like(agent_state[17])
*** new_sum_sq_rewards = jnp.ones_like(sum_sq_rewards + reward ** 2)
*** agent_state = jnp.ones_like(agent_state.at[16].set(new_sum_sq_rewards))
*** new_reward_count = jnp.ones_like(reward_count + 1)
*** agent_state = jnp.ones_like(agent_state.at[17].set(new_reward_count))
*** mean_reward = jnp.ones_like(new_average_reward)
*** reward_variance = jnp.ones_like(jnp.maximum(0.0, new_sum_sq_rewards / (new_reward_count + 1e-06) - mean_reward ** 2))
*** dynamic_reward_sensitivity = jnp.ones_like(base_reward_sensitivity / (1.0 + reward_variance))
*** raw_prediction_error = jnp.ones_like(normalized_reward * dynamic_reward_sensitivity - agent_state[choice])
*** precision_weighted_pe = jnp.ones_like(raw_prediction_error * certainty_for_chosen_arm)
** environmental_volatility = jnp.ones_like(jax.nn.sigmoid(reward_variance * 5.0 - 1.0))
*** prediction_error = jnp.ones_like(precision_weighted_pe)
** certainty_for_chosen_arm = jnp.ones_like(1.0 - agent_state[11 + choice])
*** unchosen_q_values_decay_to_average = jnp.ones_like(unchosen_q_values_decay_to_average * (1.0 - 0.005) + 0.5 * 0.005)
*** pessimistic_forgetting_rate_base = jnp.ones_like(0.002)
*** …ues_decay_to_average = jnp.ones_like(decayed_q_values * (1.0 - effective_decay_rates_for_unchosen) + new_average_reward * effective_decay_rates_for_unchosen)
*** pessimistic_forgetting_rate = jnp.ones_like(pessimistic_forgetting_rate_base + pessimistic_forgetting_rate_volatility_mod)
*** …hosen_q_values_decay_to_average = jnp.ones_like(unchosen_q_values_decay_to_average * (1.0 - pessimistic_forgetting_rate) + 0.0 * pessimistic_forgetting_rate)
*** pessimistic_forgetting_rate_volatility_mod = jnp.ones_like(environmental_volatility * 0.015)
*** unchosen_pe_influence = jnp.ones_like(-0.1 * prediction_error)
*** last_reward_trace_decay_rate = jnp.ones_like(0.1)
*** effective_trace_decay = jnp.ones_like(last_reward_trace_decay_rate * (1.0 + environmental_volatility * 0.5))
*** current_last_reward_trace = jnp.ones_like(agent_state[18:22])
*** … = jnp.ones_like(current_last_reward_trace * (1.0 - effective_trace_decay) * (1 - cho…normalized_reward * choice_mask + pe_modulated_unchosen_trace_update)
*** pe_modulated_unchosen_trace_update = jnp.ones_like(prediction_error * 0.1 * (1.0 - choice_mask) * certainty_for_chosen_arm)
*** …nchosen_updated_q_values = jnp.ones_like((unchosen_q_values_decay_to_average + unchosen_pe_influence + updated_last_reward_trace * 0.05) * (1 - choice_mask))
*** agent_state = jnp.ones_like(agent_state.at[18:22].set(updated_last_reward_trace))
effective_learning_rate = jnp.ones_like(jnp.clip(base_learning_rate + effective_learning_rate_modulator, 0.01, 0.99))
effective_learning_rate_modulator = jnp.ones_like(jax.nn.softplus(jnp.abs(prediction_error) * 0.75) * 0.3)
*** updated_chosen_q = jnp.ones_like(decayed_q_values[choice] + effective_learning_rate * prediction_error)
*** agent_state = jnp.ones_like(agent_state.at[:4].set(new_q_values))
*** new_q_values = jnp.ones_like(unchosen_updated_q_values + updated_chosen_q * choice_mask)

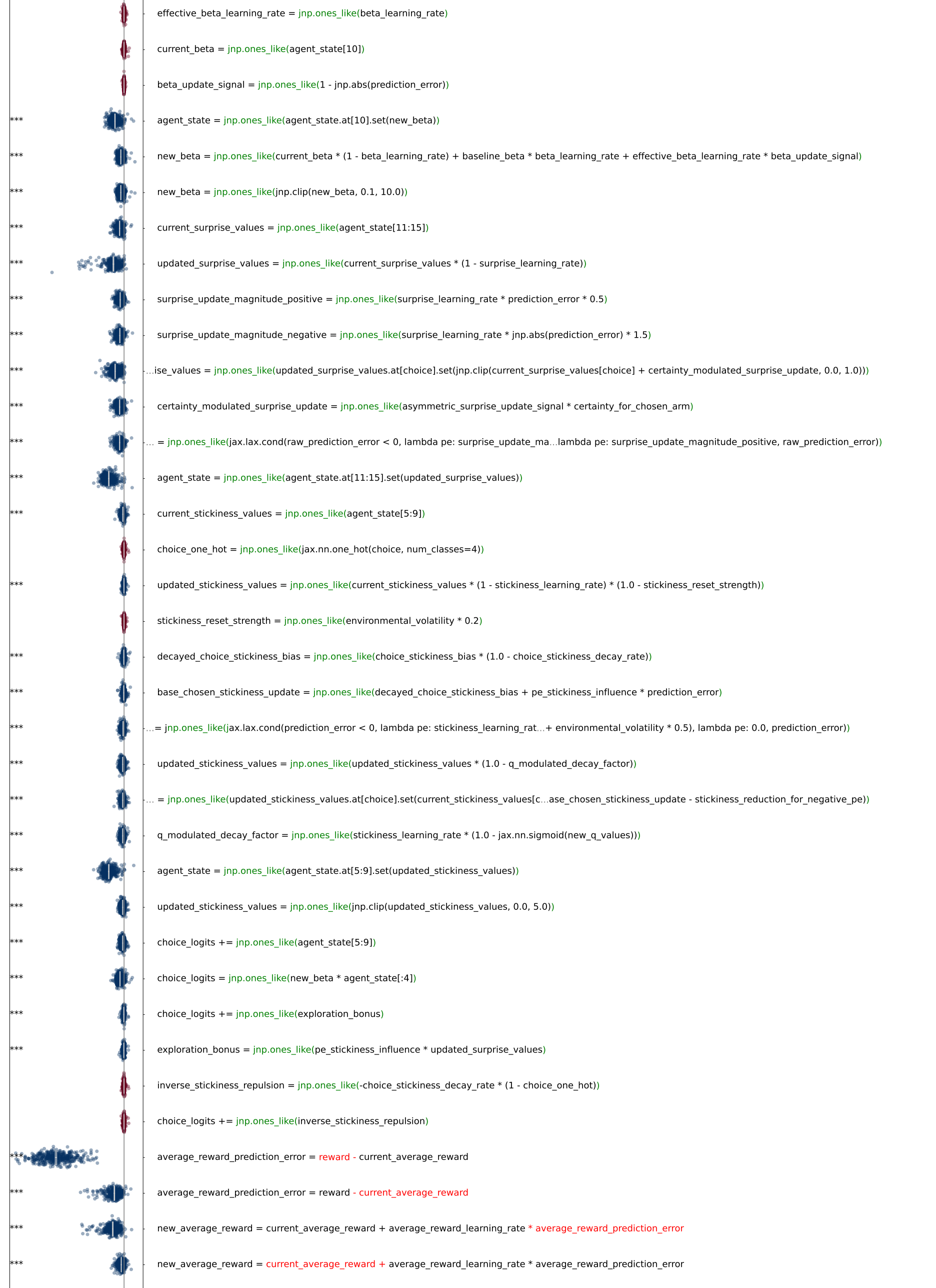
effective_beta_learning_rate = jnp.ones_like(beta_learning_rate)
current_beta = jnp.ones_like(agent_state[10])
beta_update_signal = jnp.ones_like(1 - jnp.abs(prediction_error))
*** agent_state = jnp.ones_like(agent_state.at[10].set(new_beta))
*** new_beta = jnp.ones_like(current_beta * (1 - beta_learning_rate) + baseline_beta * beta_learning_rate + effective_beta_learning_rate * beta_update_signal)
*** new_beta = jnp.ones_like(jnp.clip(new_beta, 0.1, 10.0))
*** current_surprise_values = jnp.ones_like(agent_state[11:15])
*** updated_surprise_values = jnp.ones_like(current_surprise_values * (1 - surprise_learning_rate))
*** surprise_update_magnitude_positive = jnp.ones_like(surprise_learning_rate * prediction_error * 0.5)
*** surprise_update_magnitude_negative = jnp.ones_like(surprise_learning_rate * jnp.abs(prediction_error) * 1.5)
*** …ise_values = jnp.ones_like(updated_surprise_values.at[choice].set(jnp.clip(current_surprise_values[choice] + certainty_modulated_surprise_update, 0.0, 1.0)))
*** certainty_modulated_surprise_update = jnp.ones_like(asymmetric_surprise_update_signal * certainty_for_chosen_arm)
*** … = jnp.ones_like(jax.lax.cond(raw_prediction_error < 0, lambda pe: surprise_update_ma…lambda pe: surprise_update_magnitude_positive, raw_prediction_error))
*** agent_state = jnp.ones_like(agent_state.at[11:15].set(updated_surprise_values))
*** current_stickiness_values = jnp.ones_like(agent_state[5:9])
choice_one_hot = jnp.ones_like(jax.nn.one_hot(choice, num_classes=4))
*** updated_stickiness_values = jnp.ones_like(current_stickiness_values * (1 - stickiness_learning_rate) * (1.0 - stickiness_reset_strength))
stickiness_reset_strength = jnp.ones_like(environmental_volatility * 0.2)
*** decayed_choice_stickiness_bias = jnp.ones_like(choice_stickiness_bias * (1.0 - choice_stickiness_decay_rate))
*** base_chosen_stickiness_update = jnp.ones_like(decayed_choice_stickiness_bias + pe_stickiness_influence * prediction_error)
*** …= jnp.ones_like(jax.lax.cond(prediction_error < 0, lambda pe: stickiness_learning_rat…+ environmental_volatility * 0.5), lambda pe: 0.0, prediction_error))
*** updated_stickiness_values = jnp.ones_like(updated_stickiness_values * (1.0 - q_modulated_decay_factor))
*** … = jnp.ones_like(updated_stickiness_values.at[choice].set(current_stickiness_values[c…ase_chosen_stickiness_update - stickiness_reduction_for_negative_pe))
*** q_modulated_decay_factor = jnp.ones_like(stickiness_learning_rate * (1.0 - jax.nn.sigmoid(new_q_values)))
*** agent_state = jnp.ones_like(agent_state.at[5:9].set(updated_stickiness_values))
*** updated_stickiness_values = jnp.ones_like(jnp.clip(updated_stickiness_values, 0.0, 5.0))
*** choice_logits += jnp.ones_like(agent_state[5:9])
*** choice_logits = jnp.ones_like(new_beta * agent_state[:4])
*** choice_logits += jnp.ones_like(exploration_bonus)
*** exploration_bonus = jnp.ones_like(pe_stickiness_influence * updated_surprise_values)
inverse_stickiness_repulsion = jnp.ones_like(-choice_stickiness_decay_rate * (1 - choice_one_hot))
choice_logits += jnp.ones_like(inverse_stickiness_repulsion)
*** average_reward_prediction_error = reward - current_average_reward
*** average_reward_prediction_error = reward - current_average_reward
*** new_average_reward = current_average_reward + average_reward_learning_rate * average_reward_prediction_error
*** new_average_reward = current_average_reward + average_reward_learning_rate * average_reward_prediction_error

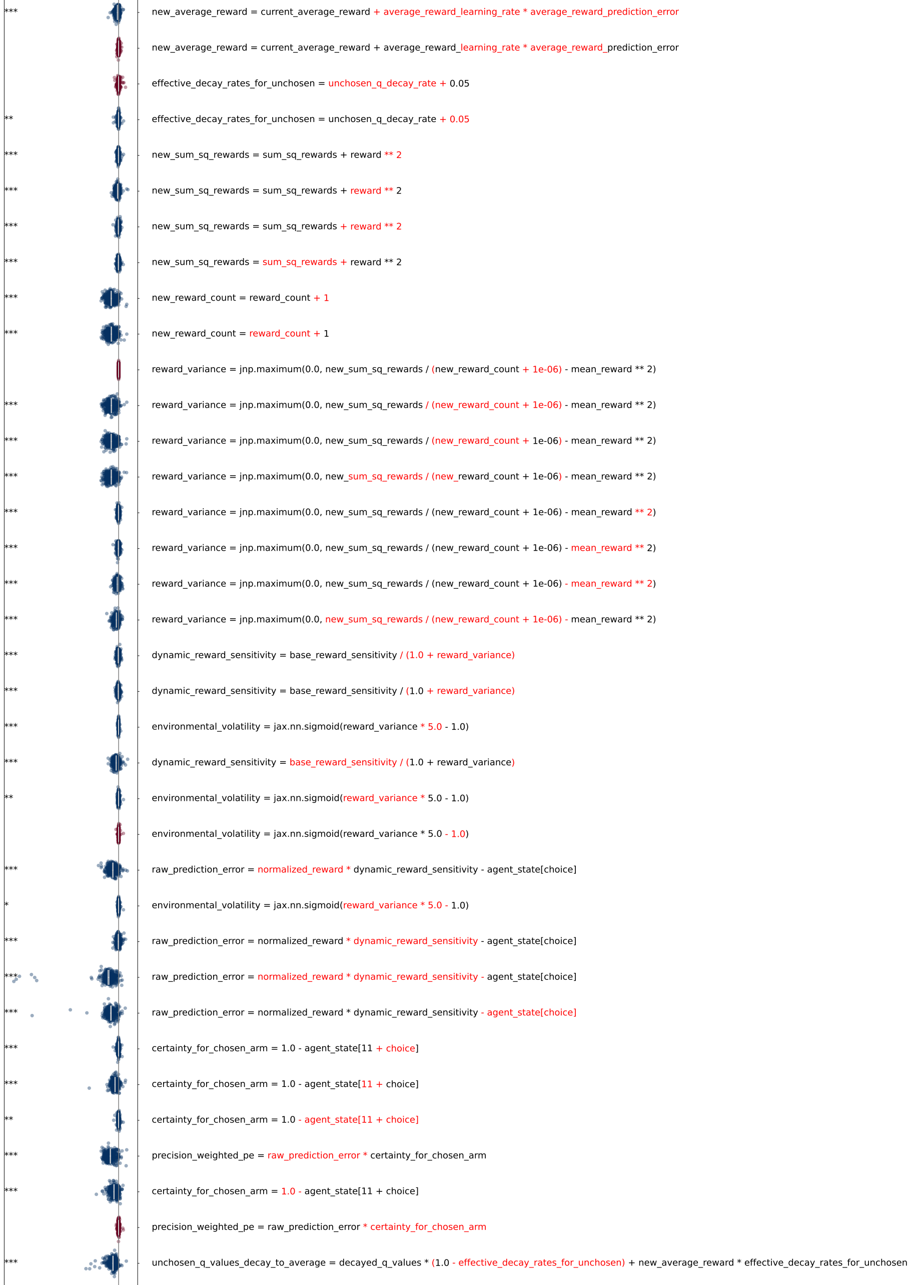

*** new_average_reward = current_average_reward + average_reward_learning_rate * average_reward_prediction_error
new_average_reward = current_average_reward + average_reward_learning_rate * average_reward_prediction_error
effective_decay_rates_for_unchosen = unchosen_q_decay_rate + 0.05
** effective_decay_rates_for_unchosen = unchosen_q_decay_rate + 0.05
*** new_sum_sq_rewards = sum_sq_rewards + reward ** 2
*** new_sum_sq_rewards = sum_sq_rewards + reward ** 2
*** new_sum_sq_rewards = sum_sq_rewards + reward ** 2
*** new_sum_sq_rewards = sum_sq_rewards + reward ** 2
*** new_reward_count = reward_count + 1
*** new_reward_count = reward_count + 1
reward_variance = jnp.maximum(0.0, new_sum_sq_rewards / (new_reward_count + 1e-06) - mean_reward ** 2)
*** reward_variance = jnp.maximum(0.0, new_sum_sq_rewards / (new_reward_count + 1e-06) - mean_reward ** 2)
*** reward_variance = jnp.maximum(0.0, new_sum_sq_rewards / (new_reward_count + 1e-06) - mean_reward ** 2)
*** reward_variance = jnp.maximum(0.0, new_sum_sq_rewards / (new_reward_count + 1e-06) - mean_reward ** 2)
*** reward_variance = jnp.maximum(0.0, new_sum_sq_rewards / (new_reward_count + 1e-06) - mean_reward ** 2)
*** reward_variance = jnp.maximum(0.0, new_sum_sq_rewards / (new_reward_count + 1e-06) - mean_reward ** 2)
*** reward_variance = jnp.maximum(0.0, new_sum_sq_rewards / (new_reward_count + 1e-06) - mean_reward ** 2)
*** reward_variance = jnp.maximum(0.0, new_sum_sq_rewards / (new_reward_count + 1e-06) - mean_reward ** 2)
*** dynamic_reward_sensitivity = base_reward_sensitivity / (1.0 + reward_variance)
*** dynamic_reward_sensitivity = base_reward_sensitivity / (1.0 + reward_variance)
*** environmental_volatility = jax.nn.sigmoid(reward_variance * 5.0 - 1.0)
*** dynamic_reward_sensitivity = base_reward_sensitivity / (1.0 + reward_variance)
** environmental_volatility = jax.nn.sigmoid(reward_variance * 5.0 - 1.0)
environmental_volatility = jax.nn.sigmoid(reward_variance * 5.0 - 1.0)
*** raw_prediction_error = normalized_reward * dynamic_reward_sensitivity - agent_state[choice]
* environmental_volatility = jax.nn.sigmoid(reward_variance * 5.0 - 1.0)
*** raw_prediction_error = normalized_reward * dynamic_reward_sensitivity - agent_state[choice]
*** raw_prediction_error = normalized_reward * dynamic_reward_sensitivity - agent_state[choice]
*** raw_prediction_error = normalized_reward * dynamic_reward_sensitivity - agent_state[choice]
*** certainty_for_chosen_arm = 1.0 - agent_state[11 + choice]
*** certainty_for_chosen_arm = 1.0 - agent_state[11 + choice]
** certainty_for_chosen_arm = 1.0 - agent_state[11 + choice]
*** precision_weighted_pe = raw_prediction_error * certainty_for_chosen_arm
*** certainty_for_chosen_arm = 1.0 - agent_state[11 + choice]
precision_weighted_pe = raw_prediction_error * certainty_for_chosen_arm
*** unchosen_q_values_decay_to_average = decayed_q_values * (1.0 - effective_decay_rates_for_unchosen) + new_average_reward * effective_decay_rates_for_unchosen

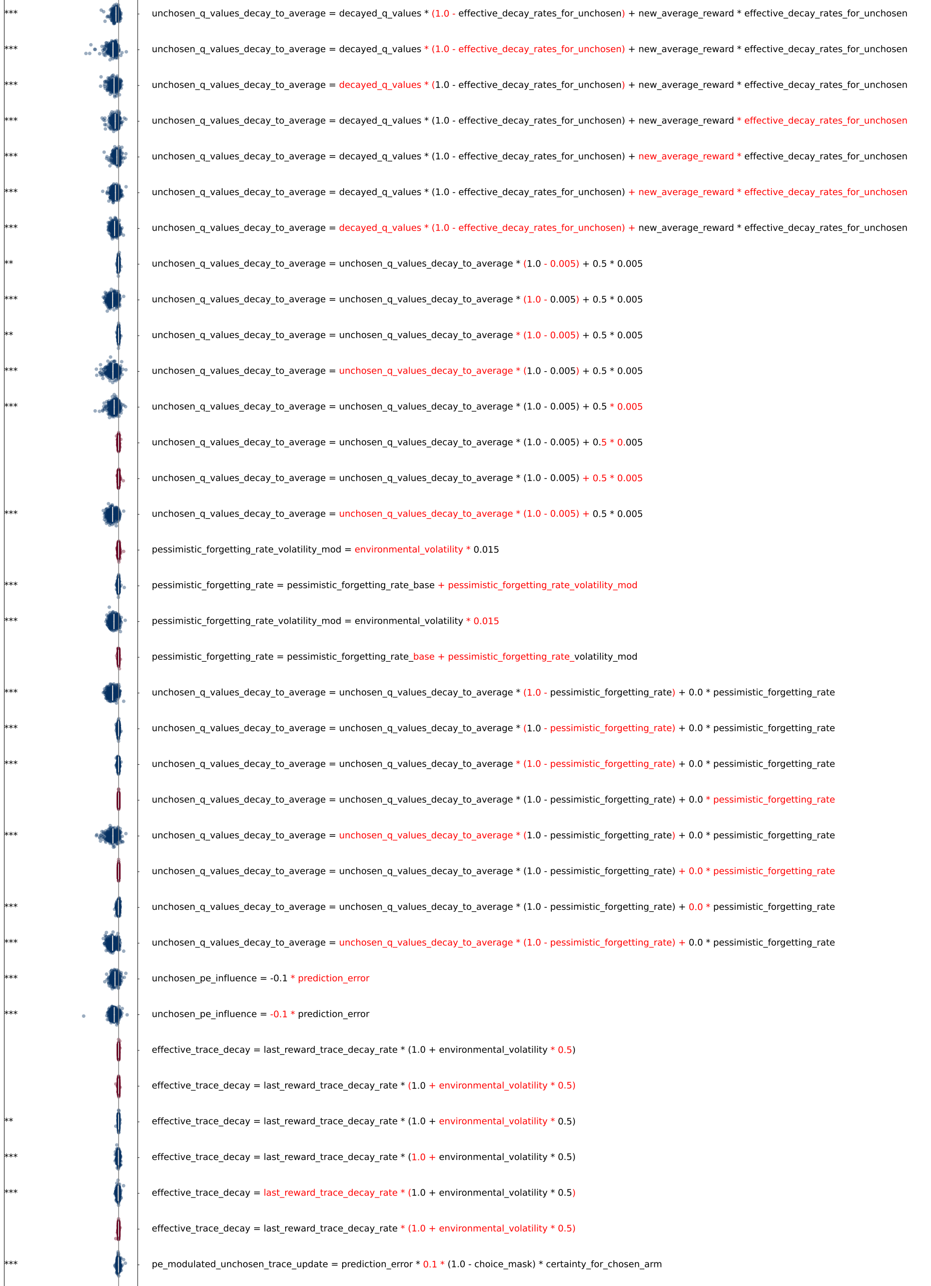

*** unchosen_q_values_decay_to_average = decayed_q_values * (1.0 - effective_decay_rates_for_unchosen) + new_average_reward * effective_decay_rates_for_unchosen
*** unchosen_q_values_decay_to_average = decayed_q_values * (1.0 - effective_decay_rates_for_unchosen) + new_average_reward * effective_decay_rates_for_unchosen
*** unchosen_q_values_decay_to_average = decayed_q_values * (1.0 - effective_decay_rates_for_unchosen) + new_average_reward * effective_decay_rates_for_unchosen
*** unchosen_q_values_decay_to_average = decayed_q_values * (1.0 - effective_decay_rates_for_unchosen) + new_average_reward * effective_decay_rates_for_unchosen
*** unchosen_q_values_decay_to_average = decayed_q_values * (1.0 - effective_decay_rates_for_unchosen) + new_average_reward * effective_decay_rates_for_unchosen
*** unchosen_q_values_decay_to_average = decayed_q_values * (1.0 - effective_decay_rates_for_unchosen) + new_average_reward * effective_decay_rates_for_unchosen
*** unchosen_q_values_decay_to_average = decayed_q_values * (1.0 - effective_decay_rates_for_unchosen) + new_average_reward * effective_decay_rates_for_unchosen
** unchosen_q_values_decay_to_average = unchosen_q_values_decay_to_average * (1.0 - 0.005) + 0.5 * 0.005
*** unchosen_q_values_decay_to_average = unchosen_q_values_decay_to_average * (1.0 - 0.005) + 0.5 * 0.005
** unchosen_q_values_decay_to_average = unchosen_q_values_decay_to_average * (1.0 - 0.005) + 0.5 * 0.005
*** unchosen_q_values_decay_to_average = unchosen_q_values_decay_to_average * (1.0 - 0.005) + 0.5 * 0.005
*** unchosen_q_values_decay_to_average = unchosen_q_values_decay_to_average * (1.0 - 0.005) + 0.5 * 0.005
unchosen_q_values_decay_to_average = unchosen_q_values_decay_to_average * (1.0 - 0.005) + 0.5 * 0.005
unchosen_q_values_decay_to_average = unchosen_q_values_decay_to_average * (1.0 - 0.005) + 0.5 * 0.005
*** unchosen_q_values_decay_to_average = unchosen_q_values_decay_to_average * (1.0 - 0.005) + 0.5 * 0.005
pessimistic_forgetting_rate_volatility_mod = environmental_volatility * 0.015
*** pessimistic_forgetting_rate = pessimistic_forgetting_rate_base + pessimistic_forgetting_rate_volatility_mod
*** pessimistic_forgetting_rate_volatility_mod = environmental_volatility * 0.015
pessimistic_forgetting_rate = pessimistic_forgetting_rate_base + pessimistic_forgetting_rate_volatility_mod
*** unchosen_q_values_decay_to_average = unchosen_q_values_decay_to_average * (1.0 - pessimistic_forgetting_rate) + 0.0 * pessimistic_forgetting_rate
*** unchosen_q_values_decay_to_average = unchosen_q_values_decay_to_average * (1.0 - pessimistic_forgetting_rate) + 0.0 * pessimistic_forgetting_rate
*** unchosen_q_values_decay_to_average = unchosen_q_values_decay_to_average * (1.0 - pessimistic_forgetting_rate) + 0.0 * pessimistic_forgetting_rate
unchosen_q_values_decay_to_average = unchosen_q_values_decay_to_average * (1.0 - pessimistic_forgetting_rate) + 0.0 * pessimistic_forgetting_rate
*** unchosen_q_values_decay_to_average = unchosen_q_values_decay_to_average * (1.0 - pessimistic_forgetting_rate) + 0.0 * pessimistic_forgetting_rate
unchosen_q_values_decay_to_average = unchosen_q_values_decay_to_average * (1.0 - pessimistic_forgetting_rate) + 0.0 * pessimistic_forgetting_rate
*** unchosen_q_values_decay_to_average = unchosen_q_values_decay_to_average * (1.0 - pessimistic_forgetting_rate) + 0.0 * pessimistic_forgetting_rate
*** unchosen_q_values_decay_to_average = unchosen_q_values_decay_to_average * (1.0 - pessimistic_forgetting_rate) + 0.0 * pessimistic_forgetting_rate
*** unchosen_pe_influence = -0.1 * prediction_error
*** unchosen_pe_influence = -0.1 * prediction_error
effective_trace_decay = last_reward_trace_decay_rate * (1.0 + environmental_volatility * 0.5)
effective_trace_decay = last_reward_trace_decay_rate * (1.0 + environmental_volatility * 0.5)
** effective_trace_decay = last_reward_trace_decay_rate * (1.0 + environmental_volatility * 0.5)
*** effective_trace_decay = last_reward_trace_decay_rate * (1.0 + environmental_volatility * 0.5)
*** effective_trace_decay = last_reward_trace_decay_rate * (1.0 + environmental_volatility * 0.5)
effective_trace_decay = last_reward_trace_decay_rate * (1.0 + environmental_volatility * 0.5)
*** pe_modulated_unchosen_trace_update = prediction_error * 0.1 * (1.0 - choice_mask) * certainty_for_chosen_arm

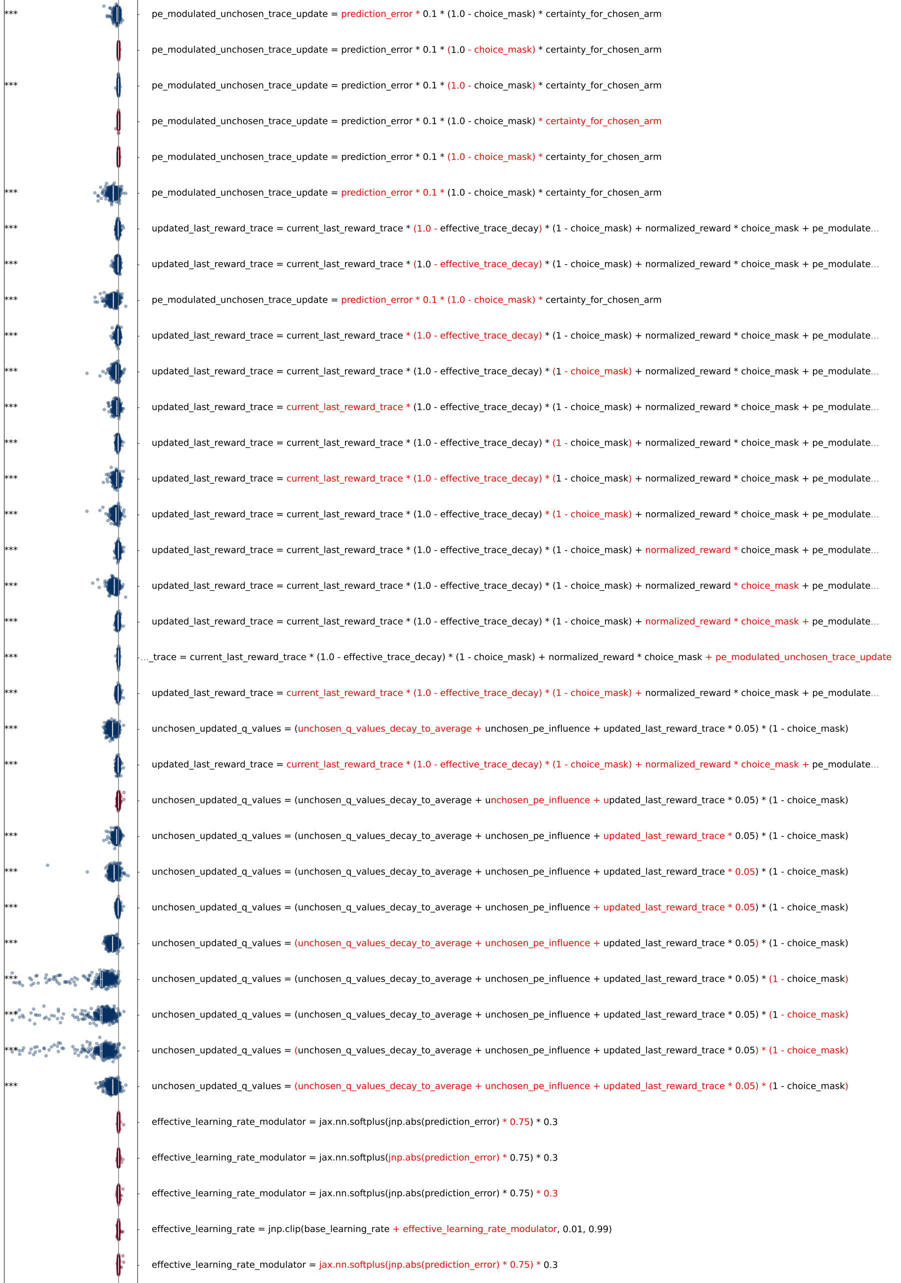

*** pe_modulated_unchosen_trace_update = prediction_error * 0.1 * (1.0 - choice_mask) * certainty_for_chosen_arm
pe_modulated_unchosen_trace_update = prediction_error * 0.1 * (1.0 - choice_mask) * certainty_for_chosen_arm
*** pe_modulated_unchosen_trace_update = prediction_error * 0.1 * (1.0 - choice_mask) * certainty_for_chosen_arm
pe_modulated_unchosen_trace_update = prediction_error * 0.1 * (1.0 - choice_mask) * certainty_for_chosen_arm
pe_modulated_unchosen_trace_update = prediction_error * 0.1 * (1.0 - choice_mask) * certainty_for_chosen_arm
*** pe_modulated_unchosen_trace_update = prediction_error * 0.1 * (1.0 - choice_mask) * certainty_for_chosen_arm
*** updated_last_reward_trace = current_last_reward_trace * (1.0 - effective_trace_decay) * (1 - choice_mask) + normalized_reward * choice_mask + pe_modulate...
*** updated_last_reward_trace = current_last_reward_trace * (1.0 - effective_trace_decay) * (1 - choice_mask) + normalized_reward * choice_mask + pe_modulate...
*** pe_modulated_unchosen_trace_update = prediction_error * 0.1 * (1.0 - choice_mask) * certainty_for_chosen_arm
*** updated_last_reward_trace = current_last_reward_trace * (1.0 - effective_trace_decay) * (1 - choice_mask) + normalized_reward * choice_mask + pe_modulate...
*** updated_last_reward_trace = current_last_reward_trace * (1.0 - effective_trace_decay) * (1 - choice_mask) + normalized_reward * choice_mask + pe_modulate...
*** updated_last_reward_trace = current_last_reward_trace * (1.0 - effective_trace_decay) * (1 - choice_mask) + normalized_reward * choice_mask + pe_modulate...
*** updated_last_reward_trace = current_last_reward_trace * (1.0 - effective_trace_decay) * (1 - choice_mask) + normalized_reward * choice_mask + pe_modulate...
*** updated_last_reward_trace = current_last_reward_trace * (1.0 - effective_trace_decay) * (1 - choice_mask) + normalized_reward * choice_mask + pe_modulate...
*** updated_last_reward_trace = current_last_reward_trace * (1.0 - effective_trace_decay) * (1 - choice_mask) + normalized_reward * choice_mask + pe_modulate...
*** updated_last_reward_trace = current_last_reward_trace * (1.0 - effective_trace_decay) * (1 - choice_mask) + normalized_reward * choice_mask + pe_modulate...
*** updated_last_reward_trace = current_last_reward_trace * (1.0 - effective_trace_decay) * (1 - choice_mask) + normalized_reward * choice_mask + pe_modulate...
*** updated_last_reward_trace = current_last_reward_trace * (1.0 - effective_trace_decay) * (1 - choice_mask) + normalized_reward * choice_mask + pe_modulate...
*** ..._trace = current_last_reward_trace * (1.0 - effective_trace_decay) * (1 - choice_mask) + normalized_reward * choice_mask + pe_modulated_unchosen_trace_update
*** updated_last_reward_trace = current_last_reward_trace * (1.0 - effective_trace_decay) * (1 - choice_mask) + normalized_reward * choice_mask + pe_modulate...
*** unchosen_updated_q_values = (unchosen_q_values_decay_to_average + unchosen_pe_influence + updated_last_reward_trace * 0.05) * (1 - choice_mask)
*** updated_last_reward_trace = current_last_reward_trace * (1.0 - effective_trace_decay) * (1 - choice_mask) + normalized_reward * choice_mask + pe_modulate...
unchosen_updated_q_values = (unchosen_q_values_decay_to_average + unchosen_pe_influence + updated_last_reward_trace * 0.05) * (1 - choice_mask)
*** unchosen_updated_q_values = (unchosen_q_values_decay_to_average + unchosen_pe_influence + updated_last_reward_trace * 0.05) * (1 - choice_mask)
*** unchosen_updated_q_values = (unchosen_q_values_decay_to_average + unchosen_pe_influence + updated_last_reward_trace * 0.05) * (1 - choice_mask)
*** unchosen_updated_q_values = (unchosen_q_values_decay_to_average + unchosen_pe_influence + updated_last_reward_trace * 0.05) * (1 - choice_mask)
*** unchosen_updated_q_values = (unchosen_q_values_decay_to_average + unchosen_pe_influence + updated_last_reward_trace * 0.05) * (1 - choice_mask)
*** unchosen_updated_q_values = (unchosen_q_values_decay_to_average + unchosen_pe_influence + updated_last_reward_trace * 0.05) * (1 - choice_mask)
*** unchosen_updated_q_values = (unchosen_q_values_decay_to_average + unchosen_pe_influence + updated_last_reward_trace * 0.05) * (1 - choice_mask)
*** unchosen_updated_q_values = (unchosen_q_values_decay_to_average + unchosen_pe_influence + updated_last_reward_trace * 0.05) * (1 - choice_mask)
*** unchosen_updated_q_values = (unchosen_q_values_decay_to_average + unchosen_pe_influence + updated_last_reward_trace * 0.05) * (1 - choice_mask)
effective_learning_rate_modulator = jax.nn.softplus(jnp.abs(prediction_error) * 0.75) * 0.3
effective_learning_rate_modulator = jax.nn.softplus(jnp.abs(prediction_error) * 0.75) * 0.3
effective_learning_rate_modulator = jax.nn.softplus(jnp.abs(prediction_error) * 0.75) * 0.3
effective_learning_rate = jnp.clip(base_learning_rate + effective_learning_rate_modulator, 0.01, 0.99)
effective_learning_rate_modulator = jax.nn.softplus(jnp.abs(prediction_error) * 0.75) * 0.3

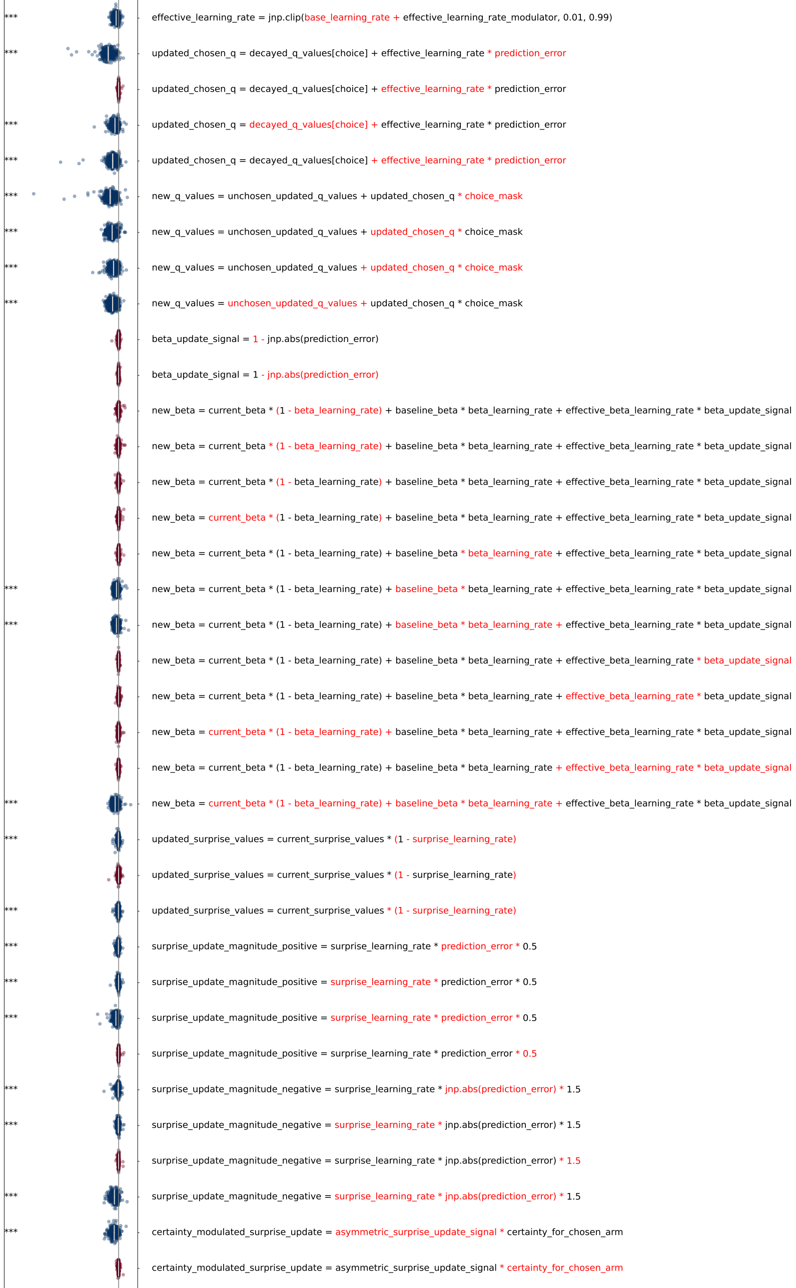

*** effective_learning_rate = jnp.clip(base_learning_rate + effective_learning_rate_modulator, 0.01, 0.99)
*** updated_chosen_q = decayed_q_values[choice] + effective_learning_rate * prediction_error
updated_chosen_q = decayed_q_values[choice] + effective_learning_rate * prediction_error
*** updated_chosen_q = decayed_q_values[choice] + effective_learning_rate * prediction_error
*** updated_chosen_q = decayed_q_values[choice] + effective_learning_rate * prediction_error
*** new_q_values = unchosen_updated_q_values + updated_chosen_q * choice_mask
*** new_q_values = unchosen_updated_q_values + updated_chosen_q * choice_mask
*** new_q_values = unchosen_updated_q_values + updated_chosen_q * choice_mask
*** new_q_values = unchosen_updated_q_values + updated_chosen_q * choice_mask
beta_update_signal = 1 - jnp.abs(prediction_error)
beta_update_signal = 1 - jnp.abs(prediction_error)
new_beta = current_beta * (1 - beta_learning_rate) + baseline_beta * beta_learning_rate + effective_beta_learning_rate * beta_update_signal
new_beta = current_beta * (1 - beta_learning_rate) + baseline_beta * beta_learning_rate + effective_beta_learning_rate * beta_update_signal
new_beta = current_beta * (1 - beta_learning_rate) + baseline_beta * beta_learning_rate + effective_beta_learning_rate * beta_update_signal
new_beta = current_beta * (1 - beta_learning_rate) + baseline_beta * beta_learning_rate + effective_beta_learning_rate * beta_update_signal
new_beta = current_beta * (1 - beta_learning_rate) + baseline_beta * beta_learning_rate + effective_beta_learning_rate * beta_update_signal
*** new_beta = current_beta * (1 - beta_learning_rate) + baseline_beta * beta_learning_rate + effective_beta_learning_rate * beta_update_signal
*** new_beta = current_beta * (1 - beta_learning_rate) + baseline_beta * beta_learning_rate + effective_beta_learning_rate * beta_update_signal
new_beta = current_beta * (1 - beta_learning_rate) + baseline_beta * beta_learning_rate + effective_beta_learning_rate * beta_update_signal
new_beta = current_beta * (1 - beta_learning_rate) + baseline_beta * beta_learning_rate + effective_beta_learning_rate * beta_update_signal
new_beta = current_beta * (1 - beta_learning_rate) + baseline_beta * beta_learning_rate + effective_beta_learning_rate * beta_update_signal
new_beta = current_beta * (1 - beta_learning_rate) + baseline_beta * beta_learning_rate + effective_beta_learning_rate * beta_update_signal
*** new_beta = current_beta * (1 - beta_learning_rate) + baseline_beta * beta_learning_rate + effective_beta_learning_rate * beta_update_signal
*** updated_surprise_values = current_surprise_values * (1 - surprise_learning_rate)
updated_surprise_values = current_surprise_values * (1 - surprise_learning_rate)
*** updated_surprise_values = current_surprise_values * (1 - surprise_learning_rate)
*** surprise_update_magnitude_positive = surprise_learning_rate * prediction_error * 0.5
*** surprise_update_magnitude_positive = surprise_learning_rate * prediction_error * 0.5
*** surprise_update_magnitude_positive = surprise_learning_rate * prediction_error * 0.5
surprise_update_magnitude_positive = surprise_learning_rate * prediction_error * 0.5
*** surprise_update_magnitude_negative = surprise_learning_rate * jnp.abs(prediction_error) * 1.5
*** surprise_update_magnitude_negative = surprise_learning_rate * jnp.abs(prediction_error) * 1.5
surprise_update_magnitude_negative = surprise_learning_rate * jnp.abs(prediction_error) * 1.5
*** surprise_update_magnitude_negative = surprise_learning_rate * jnp.abs(prediction_error) * 1.5
*** certainty_modulated_surprise_update = asymmetric_surprise_update_signal * certainty_for_chosen_arm
certainty_modulated_surprise_update = asymmetric_surprise_update_signal * certainty_for_chosen_arm

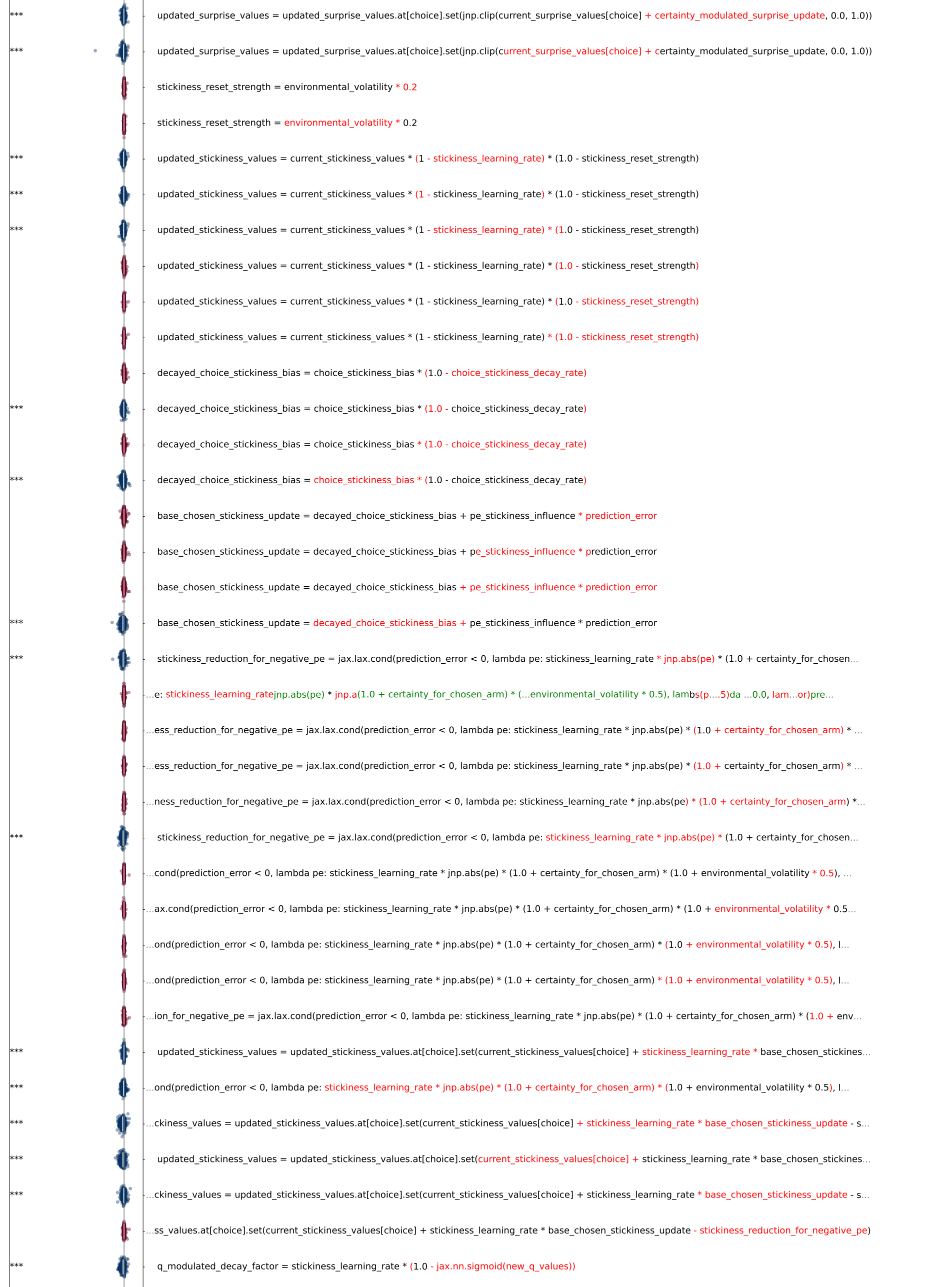
*** updated_surprise_values = updated_surprise_values.at[choice].set(jnp.clip(current_surprise_values[choice] + certainty_modulated_surprise_update, 0.0, 1.0))
*** updated_surprise_values = updated_surprise_values.at[choice].set(jnp.clip(current_surprise_values[choice] + certainty_modulated_surprise_update, 0.0, 1.0))
stickiness_reset_strength = environmental_volatility * 0.2
stickiness_reset_strength = environmental_volatility * 0.2
*** updated_stickiness_values = current_stickiness_values * (1 - stickiness_learning_rate) * (1.0 - stickiness_reset_strength)
*** updated_stickiness_values = current_stickiness_values * (1 - stickiness_learning_rate) * (1.0 - stickiness_reset_strength)
*** updated_stickiness_values = current_stickiness_values * (1 - stickiness_learning_rate) * (1.0 - stickiness_reset_strength)
updated_stickiness_values = current_stickiness_values * (1 - stickiness_learning_rate) * (1.0 - stickiness_reset_strength)
updated_stickiness_values = current_stickiness_values * (1 - stickiness_learning_rate) * (1.0 - stickiness_reset_strength)
updated_stickiness_values = current_stickiness_values * (1 - stickiness_learning_rate) * (1.0 - stickiness_reset_strength)
decayed_choice_stickiness_bias = choice_stickiness_bias * (1.0 - choice_stickiness_decay_rate)
*** decayed_choice_stickiness_bias = choice_stickiness_bias * (1.0 - choice_stickiness_decay_rate)
decayed_choice_stickiness_bias = choice_stickiness_bias * (1.0 - choice_stickiness_decay_rate)
*** decayed_choice_stickiness_bias = choice_stickiness_bias * (1.0 - choice_stickiness_decay_rate)
base_chosen_stickiness_update = decayed_choice_stickiness_bias + pe_stickiness_influence * prediction_error
base_chosen_stickiness_update = decayed_choice_stickiness_bias + pe_stickiness_influence * prediction_error
base_chosen_stickiness_update = decayed_choice_stickiness_bias + pe_stickiness_influence * prediction_error
*** base_chosen_stickiness_update = decayed_choice_stickiness_bias + pe_stickiness_influence * prediction_error
*** stickiness_reduction_for_negative_pe = jax.lax.cond(prediction_error < 0, lambda pe: stickiness_learning_rate * jnp.abs(pe) * (1.0 + certainty_for_chosen...
...e: stickiness_learning_ratejnp.abs(pe) * jnp.a(1.0 + certainty_for_chosen_arm) * (...environmental_volatility * 0.5), lambs(p....5)da ...0.0, lam...or)pre...
...ess_reduction_for_negative_pe = jax.lax.cond(prediction_error < 0, lambda pe: stickiness_learning_rate * jnp.abs(pe) * (1.0 + certainty_for_chosen_arm) * ...
...ess_reduction_for_negative_pe = jax.lax.cond(prediction_error < 0, lambda pe: stickiness_learning_rate * jnp.abs(pe) * (1.0 + certainty_for_chosen_arm) * ...
...ness_reduction_for_negative_pe = jax.lax.cond(prediction_error < 0, lambda pe: stickiness_learning_rate * jnp.abs(pe) * (1.0 + certainty_for_chosen_arm) *...
*** stickiness_reduction_for_negative_pe = jax.lax.cond(prediction_error < 0, lambda pe: stickiness_learning_rate * jnp.abs(pe) * (1.0 + certainty_for_chosen...
...cond(prediction_error < 0, lambda pe: stickiness_learning_rate * jnp.abs(pe) * (1.0 + certainty_for_chosen_arm) * (1.0 + environmental_volatility * 0.5), ...
...ax.cond(prediction_error < 0, lambda pe: stickiness_learning_rate * jnp.abs(pe) * (1.0 + certainty_for_chosen_arm) * (1.0 + environmental_volatility * 0.5...
...ond(prediction_error < 0, lambda pe: stickiness_learning_rate * jnp.abs(pe) * (1.0 + certainty_for_chosen_arm) * (1.0 + environmental_volatility * 0.5), l...
...ond(prediction_error < 0, lambda pe: stickiness_learning_rate * jnp.abs(pe) * (1.0 + certainty_for_chosen_arm) * (1.0 + environmental_volatility * 0.5), l...
...ion_for_negative_pe = jax.lax.cond(prediction_error < 0, lambda pe: stickiness_learning_rate * jnp.abs(pe) * (1.0 + certainty_for_chosen_arm) * (1.0 + env...
*** updated_stickiness_values = updated_stickiness_values.at[choice].set(current_stickiness_values[choice] + stickiness_learning_rate * base_chosen_stickines...
*** ...ond(prediction_error < 0, lambda pe: stickiness_learning_rate * jnp.abs(pe) * (1.0 + certainty_for_chosen_arm) * (1.0 + environmental_volatility * 0.5), l...
*** ...ckiness_values = updated_stickiness_values.at[choice].set(current_stickiness_values[choice] + stickiness_learning_rate * base_chosen_stickiness_update - s...
*** updated_stickiness_values = updated_stickiness_values.at[choice].set(current_stickiness_values[choice] + stickiness_learning_rate * base_chosen_stickines...
*** ...ckiness_values = updated_stickiness_values.at[choice].set(current_stickiness_values[choice] + stickiness_learning_rate * base_chosen_stickiness_update - s...
...ss_values.at[choice].set(current_stickiness_values[choice] + stickiness_learning_rate * base_chosen_stickiness_update - stickiness_reduction_for_negative_pe)
*** q_modulated_decay_factor = stickiness_learning_rate * (1.0 - jax.nn.sigmoid(new_q_values))

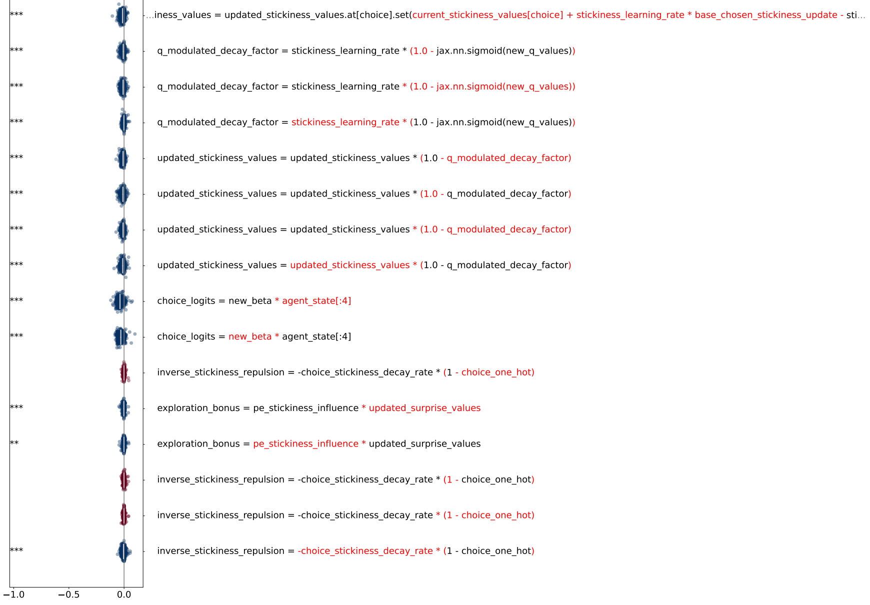

*** …iness_values = updated_stickiness_values.at[choice].set(current_stickiness_values[choice] + stickiness_learning_rate * base_chosen_stickiness_update - sti…
*** q_modulated_decay_factor = stickiness_learning_rate * (1.0 - jax.nn.sigmoid(new_q_values))
*** q_modulated_decay_factor = stickiness_learning_rate * (1.0 - jax.nn.sigmoid(new_q_values))
*** q_modulated_decay_factor = stickiness_learning_rate * (1.0 - jax.nn.sigmoid(new_q_values))
*** updated_stickiness_values = updated_stickiness_values * (1.0 - q_modulated_decay_factor)
*** updated_stickiness_values = updated_stickiness_values * (1.0 - q_modulated_decay_factor)
*** updated_stickiness_values = updated_stickiness_values * (1.0 - q_modulated_decay_factor)
*** updated_stickiness_values = updated_stickiness_values * (1.0 - q_modulated_decay_factor)
*** choice_logits = new_beta * agent_state[:4]
*** choice_logits = new_beta * agent_state[:4]
inverse_stickiness_repulsion = -choice_stickiness_decay_rate * (1 - choice_one_hot)
*** exploration_bonus = pe_stickiness_influence * updated_surprise_values
** exploration_bonus = pe_stickiness_influence * updated_surprise_values
inverse_stickiness_repulsion = -choice_stickiness_decay_rate * (1 - choice_one_hot)
inverse_stickiness_repulsion = -choice_stickiness_decay_rate * (1 - choice_one_hot)
*** inverse_stickiness_repulsion = -choice_stickiness_decay_rate * (1 - choice_one_hot)
−1.0
−0.5
0.0
